# Activation of a gene cluster in *Streptomyces lavendulae* subsp. *lavendulae* CCM 3239 induced strevertene production

**DOI:** 10.1007/s00253-026-13908-9

**Published:** 2026-06-15

**Authors:** Filip Opaterny, Rachel Javorova, Michal Soral, Lubomira Feckova, Dominika Csolleiova, Renata Novakova, Kamila Cholvadtova, Iveta Uhliarikova, Maria Kopacova, Vladimir Patoprsty, Bronislava Rezuchova, Beatrica Sevcikova, Jan Kormanec

**Affiliations:** 1 Institute of Molecular Biology, Slovak Academy of Sciences, Dubravska Cesta 21, 845 51 Bratislava, Slovak Republic; 2 Institute of Chemistry, Slovak Academy of Sciences, 845 38 Bratislava, Slovak Republic

**Keywords:** Antifungal, Biosynthetic gene cluster, Polyene, Strevertene, *Streptomyces*

## Abstract

**Abstract:**

The genome of *Streptomyces lavendulae* subsp. *lavendulae* CCM 3239 contains 36 biosynthetic gene clusters (BGCs) that are cryptic or silent, except for the previously characterized aromatic polyketide auricin. In this work, we present a simple strategy to activate BGCs in this strain by replacing the native promoter with the strong kasOp* promoter via homologous recombination. This approach was validated by strong indigoidine production following insertion of kasOp* upstream of the silent *bpsA* gene. Using this strategy, we activated a silent BGC encoding a type I polyketide synthase (PKS). Interestingly, insertion of kasOp* at different positions within this BGC—upstream of the *SLAV_02840* and *SLAV_02776* genes—led to the production of distinct compounds, both exhibiting polyene spectral features and antifungal activity. Both compounds were isolated, and structural analysis revealed that they were similar to strevertenes; consequently, they were named strevertene A2 and X. A database search indicated that their structures corresponded to the known compounds RKGS-A2215A and AB023a. In silico analysis of the biosynthetic enzymes encoded by this strevertene (*stv*) BGC enabled us to propose a biosynthetic model consistent with the observed induction in the mutant strains *S. lavendulae kasOp::2776* and *S. lavendulae kasOp::2840*. The analysis also suggested that strevertene X is an intermediate in the formation of the final polyene, strevertene A2. Notably, strevertene X exhibited approximately twofold higher antifungal activity than strevertene A2.

**Key points:**

• Strategy for activating BGCs by introducing a strong kasOp* promoter.

• *Efficient activation of the silent bpsA gene for indigoidine.*

• *Activation of silent BGC for type I PKS for polyenes strevertene A2 and X.*

**Supplementary Information:**

The online version contains supplementary material available at 10.1007/s00253-026-13908-9.

## Introduction

Gram-positive bacteria of the genus *Streptomyces* are characterized by a complex process of morphological differentiation, accompanied by physiological differentiation, which involves the production of numerous secondary metabolites with diverse biological activities, including antibiotics, antifungal substances, cytostatics, herbicides, antiparasitic agents, and immunosuppressive compounds. The biosynthetic genes for these secondary metabolites are typically organized in long (approximately 30–200 kb) physical clusters—biosynthetic gene clusters (BGCs)—together with regulatory and resistance genes. This genomic organization facilitates cloning, genetic manipulation, and transfer of BGCs to heterologous *Streptomyces* hosts (Hopwood 2007).

Historically, almost all known antibiotics and other biologically active secondary metabolites from *Streptomyces* were identified through classical screening approaches, commonly referred to as "bioprospecting." Due to the high rate of rediscovery of previously characterized compounds, this approach is now labor-intensive and less effective in discovering new bioactive compounds (Lewis 2013). However, bioinformatic analysis of more than a thousand *Streptomyces* genomes using automated genome mining software such as antiSMASH revealed a large, previously hidden potential for the production of novel secondary metabolites (Blin et al. 2023). On average, each *Streptomyces* genome contains 39 BGCs, most of which are cryptic or silent under standard laboratory conditions (Belknap et al. 2020). Several genetic strategies have been employed to activate the expression of these cryptic or silent BGCs, including overexpression or deletion of regulatory genes, introduction of strong promoters into BGCs, and transfer of BGCs into optimal heterologous *Streptomyces* hosts (Liu et al. 2021; Zhu et al. 2013).

Many antibiotics and other bioactive secondary metabolites belong to the polyketide class. Structurally diverse polyketides are synthesized via repeated decarboxylative Claisen condensation of acyl-CoA precursors catalyzed by polyketide synthases (PKSs). There are two dominant types of PKSs: type I PKSs, which synthesize reduced polyketides (such as macrolides, polyethers, and polyenes), and type II PKSs, responsible for the biosynthesis of aromatic polyketides. Type I PKSs are large, modular, multifunctional proteins that elongate, process, and release the polyketide chain through multiple consecutive condensation cycles. Each module contains a set of catalytic domains—including acyltransferase (AT), ketosynthase (KS), and acyl carrier protein (ACP)—that work together to form a β-ketoester intermediate. Additional domains, such as ketoreductase (KR), dehydratase (DH), and enoylreductase (ER), modify the β-keto group. During biosynthesis, the growing polyketide chain is transferred sequentially from module to module until a complete linear thioester intermediate is released from the final module by a thioesterase (TE). Tailoring enzymes then cyclize and modify the intermediate to produce the final biologically active polyketide (Hertweck 2009). A specific class of polyketide secondary metabolites produced by *Streptomyces* are polyene macrolides, first discovered in the 1950s. Their biosynthesis is conducted by modular type I PKSs, and tailoring enzymes introduce carboxyl groups and mycosamine sugars to the macrolactone ring. Key polyene macrolides include candicidin (FR-008), amphotericin, pimaricin, and nystatin. These compounds, primarily known for their potent antifungal and antiprotozoal properties, are critical for treating severe systemic fungal infections and parasitic diseases (Caffrey et al. 2016). Among polyenes, strevertenes are antifungal pentaene macrolides first isolated from *Streptoverticillium* sp. LL-30F848. Unlike many polyenes, these pentaene macrolides lack the typical hemiketal-tetrahydropyran and aminoglycoside groups but retain a carboxylic acid moiety (Schlingmann et al. 1999). Later, strevertenes were also isolated from *Streptomyces* strains: strevertene A and B from *S. psammotisus* KP1404 (Kim et al. 2011) and strevertene A from *Streptomyces* sp. SZK1 (Ninomiya and Kodani 2013). Strevertene A was identified by high-resolution electrospray ionization mass spectrometry (HR-ESI–MS) after using a reporter-guided strategy to activate incomplete BGC for type I PKS in *S. lavendulae* CGMCC 4.1386 (Li et al. 2018).

The strain formerly known as *S. aureofaciens* CCM 3239, obtained from the Czech Collection of Microorganisms (CCM), Brno, Czech Republic, has been our model strain for over 30 years, primarily in studies of morphological and physiological differentiation. However, the complete genomic sequencing of this strain revealed that its taxonomic classification was incorrect: it is actually *S. lavendulae* subsp. *lavendulae* CCM 3239 (Busche et al. 2018). A type II polyketide BGC, *aur1*, responsible for producing the unique aromatic polyketide antibiotic auricin, has been identified and characterized in this strain (Kormanec et al. 2014; Matulova et al. 2019). Interestingly, this BGC is located not on the chromosome but on the large linear plasmid pSA3239 (Mingyar et al. 2018). Auricin is produced only under specific growth conditions during a narrow time window at the end of the exponential growth phase accompanied by a decrease in pH. Its level declines sharply at later stages due to a pH increase (Kutas et al. 2013). This unusual production profile results from complex regulation by multiple transcriptional regulators (Kormanec et al. 2014; Mingyar et al. 2015; Novakova et al. 2022). Analysis of the CCM 3239 genome using antiSMASH version 7.0 (Blin et al. 2023) identified 36 BGCs: 29 in the genome and 7 on the linear plasmid pSA3239 (Table S1). In addition to the *aur1/aur2* BGC for auricin, the strain produces another silent peptide secondary metabolite, indigoidine (Novakova et al. 2010). Despite testing several liquid culture conditions, no other secondary metabolites were detected, suggesting that the remaining BGCs are apparently silent under the tested conditions (Mingyar et al. 2018).

The present study aimed to establish and optimize a system for activating silent BGCs in *S. lavendulae* subsp. *lavendulae* CCM 3239. Two approaches were tested to stably introduce the strong kasOp* promoter (Wang et al. 2013) upstream of the first biosynthetic gene in selected BGCs via homologous recombination. In both cases, engineered recombinant strains exhibited strong induction of secondary metabolite production. For the *bpsA* gene, encoding the non-ribosomal peptide synthase (NRPS) responsible for the blue pigment indigoidine biosynthesis (Novakova et al. 2010), over 500-fold induction of this conjugated cyclic dipeptide was achieved. For a type I PKS BGC, new polyenes similar to strevertenes were induced depending on the kasOp* insertion site within the BGC. Based on these results, a putative biosynthetic pathway for strevertenes was proposed.

## Materials and methods

### Bacterial strains, plasmids, and culture conditions

Plasmids and strains used in this study are listed in Table S2. Conditions for growth and sporulation of *S. lavendulae* subsp. *lavendulae* CCM 3239 wild-type (WT) and mutant strains have been described by Matulova et al. (2019). Solid Bennet medium (Matulova et al. 2019) was used for propagation of *S. lavendulae* subsp. *lavendulae* CCM 3239. *Streptomyces* strains were cultivated in liquid corn steep liquor-sucrose (CSLS) inoculation medium (McCormick et al. 1959) and in Bennet medium (Matulova et al. 2019). *Escherichia coli* strains were grown in standard Luria-Bertani (LB) medium, and transformation procedures were performed as described by Ausubel et al. (1995). *E. coli* DH5α (ThermoFisher Scientific Inc., Waltham, MA, USA) was used as the host for routine cloning experiments. The non-methylating *E. coli* ET12567/pUZ8002 (Kieser et al. 2000) was used to transfer plasmids from *E. coli* into *Streptomyces* strains by conjugation. When required, media were supplemented with the following antibiotics: ampicillin (Amp), 100 μg/mL (Cat. No. A9518, Sigma-Aldrich, Darmstadt, Germany); kanamycin (Kan), 50 μg/mL (Cat. No. 26899.03, SERVA, Heidelberg, Germany); apramycin (Apr), 50 μg/mL (Cat. No. A2024, Sigma-Aldrich, Darmstadt, Germany); chloramphenicol (Clm), 25 μg/mL (Cat. No. C0378, Sigma-Aldrich, Darmstadt, Germany); and nalidixic acid (NA), 20 μg/mL (Cat. No. N8878, Sigma-Aldrich, Darmstadt, Germany). Strevertene A was obtained from Bioaustralis Fine Chemicals, Smithfield, Australia (Code No. BIA-S2927).

### Recombinant DNA techniques

Gene manipulations in *E. coli* were performed as described by Ausubel et al. (1995). Chromosomal DNA from *S. lavendulae* strains was isolated according to Kormanec et al. (2021). Correct integration of the antibiotic resistance cassette containing the kasOp* promoter into *Streptomyces* chromosomes was verified by Southern-blot hybridization analysis (Ausubel et al. 1995). Southern-blotting was performed using a Hybond N membrane (Roche, Manheim, Germany) and digoxigenin (DIG)-labelled probes prepared according to the standard DIG protocol, with chemiluminescent detection using a DIG Luminescent Detection Kit (Roche, Manheim, Germany). DIG-labelled probes were generated using a DIG DNA Labelling Kit (Roche, Manheim, Germany) with the following DNA fragments: a 0.9-kb *Bgl*II-*Not*I fragment containing part of the *sa7* gene (Probe 1; Fig. S1), a 1.0-kb *Bam*HI fragment containing part of the *Slav_02776* gene (Probe 2; Fig. S2), and a 0.8-kb *Kpn*I fragment containing part of the *Slav_02840* gene (Probe 3; Fig. S2). Nucleotide sequencing was carried out using the ABI PRISM™ Dye Terminator Cycle Sequencing Ready Reaction Kit (Applied Biosystems), and analyzed on an Applied Biosystems model 373 DNA sequencer.

### Construction of pKasOpAmRb and pKasOpAmRT2

The plasmid pAMT1 was first prepared by cloning a 1.4-kb *Eco*RI-*Hin*dIII fragment containing the AprR *aac3(IV)* gene with *oriT* from plasmid pIJ773 (Gust et al. 2003) to LITMUS 28 (New England Biolabs, San Diego, CA, USA) digested with the same enzymes. Next, two complementary primers KasOpDir and KasOpRev (Table S3), containing the kasOp* promoter (Wang et al. 2013), were annealed by mixing 20 pmol of each primer in water, heating at 100 °C for 2 min, and allowing the mixture to cool to room temperature over 15 min. The annealed promoter fragment was cloned into pAMT1 digested with *Acc*65I and *Bam*HI, generating pKasOpAmRb (Fig. S3).

The construction of pKasOpAmRT2 involved multiple steps: (1) The bidirectional terminator *T5* was excised as a 450-bp *Eco*RI-*Hin*dIII (filled in with Klenow enzyme) fragment from plasmid pBSBR5 (Javorova et al. 2024) and inserted into pKasOpAmRb digested with *Nsi*I (filled in with Klenow enzyme) and *Eco*RI, yielding pKasOpAmRT1. (2) A 1.7-kb *Spe*I-*Eco*RV fragment from pGUS (Myronovskyi et al. 2011) was cloned into pAPH3 (Csolleiova et al. 2021) digested with *Xba*I and *Stu*I to generate pAPH4. (3) A 3-kb *Spe*I-*Avr*II (filled in with Klenow enzyme) fragment from pAPH4 was cloned into pSB40N (Kormanec and Sevcikova 2000) digested with *Spe*I and *Eco*RV, resulting in pAPH4B. (4) The AprR *aac3(IV)* gene with *oriT* from plasmid pIJ773 (Gust et al. 2003) was amplified by PCR using high-fidelity Q5 DNA polymerase (New England Biolabs, San Diego, CA) and primers AmR1 and AmR2 to introduce flanking polylinker sites (Table S3). The resulting 1.4-kb DNA fragment was digested with *Spe*I and *Hin*dIII and cloned into pAPH4B digested with the same restriction enzymes, yielding pAMR13. Finally, a 1.9-kb *Bgl*II-*Afl*II fragment from pKasOpAmRT1 was cloned in pAMR13 digested with *Bam*HI and *Afl*II, resulting in pKasOpAmRT2 (Fig. S4a).

### Integration of the kasOp* promoter upstream of the *bpsA* gene in *S. lavendulae* subsp. *lavendulae* CCM 3239 and determination of indigoidine

The mutant carrying an AprR-kasOp* cassette inserted upstream of the *bpsA* gene in *S. lavendulae* subsp. *lavendulae* CCM 3239 was constructed using the PCR-targeted REDIRECT procedure (Gust et al. 2003) with primers BpsAkasDir and BpsAkasRev (Table S3) and plasmid pKasOpAmRb as a template. The resulting PCR-amplified DNA fragment was introduced into the cosmid pCosSA19 (Novakova et al. 2010) in *E. coli* BW25113/pIJ790, generating cosmid pCosSA19-bpsA::kasOp. The verified cosmid pCosSA19-bpsA::kasOp was then transferred through the non-methylating *E. coli* ET12567/pUZ8002 strain and introduced into *S. lavendulae* subsp. *lavendulae* CCM 3239 by conjugation (Kieser et al. 2000) with AprR selection. Double crossover mutants were screened for AprR and Kan sensitivity. Four independent colonies were obtained, resulting in mutant strains *S. lavendulae kasOp::bpsA1–4.* Correct insertion of the cassette in the chromosome was confirmed by Southern-blot hybridization of chromosomal DNA (Fig. S1). All clones exhibited similar phenotypes.

Extraction and quantification of indigoidine were performed as described previously (Knirschova et al. 2015). Briefly, spores from the four independent *S. lavendulae kasOp::bpsA* mutant strains and the WT strain were inoculated into 2 ml CSLS inoculation medium and incubated at 28 ºC for 24 h to reach stationary phase. 0.2 ml of each homogenous culture was transferred into 20 ml of Bennet medium and grown on an orbital shaker at 28 ºC and 160 rpm for 22 h. One ml of the resulting culture was collected, centrifuged at 21 000 × *g* for 10 min, washed with water, and the cell pellets were weighed and stored at − 20 °C for 2 h. The thawed cells were resuspended in 0.5 ml dimethylsulfoxide (DMSO) to extract indigoidine. The extract was clarified by centrifugation (10 min at 21 000 × *g*) and the absorption of the blue pigment in the supernatant was measured at 613 nm (OD_613_). Indigoidine production was expressed as OD_613_ per mg of wet bacterial cells and reported as the mean ± standard deviation from four independent strains. Statistical significance was evaluated using a two-tailed Student’s t-Test.

### Integration of the kasOp* promoter upstream of the *SLAV_02776* and *SLAV_02840* genes in *S. lavendulae* subsp. *lavendulae* CCM 3239

Mutants carrying a *T5*-AprR-kasOp* cassette upstream of the *Slav_02776* and *Slav_02840* genes in the silent type I PKS BGC 2 (Fig. S4) of *S. lavendulae* subsp. *lavendulae* CCM 3239 were constructed as follows: For *Slav_02776*, a 5-kb DNA fragments encompassing the downstream region of the gene (Fig. S4b) was amplified using primers 2776BsrG and 2776Afl (Table S3), introducing *Bsr*GI and *Afl*II restriction sites. The upstream region of the *Slav_02776* gene (Fig. S4b) was amplified with primers 2776Spe and 2776Mfe (Table S3), introducing *Spe*I and *Mfe*I sites. The downstream fragment was digested with *Bsr*GI and *Afl*II, and the upstream fragment with *Spe*I and *Afl*II, and both fragments were cloned into pKasOpAmRT2 digested with the same enzymes. The resulting plasmid, pKasOp-2776 (Fig. S4b), was verified by nucleotide sequencing using primers neoRseq1, AmRseq1, mut80, and T5r (Table S3). For *Slav_02840*, a 5-kb DNA fragments covering the downstream region of the gene was amplified with primers 2840Afl2 and 2840Eco (Table S3), introducing *Afl*II and *Eco*RI sites, while the upstream region was amplified with primers 2840Spe and 2840Mfe (Table S3), introducing *Spe*I and *Mfe*I sites. Both DNA fragments were digested with the respective enzymes and cloned into pKasOpAmRT2 digested with the same enzymes. The resulting plasmid, pKasOp-2840 (Fig. S4c), was verified by nucleotide sequencing using the same set of primers: neoRseq1, AmRseq1, mut80, and T5r primers (Table S3). Plasmids pKasOp-2776 and pKasOp-2840 were transferred through the non-methylating *E. coli* ET12567/pUZ8002 strain and introduced into *S. lavendulae* subsp. *lavendulae* CCM 3239 by conjugation (Kieser et al. 2000) with AprR selection. Double crossover mutants were screened for AprR and Kan sensitivity. Three independent colonies were obtained from each construct, resulting in the mutant strains *S. lavendulae kasOp::2776–1, −2, −3* and *S. lavendulae kasOp::2840–1, −2, −3.* Correct integration of the cassette into the chromosome was confirmed by Southern-blot hybridization of chromosomal DNA (Fig. S2). All clones exhibited similar phenotypes. Genomic sequence verification was performed by nucleotide sequencing of a 3-kb PCR fragment amplified with primers 2776seqR and 2815seqD for *kasOp-2776* strains, or with primers 2840seqR and 2845seqD for *kasOp-2840* strains, using the same primers for sequencing (Table S3).

### Analysis of secondary metabolite production

A small aliquot of spores from each *Streptomyces* strain was spread onto a defined area (0.7 × 0.7 cm) of solid Bennet medium and incubated for 13-days to allow sporulation. A piece of agar from the sporulated cultures was then transferred into 100 mL Erlenmeyer flasks containing 10 mL CSLS inoculation medium and incubated on a rotatory shaker at 160 rpm at 28 °C for 2 days. Next, 2 ml of this seed culture was inoculated into a 500 mL Erlenmeyer flask containing 50 ml of Bennet medium and incubated under the same conditions. After 24 h, 48 h, 72 h, and 96 h, 5 ml culture aliquots were collected, extracted twice with an equal volume of n-butanol, dried under a vacuum, and finally dissolved in 100 µL methanol. Aliquots of 10 µL were analyzed by TLC followed by biochromatography (Novakova et al. 2022) using sensitive indicator strains: *Bacillus subtilis*, *E. coli*, and *Saccharomyces cerevisiae*. Another 10 µL aliquot was analyzed by HPLC using an OmmiSpher 5 C18 column (5 µm, 250 × 4.6 mm; Varian, Lake Forest, CA, USA), eluted with a 30-min linear gradient of acetonitrile (ACN) from 20 to 100% in 0.5% (v/v) acetic acid in water at a flow rate of 1 mL/min. HPLC analysis was performed on a Shimadzu LC-10A HPLC system equipped with a combined SPD-20A UV–VIS detector (detection at 254 and 350 nm) and an SPD-M14 PDA detector (Shimadzu, Kyoto, Japan). The desired peaks were collected and analyzed by HR-ESI–MS using an Orbitrap Elite Mass Spectrometer (Thermo Scientific, Waltham, MA, USA) in both positive and negative ionization mode. Strevertenes were quantified by comparing the peak area at 350 nm to that of a standard strevertene A solution of known concentration (Bioaustralis Fine Chemicals). 1 μg was analyzed under the same conditions by HPLC.

### Gene expression analysis by Reverse transcription-PCR (RT-PCR)

The growth conditions described above were used for RNA isolation. All three strains, *S. lavendulae* subsp. *lavendulae* CCM 3239, *S. lavendulae kasOp::2840*, and *S. lavendulae kasOp::2776* were grown in 50 ml of Bennet medium for 72 h. Total RNA from the mycelium was isolated according to a previously described procedure (Kormanec 2001). The isolated total RNA was treated with RNase-free DNase (Cat. No. 79254, Qiagen, Hilden, Germany) to remove contaminating DNA. The concentration and purity of RNA was determined by measuring the optical density (OD) at 260 nm/280 nm. RT-PCR was performed with the Qiagen OneStep RT-PCR kit (Cat. No. 210212, Qiagen) according to the manufacturer’s instructions. Reaction mixtures contained 10 pmol of each primer and 200 ng of total RNA as a template in a total volume of 20 ml. DMSO (final 5%, v/v) was used in all PCR reactions along with RNAse Inhibition (40 U/reaction, Cat. No. Y9240L, Qiagen). The conditions were as follows: for cDNA synthesis, 50 °C for 30 min, then 97 °C for 15 min; for amplification, 30 cycles at 97 °C for 30 s, 55 °C for 30 s, and 72 °C for 1 min, and final extension at 72 °C for 5 min. Primers (20–22-mers; average T_m_ value 60 ºC) were designed using Oligo Analyzer software v 1.0 to generate PCR products of approximately 500 bp in length. The primers used to amplify the respective genes were as follows:(Table S3): *Slav_02840* gene (454-bp product with primers 2840dir and 2840rev); *Slav_02825* gene (454-bp product with primers 2825dir and 2825rev); *Slav_02815* gene (477-bp product with primers 2815dir and 2815rev); *Slav_02776* gene (508-bp product with primers 2776dir and 2776rev). The *hrdB* gene (amplified 513-bp product with the primers hrdBdir and hrdBrev, Table S3) encoding the housekeeping sigma factor of RNA polymerase was used as an internal control to normalize RNA samples. It is expressed at a constant level (Kormanec and Farkasovsky 1993). The products after RT-PCR reactions were separated by electrophoresis on a 2% (w/v) agarose gel and visualized by staining with ethidium bromide. The RT-PCR experiments were performed three times for each primer pair. Negative-control experiments were performed under the same conditions without the presence of reverse transcriptase to confirm that the amplified products originated from RNA templates and not from contaminating DNA.

### Isolation of strevertene X and A2

To isolate strevertene X, the growth conditions described above were used. *S. lavendulae kasOp::2776* strain was inoculated into 2 × 10 mL of CSLS inoculation medium and grown for 2 days. Next, 2 mL of this seed culture was inoculated into 8 × 50 mL of Bennet medium and grown for 72 h. The pooled 400 mL culture was extracted twice with 200 mL of n-butanol, and the extract was evaporated under vacuum. The resulting yellow-brown pellet was dissolved in 8 mL of 9:1 dichloromethane: methanol and purified through a 20 mL silica gel flash column equilibrated with the same solvent. The first fraction (200 mL) with a pale-yellow color was concentrated and dissolved in 1 mL of methanol.

0.2 mL portions of this solution were successively purified by semi-preparative reversed-phase HPLC using an OmniSpher C18 column (5 μm, 250 × 10 mm; Varian, Lake Forest, CA, USA) with isocratic elution of 40% ACN and 0.5% acetic acid in water at a flow rate of 4 mL/min (VIS detection at 350 nm). The major peak corresponding to strevertene X was collected, yielding 6 mg. The purity and yield of strevertene X were estimated by analytical HPLC as described above.

To isolate strevertene A2, the growth conditions described above were used. *S. lavendulae kasOp::2840* strain was inoculated into 2 × 10 mL of CSLS inoculation medium and grown for 2 days. Next, 2 mL of this seed culture was inoculated into 8 × 50 mL of Bennet medium and grown for 72 h. The pooled 400 mL culture was extracted twice with 200 mL of n-butanol, and the extract was evaporated under vacuum. The yellow–brown pellet was dissolved in 16 mL of water and subjected to C18 reversed-phase silica gel (Sigma-Aldrich, Darmstadt, Germany) flash column chromatography (4 mL column equilibrated in water). The column was gradually eluted with 20 mL of water, followed by 10, 20, 30, and 40% (v/v) ACN in water, and finally with pure ACN. The fraction eluted with 20% ACN contained strevertene A2. This fraction was diluted with water to 10% ACN and further purified through two Bond Elut columns (500 mg; Agilent Technologies, Santa Clara, CA, USA) equilibrated with water. The columns were washed with 4 mL of 10% ACN and eluted with 4 mL of 100% methanol. The concentrated 0.2 mL fraction was successively purified by semi-preparative reversed-phase HPLC using an OmniSpher C18 column (5 μm, 250 × 10 mm; Varian, Lake Forest, CA, USA) with isocratic elution of 40% ACN and 0.5% acetic acid in water at a flow rate of 4 mL/min (VIS detection at 350 nm). The major peak corresponding to strevertene A2 was collected, yielding 2.5 mg. The purity and yield of strevertene A2 were estimated by analytical HPLC as described above.

### NMR analysis

The NMR spectra of strevertene A2 and strevertene X were recorded on an Avance III HD 600 MHz NMR spectrometer (Bruker BioSpin, Germany) equipped with a TCI H-C/N-D-05-Z helium–cooled cryoprobe. For NMR sample preparation, approximately 3 mg of the studied compounds were dissolved in 180 μL of the chosen perdeuterated solvent—CD_3_OD (99.96% D, Eurisotop, France) or DMSO-*d*6 (99.9% D, Merck, Germany)—and transferred into a 3 mm NMR tube. A standard series of NMR experiments was conducted, including: ^1^H, ^13^C, ^1^H-^1^H Correlation Spectroscopy (COSY), ^1^H-^13^C Heteronuclear Single-Quantum Coherence (HSQC), ^1^H-^13^C Heteronuclear Multiple Bond Correlation (HMBC), selective 1D excitation with the utilization of double pulsed field gradient spin echo (dpfgse), selective 1D Total Correlation Spectroscopy (1D TOCSY) with a stepwise prolongation of the duration of the spin lock (mixing time), Nuclear Overhauser Effect Spectroscopy (NOESY), and Heteronuclear Single-Quantum Coherence Total Correlation Spectroscopy (HSQC-TOCSY) measurements. The chemical shift axes were calculated with tetramethylsilane (TMS) serving as the reference (*δ*_H_ = *δ*_C_ = 0 ppm); in the case of spectra obtained from samples dissolved in CD_3_OD, the ^1^H and ^13^C chemical shift scales were then correctly shifted by using the central peaks of the multiplets of the residual non-perdeuterated solvent (CD_2_HOD: *δ*_H_ = 3.31 ppm) and the perdeuterated solvent (CD_3_OD: *δ*_C_ = 49.000 ppm) as secondary references, respectively. Unless otherwise specifically mentioned, the presented data were obtained from spectra measured at 25 °C.

### MIC determination

The minimum inhibitory concentration (MIC) of the analyzed strevertenes against the sensitive yeast strain *Saccharomyces cerevisiae* BY4742 was determined using a conventional broth microdilution assay in 96-well plates. Compounds were dissolved in methanol to a final concentration of 1 mg/mL. Yeast was grown in liquid YPD medium (1% yeast extract, 2% peptone, 2% glucose, pH 7) for 16 h at 30 °C with shaking at 160 rpm. The culture was then diluted 500-fold in fresh YPD. A similar dilution was prepared in the presence of the highest compound concentration (20 μg/mL). Serial dilutions of each compound were prepared by mixing the two cultures, and 150 μL aliquots were dispensed into 96-well plates. Final compound concentrations were 20, 10, 5, 2.5, 1.25, 0.625, and 0.3125 μg/mL. Methanol alone was used as a negative control. Plates were incubated at 30 °C with shaking at 160 rpm, and A_600_ was measured at 1 h intervals using a Synergy HT microplate reader (BioTek). All experiments were performed in triplicate.

### Bioinformatic analysis

The whole-genome sequence of *S. lavendulae* subsp. *lavendulae* CCM 3239 can be found in GenBank (http://www.ncbi.nlm.nih.gov/genbank/) under accession numbers CP024985 (chromosome) and CP024986/KJ396772 (linear plasmid pSA3239) (Busche et al. 2018). Secondary metabolite BGCs were identified through antiSMASH version 7.0 (Blin et al. 2023). Manual cluster analysis was performed using BLAST (Altschul et al. 1990).). Protein domains were analyzed via multiple sequence alignment using Clustal X (Thompson et al. 1997), and the results were visualized using Boxshade (https://junli.netlify.app/apps/boxshade/). Known BGCs for various compounds were studied using the MIBiG repository (Medema et al. 2015).

## Results

### Activation of the indigoidine biosynthetic gene bpsA

**Fig. 1:**
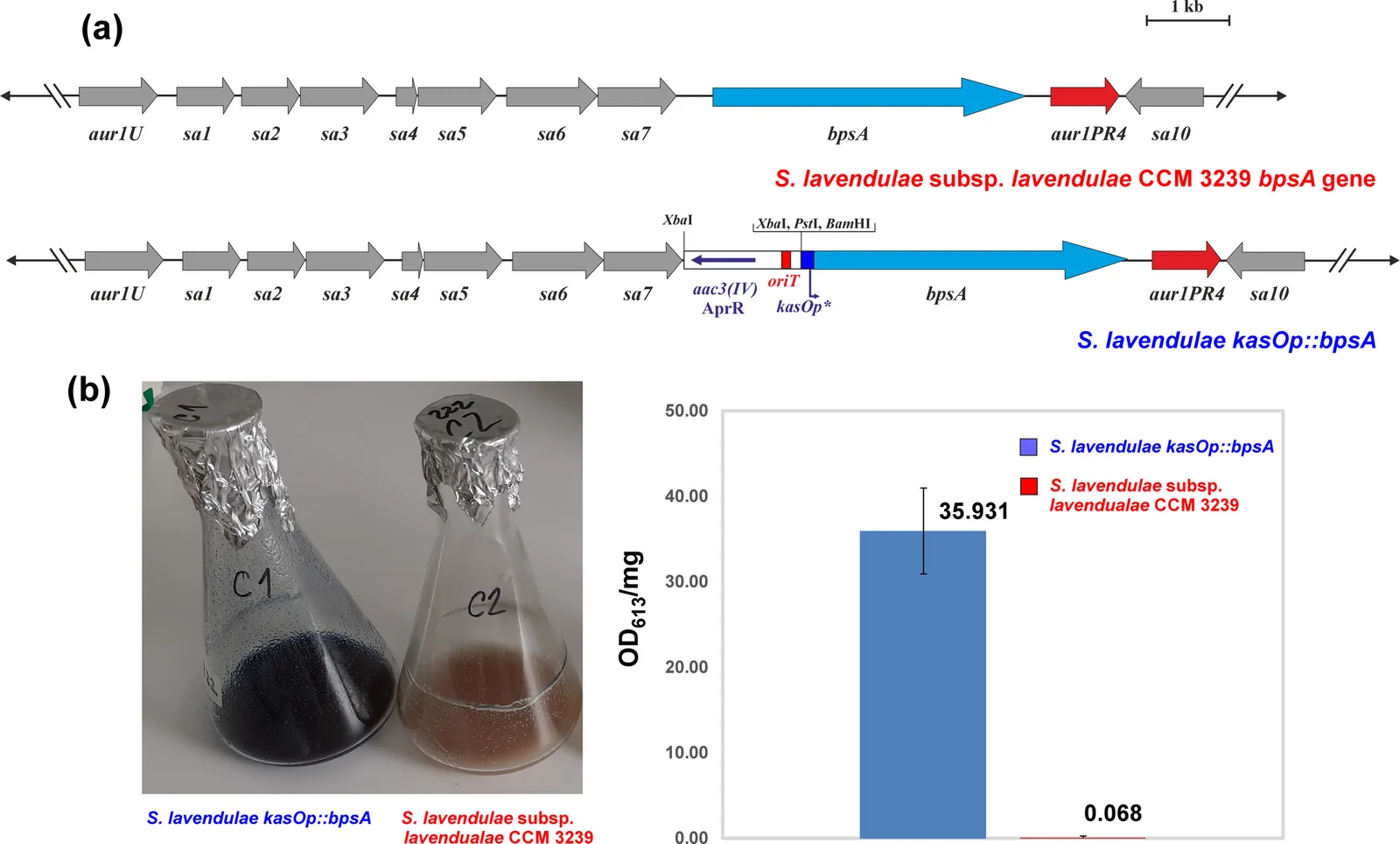
Activation of the silent *bpsA* gene in *S. lavendulae* subsp. *lavendulae* CCM 3239 by insertion of the strong kasOp* promoter upstream of the gene in the genome. **a** Scheme of the WT and mutated genomic regions showing integration of the AprR-kasOp* cassette upstream of the *bpsA* gene. Thick arrows indicate the direction and size of genes; the blue arrow represents the *bpsA* gene and the red arrow the regulatory gene. The blue box with a bent arrow indicates the position of the kasOp* promoter. Gene labeling is based on the sequence with GenBank accession no. KJ396772. **b** Production of indigoidine in the WT strain and the *S. lavendulae kasOp::bpsA* mutant. Indigoidine levels (A₆₁₃/mg) were measured after 22 h of growth in liquid Bennet medium as previously described (Knirschova et al. 2015). Each point represents the mean of four assays, and error bars indicate the standard deviation

The biosynthesis of secondary metabolites in streptomycetes is predominantly controlled at the transcriptional level. Artificial culture conditions of *Streptomyces* strains can lead to transcriptional silencing of BGCs, as the signals and environmental conditions in their natural habitat differ from those in standard liquid culture used for metabolite isolation. One approach to activate these BGCs is the insertion of well-characterized strong promoters upstream of biosynthetic or regulatory genes within silent BGCs (Ji et al. 2024). To test this strategy, we constructed a cassette containing the strong kasOp* promoter (Wang et al. 2013) and the apramycin resistance (AprR) gene, resulting in plasmid pKasOpAmRb (Fig. S3, Table S2). As a proof-of-concept, we targeted the known silent *bpsA* gene, which encodes an NRPS responsible for the synthesis of the blue pigment indigoidine (Novakova et al. 2010). Indigoidine can be readily quantified (Knirschova et al. 2015). The *bpsA* promoter region was replaced by the AprR-kasOp* cassette using the PCR-targeted REDIRECT strategy (Gust et al. 2003), with pKasOpAmRb as a template and primers BpsAkasDir and BpsAkasRev (Fig. S3) (Table S3). The PCR-amplified cassette was inserted into cosmid pCosSA19 (Novakova et al. 2010), generating pCosSA19-*bpsA*::kasOp, which was subsequently used for conjugation into *S. lavendulae* subsp. *lavendulae* CCM 3239 to produce a double crossover mutant (Fig. 1a). Four Apr-resistant clones were identified, and correct integration was confirmed by Southern-blot hybridization (Fig. S1). Phenotypic analysis of the resulting *S. lavendulae kasOp::bpsA* mutants revealed dramatic overproduction of indigoidine compared to the wild-type (WT) strain. The mutant produced over 500-fold higher levels of indigoidine than WT (Fig. 1b), confirming the efficient activation of the indigoidine BGC using this system. However, this approach requires a cosmid library of the strain under investigation and may influence transcription of neighboring genomic regions due to expression of the AprR *“aac3(IV)”* gene.

### Activation of a type I PKS BGC

**Fig. 2:**
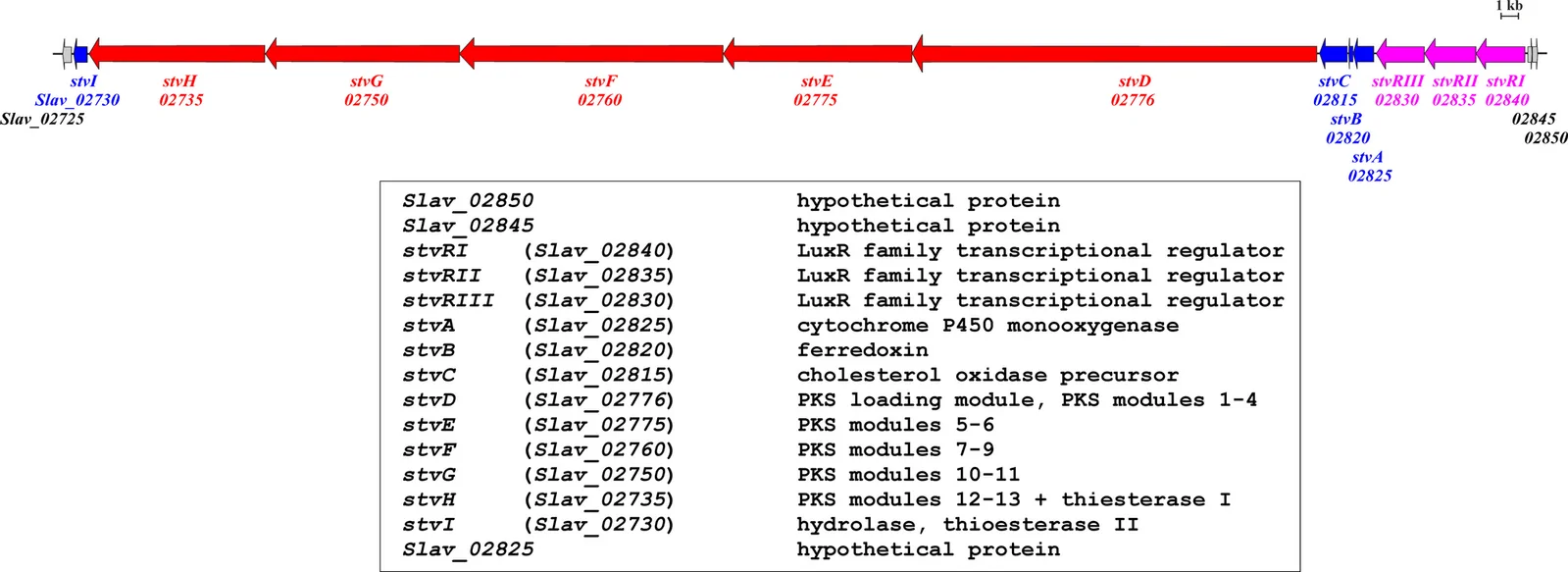
Organization of the strevertene A2 (*stv*) BGC in *S. lavendulae* subsp. *lavendulae* CCM 3239. The predicted functions of the deduced gene products are indicated below the scheme. Thick arrows indicate the direction and size of genes; red arrows represent type I PKS biosynthetic genes, blue arrows tailoring biosynthetic genes, and pink arrows regulatory genes. Gene labeling is based on the *S. lavendulae* subsp. *lavendulae* CCM 3239 genomic sequence (GenBank accession no. CP024985). The complete sequence of the *stv* BGC with annotation of all genes is shown in Fig. S49

We next targeted a cryptic/silent type I PKS BGC (BGC 2, Table S1) for activation by kasOp* promoter insertion upstream of either the *SLAV_02840* gene, encoding a LAL family transcriptional activator, or the *SLAV_02776* gene, encoding a type I PKS biosynthetic enzyme. The *SLAV_02776* gene is the first gene in a large operon containing type I PKS genes (Fig. 2). To prevent transcriptional read-through from surrounding regions, we constructed a new plasmid, pKasOpAmRT2, containing a bidirectional terminator *T5* (Javorova et al. 2024) (Fig. S4). DNA fragments flanking the promoter regions of both target genes were amplified by PCR and cloned into pKasOpAmRT2, resulting in plasmids pKasOp-2776 and pKasOp-2840 (Fig. S4). These constructs were integrated into the chromosome via homologous recombination, yielding the AprR strains *S. lavendulae kasOp::2776* and *kasOp::2840*. Correct integration was verified by Southern-blot hybridization (Fig. S2) and nucleotide sequencing of PCR-amplified spanning regions (Fig. S5). Transcriptional analysis of four selected genes from BGC2 (*Slav-02840, Slav_02825, Slav_02815, Slav_02776*) in WT, *S. lavendulae kasOp::2840* and *kasOp::2776* mutants was performed by RT-PCR using RNA isolated after 72 h, when both strevertene A2 and X production was maximal. The *hrdB* gene (*Slav_10790*) encoding the housekeeping sigma factor of RNA polymerase, which is constitutively expressed during growth (Kormanec and Farkasovsky 1993) was used as an internal control to normalize RNA samples. The results showed that the genes were expressed at very low levels in the WT strain and were differentially activated after insertion of the kasOp* promoter upstream of the *Slav-02840* and *Slav_02776* genes (Fig. S6).

**Fig. 3:**
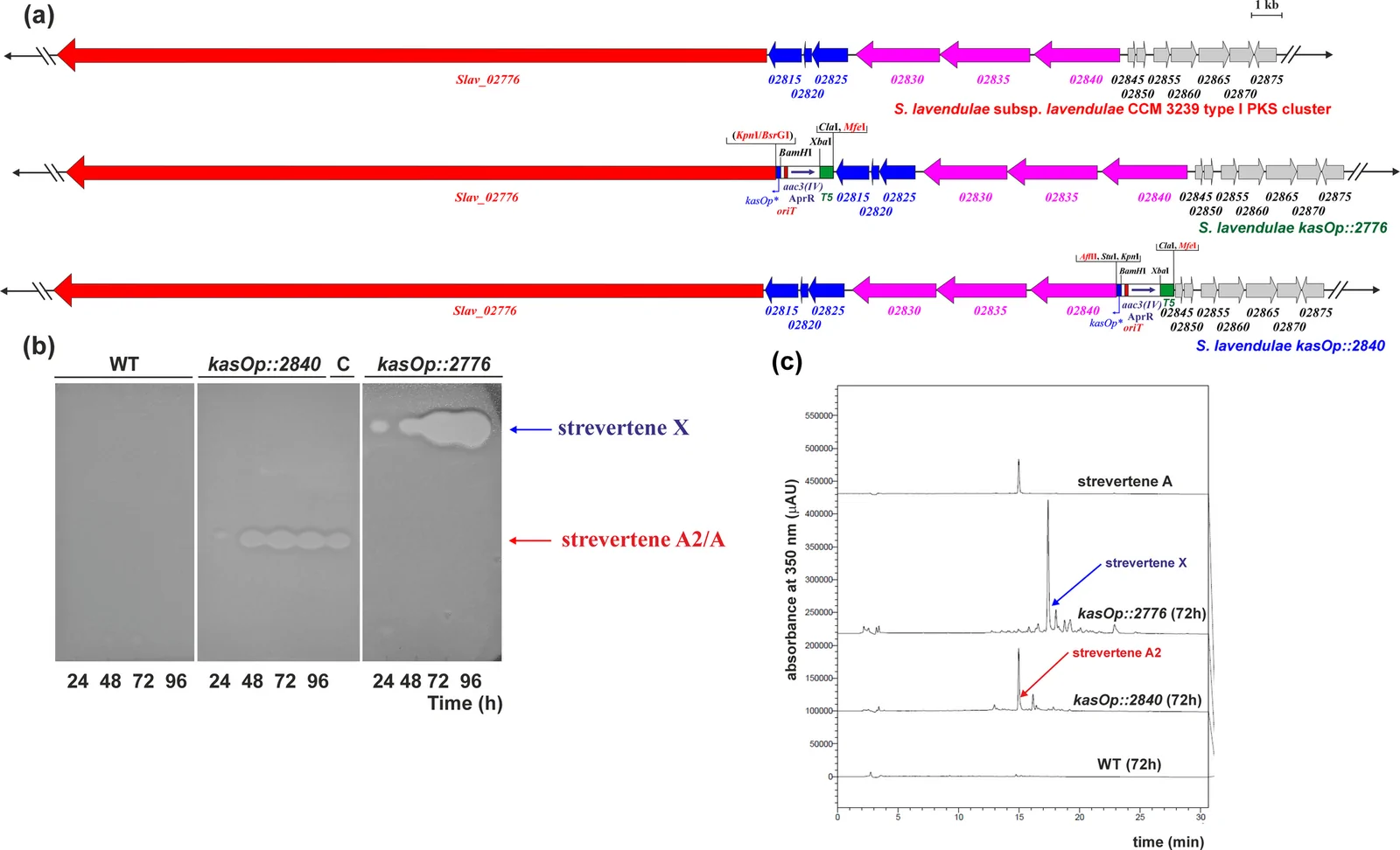
Activation of the silent *stv* BGC for strevertene A2 production in *S. lavendulae* subsp. *lavendulae* CCM 3239 by insertion of the strong kasOp* promoter upstream of the *Slav_02776* and *Slav_02840* genes in the genome. **a** Scheme of the WT and mutated genomic regions showing integration of the AprR-kasOp* cassette upstream of the *Slav_02776* and *Slav_02840* genes. Thick arrows indicate the direction and size of genes; red arrows represent type I PKS biosynthetic genes, blue arrows tailoring biosynthetic genes, and pink arrows regulatory genes. Blue boxes with bent arrows indicate the position of the kasOp* promoter. Gene labeling is based on the *S. lavendulae* subsp. *lavendulae* CCM 3239 genomic sequence (GenBank accession no. CP024985). **b** Detection of strevertenes in 10 μL ethyl acetate extracts from *S. lavendulae* subsp. *lavendulae* CCM 3239 (WT), the *kasOp::2840* mutant, and the *kasOp::2776* mutant strains grown in Bennet medium to the indicated time points. **a** TLC analysis followed by bioautography using a yeast test strain, as described in Experimental procedures. Lane C contains the strevertene A standard (Bioaustralis). **c** HPLC analysis of 10 μL ethyl acetate extracts from the indicated strains grown for 72 h in Bennet medium (see Materials and methods for details). Metabolite elution was monitored at 350 nm. For comparison, 1 μg of strevertene A (Bioaustralis) was analyzed under identical conditions. Arrows indicate the positions of strevertene peaks

Phenotypic analysis of the *S. lavendulae kasOp::2840* mutant using thin-layer chromatography (TLC), biochromatography, and high-performance liquid chromatography (HPLC) revealed overproduction of a yellow compound inhibiting yeast growth (Fig. 3), with no inhibition observed for *B. subtilis* or *E. coli* (data not shown). The UV spectrum exhibited absorption maxima at 319, 334, and 351 nm (Fig. S7), consistent with conjugated pentaene macrolides. Comparison with strevertene A (Schlingmann et al. 1999) (Bioaustralis Fine Chemicals, Smithfield, Australia) showed identical TLC Rf (0.4), HPLC Rt (15 min), and spectral properties (Fig. S7).

HR-ESI–MS showed an intense molecular ion peak at *m/z* 603.3139 [M + Na]^+^ and a molecular ion peak at *m/z* 579.3180 [M − H]^−^, which were almost identical to those observed for strevertene A (Fig. S8). These values, after calculation, indicate a molecular mass of 580.3246/580.3253 corresponding to the molecular formula C_31_H_48_O_10_ (calculated 580.3247), which is identical to that of strevertene A. Production was scaled up for isolation, and nuclear magnetic resonance (NMR) analysis (Figs. S9–S24; Table S4) confirmed that the compound is a strevertene, with slight differences in hydroxyl group positions compared to strevertene A (Fig. 5). Details about the NMR structural analysis are in the Supporting Information file. This compound was named strevertene A2. Literature comparison revealed its structure matches RKGS-A2215A from *S. lavendulae* FRI-5 (Yoshioka et al. 2021).

**Fig. 4:**
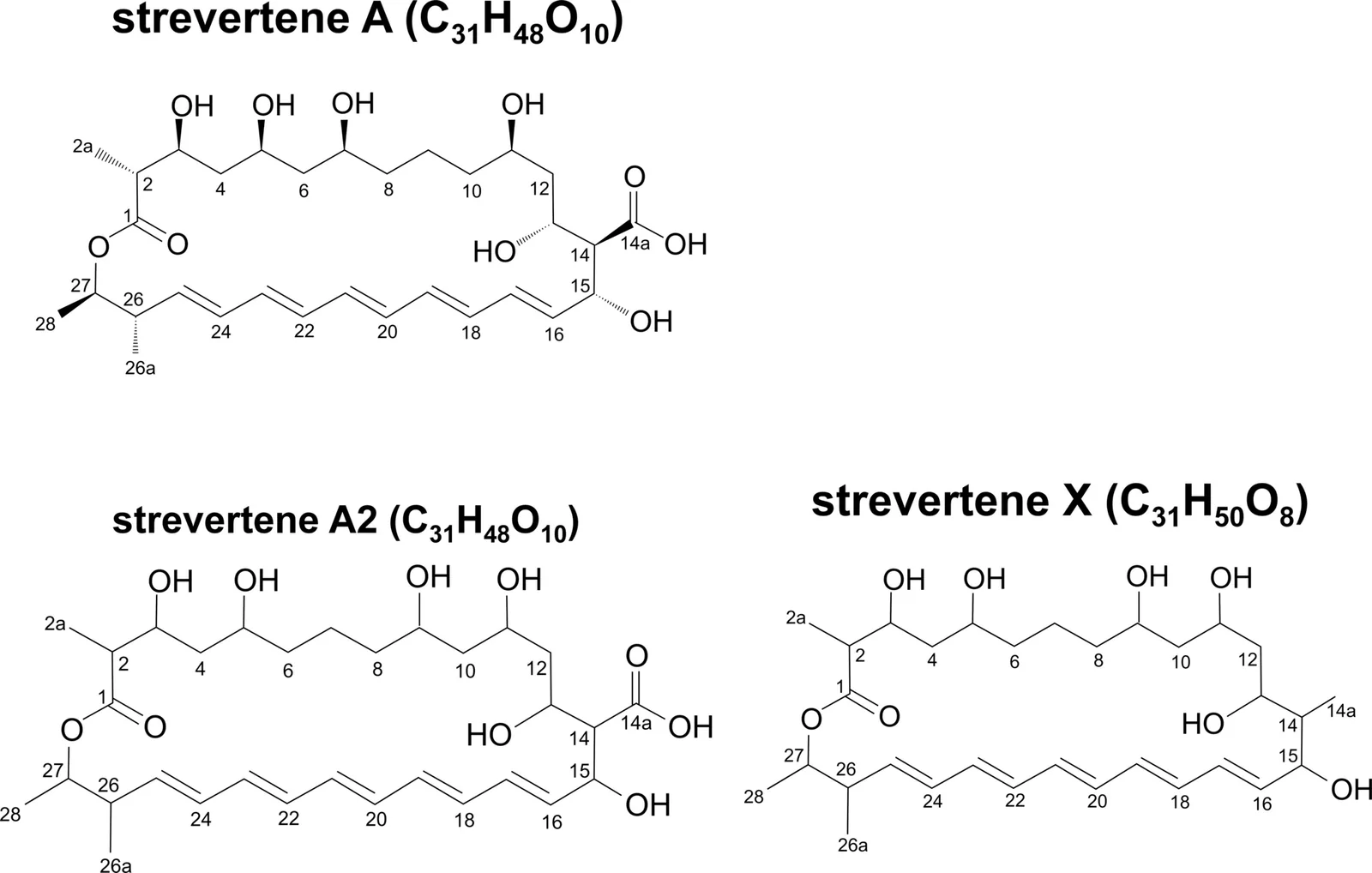
Chemical structures of strevertenes

**Fig. 5:**
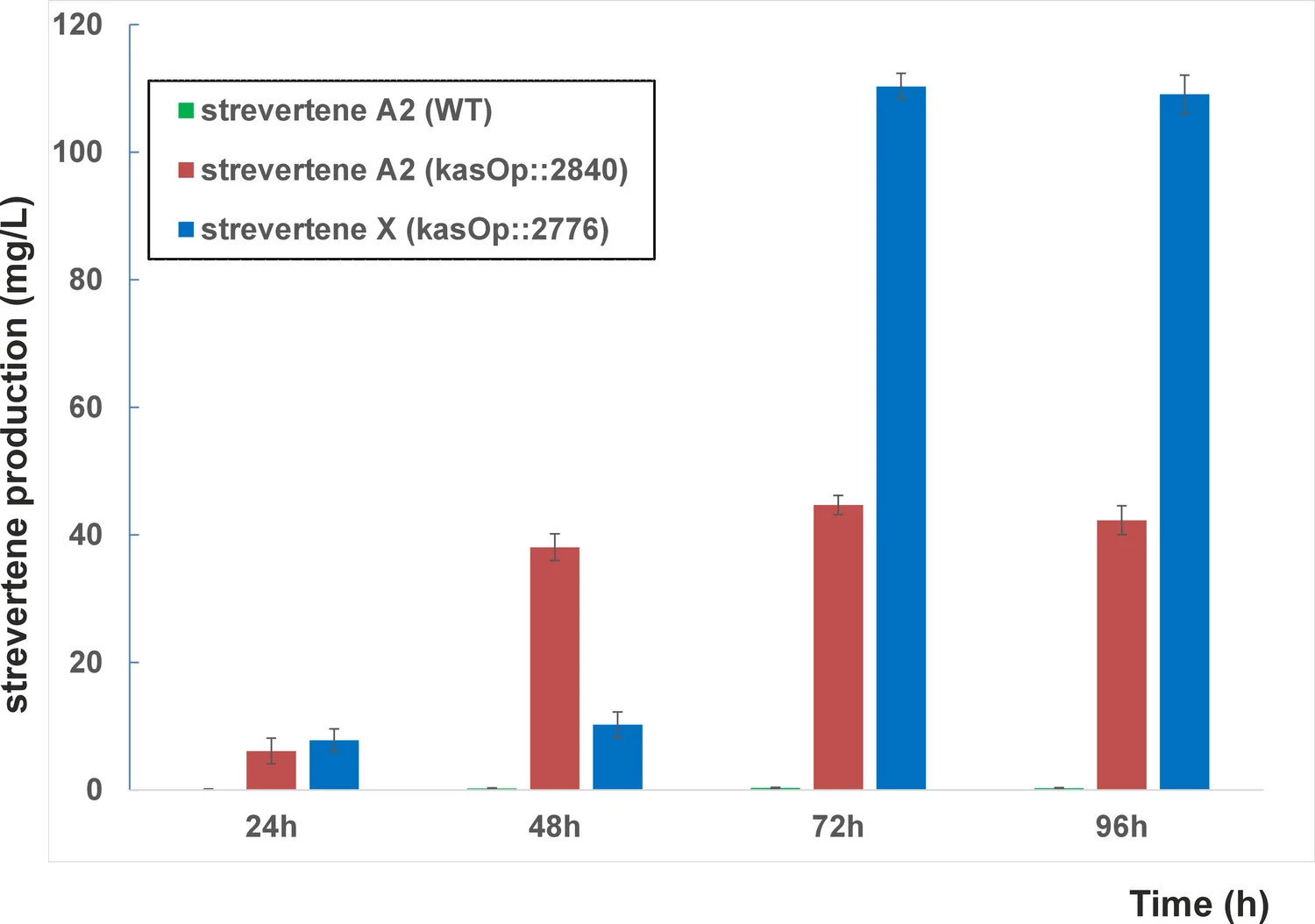
Production of strevertene A2 and strevertene X in *S. lavendulae* subsp. *lavendulae* CCM 3239 (WT), the *kasOp::2840* mutant, and the *kasOp::2776* mutant strain grown in Bennet medium at the indicated time points. The amounts of compounds were determined in the culture broths by HPLC as described in Materials and methods. Each value represents the mean of three assays, and the error bars indicate the standard deviation

The *S. lavendulae kasOp::2776* mutant overproduced a similar yellow compound with different TLC Rf (0.82) and HPLC Rt (17.3 min) values (Fig. 3). Its spectral properties were comparable to those of strevertene A (Fig. S7). However, HR-ESI–MS showed molecular ion peaks at *m/z* 573.3395 [M + Na]^+^ and *m/z* 549.3431 [M − H]^−^ (Fig. S8), indicating a molecular mass of 550.3502/550.3503, corresponding to the molecular formula C_31_H_50_O_8_ (calculated 550.3506). NMR analysis (Figs. S25–S38; Table S5) indicated that the carboxyl group at position 14a is replaced by a methyl group (Fig. 4). This compound was named strevertene X, and literature comparison showed it matches AB023a from *Streptomyces* sp. SD581 (Bortolo et al. 1993). The yields of strevertene A2 and X were maximal after 72 h (Fig. 5), 44.7 ± 1.5 mg/L and 110.3 ± 2.0 mg/L, respectively. Strevertene X was not identified in the WT strain, the yield of strevertene A2 was maximal after 72 h and amounted 0.34 ± 0.1 mg/L.

These findings demonstrate that insertion of kasOp* at different positions within the type I PKS BGC 2 activates distinct polyene compounds. When kasOp* is inserted upstream of *SLAV_02840*, the final polyene strevertene A2 is produced, likely through activation of the LAL family transcriptional activator and downstream genes, or directly activating the entire BGC, as all biosynthetic genes are co-directional. Transcriptional data indicate that insertion of the kasOp* promoter into this region resulted in induced expression of all four genes in the operon (Fig. S6). Insertion upstream of *SLAV_02776* likely bypasses upstream genes (*SLAV_02825*, *SLAV_02820*, *SLAV_02815*), leading to accumulation of the intermediate strevertene X (Figs. 2, 3). Based on these results, we designated this type I PKS BGC 2 as the strevertene A2 *stv* BGC.

### Biological activities of strevertenes

Strevertenes are known to be active against yeast and fungal strains (Schlingmann et al. 1999; Kim et al. 2011). We determined the MIC values of strevertenes against *S. cerevisiae* as described in the Material and Method section. Commercial strevertene A had an MIC of 20 μg/mL, consistent with previous reports (Schlingmann et al. 1999; Kim et al. 2011). Strevertene A2 exhibited an identical MIC of 20 μg/mL, in agreement with the MIC for the identical compound RKGS-A2215A (Yoshioka et al. 2021). Strevertene X was more potent, with an MIC value of 10 μg/mL, which corresponds to the MIC value for the identical compound AB023a against *Candida albicans* (Cidaria et al. 1993).

### Biosynthetic pathway of strevertenes

**Fig. 6:**
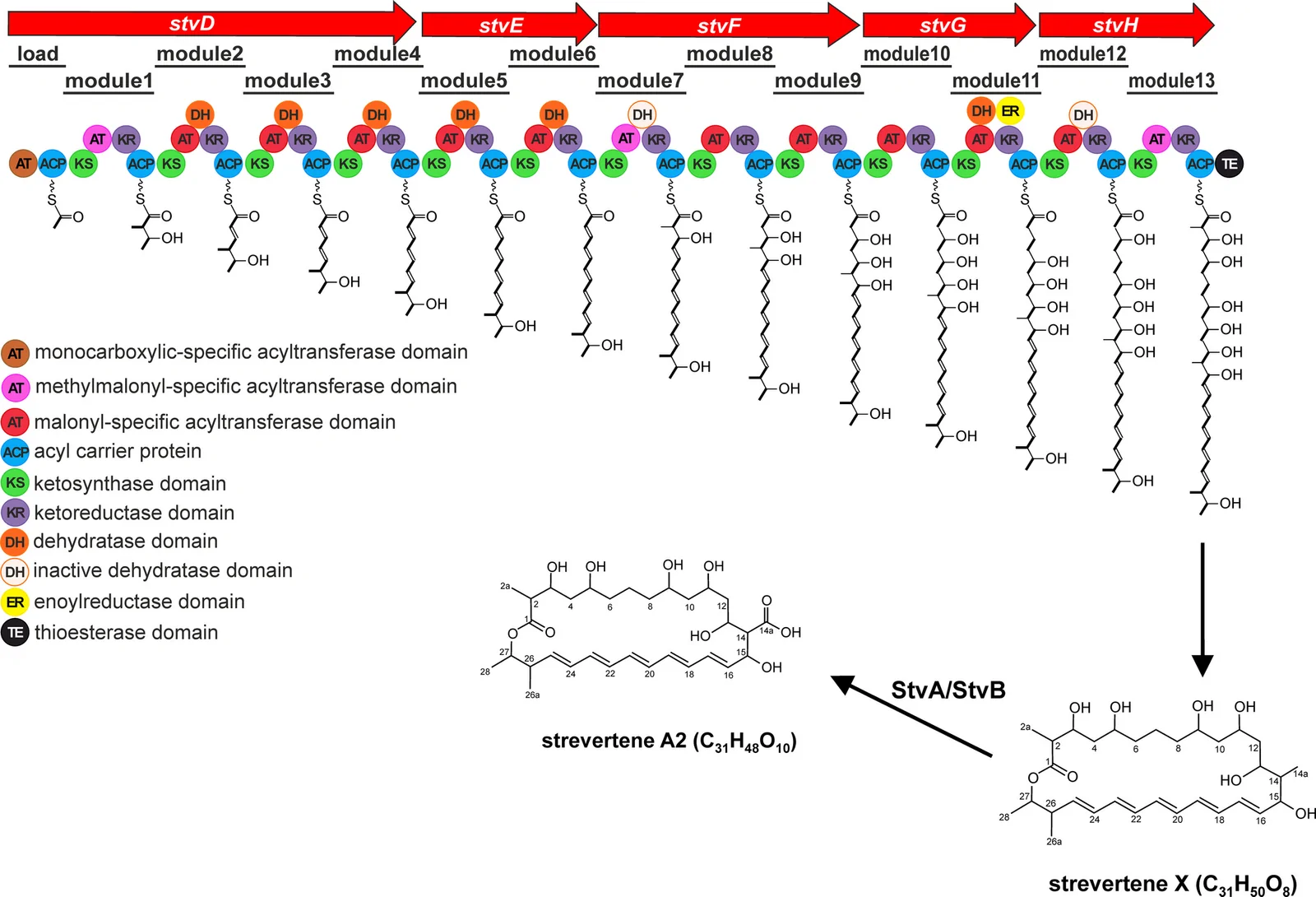
Domain organization of the *stv* type I PKS and a proposed model for strevertene A2 biosynthesis in *S. lavendulae* subsp. *lavendulae* CCM 3239

To propose biosynthetic pathways for strevertene A2 and X produced by our genetically-manipulated strains, we performed in silico bioinformatic analyses of the *stv* BGC. The antiSMASH 7.0 analysis (Blin et al. 2023), together with additional bioinformatic analyses of the cluster, revealed 12 genes encoding products with proposed roles in biosynthesis and regulation (Fig. 2). Five open reading frames (StvD to StvH) showed high sequence similarity to the type I PKS domains, comprising one loading module and 13 extender modules (Fig. 6). The terminal module (module 13) contains the TE domain required for hydrolysis and cyclization of the fully assembled polyketide chain. Each module contains several domains involved in modification of the growing polyketide chain. All 13 extender modules contain an active KS domain harboring the three conserved motifs required for catalytic activity (Fig. S39) (Keatinge-Clay 2012). The loading module lacks the KS domain. All AT domains contained the conserved GHSxGE motif essential for AT activity (Yadav et al. 2003). Analysis of AT substrate specificity (Fig. S40) revealed that AT2, 3, 4, 5, 6, 8, 9, 10, 11, 12 are predicted to select malonyl-CoA, whereas AT1, AT7, and AT13 are predicted to select methylmalonyl-CoA as extender units. The loading AT domain is specific for monocarboxylic substrates (Yadav et al. 2003). These predictions are consistent with the NMR structures of strevertene A2 and X. AntiSMASH 7.0 analysis (Blin et al. 2023) identified 13 KR domains in all extender modules (Fig. 6). All KR domains appear to be functional and are consistent with the NMR structures of strevertene A2 and X. They contain the three conserved amino acid residues (Ser, Tyr, Asn) essential for KR catalytic activity (Reid et al. 2003) (Fig. S41).

KR domains stereospecifically reduce the β-ketone to the corresponding hydroxyl group during chain extension and can be classified as either type A or type B. The absolute configuration of the resulting hydroxyl group can be predicted based on amino acid signatures in six specific regions, yielding six KR subtypes (Keatinge-Clay 2007). Based on the amino acid sequence alignment, KR8, KR9, KR10, KR12, and KR13 were assigned as type A without epimerase activity (subtype A1, lacking the LDD motif in region 1 and containing Trp in region 2). KR1, KR2, KR3, KR4, KR5, KR6, and KR11 were assigned as type B without epimerase activity (subtype B1, containing the LDD motif in region 1) (Keatinge-Clay 2007).

KR7 could not be assigned, as it lacks the characteristic amino acid residues in the defined regions (Fig. S41). Eight DH domains were identified by antiSMASH 7.0 analysis (Blin et al. 2023) in modules 2, 3. 4, 5, 6, 7, 11, and 12 (Fig. 6). With the exception of the DH domains in modules 7 and 12, all DH domains contain all four conserved motifs required for catalytic activity (Keatinge-Clay 2008). The DH12 domain in module 12 is clearly nonfunctional, while the DH7 domain in module 7 lacks several conserved catalytic and structural residues found in functional DH domains (Fig. S42). Only one ER domain was identified, located in module 11, and is responsible for the saturated bond between C7-C8 (Fig. 6). This ER domain contains the conserved motif LxHxxxGGVGxxAxxxA required for ER activity (Kakavas et al. 1997) (Fig. S43). The type I PKS genes are followed by *stvI* (*Slav_02730*) (Fig. 2), which encodes a type II TE. This discrete TE is proposed to function in proofreading during polyketide chain assembly (Kotowska and Pawlik 2014).

Three open reading frames (*stvA*, *stvB*, and *stvC*) are located upstream of the type I PKS biosynthetic genes (Fig. 2). The *stvA* (*Slav_02825*) gene encodes a cytochrome P450 monooxygenase homolog with high similarity to cytochrome P450 monooxygenases from several polyene macrolide BGCs, including NysN from the nystatin BGC (Brautaset et al. 2008), AmphN from the amphotericin BGC (Carmody et al. 2005), FscP from the candicidin BGC (Chen et al. 2009), and PimG and ScnG from the pimaricin BGCs (Aparicio et al. 2016; Qi et al. 2015) (Fig. S44). All of these enzymes have been shown to oxidize the exocyclic methyl group of the cyclized polyketide intermediate to a carboxyl group at the position corresponding to C14 in strevertene A (Brautaset et al. 2008; Carmody et al. 2005; Chen et al. 2009; Aparicio et al. 2016; Qi et al. 2015). The *stvB* (*Slav_02820*) gene, located immediately downstream of *stvA* (Fig. 2), encodes a small ferredoxin-like protein. Genes encoding close homologs (NysM, AmphM, FscFE, PimF, and ScnF) are consistently located downstream of cytochrome P450 monooxygenase genes in previously characterized polyene macrolide BGCs (Fig. S45). AmphM has been proposed to function as a ferredoxin partner that cooperates with the AmphN cytochrome P450 monooxygenase (Carmody et al. 2005). Another gene, *stvC* (*Slav_02815*), located upstream of the type I PKS biosynthetic genes (Fig. 2), encodes a cholesterol oxidase homolog with high similarity to PimE and ScnE from pimaricin BGCs (Fig. S46). PimE is essential for both the production and export of pimaricin and is believed to function as a sensor detecting ergosterol in the fungal membrane (Mendes et al. 2007).

Three additional genes (*stvRI*, *stvRII*, and *stvRIII*) are located immediately upstream of the PKS biosynthetic genes (Fig. 2). These genes encode large proteins (> 900 amino acids) that belong to the Large ATP-binding LuxR-family (LAL) of transcriptional regulators (De Schrijver and De Mot 1999). Each regulator contains an N-terminal nucleotide-binding domain with Walker A and B motifs (Walker et al. 1982) and a C-terminal LuxR-type helix–turn–helix (HTH) DNA-binding domain. These regulators show strong similarity to NysRI, NysRII, and NysRIII from the nystatin BGC (Sekurova et al. 2004), AmphRI, AmphRII, and AmphRIII from the amphotericin BGC (Carmody et al. 2004), and FscRI, FscRII, and FscRIII from the FR-008/candicidin BGC (Zhu et al. 2020) (Fig. S47). In all three BGCs, these regulatory genes are essential for antibiotic biosynthesis. Moreover, the organization of these regulatory genes is collinear in the polyene BGCs and in the *stv* BGC responsible for strevertene A2 biosynthesis. Based on this bioinformatic analysis, a biosynthetic pathway was proposed for both strevertenes X and A2 (Fig. 6).

The type I modular PKS encoded by *stvD–stvH* catalyzes polyketide biosynthesis through 13 rounds of decarboxylative condensation, using acetyl-CoA as the starter unit (loading module), three methylmalonyl-CoA extender units (modules 1, 7, and 13), and ten malonyl-CoA extender units (modules 2–6 and 8–12). The linear polyketide intermediate is released and cyclized by the thioesterase domain of StvH, yielding strevertene X. The fidelity of polyketide chain assembly is likely ensured by the type II TE StvI. As described above, the predicted structure of strevertene X is in good agreement with the NMR data. Following formation of strevertene X, the cytochrome P450 enzyme StvA, likely in cooperation with the ferredoxin StvB, catalyzes oxidation of the exocyclic methyl group at C14 to a carboxyl group, thereby yielding strevertene A2. This proposed biosynthetic pathway is consistent with the observed production of strevertenes X and A2 in genetically manipulated strains carrying the *kasOp*^⁎^ promoter inserted upstream of either the *stvD* (*SLAV_02776*) biosynthetic gene or the *stvRI* (*SLAV_02840*) regulatory gene (Figs. 2 and 3).

## Discussion

Genome sequencing of Gram-positive bacteria of the genus *Streptomyces* has revealed their remarkable potential for producing novel natural products that can be developed into therapeutic compounds. However, the vast majority of BGCs identified in *Streptomyces* genomes are cryptic or transcriptionally silent (Belknap et al. 2020). As summarized in the Introduction, several genetic strategies have been developed to overcome this limitation. Approaches to activate silent BGCs primarily rely on genetic manipulation to control the expression of biosynthetic genes or cluster-situated positive regulatory genes, most commonly through the introduction of strong promoters (Liu et al. 2021; Zhu et al. 2013). Various native promoter refactoring strategies, including CRISPR-based systems, have been applied to activate silent BGCs; however, their implementation in non-model *Streptomyces* strains remains challenging and subject to technical limitations (Zhang et al. 2017; Zhou et al. 2024).

Genome mining of the auricin-producing strain *Streptomyces lavendulae* subsp. *lavendulae* CCM 3239 enabled the identification of 36 BGCs involved in secondary metabolite biosynthesis (Table S1), including the previously characterized auricin BGC (Busche et al. 2018; Kormanec et al. 2014; Matulova et al. 2019) and a silent BGC for indigoidine (Novakova et al. 2010). In this study, we developed a simple strategy to activate BGCs in this strain by replacing native promoters with the strong *kasOp*^⁎^ promoter (Wang et al. 2013) via homologous recombination. Two approaches were employed. In the first, the native promoter of the known silent biosynthetic gene *bpsA* from the indigoidine BGC (Novakova et al. 2010) was replaced with *kasOp*^⁎^ using the PCR-targeted REDIRECT strategy (Gust et al. 2003) and the cosmid pCosSA19 containing the *bpsA* region. The resulting validated *S. lavendulae kasOp::bpsA* mutant exhibited dramatically increased indigoidine production compared to the WT strain (Fig. 1b), confirming the effectiveness of this approach. However, this strategy has limitations, as it requires the availability of a cosmid library for the target strain and may exert polar effects on transcription of adjacent genomic regions. In the second approach, native promoter regions upstream of the proposed biosynthetic gene *SLAV_02776* (*stvD*) and the positive regulatory gene *SLAV_02840* (*stvRI*) in the cryptic type I PKS BGC 2 (Table S1; Fig. 3) were replaced with the *kasOp*^⁎^ promoter using the plasmid vector pKasOpAmRT2, which contains a bidirectional *T5* terminator to prevent polar transcriptional effects. Successful induction of novel natural products confirmed that this BGC was transcriptionally silent under standard conditions. Interestingly, insertion of the *kasOp*^⁎^ promoter at different positions within this type I PKS BGC resulted in activation of distinct polyene compounds. Insertion upstream of *stvRI* (*SLAV_02840*) at the beginning of the BGC, where all genes are oriented in the same direction (Figs. 2, 3), led to production of a polyene compound closely related to strevertene A (9), but with altered hydroxyl group positioning. This compound was designated strevertene A2 (Fig. 4). In contrast, insertion upstream of *stvD* (*SLAV_02776*) resulted in production of a distinct polyene lacking the carboxyl group at C14, which is replaced by a methyl group (Fig. 4); this compound was named strevertene X.

Strevertenes represent a subgroup of polyene macrolides that lack the characteristic hemiketal–tetrahydropyran and aminoglycoside moieties but retain a carboxylic acid group (Schlingmann et al. 1999). Their biosynthesis is therefore expected to resemble that of classical polyene macrolides such as candicidin (FR-008), amphotericin, pimaricin, and nystatin, which are synthesized by modular type I PKSs (Caffrey et al. 2016). Combining in silico analysis of biosynthetic enzymes and PKS domain organization enabled us to propose a biosynthetic model for strevertene A2 (Fig. 6) that is consistent with the production of polyenes observed in both engineered strains. The modular type I PKS encoded by *stvD–stvH* catalyzes the formation of a linear polyketide intermediate, which is released and cyclized by the TE domain of StvH to yield strevertene X. Subsequently, the cytochrome P450 enzyme StvA, likely in cooperation with the ferredoxin StvB, oxidizes the exocyclic methyl group at C14 of strevertene X to a carboxyl group, producing strevertene A2. This terminal oxidative tailoring step mirrors those observed in the biosynthesis of candicidin, amphotericin, pimaricin, and nystatin, mediated by homologous cytochrome P450 monooxygenase/ferredoxin pairs FscP/FscFE, AmphN/AmphM, PimG/PimF, and NysN/NysM, respectively (Brautaset et al. 2008; Carmody et al. 2005; Chen et al. 2009; Aparicio et al. 2016; Qi et al. 2015). Notably, although strevertene X is a biosynthetic intermediate, it exhibited approximately twofold higher antifungal activity than the final product strevertene A2. Similar enhancements in biological activity have been reported for other polyene derivatives in which the exocyclic carboxyl group is replaced by a methyl group, including pimaricin (Qi et al. 2015), amphotericin (Carmody et al. 2005), candicidin (FR-008) (Chen et al. 2009), nystatin (Brautaset et al. 2008), and tetramycin B (Sheng et al. 2020).

The *stv* BGC identified in *S. lavendulae* subsp. *lavendulae* CCM 3239 likely represents the first characterized BGC responsible for strevertene-like polyene biosynthesis. Database mining using both nucleotide sequences and predicted protein products identified several homologous BGCs with conserved gene organization and 74–91% nucleotide identity, predominantly in *Streptomyces* genomes lacking associated metabolite characterization (Fig. S48). During preparation of this manuscript, a homologous *lad* BGC from *S. lavendulae* FRI-5 was reported and shown to direct biosynthesis of lavencidin (which differs from strevertene A2 by an ethyl group at C2) and RKGS-A2215A (identical to strevertene A2) (Otsuka et al. 2025). Aside from the *stv* BGC, this *lad* cluster represents the only other characterized member of this BGC family. Although nucleotide identity between the *stv* and *lad* BGCs is relatively modest (79%), all homologous genes are collinear, and domain analysis revealed conserved KS, AT, KR, DH, ER, and ACP domains across all five type I PKS ORFs, indicating a conserved biosynthetic logic. The production of both lavencidin and RKGS-A2215A by the *lad* BGC suggests flexibility at position C2, which may also apply to the *stv* BGC. Indeed, HR-MS analysis of a minor compound detected in extracts of the *S. lavendulae kasOp*::2776 strain revealed a + 14 Da mass shift, consistent with substitution of a methyl group by an ethyl group (data not shown). This variability is likely due to relaxed substrate specificity of the monocarboxylic acid-selective AT domain in the loading module, which may accept propionyl-CoA in addition to acetyl-CoA. Similar substrate flexibility has been observed during biosynthesis of AB023a/AB023b (Bortolo et al. 1993) and strevertenes A and B (Schlingmann et al. 1999). Notably, loading modules of type A PKSs containing an AT–ACP bidomain, as present in erythromycin and avermectin PKSs, are known to exhibit relaxed substrate specificity (Long et al. 2002).

In conclusion, we describe a simple and efficient strategy for activation of silent BGCs in *S. lavendulae* subsp. *lavendulae* CCM 3239 by replacing native promoters with the strong *kasOp*^⁎^ promoter via homologous recombination. Using this approach, we activated a silent type I PKS BGC and demonstrated that promoter insertion at different genomic positions can yield distinct polyene products. These included the final product strevertene A2, closely related to strevertene A, and the biosynthetic intermediate strevertene X, which displayed significantly enhanced antifungal activity. in silico analysis of the biosynthetic machinery supported a biosynthetic model consistent with the observed production of strevertenes A2 and X.

## Supplementary Information

Below is the link to the electronic supplementary material.

ESM 1 (PDF 6.81 MB)

## Data Availability

The authors confirm that all relevant data are included in the article and/or its supplementary information file. Raw NMR data are available for download from the BMRBog database (Hoch et al. 2023) at https://bmrbig.org under the entry number bmrbig140.

## Abbreviations

*aa*: Amino acid(s)
*CAN*: Acetonitrile
*ACP*: Acyl carrier protein
*Amp*: Ampicillin
*Apr*: Apramycin
*AprR*: Apr resistance
*AT*: Acyltransferase
*BGC*: Biosynthetic gene cluster
*CCM*: Czech Collection of Microorganisms
*Clm*: Chloramphenicol
*COSY*: Correlation Spectroscopy
*CSLS*: Corn steep liquor-sucrose
*DH*: Dehydratase
*DIG*: Digoxigenin
*DMSO*: Dimethylsulfoxide
*ER*: Enoylreductase
*HPLC*: High-performance liquid chromatography
*HR-ESI-MS*: High-resolution electrospray ionization mass spectrometry
*HMBC*: Heteronuclear Multiple Bond Correlation
*HSQC*: Heteronuclear Single-Quantum Coherence
*HTH*: Helix–turn–helix
*Kan*: Kanamycin
*kb*: Kilobase(s)
*KR*: Ketoreductase
*KS*: Ketosynthase
*LB*: Luria-Bertani
*MIC*: Minimum inhibitory concentration
*NA*: Nalidixic acid
*NMR*: Nuclear magnetic resonance
*NOESY*: Nuclear Overhauser Effect Spectroscopy
*NRPS*: Non-ribosomal peptide synthase
*PKS*: Polyketide synthase
*RBS*: Ribosome binding site
*RT-PCR*: Reverse transcription-PCR
*TE*: Thioesterase
*TLC*: Thin-layer chromatography
*TMS*: Tetramethylsilane
*TOCSY*: Total Correlation Spectroscopy
*WT*: Wild-type
