# Supplementary material for: Activation of a gene cluster in Streptomyces lavendulae subsp. lavendulae CCM 3239 induced strevertene production
Source: Appl Microbiol Biotechnol. 2026 Jun 15;110:255. doi: 10.1007/s00253-026-13908-9 (PMC13500442; doi:10.1007/s00253-026-13908-9)
Supplement: Supplementary file 1 [file 253_2026_13908_MOESM1_ESM.pdf]

Applied Microbiology and Biotechnology

Supporting information for:

Activation of a gene cluster in *Streptomyces lavendulae* subsp. *lavendulae* CCM 3239 induced strevertene production

Filip Opaterny[1], Rachel Javorova[1], Michal Soral[2], Lubomira Feckova[1], Dominika Csolleiova[1], Renata Novakova[1], Kamila Cholvadtova[1], Iveta Uhliarikova[2], Maria Kopacova[2], Vladimir Patoprsty[2], Bronislava Rezuchova[1], Beatrica Sevcikova[1], Jan Kormanec[1*]

[1] Institute of Molecular Biology, Slovak Academy of Sciences, 845 51 Bratislava, Slovak Republic.
[2] Institute of Chemistry, Slovak Academy of Sciences, 845 38 Bratislava, Slovak Republic.

[*] *Corresponding author:* Jan Kormanec, Institute of Molecular Biology, v.v.i., Slovak Academy of Sciences, Dubravska cesta 21, 845 51 Bratislava, Slovak Republic.
Tel: +421 2 59307419, Fax: +421 2 59307416, E-mail: jan.kormanec@savba.sk
ORCID ID: 0000-0003-0377-6413

Running title: Activation of antifungal strevertenes

Contents

Table S1. Biosynthetic gene clusters (BGCs) in the genome of *S. lavenduale* subsp. *lavendulae* CCM 3239.
Table S2. Bacterial strains and plasmids used in this study.
Table S3. Oligonucleotides used in this study.
Figure S1. A Genetic organization of the *bpsA* gene in wild-type and mutant strains and Souther blot hybridization analysis.
Figure S2. A Genetic organization of the *Slav_02776* and *Slav_02840* genes in wild-type and mutant strains and Souther blot hybridization analysis.
Figure S3. Scheme of the plasmid pKasOpAmRb.
Figure S4. Scheme of the plasmid pKasOpAmRT2 and insertion of the *kasOp** promoter upstream of the *SLAV_02776* and *SLAV_02840* genes.
Figure S5. Verification of the correct integration in *S. lavendulae, kasOp::2776* and *S. lavendulae, kasOp::2840* strains by nucleotide sequencing.
Figure S6. Transcriptional analysis of four selected genes from BGC2 (*Slav-02840, Slav_02825, Slav_02815, Slav_02776*) by RT-PCR
Figure S7. UV/VIS spectra of strevertenes.
Figure S8. HR-ESI-MS spectra of strevertenes.
Table S4. NMR data for strevertene A2 in DMSO-d_6
Figures S9-S24. NMR data for strevertene A2 in DMSO-d_6
Table S5. NMR data for strevertene X in CD_3OD.

Figures S25-S38. NMR data for strevertene X in CD_3OD
Figure S39. Amino acid alignment of *stv* BGC KS domains.
Figure S40. Amino acid alignment of *stv* BGC AT domains.
Figure S41. Amino acid alignment of *stv* BGC KR domains.
Figure S42. Amino acid alignment of *stv* BGC DH domains.
Figure S43. Comparison of the *stv* BGC domain of ER11 with the EryER4 domain from the erythromycin PKS.
Figure S44. Comparison of StvA from the *stv* BGC with cytochrome P450 monooxygenases.
Figure S45. Comparison of StvB from the *stv* BGC with ferredoxin-like proteins.
Figure S46. Comparison of StvC from the *stv* BGC with cholesterol oxidases PimE and ScnE.
Figure S47. Comparison of putative regulators StvRI, StvRII, StvRIII with the LAL family of transcriptional regulators.
Fig. S48. Physical maps of *S. lavendulae* subsp. *lavendulae* CCM 3239 *stv* BGC and other similar BGCs.
Fig. S49. Nucleotide sequence of the *stv* BGC

Table S1. Biosynthetic gene clusters (BGCs) identified in the genome of *S. lavenduale* subsp. *lavendulae* CCM 3239 (GenBank Acc. No. CP024985 for chromosome and KJ396772 for linear plasmid pSA3239) by antiSMASH version 7.0 (Blin et al. 2023).

S. lavendulae subsp. lavendulae 3239 chromosome, 8,691,711 bp						
**Region**	**Type**	**From**	**To**	**Most similar known cluster**		**Similarity**
**BGC 1**	NRPS, NRPS-like	103,999	160,190	WS9326	NRP	12%
**BGC 2**	lanthipeptide-class-iv, type I PKS	549,149	642,970	kendomycin B	Polyketide	18%
**BGC 3**	RRE-containing cluster, LAP, thiopeptide, NRPS, type III PKS	816,702	936,667	alkylresorcinol	Polyketide	100%
**BGC 4**	melanin	990,722	1,001,114	saframycin A / saframycin B	NRP	4%
**BGC 5**	melanin	1,011,727	1,022,104	melanim	Other	100%
**BGC 6**	terpene	1,026,377	1,068,296	ebelactone	Polyketide	5%
**BGC 7**	aminopolycarboxylic-acid	1,199,169	1,212,574	EDHA	Other	55%
**BGC 8**	terpene	1,620,603	1,647,549	hopene	terpene	61%
**BGC 9**	type I PKS	1,800,397	1,992,154	linearmycin A, B, C	Polyketide modular type I polyketide	100%
**BGC 10**	NAPAA	2,087,411	2,121,382	ε-poly-L-lysine	NRP	100%
**BGC 11**	NRPS	2,144,126	2,206,900	omnipeptin	NRP:Cyclic depsipetide	11%
**BGC 12**	terpene	2,209,815	2,232,052	toxoflavin / fervenulin	Other	14%
**BGC 13**	RiPP-like	2,309,818	2,232,218			
**BGC 14**	NRPS, hydrogen-cyanide	2,347,229	2,404,356	cahuitamycin A, B, C	NRP	8%
**BGC 15**	NRPS,ladderane	2,435,270	2,510,167	colibrimycin	NRP + other	11%
**BGC 16**	NI-siderophore	2,582,344	2,616,190	kinamycin	polyketide	19%
**BGC 17**	CDPS	2,945,770	2,966,567	BD-12	NRP	7%
**BGC 18**	lanthipeptide-class-v	4,503,799	4,545,955	pristinin A3	RiPP	11%
**BGC 19**	lanthipeptide-class-v, butyrolactone	4,672,692	4,714,684	neocarzinostatin	Polyketide	8%
**BGC 20**	NI-siderophore	5,351,832	5,381,613	legonoxamine A, desferrioxamin B	Other	100%
**BGC 21**	NRPS-like	6,479,658	6,523,545	α-naphtocyclinoic acid/fogamycin	Polyketide	7%
**BGC 22**	thiopeptide, LAP	7,015,654	7,044,963	lactazole	RiPP: Thiopeptide	55%
**BGC 23**	terpene	7,261,093	7,282,142			
**BGC 24**	melanin	7,315,757	7,326,179	melanin	Other	100%
**BGC 25**	Type II PKS	7,470,274	7,542,834	spore pigment	Polyketide	66%
**BGC 26**	pyrrolidine	7,740,612	7,772,194	anisomycin	Other	84%
**BGC 27**	melanin	7,855,250	7,865,675	saframycin A, B	NRP	4%
**BGC 28**	NRPS-like, RiPP-like	8,254,308	8,307,732	streptothricin	NRP	87%
**BGC 29**	NRPS-like, NRPS	8,531,522	8,587,713	WS9326	NRP	12%
S. lavendulae subsp. lavendulae 3239 linear plasmid pSA3239, 241,076 bp						
**Region**	**Type**	**From**	**To**	**Most similar known cluster**		**Similarity**
**BGC 1**	Type II PKS	1	30,974	xantholipin	polyketide	16%
**BGC 2**	Type II PKS, butyrolactone	51,003	89,936	auricin	polyketide/saccharide	100%
**BGC 3**	Type II PKS	73,052	116,903	auricin deoxysugar moieties	saccharide	100%
**BGC 4**	NRPS	86,904	96,903	indigoidine	NRP	100%
**BGC 5**	NRPS	127,638	141,373		NRP	13%
**BGC 6**	Other	141,360	148,702	valanymycin	Azoxy compound	70%
**BGC 7**	Type I PKS	159,759	203,694	medermycin	polyketide	11%

Abbrevations: PKS-Polyketide synthase; NRPS-Non-ribosomal peptide synthetase; NAPAA-non-α-poly-amino acids like ε-Polylysin; RiPP-ribosomally synthesised and post-translationally modified peptide product; RRE-RiPP recognition element; CDPS-tRNA-dependent cyclodipeptide synthase; LAP-Linear azol(in)e-containing peptide

Table S2. Bacterial strains and plasmids used in this study.

Strain or plasmid	Genotypes and relevant characteristics[a]	Reference or source
Strains		
S. lavendulae subsp. *lavendulae* CCM 3239	Wild-type	(Busche et al. 2018)
S. lavendulae kasOp::bpsA	Apr^R, the 300-bp region upstream of *bpsA* replaced by the AprR-*kasOp** cassette *in S. lavendulae* subsp. *lavendulae* CCM 3239	this study
S. lavendulae kasOp::2776	Apr^R, the 35-bp region upstream of *Slav_02776* replaced by the AprR-*kasOp** cassette in*S. lavendulae* subsp. *lavendulae* CCM 3239	this study
S. lavendulae kasOp::2840	Apr^R, the 131-bp region upstream of *Slav_02840* replaced by the AprR-*kasOp** in *S. lavendulae* subsp. *lavendulae* CCM 3239	this study
E. coli DH5α	F$^-$ *supE44* Δ*lacU169* (ϕ80d*lacZ*ΔM15) *hsdR17 recA1 endA1 gyrA96 thi-1 relA1*, host strain for plasmid cloning and propagation	ThermoFisher Scientific
E. coli ET12567/pUZ8002	non-methylating strain used for conjugation from *E. coli* to *Streptomyces*, *recF dam dcm hsdC*, Clm^R Str^R Tet^R Kan^R	(Kieser et al. 2000)
E. coli BW25113	Δ(*araD-rhaB*) Δ*lacZ4787* (*::rrnB-4*) *lacIp-40000(*lacI^Q*)* λ$^-$ *rpoS369*(Am) *rph-1* Δ(*rhaD-rhaB*)568 *hsdR514*, host strain for λ RED recombination	(Gust et al. 2003)
Plasmids		
pCosSA19	Kan^R, *ori* colE1, the 41-kb *Sau*3AI DNA fragment from pSA3239 in sCos-1	(Novakova et al. 2010)
pCosSA19-bpsA::kasOp	Kan^R, Apr^R, *ori* colE1, the 0.3-kb region upstream of bpsAS replaced by the AprR-*kasOp** cassette in pCosSA19	this study
LITMUS 28	Amp^R, *ori* colE1, *E. coli* plasmid cloning vector	New England Biolabs
pIJ773	Amp^R, Apr^R, *ori* colE1, *E. coli* plasmid vector	(Gust et al. 2003)
pAMT1	Amp^R, Apr^R, *ori* colE1, the *aac3(IV)* gene in LITMUS 28	this study
pKasOpAmRb	Amp^R, Apr^R, *ori* colE1, the *kasOp** promoter in pAMT1	this study
pBSBR5	Amp^R, *ori* colE1, *E. coli* plasmid vector	(Javorova et al. 2024)
pKasOpAmRT1	Amp^R, Apr^R, *ori* colE1, the *T5* terminator in pKasOpAmRb	this study
pGUS	Apr^R, Spc^R, *ori* colE1, *E. coli gusA* reporter plasmid vector	(Myronovskyi et al. 2011)
pAPH3	Amp^R, Kan^R, *ori* colE1, *E. coli* plasmid vector	(Csolleiova et al. 2021)
pSB40N	Amp^R, *ori* colE1, *E. coli* plasmid vector	(Kormanec and Sevcikova 2000)
pAPH4	Amp^R, Kan^R, *ori* colE1, the *gusA* gene in pAPH3	this study
pAPH4B	Amp^R, Kan^R, *ori* colE1, the *gusA* gene in pSB40N	this study
pAMR13B	Amp^R, Kan^R, Apr^R, *ori* colE1, the *aac3(IV)* gene in pAPH4B	this study
pKasOpAmRT2	Amp^R, Kan^R, Apr^R, *ori* colE1, the the *kasOp** promoter in pAMR13	this study

pKasOp-2776	Amp^R, Kan^R, Apr^R, *ori* colE1, 5-kb regions upstream and downstream of the *Slav_02776* gene inserted in pKasOpAmRT2	this study
pKasOp-2840	Amp^R, Kan^R, Apr^R, *ori* colE1, 5-kb regions upstream and downstream of the *Slav_02840* gene inserted in pKasOpAmRT2	this study

[a] Clm^R, chloramphenicol resistance; Amp^R, ampicillin resistance; Apr^R, apramycin resistance; Kan^R, kanamycin resistance; Str^R, streptomycin resistance; Spc^R, spectionomycin resistance; Tet^R, tetracycline resistance

Table S3. Oligonucleotides used in this study. Cloning sites are underlined.

Oligonucleotide	Sequence (5′ → 3′)
KasOpDir	GATCCATTCGAACGGTCTCTGCTTTGACAACATGCTGTGCGGTGTTGTAAAGTCGTGGCCAGGAGATGACTGACTGAG
KasOpRev	GTACCTCAGTCAGTCATCTCCTGGCCACGACTTTACAACACCGCACAGCATGTTGTCAAAGCAGAGACCGTTCGAATG
AmR1	CCCCACTAGTCCCAATTGCCTGTACACCGCTAGCCCCATATGCCATCGATTCCGGGGATCCGTCGACC
AmR2	CCCCAAGCTTCCGGTACCCCGAATTCCCATTAATCCCTTAAGCCCCTAGGCCACATGTAGGCTGGAGCTGCTTC
BpsAkasDir	CGGATCAGTGACCCGGCTCCTCCAGAGCCGGTGAACGCCCTGTAGGCTGGAGCTGCTTC
BpsAkasRev	CGCTGGTCTCCTGAAGAGTCATTTCCCGCTTCCTTACGTTTACCTCAGTCAGTCATCTCC
2776BsrG	CCCCTGTACAGCGCGAATCGTCACGGGGTTTCC
2776Afl	CCCCCTTAAGTTGACCATGCTGTCGAGGGAGG
2776Spe	CCCCACTAGTAGGACTGGGAGAGCGACGTACTGC
2776Mfe	CCCCCAATTGGGTTTACGACGCCTTGATGTCC
2840Eco	CCCCCGAATTCCACTCCAGCAGGTCGGCGAAGG
2840Afl2	CCCCTTAAGGTCGCCTACGATCTTGTTTGTGC
2840Spe	CCCCACTAGTGATCAAGGCGCTGACCACGCTCG
2840Mfe	CCCCCAATTGATTCCCGGACCCCCGAAGAAACG
neoRseq1	GGTCAGCACCGTTTCTGCGGACTGG
AmRseq1	GGAATCCTGCTCTGCGAGGCTGGC
mut80	GGGTTCCGCGCACATTTCCCCG
T5r	GCCGAGGTAGCTCTGGATCTGG
2776seqR	CGTATCGGGGAGGCGGATGTCC
2815seqD	GGGCATCGACGACTGGGACAACC
2840seqR	GCTGGGCCAGGACGATCAGCAGG
2845seqD	CGCGTCGGAGACGTGCAGCAGC

2840dir	ACACCGCCGCACTGGACGACC
2840rev	GGGACGCCGGTGCGCTGGGC
2825dir	GCCCGGATGGAATTCGCCGACG
2825rev	CGACCTCGGTGGACTCCCCG
2815dir	GTCGTCATCGGGACGGGCTACG
2815rev	CTTGTACCAGTCGGTGCCCTCG
2776dir	CGAAGCCGACCCGGCGTTCG
2776rev	GTACCCCCCGACGCAACCACC
hrdBdir	GCAAGGCCGAGGGGCAGATCG
hrdBrev	CTCCTCGGCCTCGGCCTTCG

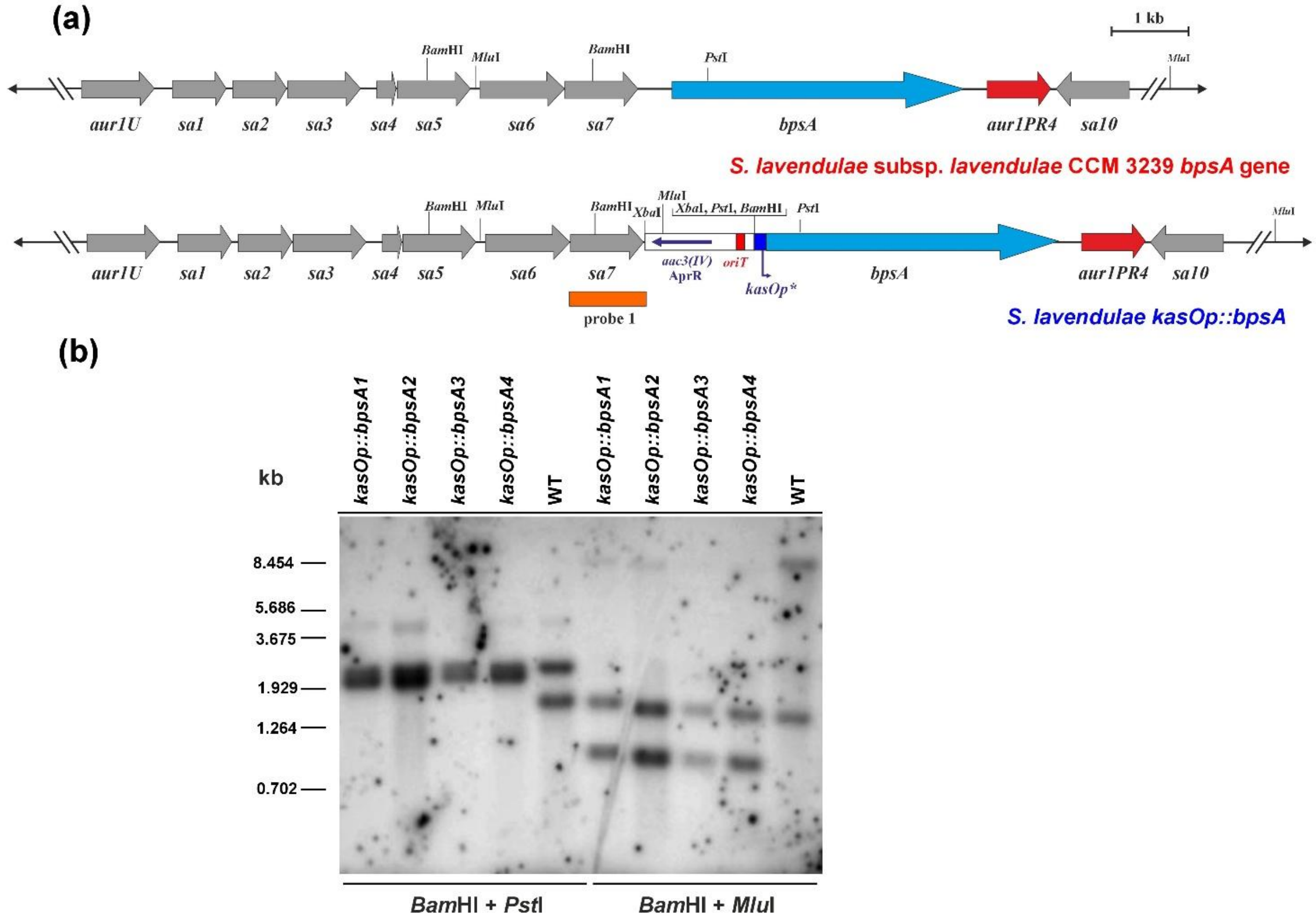

Fig. S1. a Genetic organization of the region surrounding the *bpsA* gene in wild-type *S. lavendulae* subsp. *lavendulae* CCM 3239 and the recombinant strain *S. lavendulae kasOp::bpsA.* Thick arrows indicate the direction and size of genes; the blue arrow represents the *bpsA* gene and the red arrow the regulatory gene. Gene labelling is based on the sequence with GenBank accession No. KJ396772. The box with the blue arrow indicates the AprR-*kasOp** cassette containing the AprR *aac3(IV)* gene and the *oriT* origin of transfer (red column) from plasmid pIJ773 (Gust et al. 2003). The orange bar below the maps indicates the position of probe 1 used for Southern hybridization analysis. Relevant restriction sites are shown. **b** Southern blot hybridization analysis of four *S. lavendulae kasOp::bpsA* clones and wild-type *S. lavendulae* subsp. *lavendulae* CCM 3239 (WT) as a control. One μg of genomic DNA from each strain was digested with the indicated restriction endonucleases and separated by electrophoresis in a 0.8% (w/v) agarose gel. After transfer to a Hybond N membrane, hybridization was performed according to the standard DIG protocol, as described in Experimental procedures, using DIG-labelled probe 1 covering the *sa7* gene. *Bst*EII-digested lambda DNA was used as a size standard.

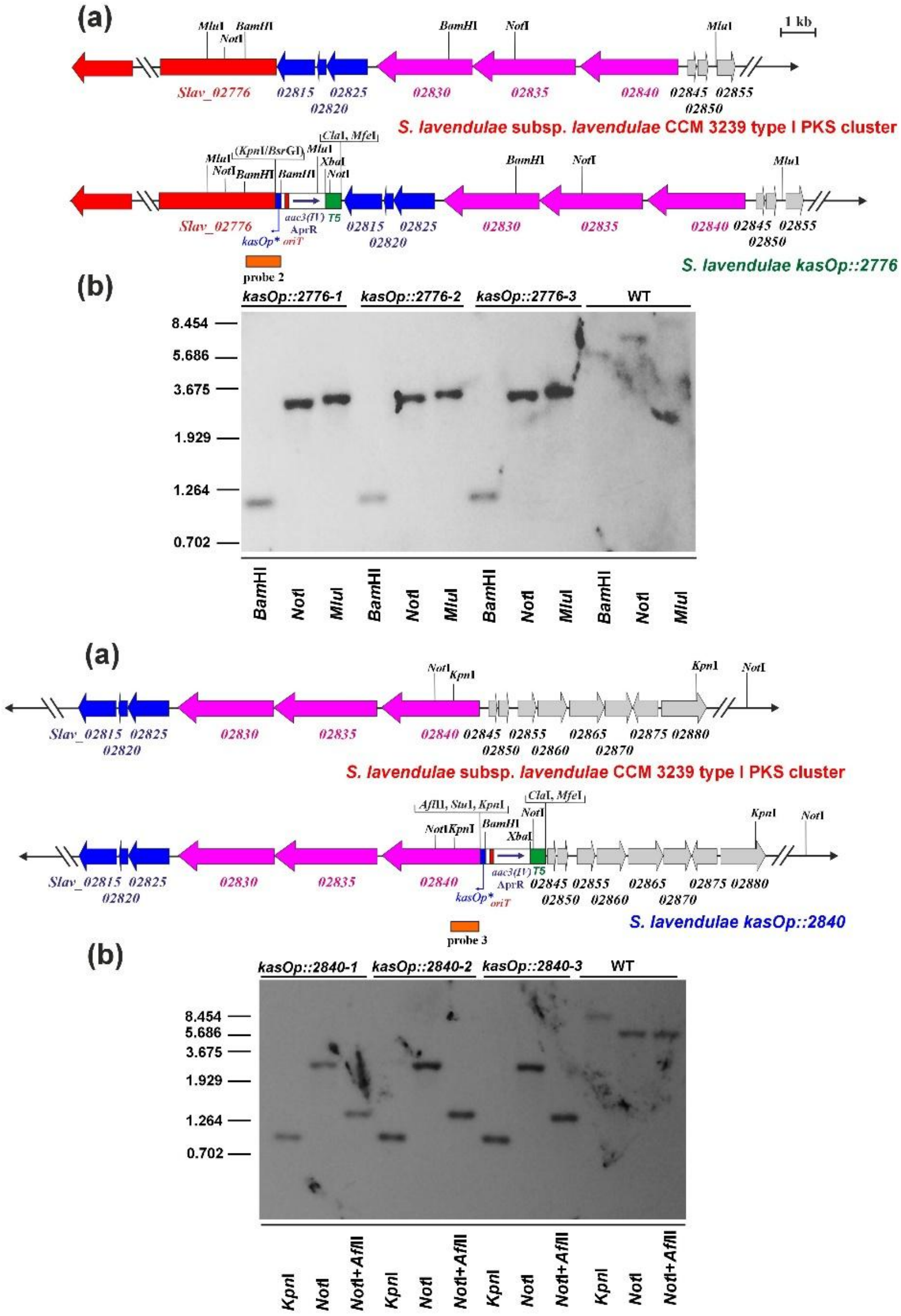

Fig. S2. a Genetic organization of the region surrounding the inserted *T5*-AprR-*kasOp** cassette within the *stv* BGC of wild-type *S. lavendulae* subsp. *lavendulae* CCM 3239 and the recombinant strains *S. lavendulae kasOp::2776* and *S. lavendulae kasOp::2840.* Thick arrows indicate the direction and size of genes; the red arrows represent type I PKS biosynthetic genes, blue arrows tailoring biosynthetic genes, and the pink arrows regulatory genes. The blue box with a bent arrow indicates the position of the *kasOp** promoter. Gene labelling is based on the *S. lavenduale* subsp. *lavendulae* CCM 3239 genomic sequence (GenBank accession No. CP024985). The box with the blue arrow indicates the AprR-*kasOp** cassette containing the *aac3(IV)* AprR gene and the *oriT* origin of transfer (red column) from plasmid pIJ773 (Gust et al. 2003). The orange bar below the maps indicates the position of probe 1 used for Southern hybridization analysis. Relevant restriction sites are shown. **b** Southern blot hybridization analysis of three clones each from *S. lavendulae kasOp::2776* and *S. lavendulae kasOp::2840,* and wild-type *S. lavendulae* subsp. *lavendulae* CCM 3239 (WT) used as a control. One μg of genomic DNA from each strain was digested with the indicated restriction endonucleases and separated by electrophoresis in a 0.8% (w/v) agarose gel. After transfer to a Hybond N membrane, hybridization was performed according to the standard DIG protocol as described in Experimental procedures, using DIG-labelled probe 2 or probe 3 covering the *Slav_02776* and *Slav_02840* genes, respectively. *Bst*EII-digested lambda DNA was used as a size standard.

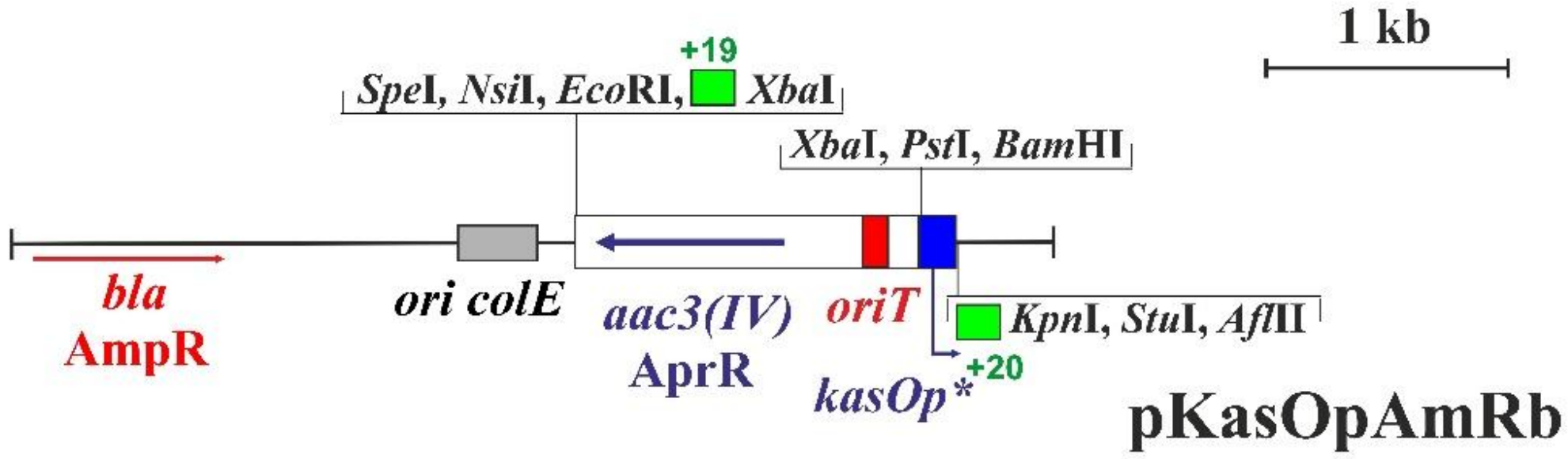

Fig. S3 Constructed plasmid pKasOpAmRb containing a cassette with the strong *kasOp** promoter (Wang et al. 2013) and the *aac3(IV)* AprR gene with the *oriT* origin of transfer from plasmid pIJ773 (Gust et al. 2003), cloned into the backbone of the *E. coli* plasmid pBluescript II KS+ carrying the ColE1 replication origin and the *bla* AmpR gene. The positions of the +19 and +20 priming sequences are indicated by green boxes.

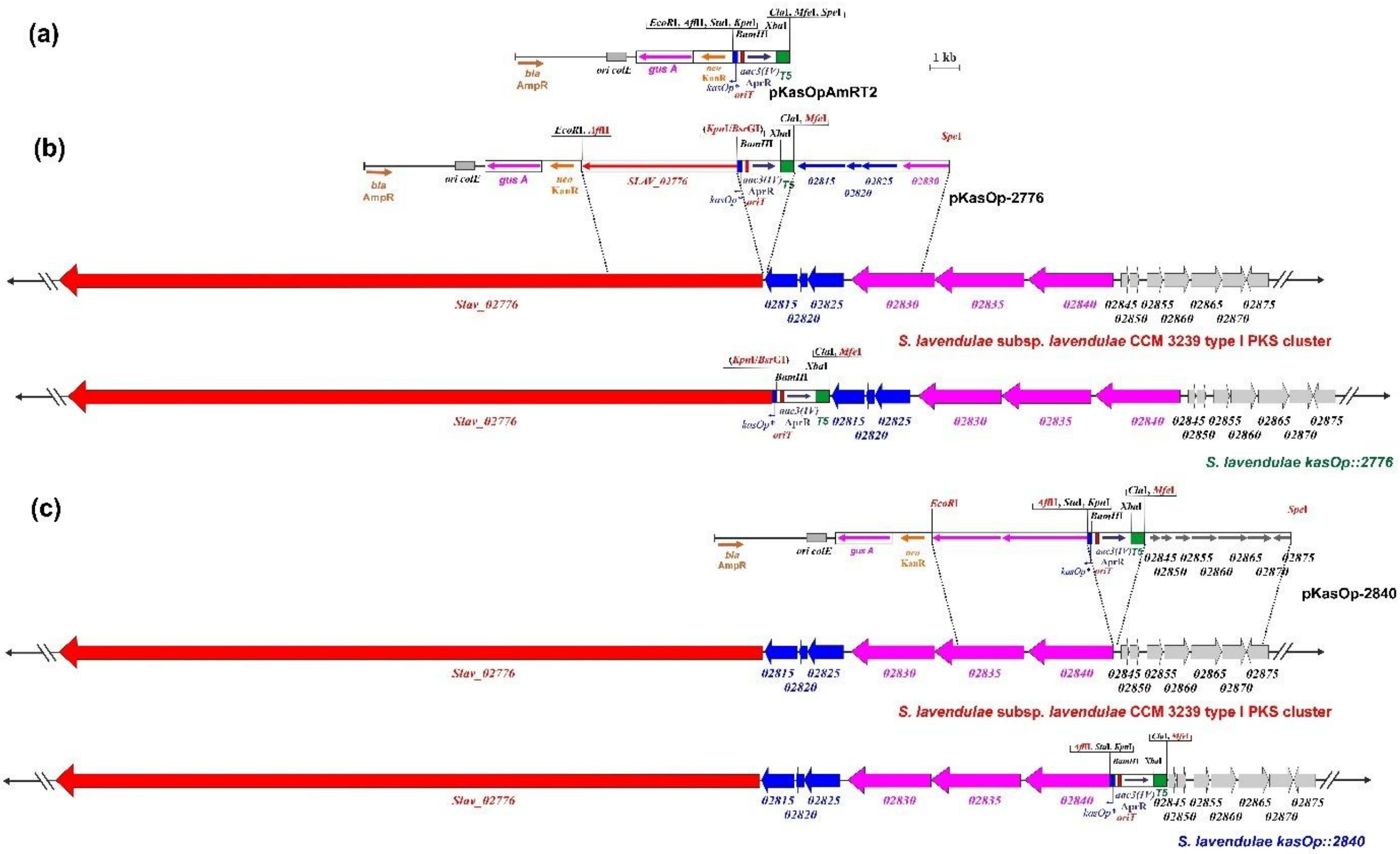

Fig. S4 Activation of the silent *stv* BGC for strevertene A2 production in *S. lavendulae* subsp. *lavendulae* CCM 3239 by insertion of the strong *kasOp** promoter upstream of the *Slav_02776* and *Slav_02840* genes in the genome. **a** Plasmid pKasOpAmRT2 containing a cassette with the strong *kasOp** promoter (Wang et al. 2013), the bidirectional *T5* terminator from the *S. lavendulae* subsp. *lavendulae* CCM 3239 genome (Javorova et al. 2024), and the *aac3(IV)* AprR gene with the *oriT* origin of transfer from plasmid pIJ773 (Gust et al. 2003), cloned into the backbone of *E. coli* plasmid pBluescript II KS+ containing the ColE1 replication origin and the *bla* AmpR gene. **b** Scheme of the recombinant plasmid pKasOp-2776, containing 5-kb regions upstream and downstream of the *Slav_02776* gene in plasmid pKasOpAmRT2, and the WT and mutated genomic regions showing integration of the *T5*–AprR–*kasOp** cassette upstream of *Slav_02776*, encoding a type I PKS. **c** Scheme of the recombinant plasmid pKasOp-2840, containing 5-kb regions upstream and downstream of the *Slav_02840* gene in plasmid pKasOpAmRT2, and the corresponding WT

and mutated genomic regions showing integration of the *T5*–AprR–*kasOp** cassette upstream of *Slav_02840*, encoding a LuxR-family regulatory protein. For panels **b** and **c**, thick arrows indicate the direction and size of genes; red arrows represent type I PKS biosynthetic genes, blue arrows tailoring biosynthetic genes, and pink arrows regulatory genes. Blue boxes with bent arrows indicate the position of the *kasOp** promoter. Gene labeling is based on the *S. lavendulae* subsp. *lavendulae* CCM 3239 genomic sequence (GenBank accession no. CP024985).

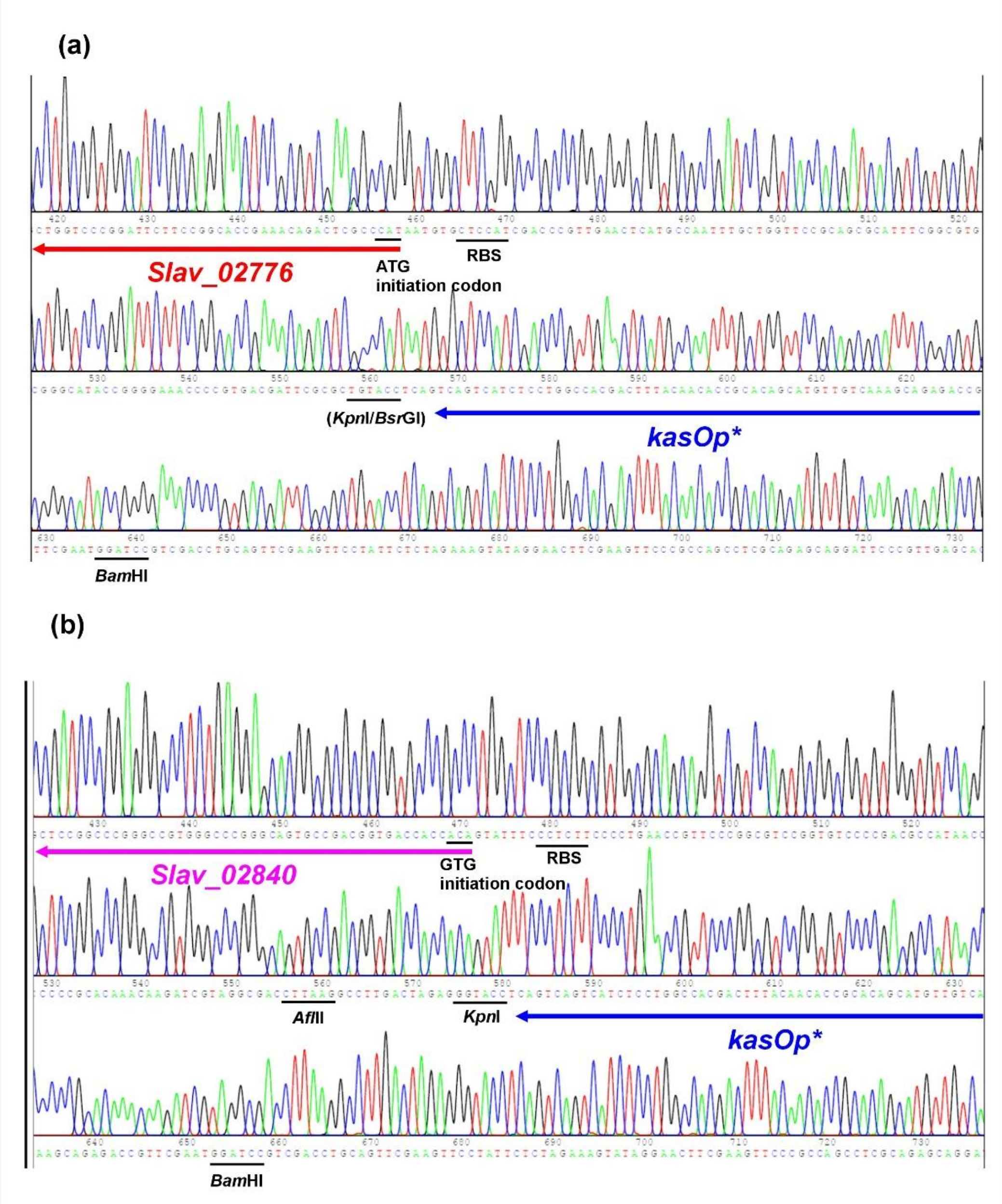

Fig. S5. Verification of correct integration of the *T5*-AprR-*kasOp** cassette into the chromosome of *S. lavendulae kasOp::2776* **a** and *S. lavendulae kasOp::2840* **b** strains by nucleotide sequencing of adjacent chromosomal regions. Shown are representative nucleotide sequences from validated ~3-kb PCR fragments amplified using the 2776seqR/2815seqD primers (Table S3) and sequenced using 2776seqR **a,** and from and a 3-kb PCR fragment amplified using the 2840seqR/2845seqD primers (Table S3) and sequenced with 2840seqR **b**. Arrows indicate positions and orientations of the *kasOp** promoter and neighboring genes, with RBS and initiation codons indicated. Relevant restriction sites are shown within the sequence.

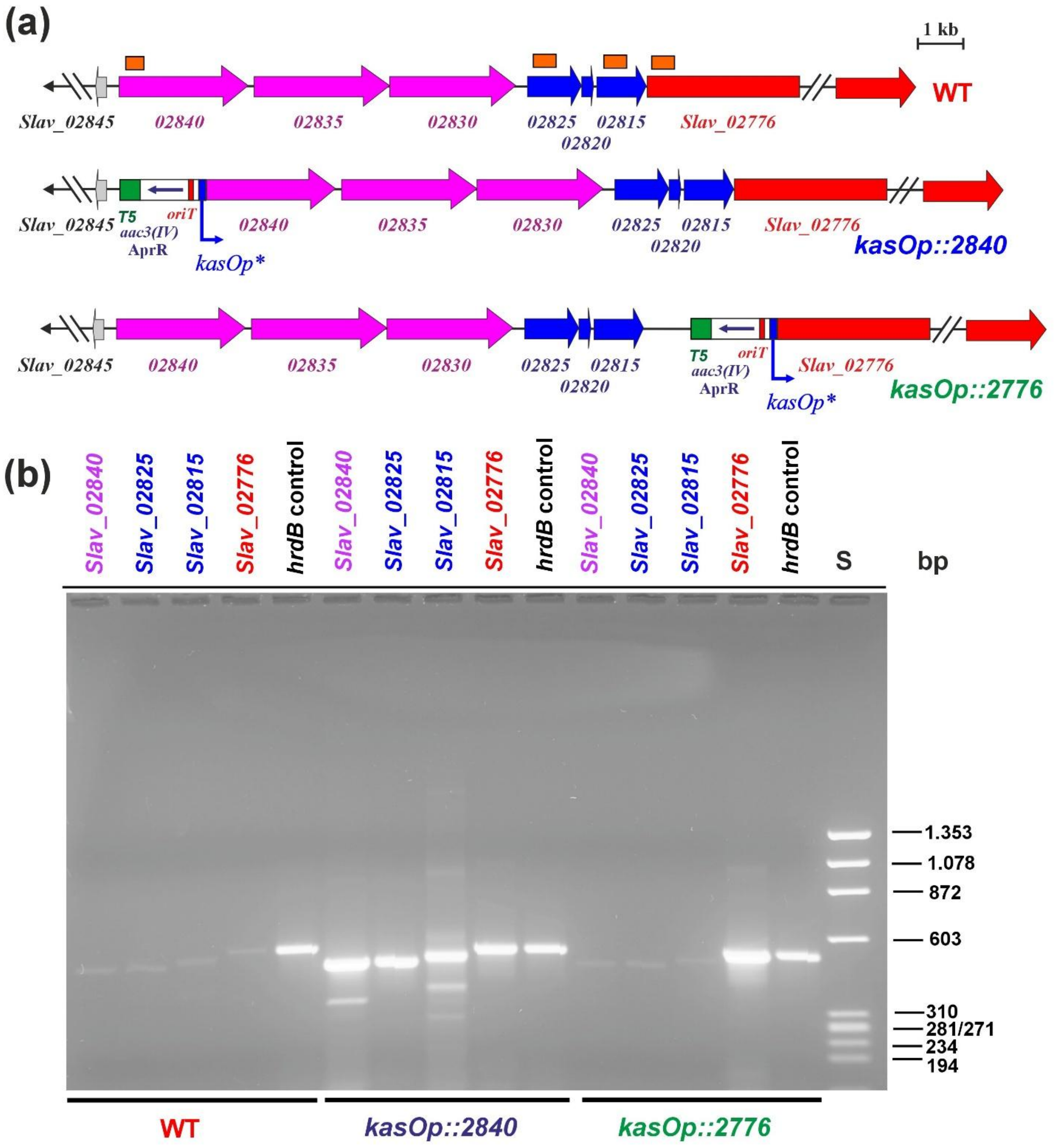

Fig. S6. Transcriptional analysis of four selected genes from BGC2 (*Slav-02840*, *Slav_02825*, *Slav_02815*, *Slav_02776*) by RT-PCR in WT, *S. lavendulae kasOp::2776* and *kasOp::2840* mutants. **a** Scheme of WT and mutated genomic regions showing integration of the AprR–*kasOp** cassette upstream of the *SLAV_02840* and *SLAV_02776* genes. Thick arrows indicate the direction and size of genes. Blue boxes with bent arrows indicate the position of the *kasOp** promoter. The orange bar above the maps indicates the position of RT-PCR products. Gene labeling is based on the *S. lavendulae* subsp. *lavendulae* CCM 3239 genomic sequence (GenBank accession no. CP024985). **b** RT-PCR analysis of four selected genes from type I PKS BSC in wild-type *S. lavendulae* subsp. *lavendulae* CCM 3239 (WT), *S. lavendulae kasOp::2840* and *kasOp::2776* mutants using RNA isolated at 72 h. The *hrdB* gene was used as an internal control to normalize RNA samples. The products after RT-PCR reactions (details are in the Material and methods section) were separated by electrophoresis on a 2% (w/v) agarose gel and stained with EtBr. *Hae*III-digested PhiX174 DNA was used as a size standard (lane S).

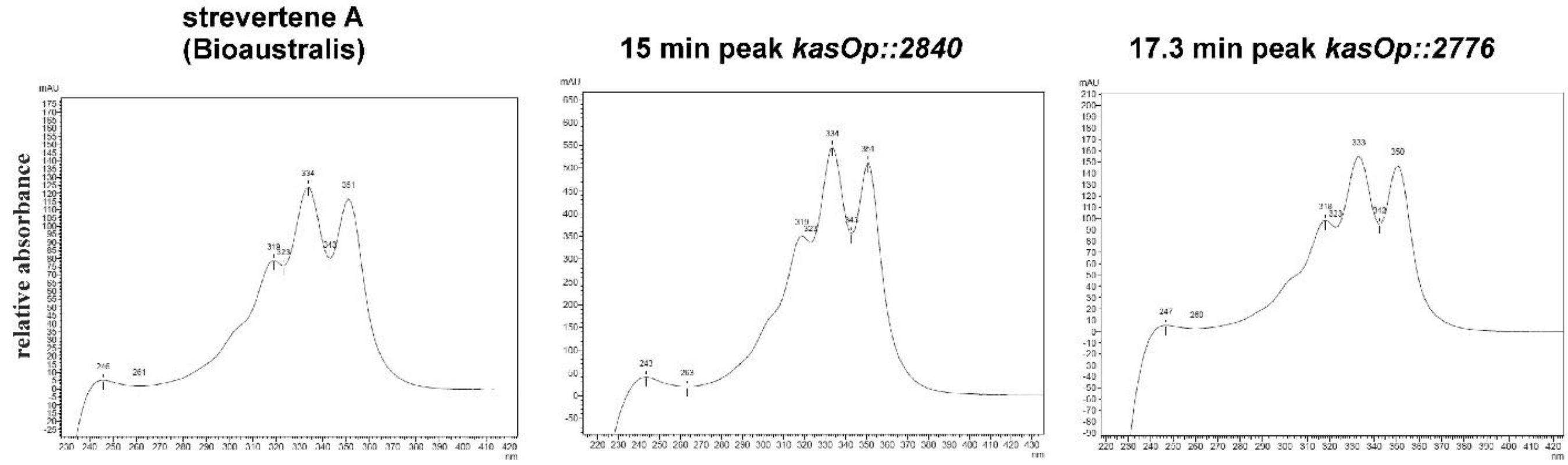

Fig. S7. UV/VIS spectra of strevertene A (Bioaustralis), the HPLC peak at Rt = 15 min from the S. *lavendulae kasOp::2840* mutant, and the HPLC peak at Rt = 17.3 min from the *S. lavendulae kasOp::2840* mutant. The spectra show polyene characteristic λmax values at 319 nm, 334 nm, and 351 nm, consistent with previously reported data (Schlingmann et al. 1999).

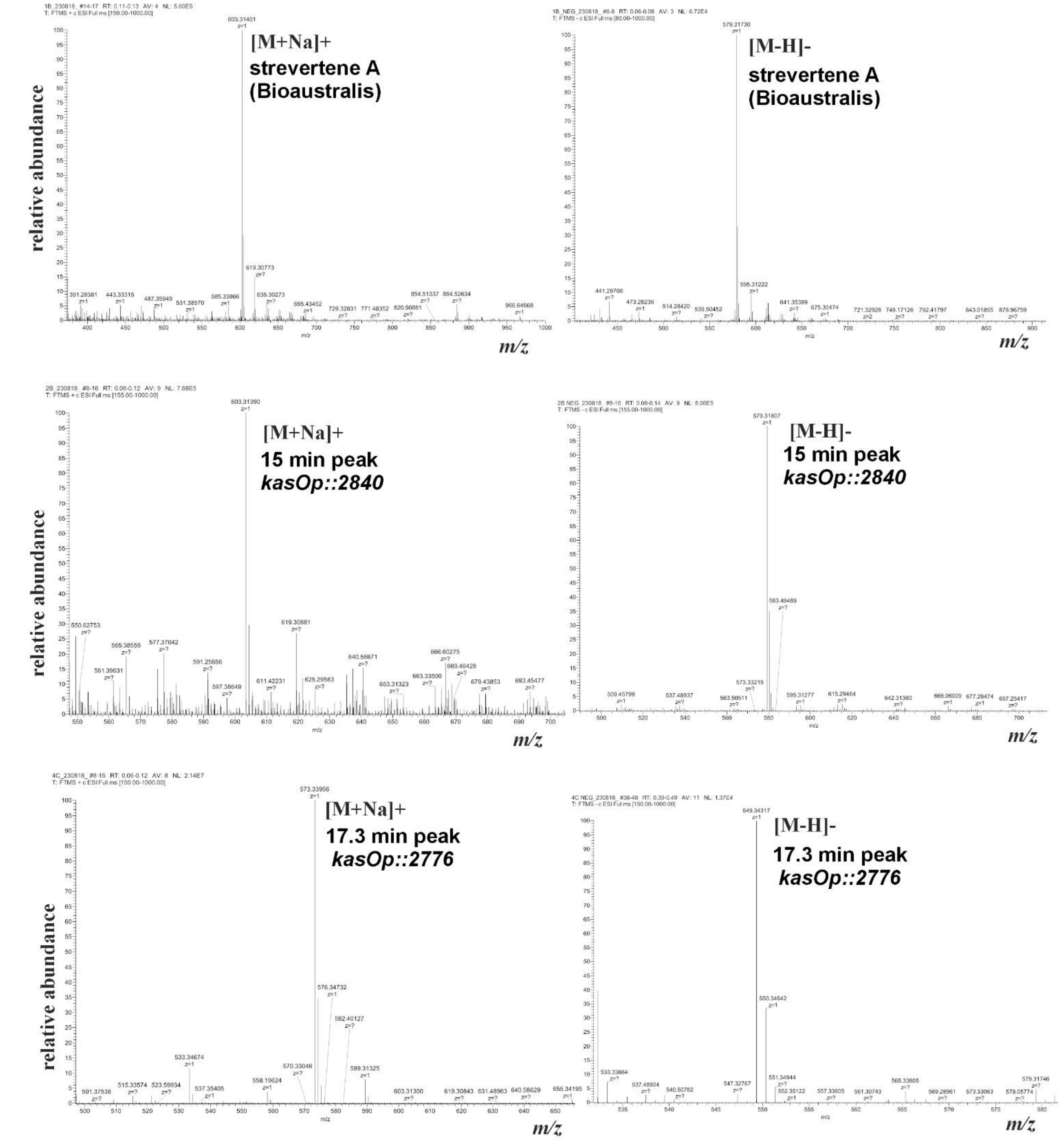

Fig. S8. HR-ESI-MS spectra in positive and negative ion modes for strevertene A (Bioaustralis), the HPLC peak at Rt = 15 min from the S. *lavendulae kasOp::2840* mutant, and the HPLC peak at Rt = 17.3 min from the *S. lavendulae kasOp::2776* mutant.

Structural elucidation of strevertene A2

The ^{1}H NMR signals of strevertene A2 were observed approximately in the spectral window 0.5–6.5 ppm (Fig. S9). Three doublet-shaped methyl signals were detectable at δ_H = 1.010, 1.068, and 1.181 ppm. It was assumed that the substance could belong to the strevertene compound class. The protons of the methyl group at δ_H = 1.068 ppm showed long-range correlations with three carbon nuclei (δ_C = 46.29, 72.03, and 173.74 ppm), the latter corresponding to a carbonyl group (Fig. S16). These carbon chemical shifts were assigned to C2, C3, and C1, respectively (see Table S4 for the used atom labeling), placing the methyl group at position 2a. The corresponding ^{1}H chemical shifts of H2 and H3 (δ_{H2} = 2.257 ppm, δ_{H3} = 3.661 ppm) were obtained from the HSQC spectrum (Figs. S14 and S15), and their assignment was confirmed by COSY correlations between H2a–H2 and H2–H3 (Fig. S12). HSQC signal polarity indicated that C3 belongs to a CH group, and the elevated ^{1}H and ^{13}C chemical shifts suggested the presence of a directly bonded electronegative atom. H3 exhibited further COSY interactions with protons at δ_H = 1.280 and 5.161 ppm. The latter proton lacked any HSQC correlation, consistent with direct bonding to a heteroatom (assigned as 3-OH), whereas the former signal corresponded to a CH_2 group (δ_C = 41.49 ppm) with two diastereotopic protons. A weak HMBC correlation between H3 (δ_H = 3.661 ppm) and a carbon at δ_C = 71.11 ppm suggested the assignment of this ^{13}C NMR signal to C5 and indicated a hydroxylated group. The hydroxylation was proven by the comparison of HMBC correlation patterns of proton 3-OH (δ_H = 5.161 ppm) and another *O*-bonded proton δ_H = 4.807 ppm – these two protons shared an HMBC interaction with carbon C4 (δ_C = 41.49 ppm), thus suggesting the identification of the latter proton as 5-OH. Additional HMBC correlations of 5-OH with C6 (δ_C = 38.07) and C5 (δ_C = 71.11 ppm) confirmed these assignments. The HSQC spectrum revealed the positions of H5 and H6 (δ_{H5} = 3.514 ppm; δ_{H6} = 1.078 ppm, CH2 group).

Due to a partial overlap of ^{1}H signals in the aliphatic region, a HSQC-TOCSY spectrum and selective 1D TOCSY spectra were recorded. A TOCSY magnetization transfer was visible from the ^{1}H–^{13}C pairs at positions 3, 4, 5, and 2a onto the proton H2 (δ_{H2} = 2.257 ppm) in the HSQC-TOCSY spectrum. This was implied by the ^{13}C coordinates of the correlation signals observed at the frequency of H2 (encoded to the magnetization of H3, H4, H5, and H2a during the evolution period prior to the spin lock) (Fig. S17). When analyzing the HSQC-TOCSY transfer to H3 (δ_{H2} = 3.661 ppm), correlation signals were observed with the f1-frequencies of C2, C4, C5, C6, C2a, and also at a frequency of δ_C = 22.34 ppm; the latter was assumed to be the chemical shift of C7. Using the same method, the resonance frequency of C8 was determined from the observation of a correlation signal at the coordinates δ_{H5} = 3.514 ppm and δ_C = 38.71 ppm. The corresponding ^{1}H chemical shifts of H7 and H8 were obtained from HSQC (δ_{H7} = 0.944 and 1.697 ppm; δ_{H8} = 1.097 ppm). The HSQC-TOCSY spectrum further enabled the assignment of position 9: the ^{1}H-^{13}C pair with δ_{H9} = 3.457 ppm and δ_{C9} = 71.83 ppm transferred its magnetization to H6, H7, and H8 during a 60 ms spin lock. COSY and HMBC interactions of the proton at δ_H = 4.849 ppm (9-OH) supported the assignment, observing HMBC correlations to C8, C9, and an unassigned carbon at δ_C = 42.83 ppm, found to be C10. After the extraction of the ^{1}H NMR frequencies of the H10 protons from HSQC (δ_{H10} = 1.121 and 1.300 ppm), the COSY spectrum was inspected – the chemical shift value of H11 was obtained straightforwardly (δ_{H11} = 3.783 ppm). The proton H11 further possessed a homonuclear interaction with a proton resonating at δ_H = 1.465 ppm. The latter proton was found to belong to a CH_2 group, with δ_H = 1.465 and 1.504 ppm, and δ_C = 40.78 ppm, as observed in HSQC; the group was identified as a methylene group at position 12. It was noticed that the ^{13}C NMR signal of C12 was broader than the previous ^{13}C NMR signals. The absence of any COSY and HMBC signals, that could provide the means of the identification of the groups at positions 13 and 14 was, again, compensated by the information gained from the HSQC-TOCSY spectrum: magnetization transfer to H11 and H12 (δ_{H11} = 3.783 ppm; δ_{H12} = 1.465 and 1.504 ppm) revealed a ^{1}H–^{13}C pair at δ_H = 3.309 ppm and δ_C = 66.78 ppm, assigned to C13.

The lack of 2D NMR correlation signals due to rapid T2-relaxation drew further attention to position 27 of the molecule. The existence of the lactone moiety was proven by the observation of

the HMBC signal at δ_{H27} = 4.638 ppm and δ_{C1} = 173.74 ppm. H27 showed COSY interactions with δ_H = 1.181 ppm (methyl at position 28) and δ_H = 2.345 ppm (CH at position 26). Additional assignments included methyl at the position 26a (δ_{H26a} = 1.010 ppm; δ_{C26a} = 15.65 ppm) and an sp^2 CH group in position 25 (δ_{H25} = 5.910 ppm; δ_{C25} = 136.35 ppm), consistent with the pentaene chain. Stepwise inspection of COSY, HSQC, and HMBC from H25 enabled the assignment of CH groups at positions 22–24. TOCSY (150 ms) from H25 revealed correlations with δ_H = 4.073 and 5.524 ppm, corresponding to CH15 and CH16 (δ_C = 71.57 and 134.80 ppm).

After a partial success in the assignment of selected proton and carbon signals belonging to the unsaturated chain, attention was focused on the identification of the group at position 14. A broad COSY signal was noticed between H15 (δ_{H15} = 4.073 ppm) and a proton with a broad signal at δ_H = 2.371 ppm. Interestingly, no HSQC signal was found at the latter ^{1}H frequency. As in the case of the signals of 3-OH, 5-OH, and 9-OH, this fact could point out that the previously mentioned signal also belongs to a proton directly bonded to a heteroatom. However, it has been speculated that another reason for its absence could be rapid T2-relaxation, leading to broad signal shapes or potential signal disappearance due to long pulse experiments. In the HSQC-TOCSY spectrum, a faint correlation signal was visible at the coordinates of δ_{H15} = 4.073 ppm and δ_C = 56.64 ppm. Despite difficulties, this carbon signal was identified as C14 and found to carry the proton δ_H = 2.371 ppm, now denoted as H14 – this was determined from ^{13}C and HSQC NMR measurements at 45 °C. The temperature was elevated to alter the rate of any dynamic equilibrium that might be causing the signal broadening.

HR-ESI-MS confirmed the molecular formula $C_{31}H_{48}O_{10}$ (Fig. S8). The presence of a carboxylic acid was expected, likely bonded to C14, though its ^{13}C signal was broadened due to dynamic equilibrium and could not be directly observed. Attempts to shift the equilibrium (DCl in DMSO-d_6, elevated temperature) yielded minimal improvement; high temperatures in the presence of acid caused partial decomposition.

Comparison with literature data showed that its structure matches that of RKGS-A2215A from *S. lavendulae* FRI-5 (Yoshioka et al. 2021) with excellent agreement of ^{1}H and ^{13}C chemical shifts (Table S4), supporting the positions and orientations of hydroxy groups. A broad peak at ~174.7 ppm (^{13}C NMR at 45 °C) was noted but could not be unambiguously assigned. The ^{13}C NMR signal of the carboxyl C14a was observable as a broad singlet at δ_C = 174.56 ppm in a process sample which was contaminated by two co-eluates in the chromatographic process. The correct position of the carboxyl was proven by the observation of the correlation signals resulting from the existence of $^3J_{C14aH13}$, $^2J_{C14aH14}$, $^3J_{C14aH15}$ in the HMBC spectrum measured from the above-mentioned sample. It was noted that the ^{13}C NMR shift of C14a slowly changed overtime. Previous work indicated acetylation was required to detect C14 and C14a (Yoshioka et al. 2021). Dissolution in CD_3OD led to rapid decomposition.

Table S4. NMR data for strevertene A2 in DMSO-d_6.

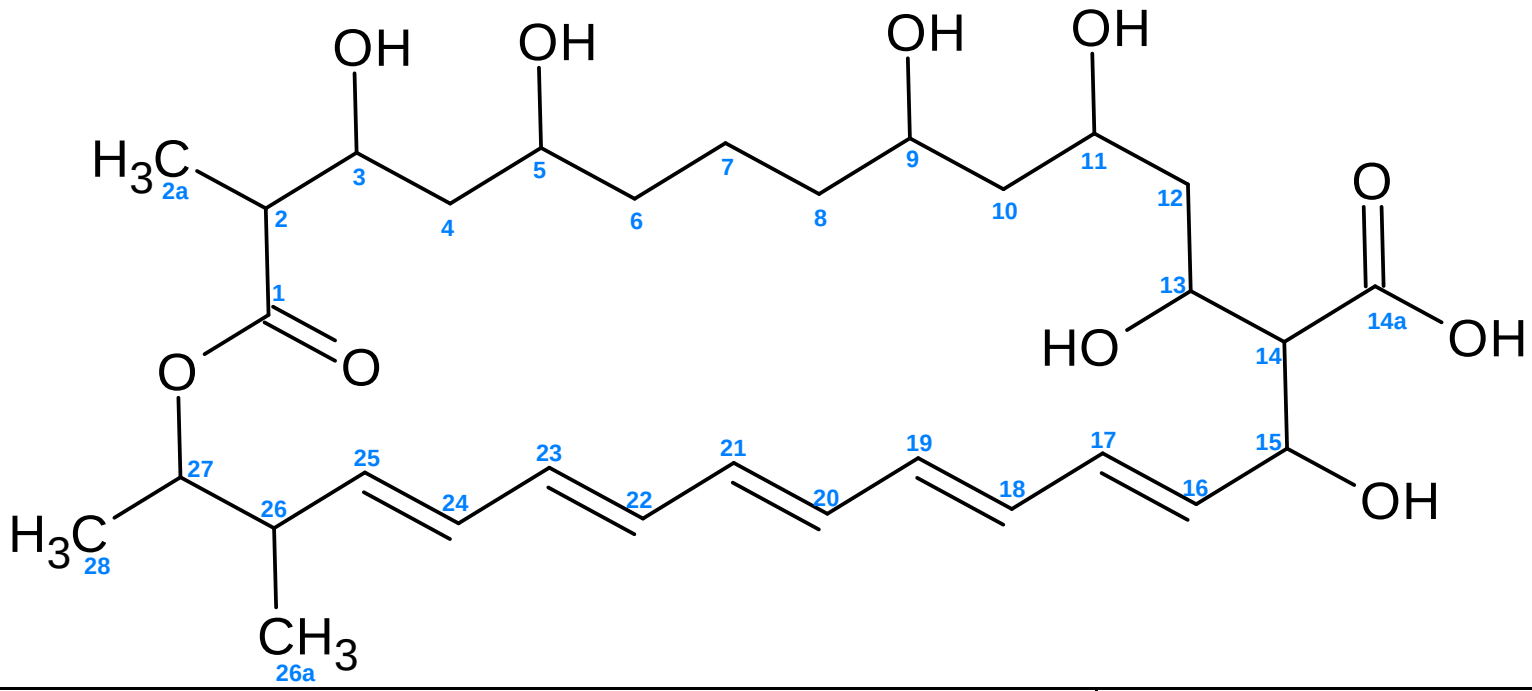

Position	Experimental data			Data of RKGS-A2215A by (Yoshioka et al. 2021)	
	δ_H / ppm	multiplicity; *J* / Hz	δ_C / ppm	δ_H / ppm	δ_C / ppm
1	-	-	173.74	-	173.8
2	2.257	dq; 7.9, 7.0	46.29	2.26	46.4
3	3.661	ddt; 8.8, 7.9, 3.5	72.03	3.65	72.1
4	1.270	m	41.49	1.26	41.6
	1.281	m			
5	3.514	m	71.11	3.51	71.2
6	1.078	m	38.07	1.03-1.11	38.2
7	0.944	m	22.34	0.93	22.4
	1.697	m		1.69	
8	1.097	m	38.71	1.03-1.11	38.8
9	3.457	m	71.83	3.46	72.0
10	1.121	m	42.83	1.09	42.9
	1.300	m		1.28	
11	3.783	br.t; 8.2	69.38	3.77	69.5
12	1.465	m	40.78	1.46	40.8
	1.504	m		1.51	
13	3.309	m	66.78	3.35	66.8
14	2.371	m	56.77[a]	2.41	57.0
15	4.073	br.t; 8.5	71.57	4.07	71.6
16	5.524	dd; 14.7, 8.8	134.80	5.51	134.8
17	6.147	m	131.39	6.16	131.6
18	6.258	m	131.20	6.20-6.28	131.2
19	6.225-6.323	m	133.17		131.9-133.4
20			131.85/133.17[b]		
21			131.85/133.17[b]		
22	6.232	m	131.14		
23	6.246	m	133.08		133.3
24	6.113	m	129.19	6.26	133.3
25	5.910	dd; 15.5, 6.5	136.35	5.91	136.4
26	2.345	m	39.65	2.35	40.0
27	4.638	dq; 8.9, 6.2	73.68	4.67	73.8
28	1.181	d; 6.2	18.75	1.18	18.9
2a	1.068	d; 7.0	13.73	1.07	13.9
14a	-	-	174.56[c]	-	174.6
26a	1.010	d; 6.9	15.65	1.01	15.7
3-OH	5.161	d; 3.5	-	5.17	-
5-OH	4.807	d; 3.9	-	4.81	-
9-OH	4.849	br.s	-	4.86	-

[a] Value extracted from the ^{13}C NMR spectrum measured at 45 °C.
[b] Could not be unambiguously assigned. The signal at δ_C = 133.17 ppm had a double intensity due to overlap with C19.
[c] Value extracted from a ^{13}C NMR spectrum of a process sample. The signal was not detected in the final sample.

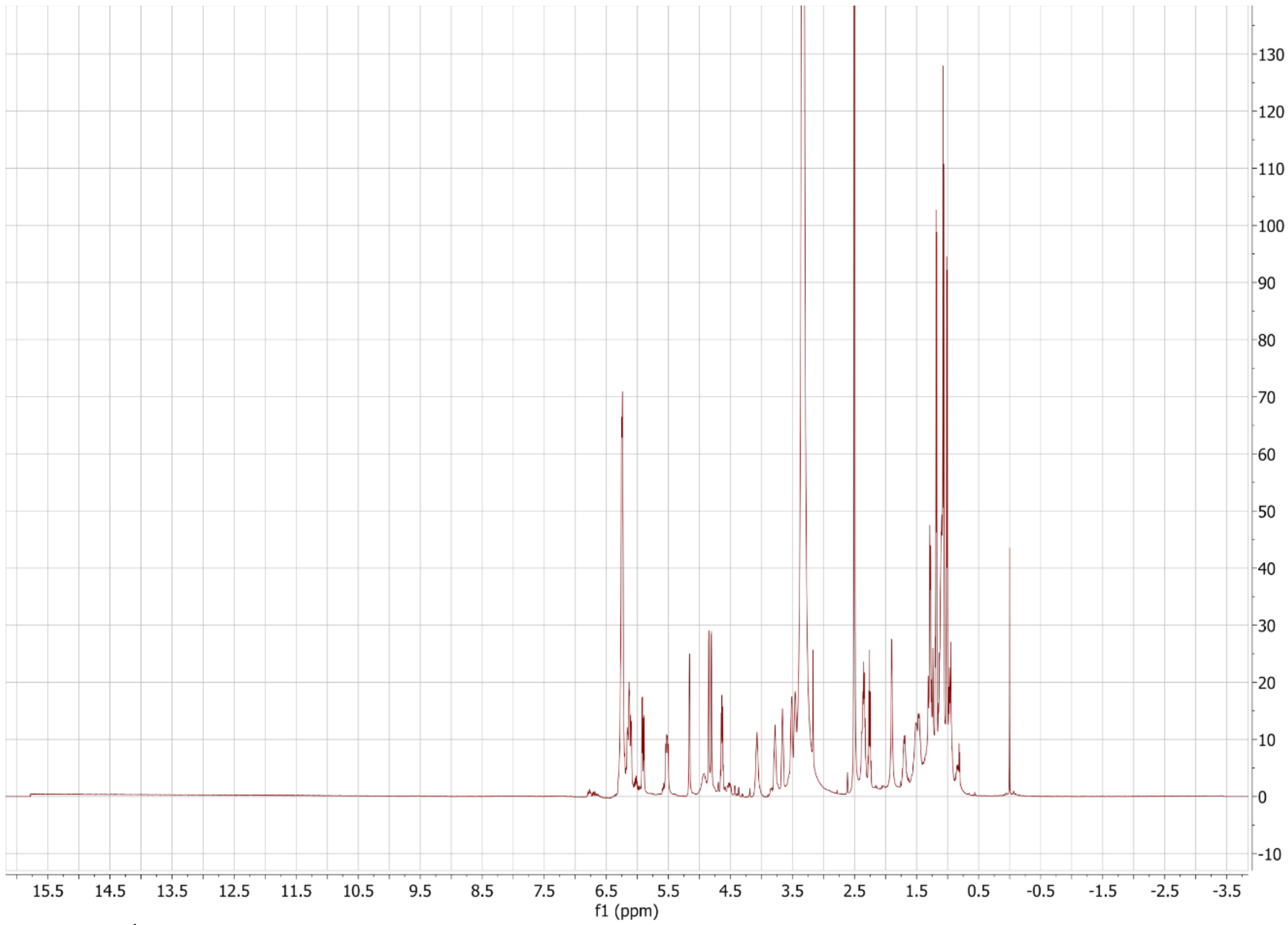

Fig. S9. ^{1}H NMR spectrum of strevertene A2 (DMSO-d_6, 600 MHz).

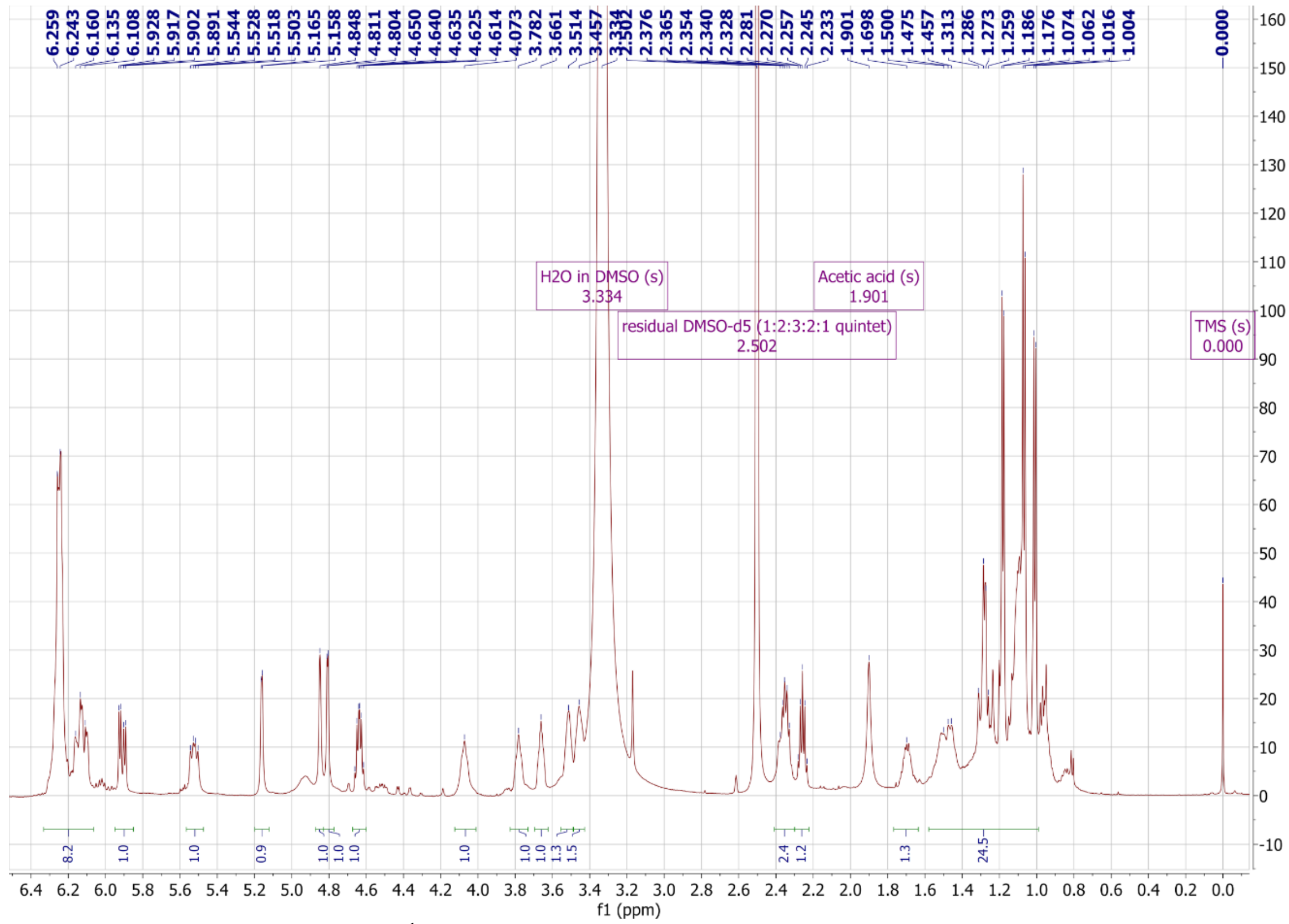

Fig. S10. Selected region of the ^{1}H NMR spectrum of strevertene A2 (DMSO-d_6, 600 MHz).

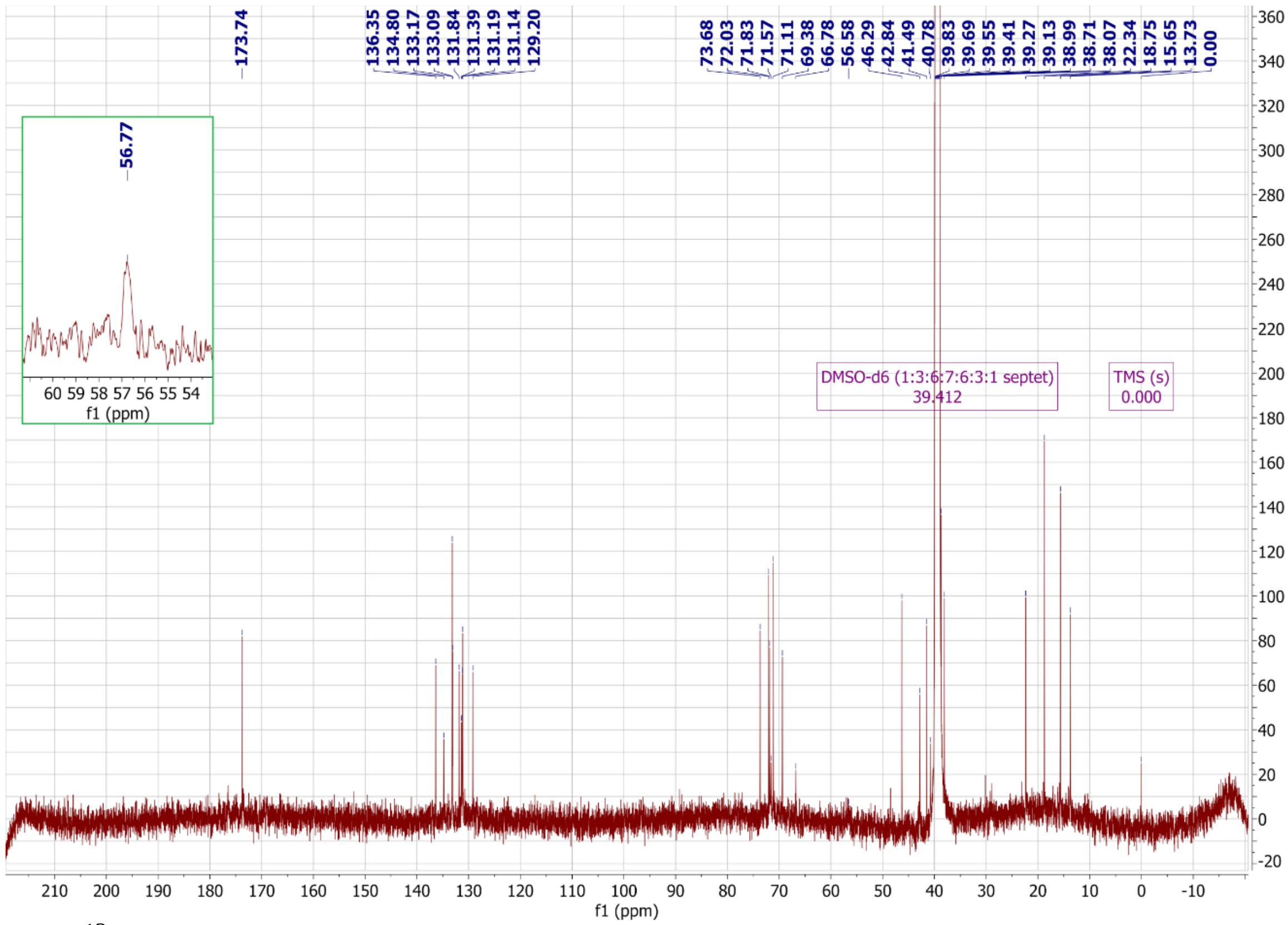

Fig. S11. ^{13}C NMR spectrum of strevertene A2 (DMSO-d_6, 151 MHz). The inset shows the broad signal of C-14 as observed at 45 °C after exponential apodization.

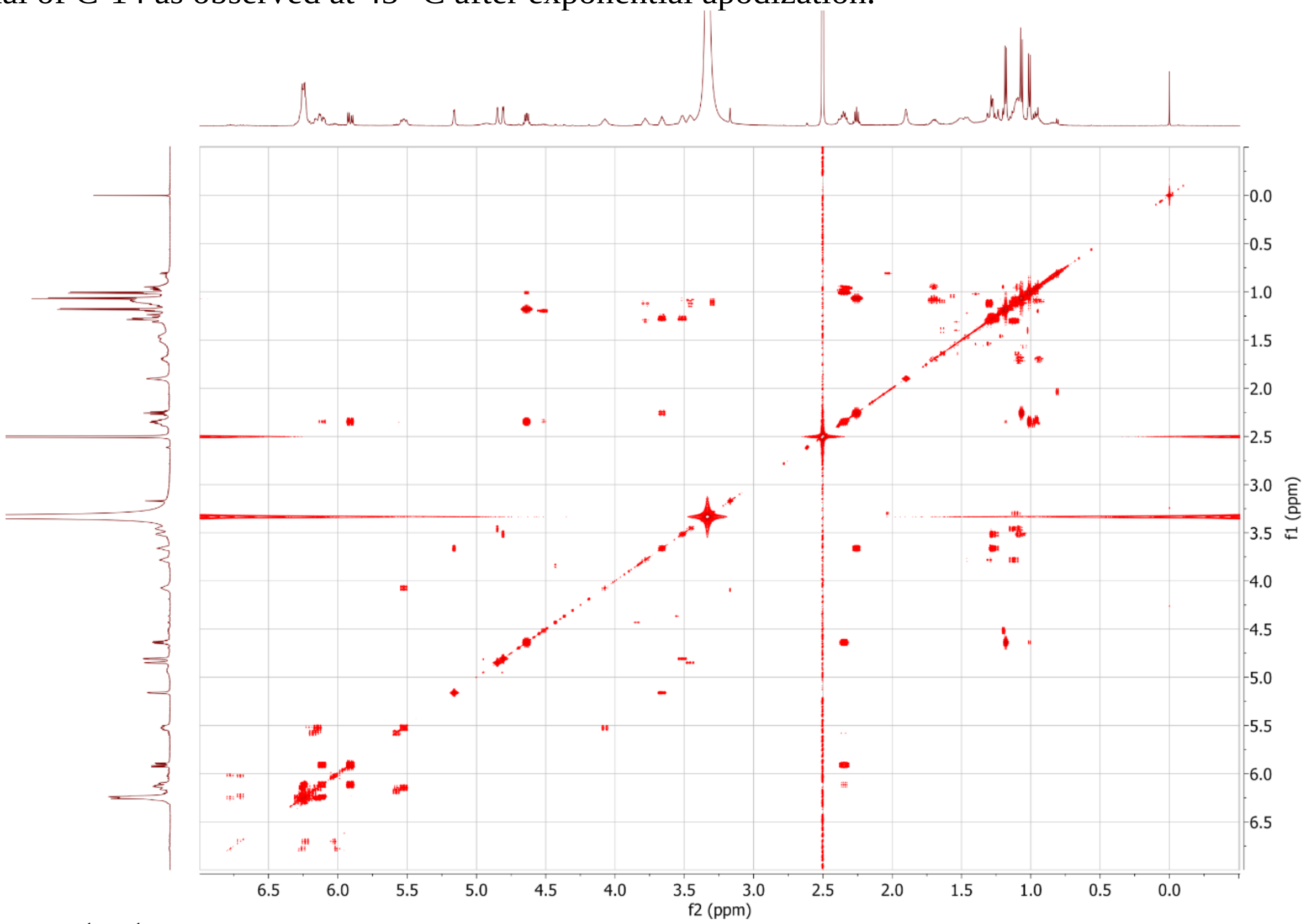

Fig. S12. ^{1}H-^{1}H COSY NMR spectrum of strevertene A2 (DMSO-d_6, 600/600 MHz).

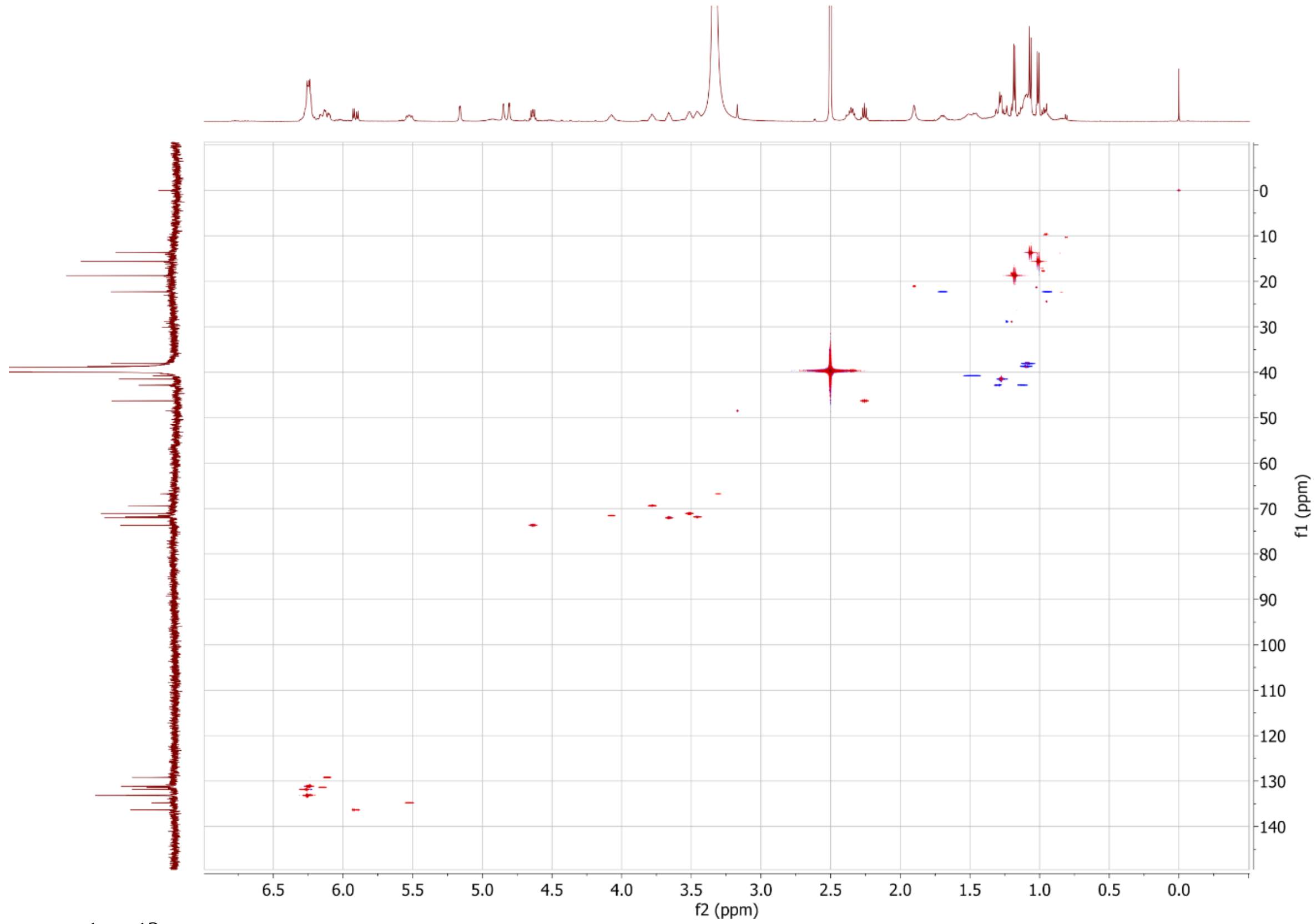

Fig. S13. ^{1}H-^{13}C HSQC NMR spectrum of strevertene A2 (DMSO-d_6, 600/151 MHz).

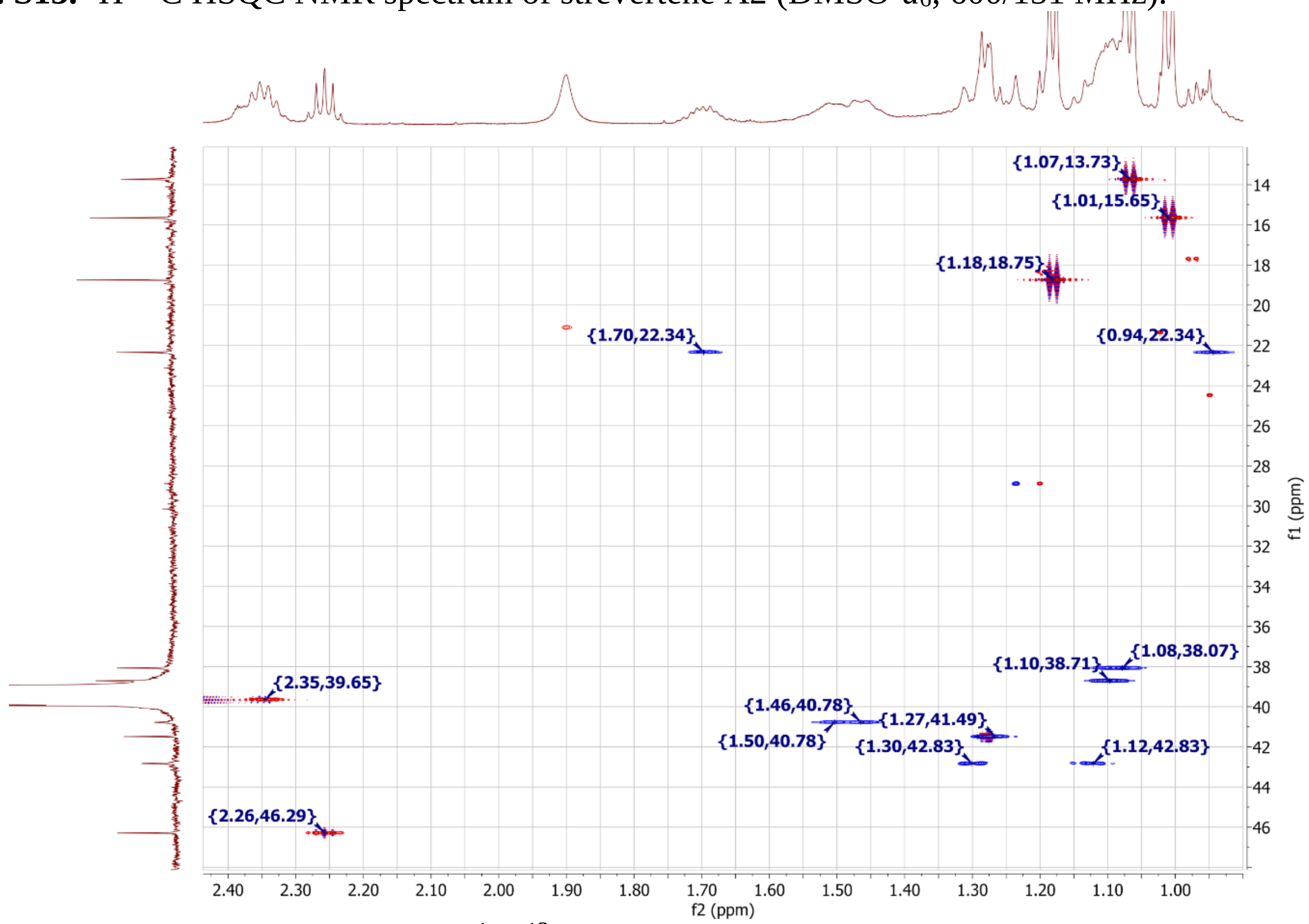

Fig. S14. A zoomed in section of the ^{1}H-^{13}C HSQC NMR spectrum of strevertene A2 (DMSO-d_6, 600/151 MHz).

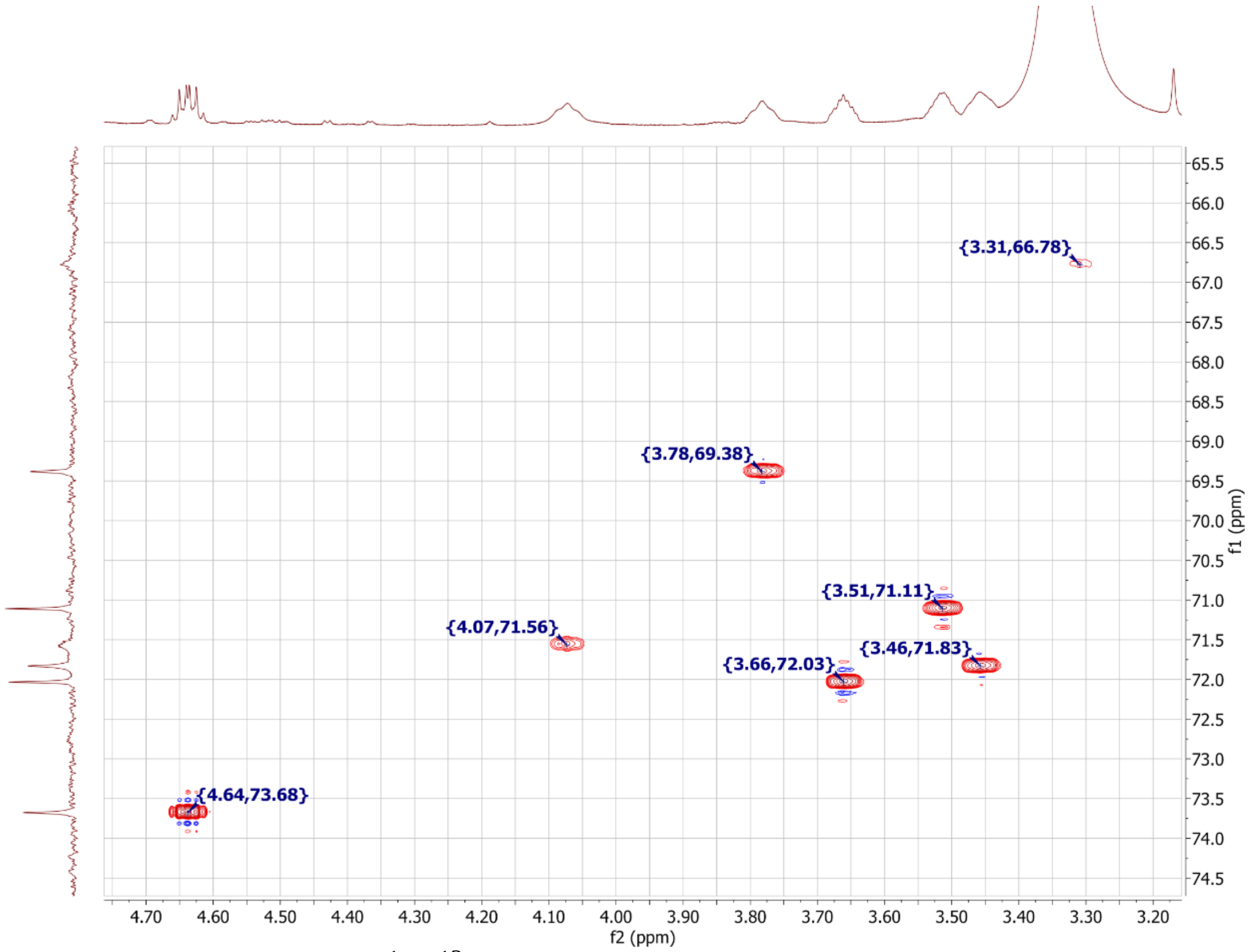

Fig. S15. A zoomed in section of the ^{1}H-^{13}C HSQC NMR spectrum of strevertene A2 (DMSO-d_6, 600/151 MHz).

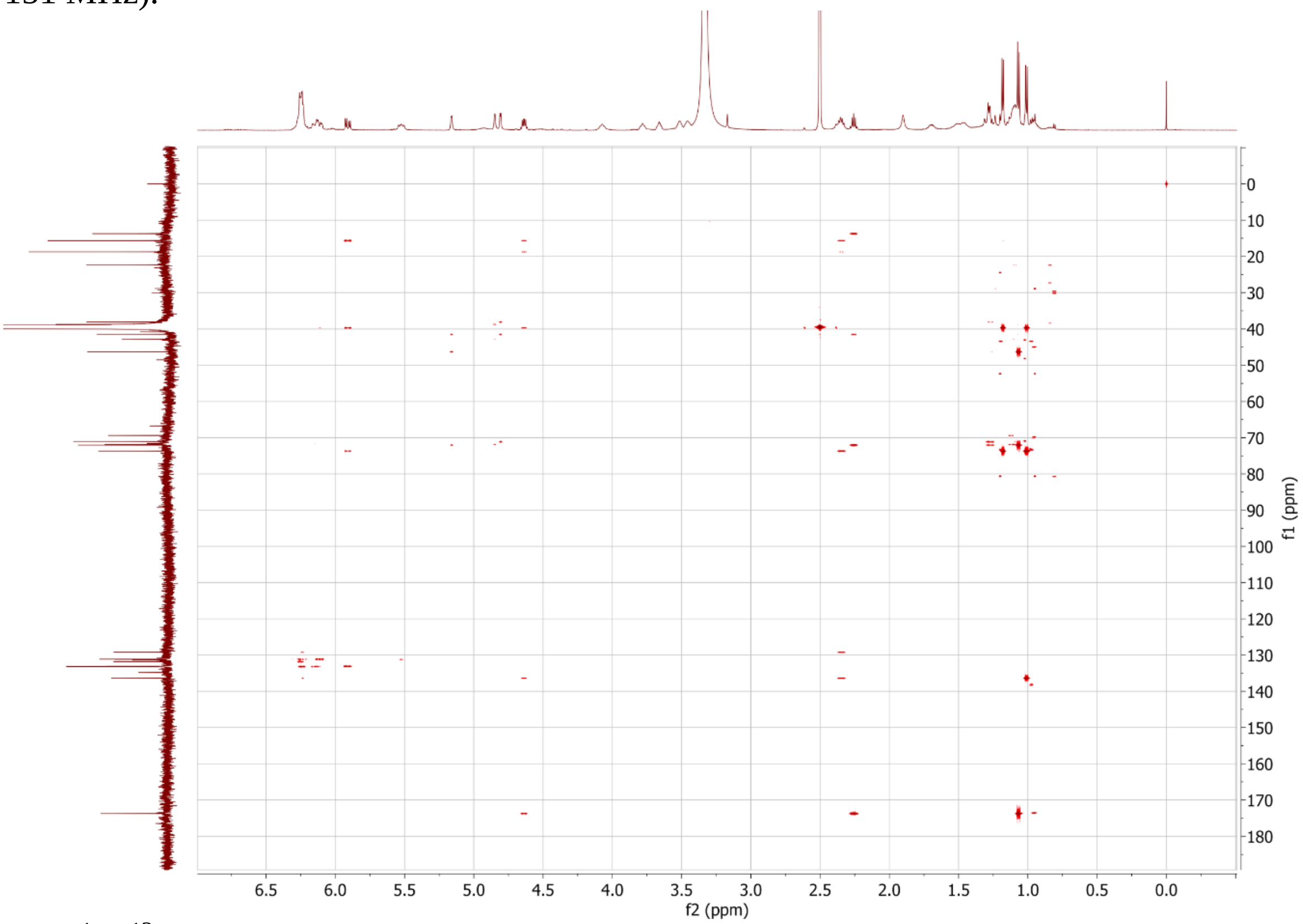

Fig. S16. ^{1}H-^{13}C HMBC NMR spectrum of strevertene A2 (DMSO-d_6, 600/151 MHz).

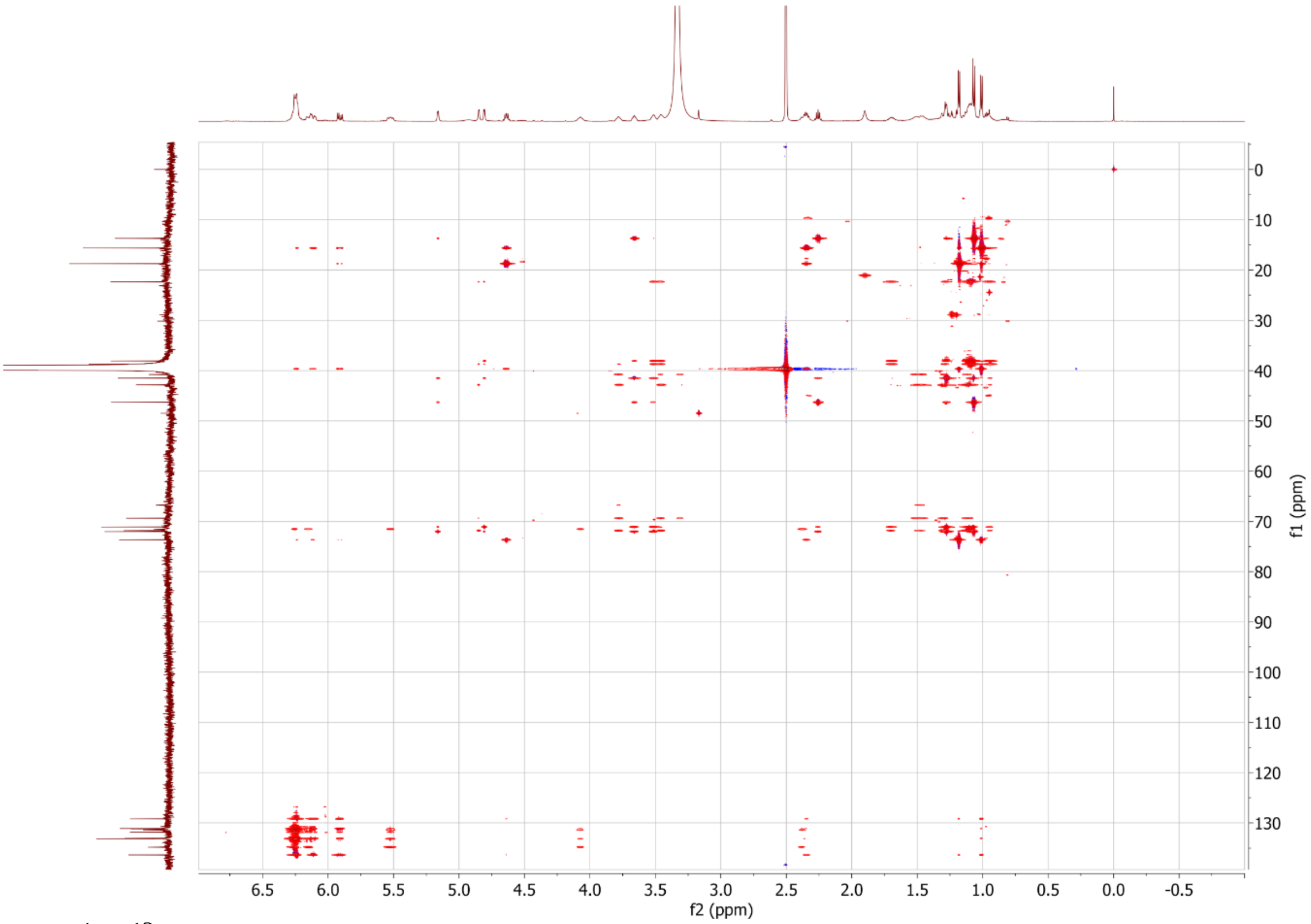

Fig. S17. ^{1}H-^{13}C HSQC-TOCSY NMR spectrum of strevertene A2 (DMSO-d_6, 600/151 MHz). The spinlock duration was set to 60 ms.

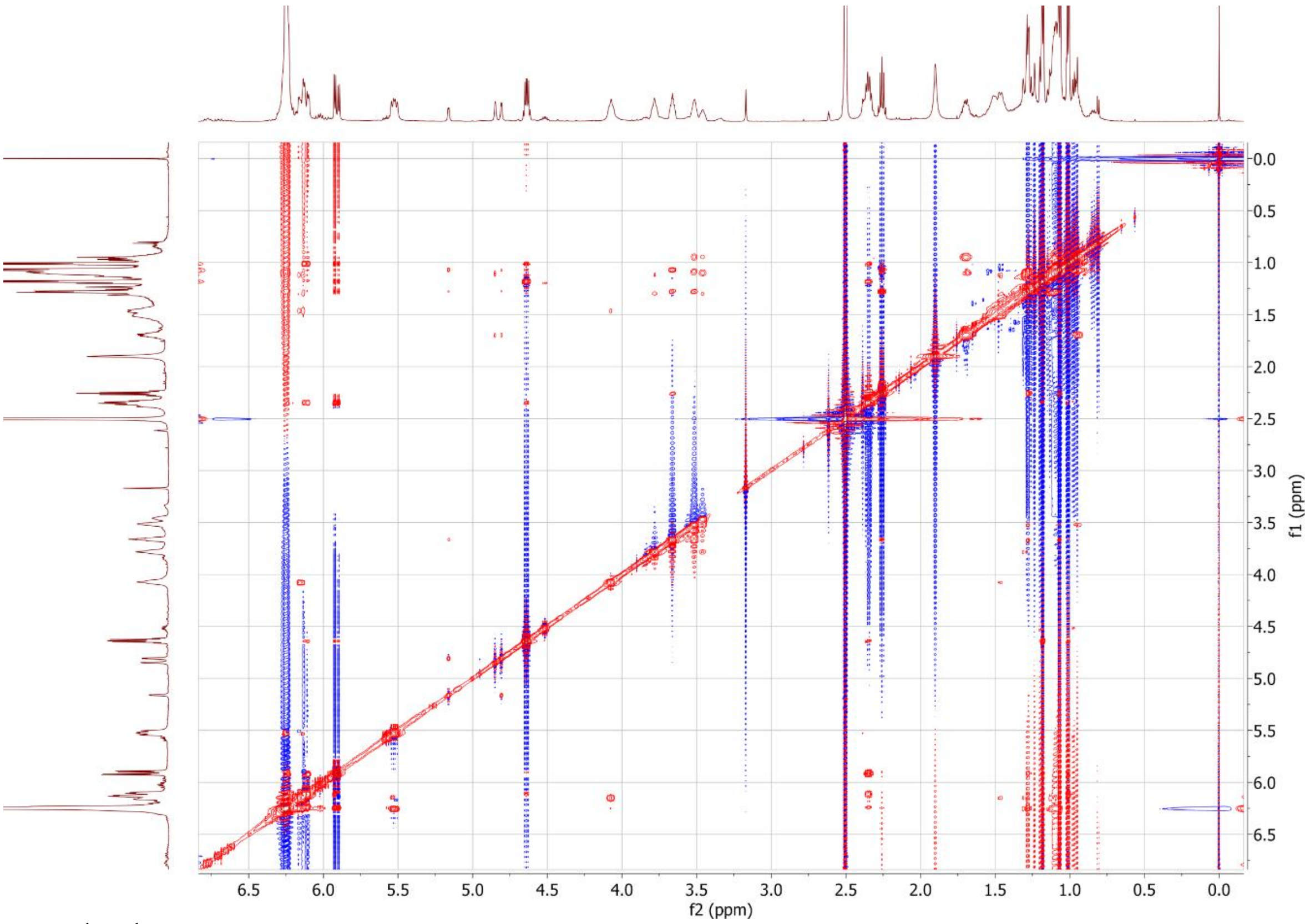

Fig. S18. ^{1}H-^{1}H NOESY NMR spectrum of strevertene A2 (DMSO-d_6, 600/600 MHz).

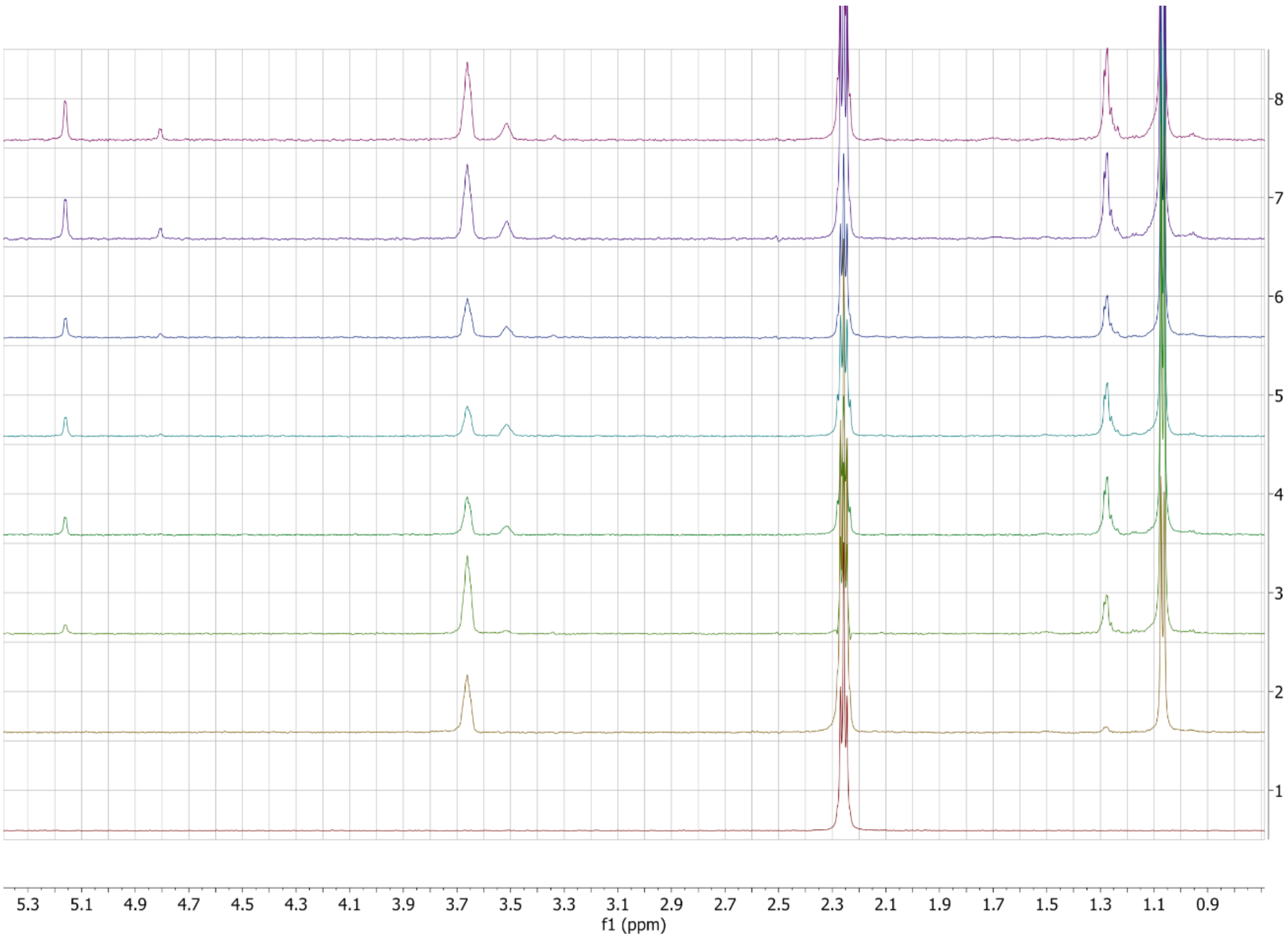

Fig. S19. A series of selective 1D NMR experiments of strevertene A2 (DMSO-d_6, 600 MHz). The spectrum 1 (bottom) is a selective excitation of the signal of proton H2 with the use of the double pulsed field gradient spin echo (dpfgse) selective excitation block. The spectra 2 to 8 are selective 1D TOCSY spectra obtained with the selective excitation of H2. With the ascending number of the spectrum, the spinlock durations within the 1D TOCSY experiment were set to: 0.020, 0.035, 0.050, 0.065, 0.080, 0.095, and 0.110 s.

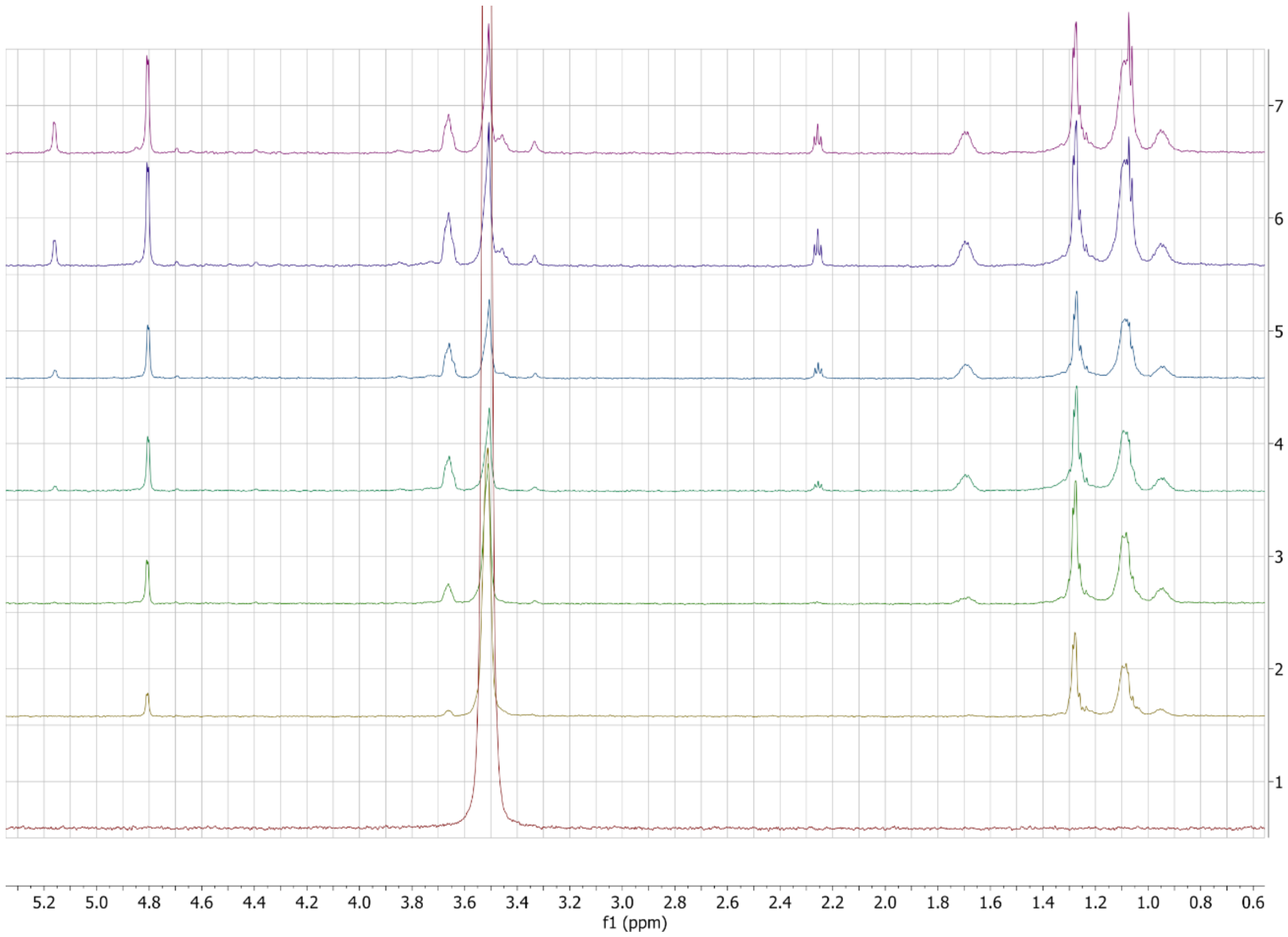

Fig. S20. A series of selective 1D NMR experiments of strevertene A2 (DMSO-d_6, 600 MHz). The spectrum 1 (bottom) is a selective excitation of the signal of proton H5 with the use of the double pulsed field gradient spin echo (dpfgse) selective excitation block. The spectra 2 to 7 are selective 1D TOCSY spectra obtained with the selective excitation of H5. With the ascending number of the spectrum, the spinlock durations within the 1D TOCSY experiment were set to: 0.015, 0.030, 0.045, 0.060, 0.075, and 0.090 s.

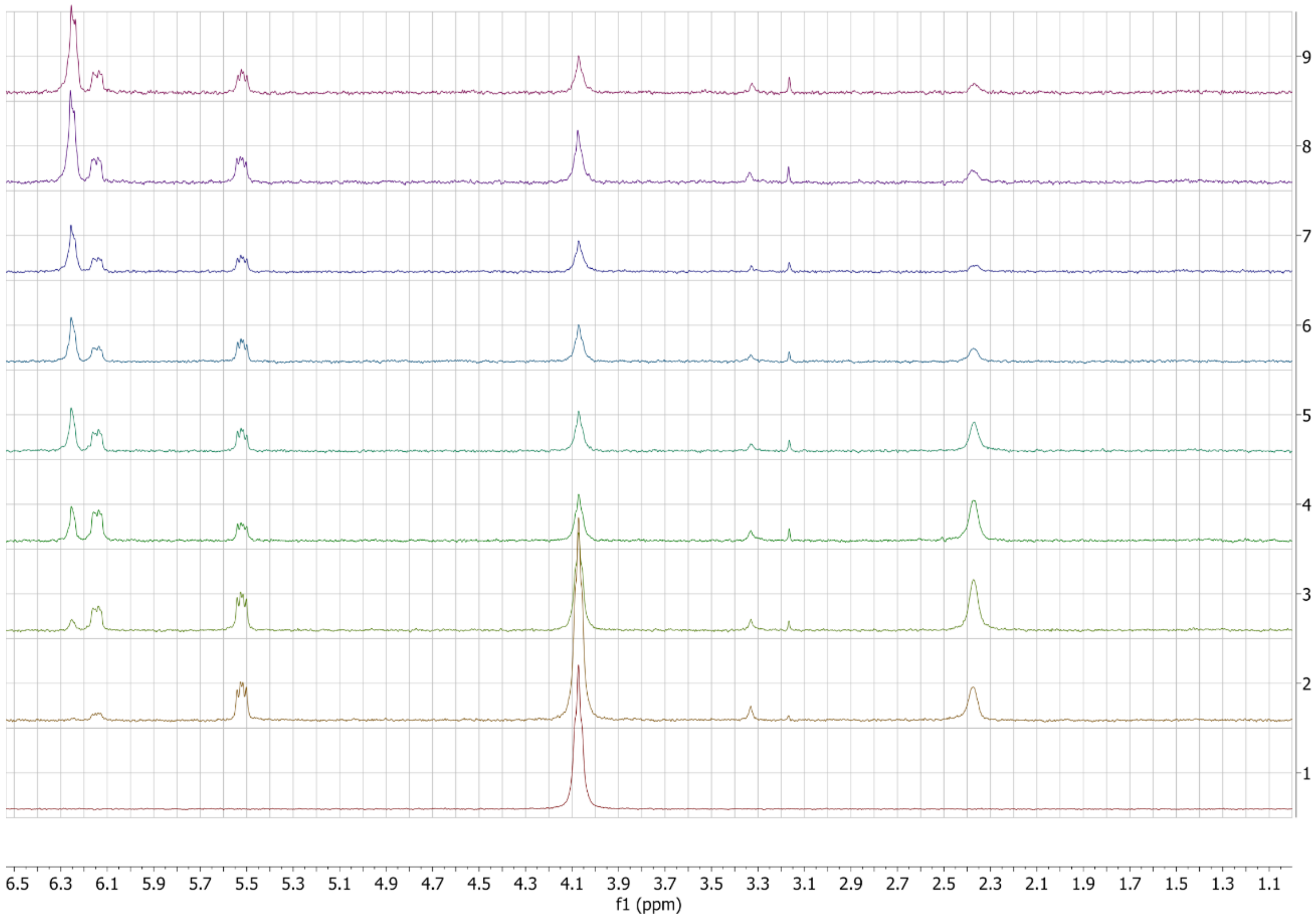

Fig. S21. A series of selective 1D NMR experiments of strevertene A2 (DMSO-d_6, 600 MHz). The spectrum 1 (bottom) is a selective excitation of the signal of proton H15 with the use of the double pulsed field gradient spin echo (dpfgse) selective excitation block. The spectra 2 to 9 are selective 1D TOCSY spectra obtained with the selective excitation of H15. With the ascending number of the spectrum, the spinlock durations within the 1D TOCSY experiment were set to: 0.015, 0.030, 0.045, 0.060, 0.075, 0.090, 0.105, and 0.120 s.

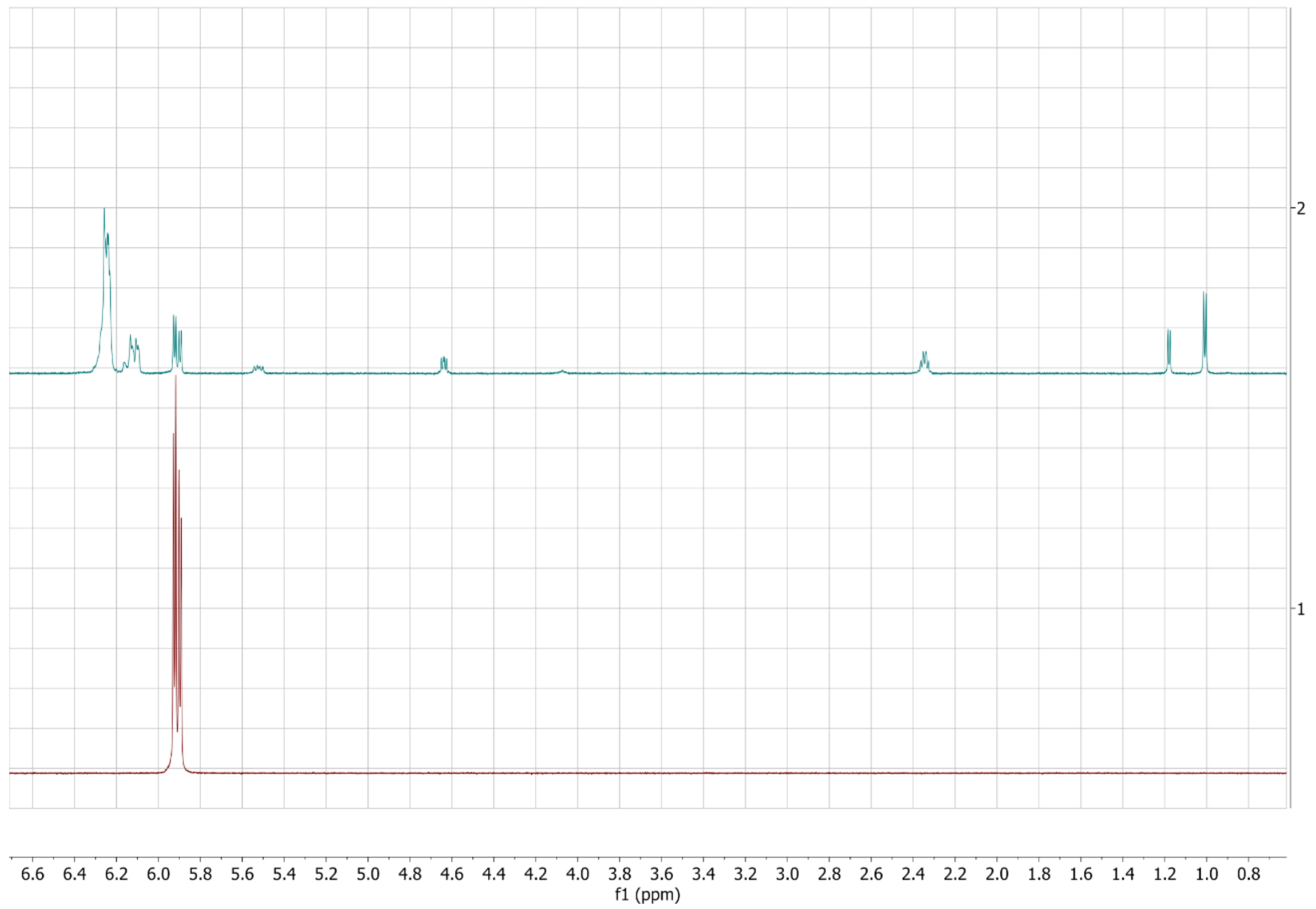

Fig. S22. A series of selective 1D NMR experiments of strevertene A2 (DMSO-d_6, 600 MHz). The spectrum 1 (bottom) is a selective excitation of the signal of proton H25 with the use of the double pulsed field gradient spin echo (dpfgse) selective excitation block. The spectrum 2 is a selective 1D TOCSY spectra obtained with the selective excitation of H25. The spinlock duration within the 1D TOCSY experiment was set to 0.150 s.

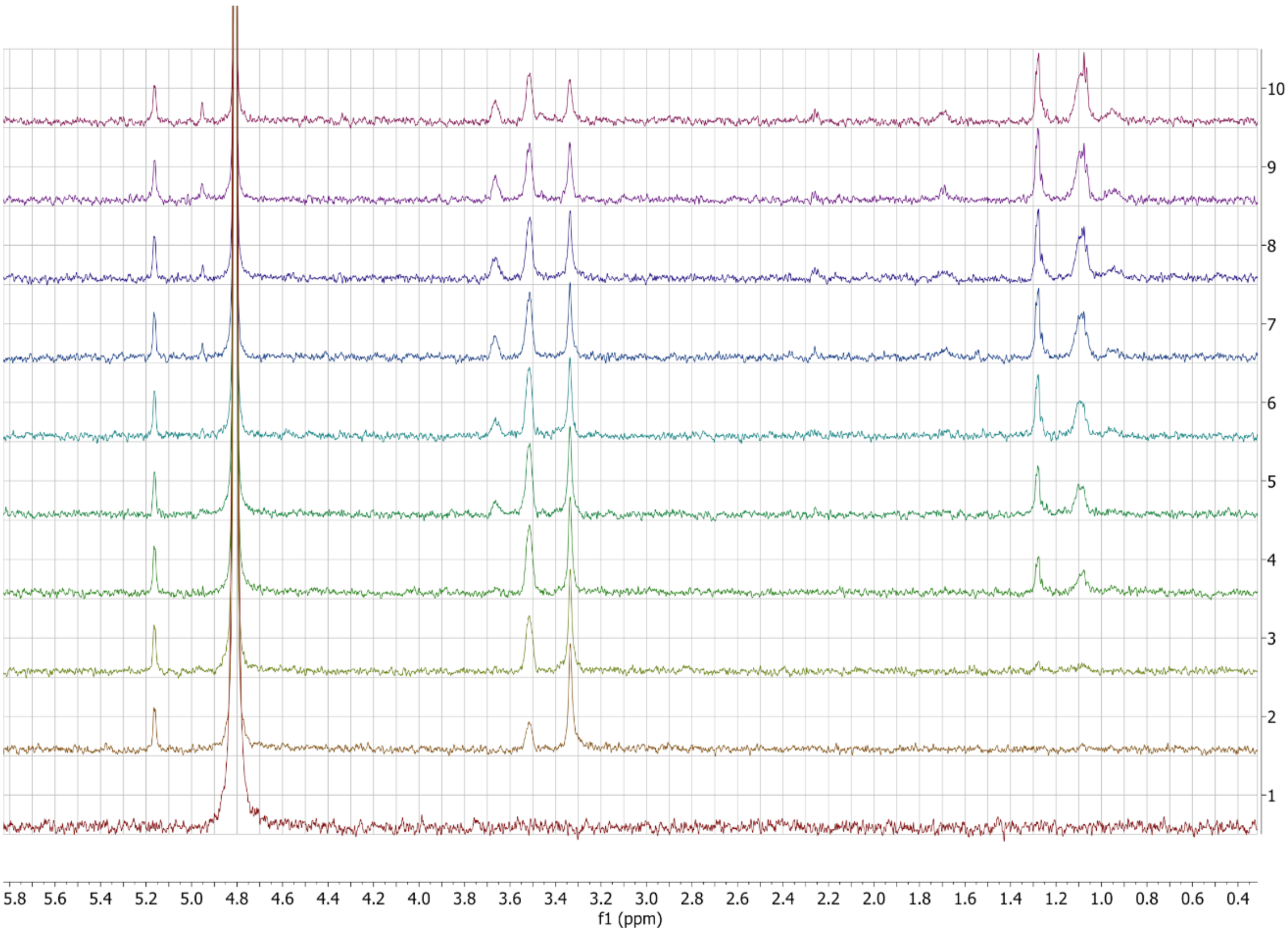

Fig. S23. A series of selective 1D NMR experiments of strevertene A2 (DMSO-d_6, 600 MHz). The spectrum 1 (bottom) is a selective excitation of the signal of proton 5-OH with the use of the double pulsed field gradient spin echo (dpfgse) selective excitation block. The spectra 2 to 10 are selective 1D TOCSY spectra obtained with the selective excitation of 5-OH. With the ascending number of the spectrum, the spinlock durations within the 1D TOCSY experiment were set to: 0.015, 0.030, 0.045, 0.060, 0.075, 0.090, 0.105, 0.120, and 0.150 s.

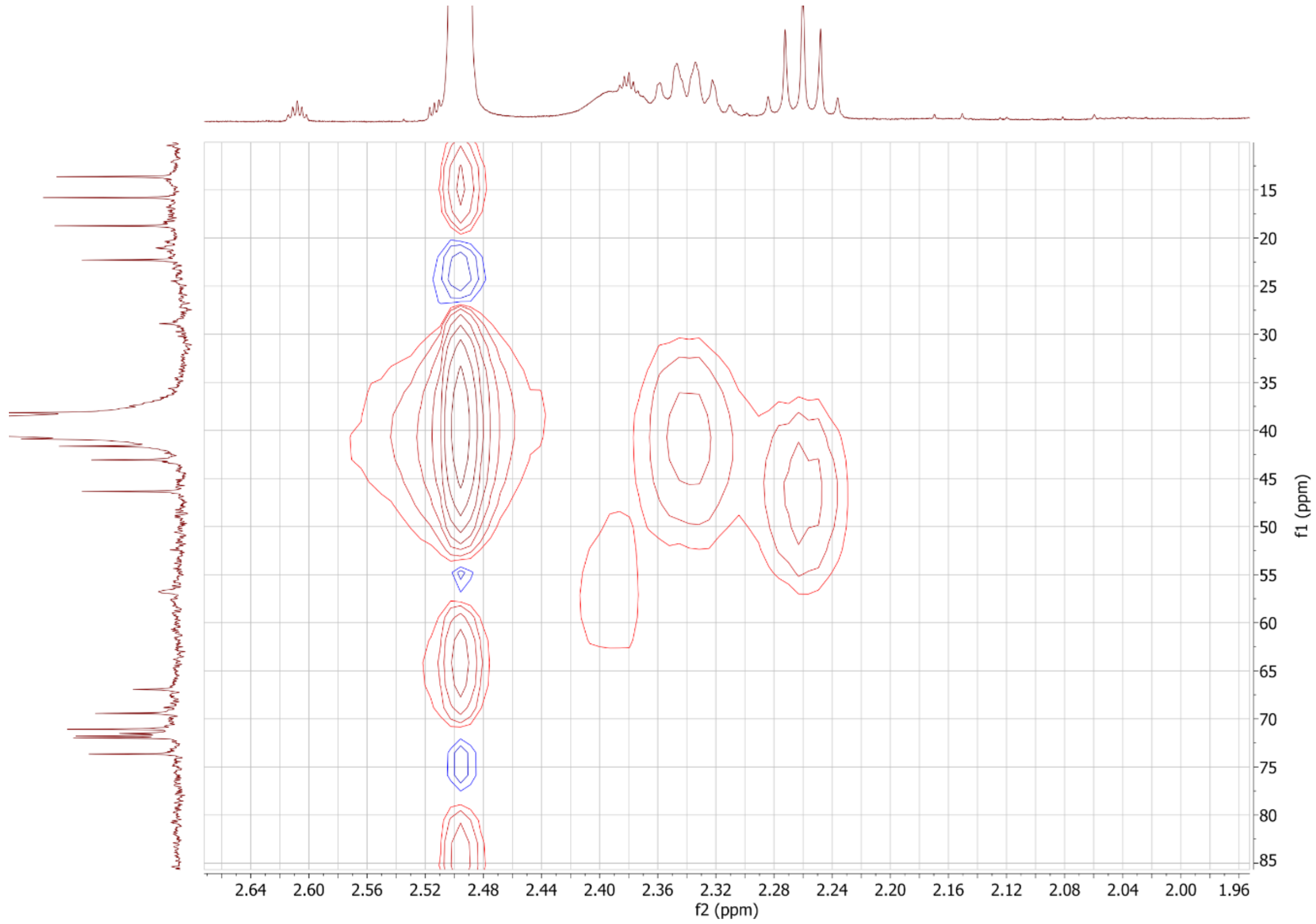

Fig. S24. A selected section from the HSQC NMR spectrum of strevertene A2 measured at 45 °C (DMSO-d_6, 600/151 MHz). The HSQC signal of the pair H14-C14 appeared at frequencies of approximately δ_H = 2.39 ppm and δ_C = 56.7 ppm with the signal polarity corresponding to CH-groups.

Structural elucidation of strevertene X

The standard ^{1}H NMR spectrum of strevertene X showed complex multiplets throughout the entire spectral window (Fig. S25). The ^{13}C NMR spectrum (Fig. S27) revealed 34 dominant signals, suggesting that the sample contained mainly a single compound with minor impurities. Due to these impurities, the structure elucidation was performed stepwise, analyzing local homonuclear and heteronuclear interactions to gradually resolve the macrocyclic core. One of the four doublet-shaped methyl ^{1}H signals (δ_{H} = 1.155 ppm; Fig. S26) was chosen as the starting point. In the HSQC spectrum (Figs. S29 and S30), a correlation peak was observed at this ^{1}H resonance frequency with a f1-coordinate δ_{C} = 13.52 ppm. HMBC correlations (Fig. S34) associated this methyl signal with carbons at δ_{C} = 47.87, 74.11, and 176.23 ppm, the latter being the carbonyl carbon C1. According to HSQC, δ_{C2} = 47.87 ppm correlated with δ_{H2} = 2.337 ppm (Fig. S31), and δ_{C3} = 47.87 ppm correlated with δ_{H3} = 3.872 ppm (Fig. S31); COSY correlations (Fig. S28) and J_{HH} analysis confirmed these assignments. H3 showed further homonuclear correlations, revealing a neighboring methylene group (δ_{H4} = 1.364 and 1.415 ppm, δ_{C4} = 42.55 ppm; Fig. S31). HMBC correlations of H3 with C1, C2, C4, C2a, and δ_{C} = 73.62 ppm were observed. The latter, identified as δ_{C5} (δ_{H5} = 3.659 ppm), corresponded to a CH group with an electronegative substituent; its position was confirmed via COSY interactions with H4. H4 protons, being well-resolved, displayed HMBC correlations with C2, C3, C5, and an unassigned carbon at δ_{C} = 39.60 ppm, assigned to C6. HSQC confirmed C6–H6 as a methylene group (δ_{H} = 1.199 and 1.256 ppm, δ_{C} = 39.60 ppm). Another methylene group with overlapping ^{1}H signals was also observed.

To continue, a proton correlating with both C5 and C6 was sought in the HMBC spectrum. A candidate at δ_{H} = 1.723 ppm showed correlations with δ_{C6} and a merged signal between δ_{C5} = 73.62 ppm and δ_{C} = 73.70 ppm, suggesting it is H7. Selective 1D TOCSY experiments (Figs. S36–S38) supported this assignment. H7 (δ_{H7} = 1.723 and 1.095 ppm) displayed HMBC correlations with δ_{C} = 40.06 ppm (CH_2, δ_{H} = 1.214 and 1.291 ppm) and δ_{C} = 73.70 ppm (deshielded CH, δ_{H} = 3.602 ppm), which were assigned to positions C8 and C9, respectively.

HMBC and COSY correlations further allowed the assignment of the methylene at position 10 (δ_{C10} = 44.65 ppm, δ_{H10} = 1.266 and 1.495 ppm). Well-resolved COSY cross-peaks enabled stepwise identification of positions 11–16 and 14a (Table S5). C17 was assigned from HMBC signals of H15 (δ_{H15} = 3.828 ppm), correlating with δ_{C} = 10.79 (C29), 46.74 (C14), 70.30 (C13), 135.70 (C16), and 133.01 ppm. The unassigned δ_{C} = 133.01 ppm was assigned to C17, and HSQC revealed δ_{H17} = 6.237 ppm, showing strong coupling with H18.

The carbonyl at C1 was further analyzed. In addition to δ_{H2} = 2.337 ppm, δ_{H3} = 3.872 ppm, and δ_{H2a} = 1.155 ppm, a proton at δ_{H27} = 4.754 ppm correlated with C1 (δ_{C1} = 176.23 ppm). HSQC indicated δ_{C27} = 75.67 ppm; the increased chemical shift value and the multiplet pattern (doublet of quartets, J_{HH} = 9.4, 6.3 Hz) suggested that H27 is bonded to one methyl, one CH, and one oxygen, defining C1 as part of a lactone. COSY correlations confirmed the interactions of H27 with H28 (δ_{H} = 1.249 ppm) and H26 (δ_{H} = 2.349 ppm). Corresponding ^{13}C signals were δ_{C31} = 19.11 ppm and δ_{C26} = 42.76 ppm. H26 further correlated with a methyl (δ_{H} = 1.061 ppm, δ_{C26a} = 16.93 ppm) and a sp^2 CH group (δ_{H} = 5.831 ppm, δ_{C25} = 137.77 ppm). COSY allowed the assignment of H24 (δ_{H24} = 6.147 ppm) via its interaction with H25 (δ_{H25} = 5.831 ppm). Stepwise analysis of HSQC and HMBC enabled partial assignments for positions 22–24. A 1D TOCSY experiment with a 150 ms spin lock confirmed the macrocycle, showing polarization transfer from H16 (δ_{H16} = 5.666 ppm; Fig. S35) to H24 (δ_{H24} = 6.147 ppm).

Electronegative substituents of CH groups at positions 3, 5, 9, 11, 13, and 15 were assigned as hydroxy groups, supported by HR-ESI-MS signals of the hexahydroxy derivative. Comparison with literature data (Bortolo et al. 1993) showed excellent agreement, except for minor differences in methylene assignments at positions 4, 8, and 10 (Table S5). The spatial configuration of chiral centers was assumed to match that of strevertene A. Coupling constants J_{H13H14} and J_{H14H15} were similar to those reported for the methyl ester of strevertene A in DMSO-d_6 (Schlingmann et al. 1999).

Table S5. Comparison of experimental and literature NMR data of strevertene X in CD_3OD.

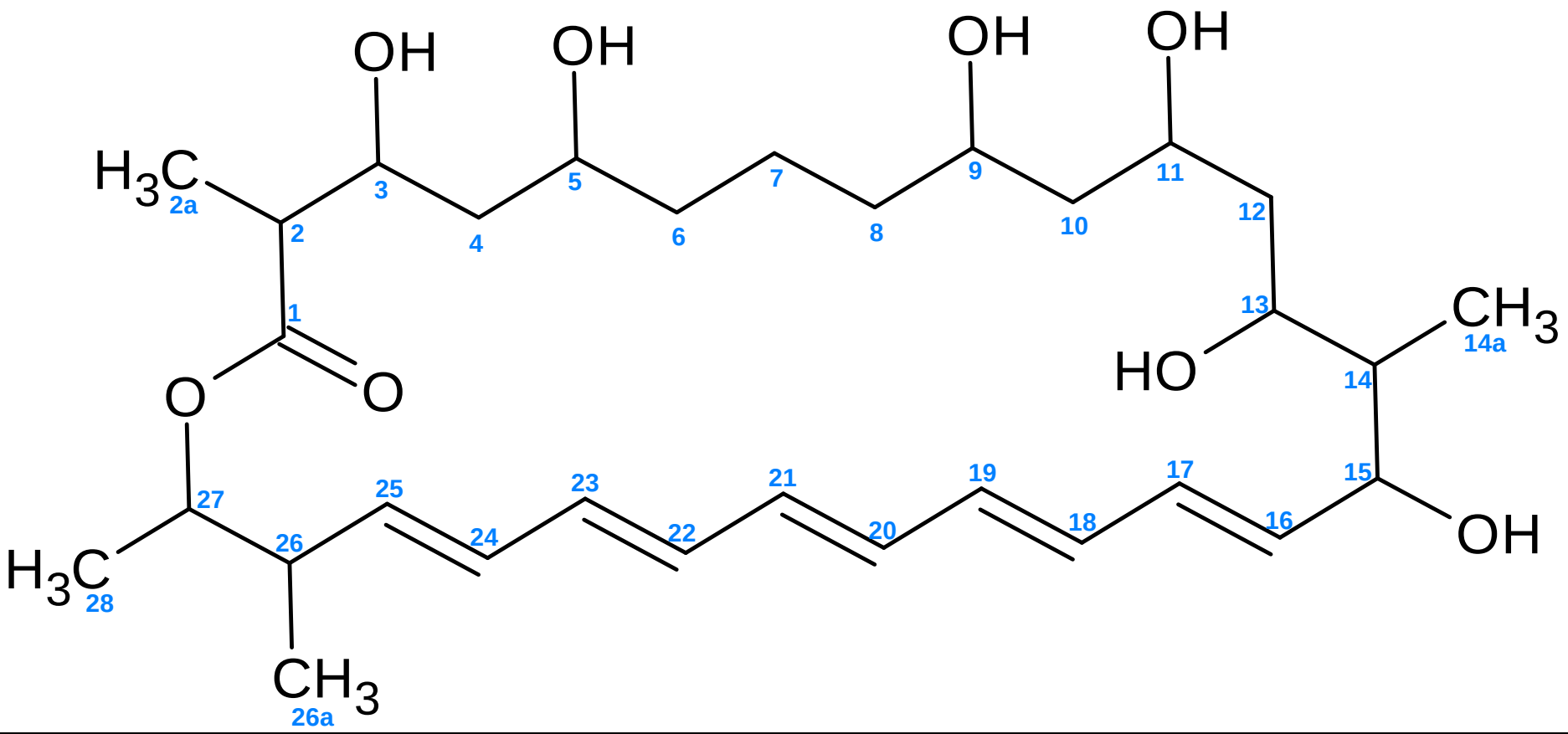

Position	Experimental data			Data of AB023a by (Bortolo et al. (1993)	
	δ_H / ppm	multiplicity; *J* / Hz	δ_C / ppm	δ_H / ppm	δ_C / ppm
1	-	-	176.23	-	176.4
2	2.337	qui; 7.0	47.87	2.34	47.8
3	3.872	ddd; 10.3, 7.0, 2.7	74.11	3.86	74.0
4	1.364	td; 14.2, 10.0	42.55	1.31	44.8
	1.415	ddd; 14.2, 3.2, 2.7		1.44	
5	3.659	m	73.62	3.65	73.5
6	1.199	m	39.60	1.23	39.6
	1.256	m			
7	1.095	m	23.63	1.02	23.5
	1.723	qt; 12.6, 4.3		1.73	
8	1.214	m	40.06	1.40	42.7
	1.291	m			
9	3.602	m	73.70	3.59	73.7
10	1.266	ddd; 14.3, 10.5, 8.8	44.65	1.26	40.0
	1.495	ddd; 14.3, 3.5, 2.5		1.45	
11	3.959	m	71.67	3.95	71.8
12	1.465	m	39.67	1.45	40.0
13	3.669	m	70.30	3.66	70.5
14	1.830	dqd; 8.8, 6.8, 3.6	46.74	1.82	46.9
15	3.828	dd; 8.8, 7.9	76.69	3.82	76.7
16	5.666	dd; 14.5, 7.9	135.70	5.65	135.7
17	6.237	m	133.01	6.22	132.7
18	6.268	m	132.66	6.24	134.7~132.7
19	6.225-6.323	m	134.86 / 134.78 / 133.46		
20					
21					
22			132.75		
23	6.239	m	134.39		
24	6.147	m	131.41	6.15	131.5
25	5.831	dd; 15.4, 7.5	137.77	5.82	137.8
26	2.349	ddq; 9.4, 7.5, 6.8	42.76	2.35	43.1
27	4.754	dq; 9.4, 6.3	75.67	4.74	75.7
28	1.249	d; 6.3	19.11	1.24	19.0
2a	1.155	d; 7.0	13.52	1.15	13.4
14a	1.026	d; 6.8	10.79	1.02	10.9
26a	1.061	d; 6.8	16.93	1.05	17.0

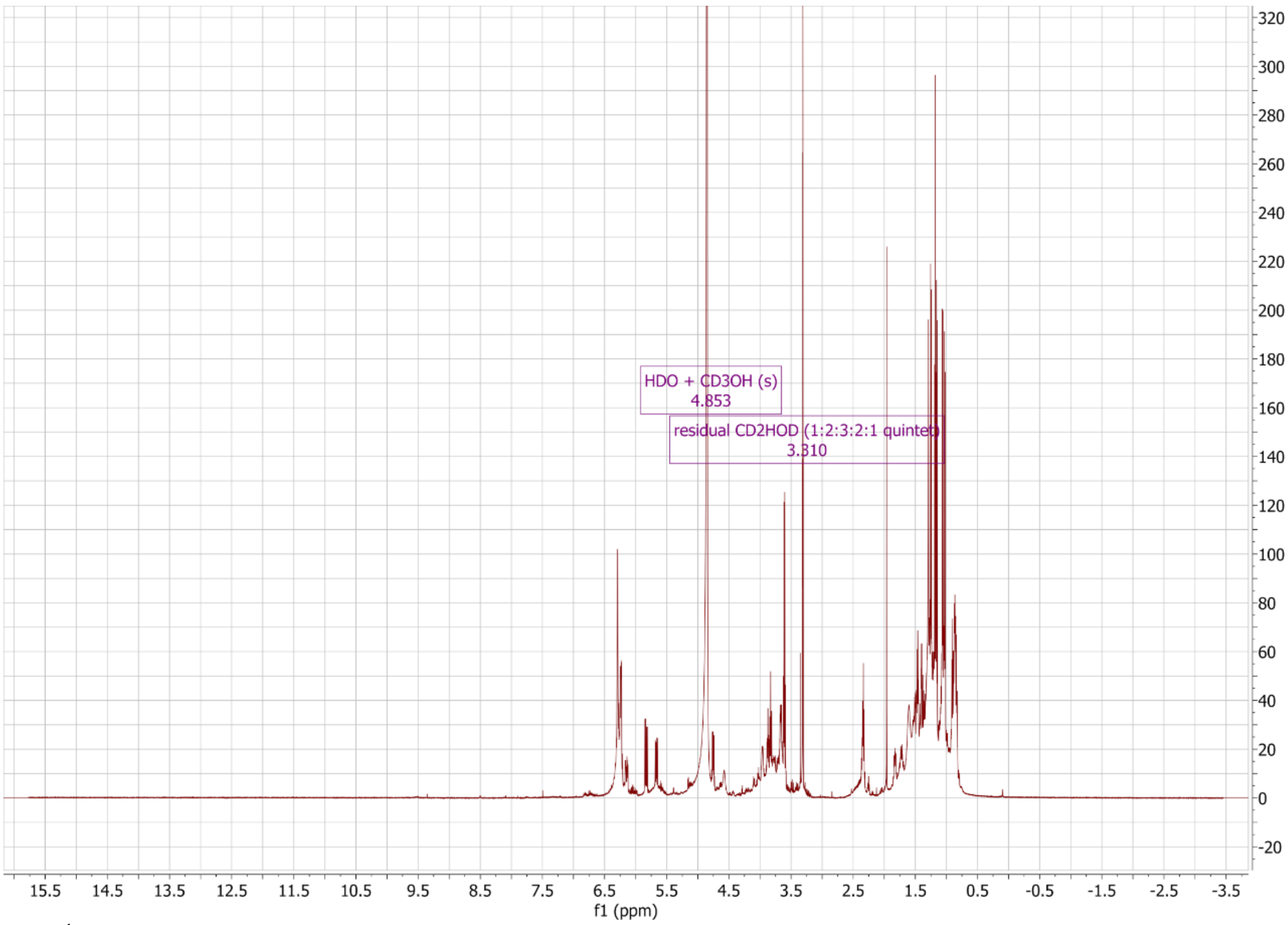

Fig. S25. ^{1}H NMR spectrum of strevertene X (CD_3OD, 600 MHz).

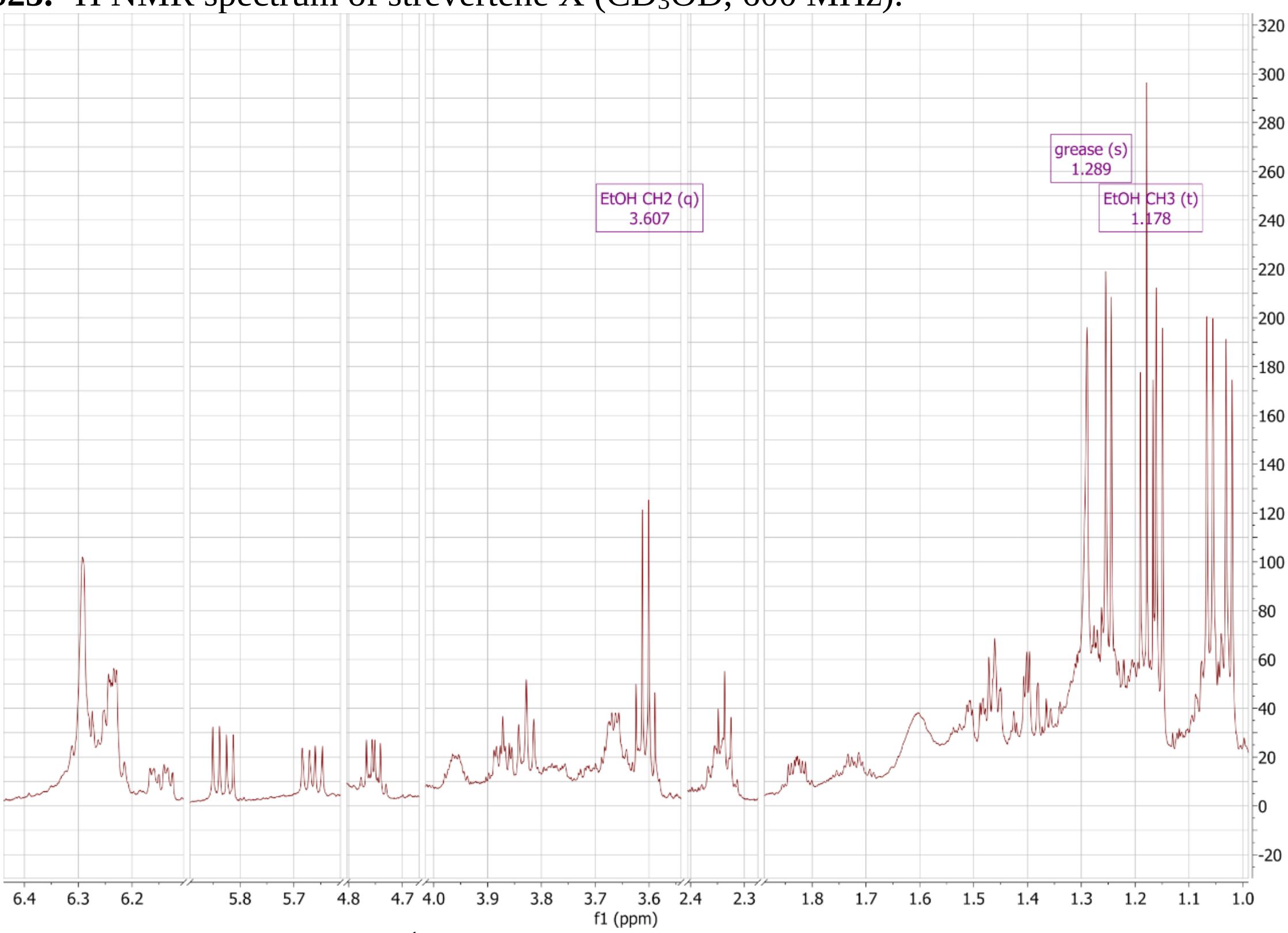

Fig. S26. Selected regions of the ^{1}H NMR spectrum of strevertene X (CD_3OD, 600 MHz). The signals of impurities (ethanol, grease) were annotated.

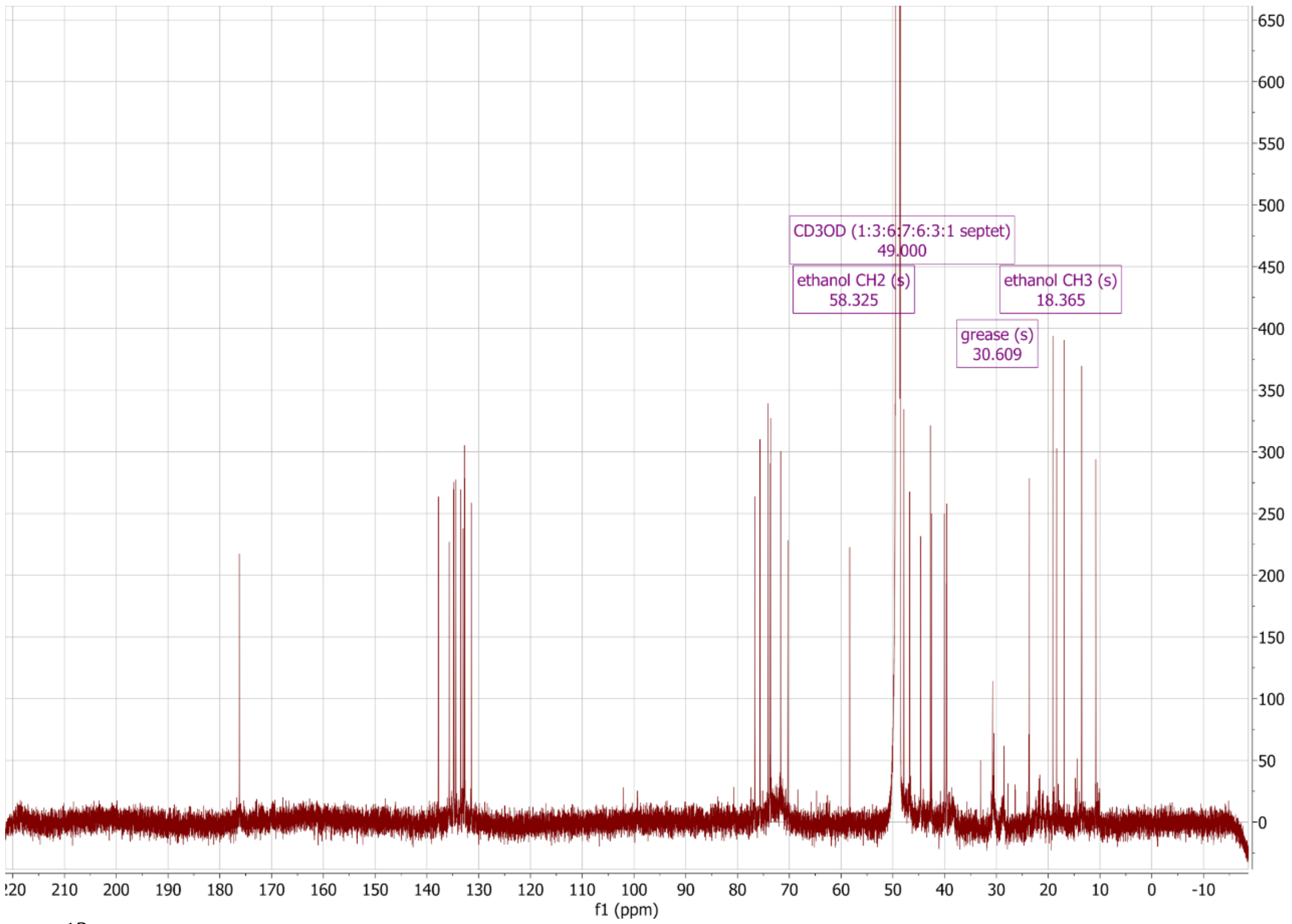

Fig. S27. ^{13}C NMR spectrum of strevertene X (CD_3OD, 151 MHz).

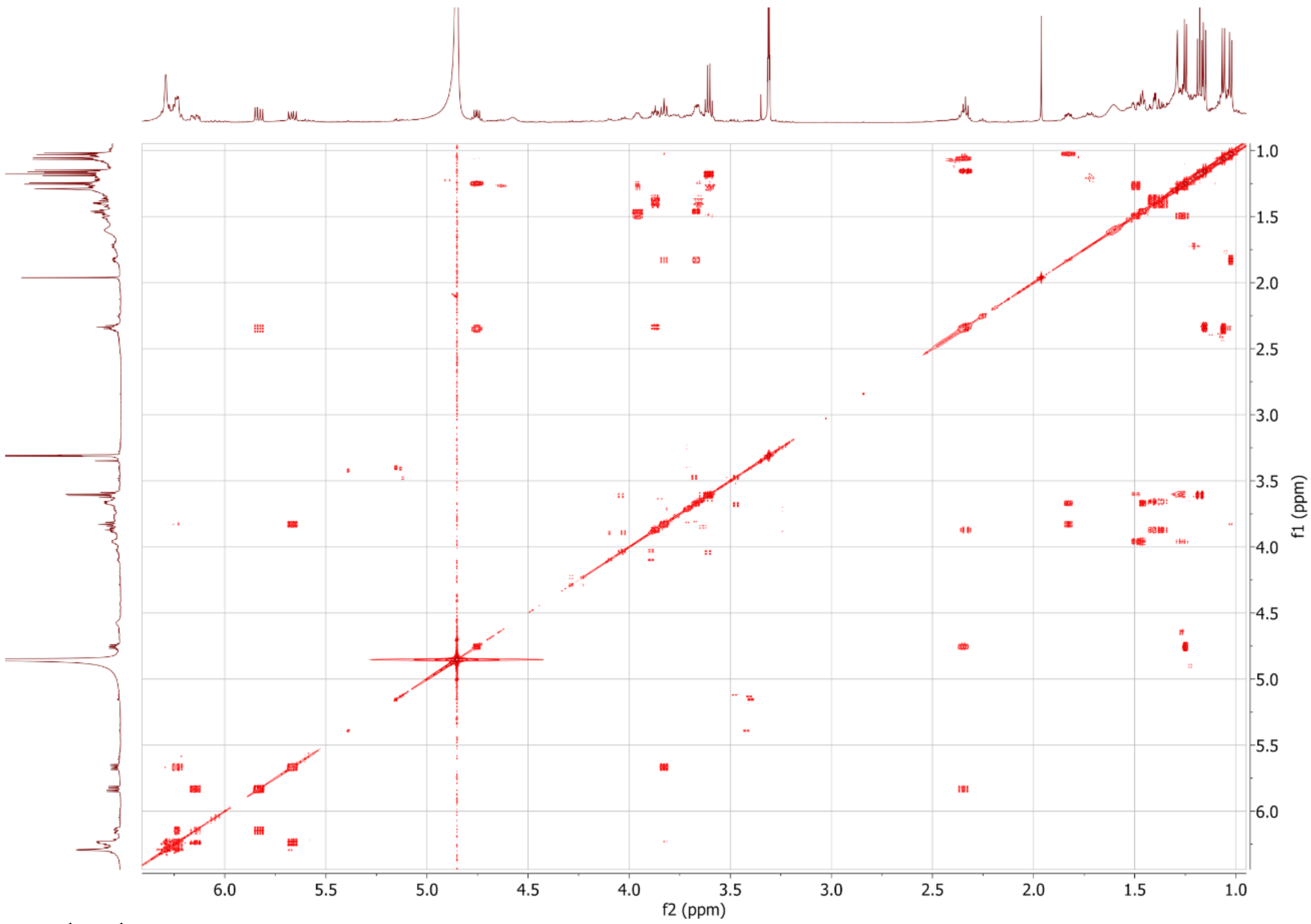

Fig. S28. ^{1}H-^{1}H COSY NMR spectrum of strevertene X (CD_3OD, 600/600 MHz).

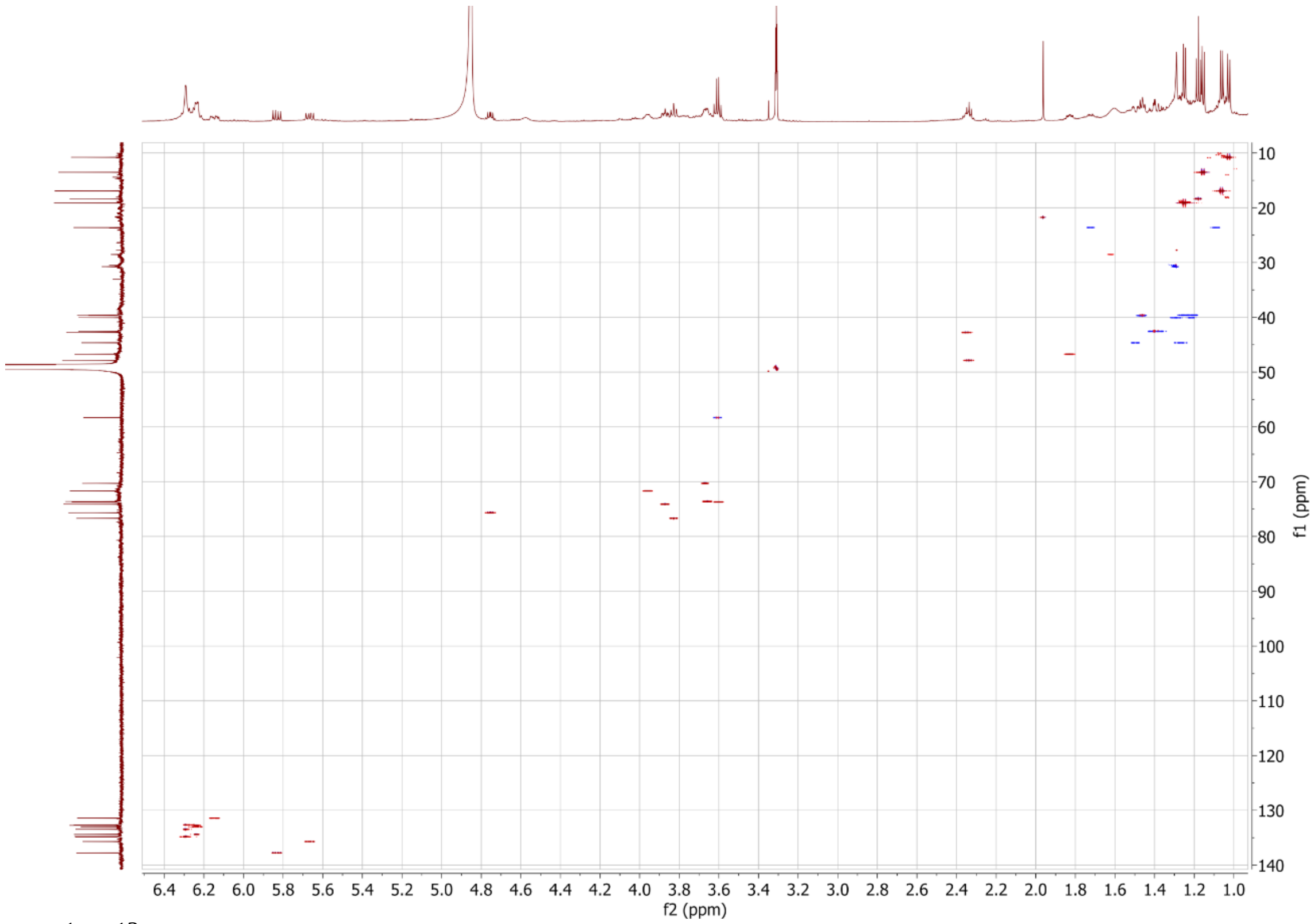

Fig. S29. ^{1}H-^{13}C HSQC NMR spectrum of strevertene X (CD_3OD, 600/151 MHz).

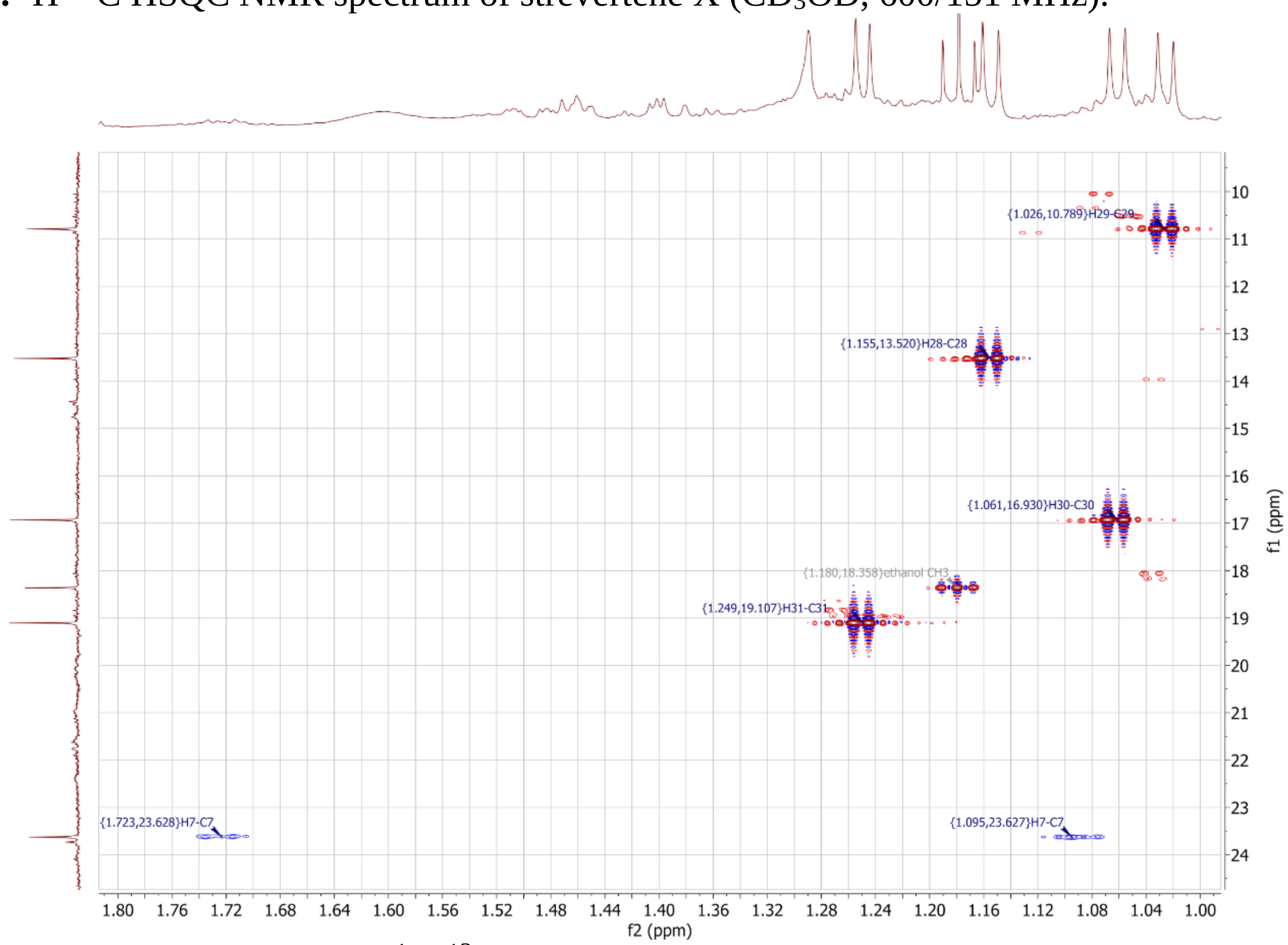

Fig. S30. A selected section of the ^{1}H-^{13}C HSQC NMR spectrum of strevertene X (CD_3OD, 600/151 MHz).

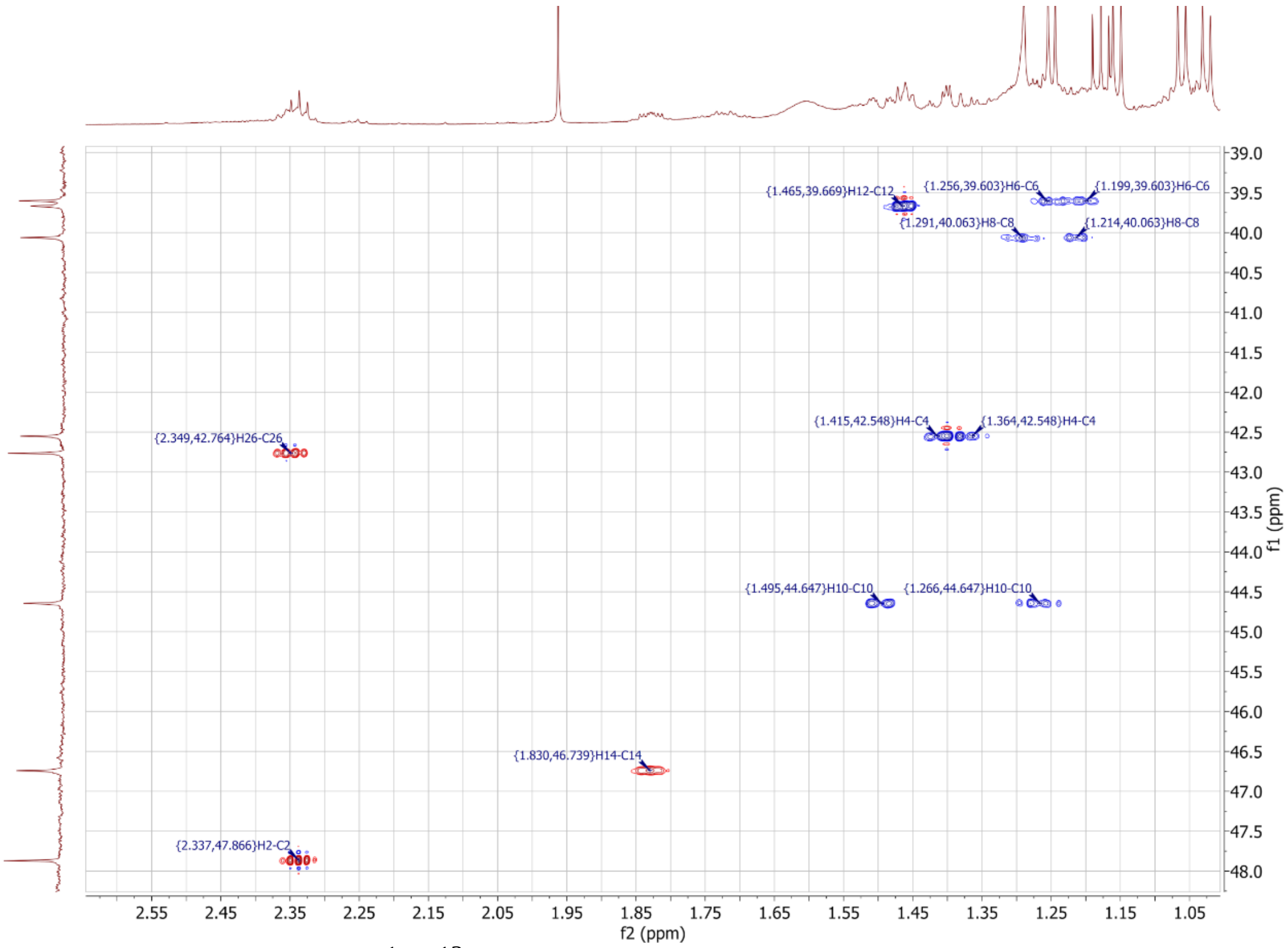

Fig. S31. A selected section of the ^{1}H-^{13}C HSQC NMR spectrum of strevertene X (CD_3OD, 600/151 MHz).

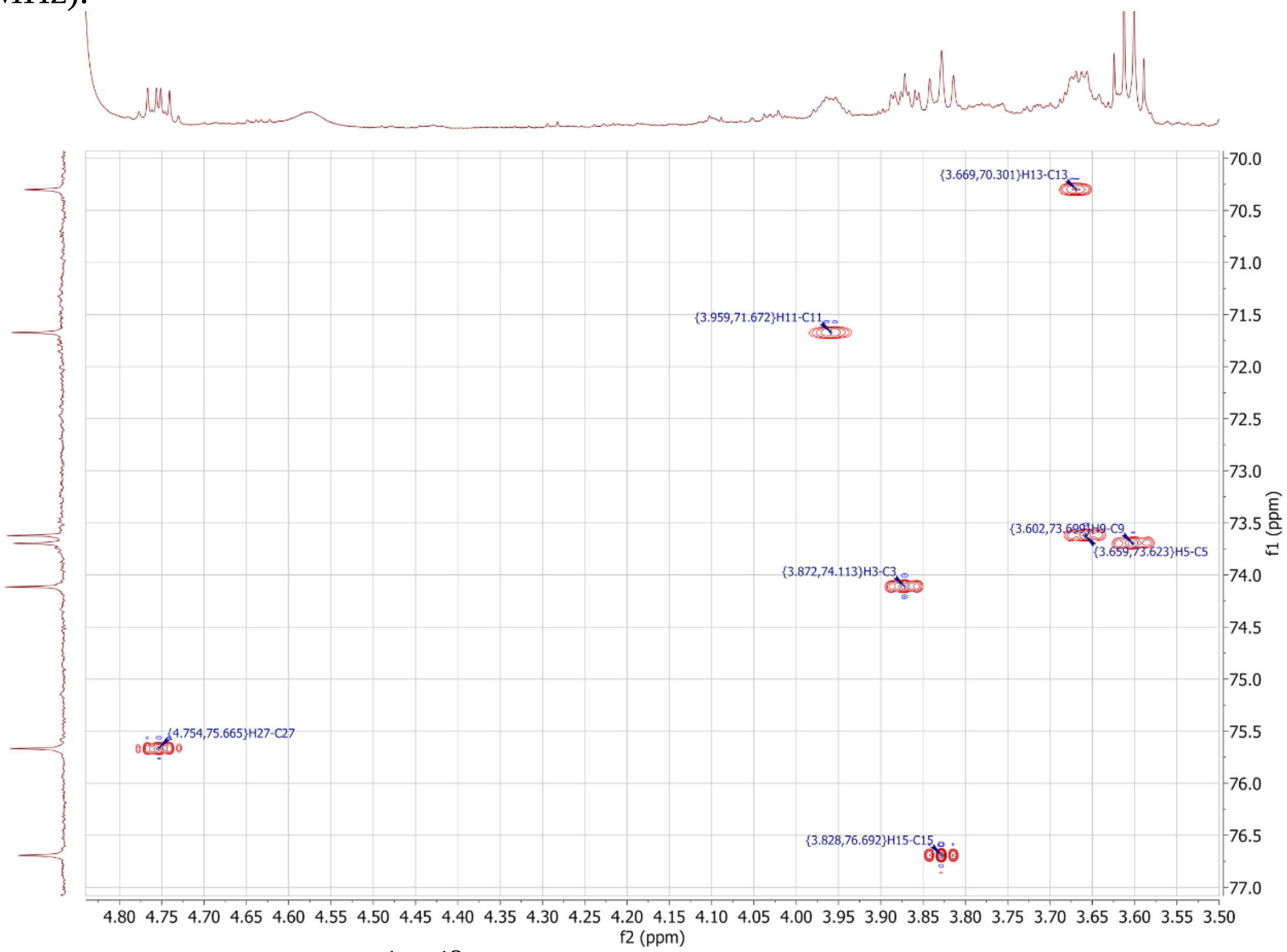

Fig. S32. A selected section of the ^{1}H-^{13}C HSQC NMR spectrum of strevertene X (CD_3OD, 600/151 MHz).

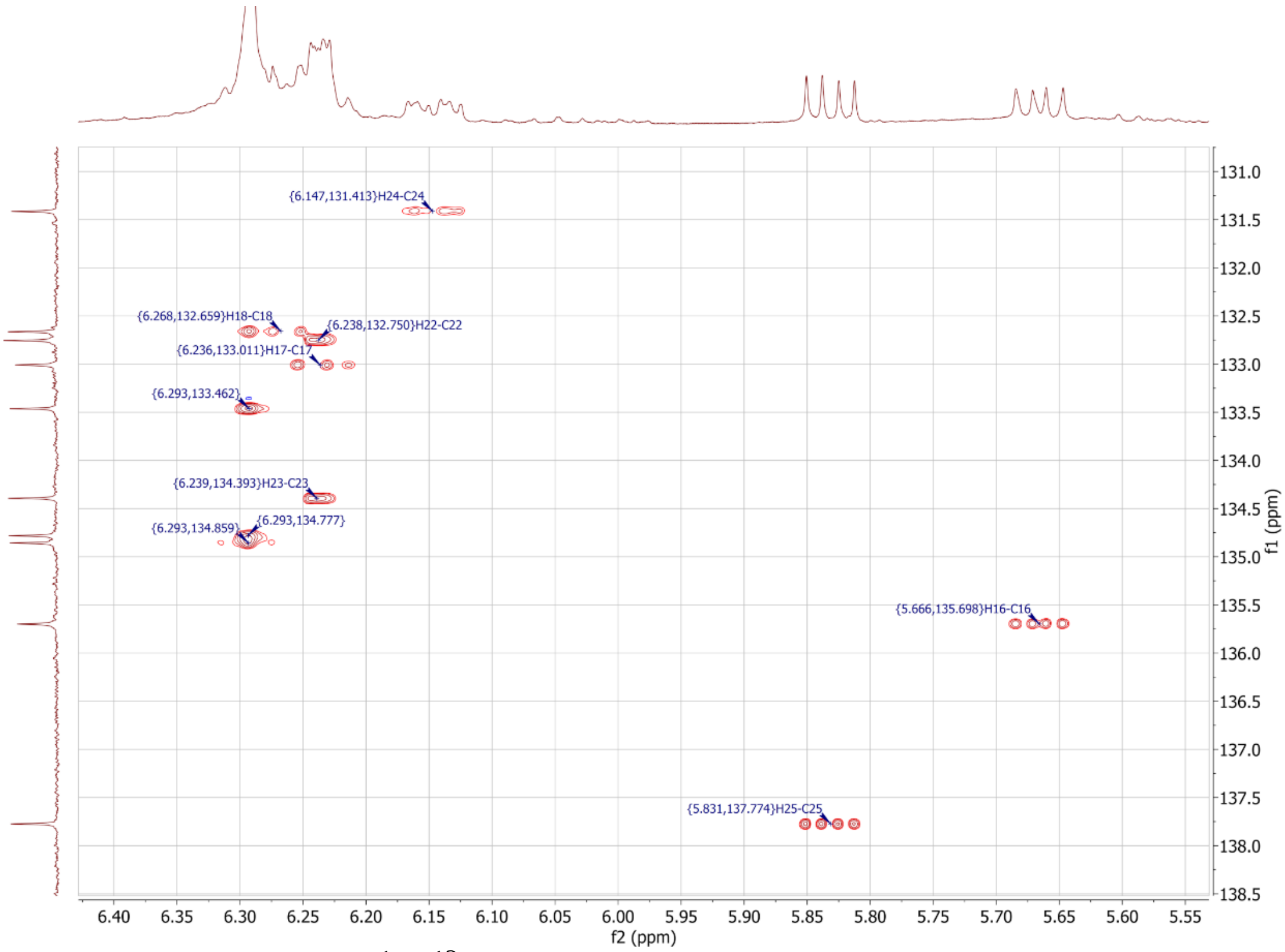

Fig. S33. A selected section of the ^{1}H-^{13}C HSQC NMR spectrum of strevertene X (CD_3OD, 600/151 MHz)..

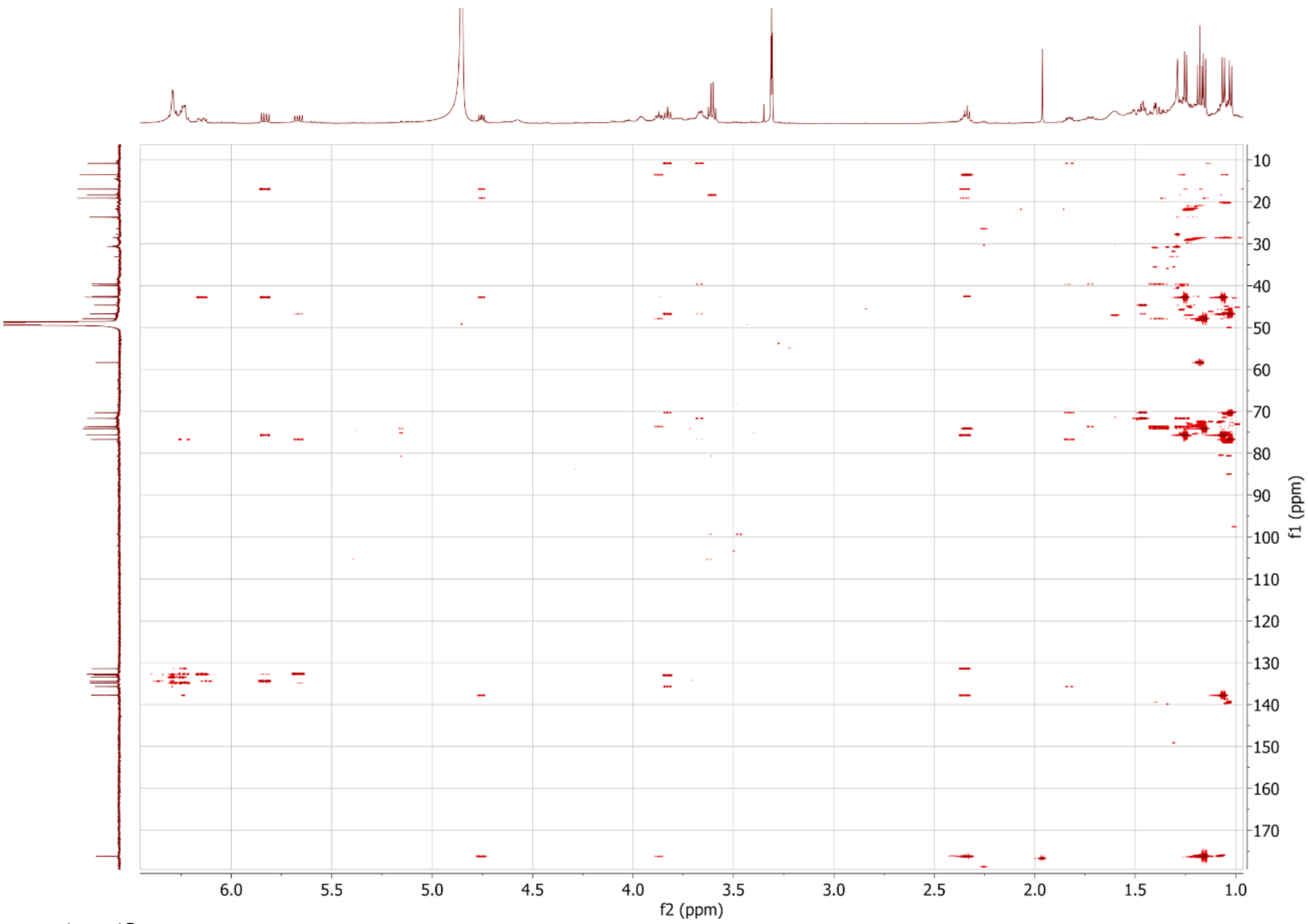

Fig. S34. ^{1}H-^{13}C HMBC NMR spectrum of strevertene X (CD_3OD, 600/151 MHz).

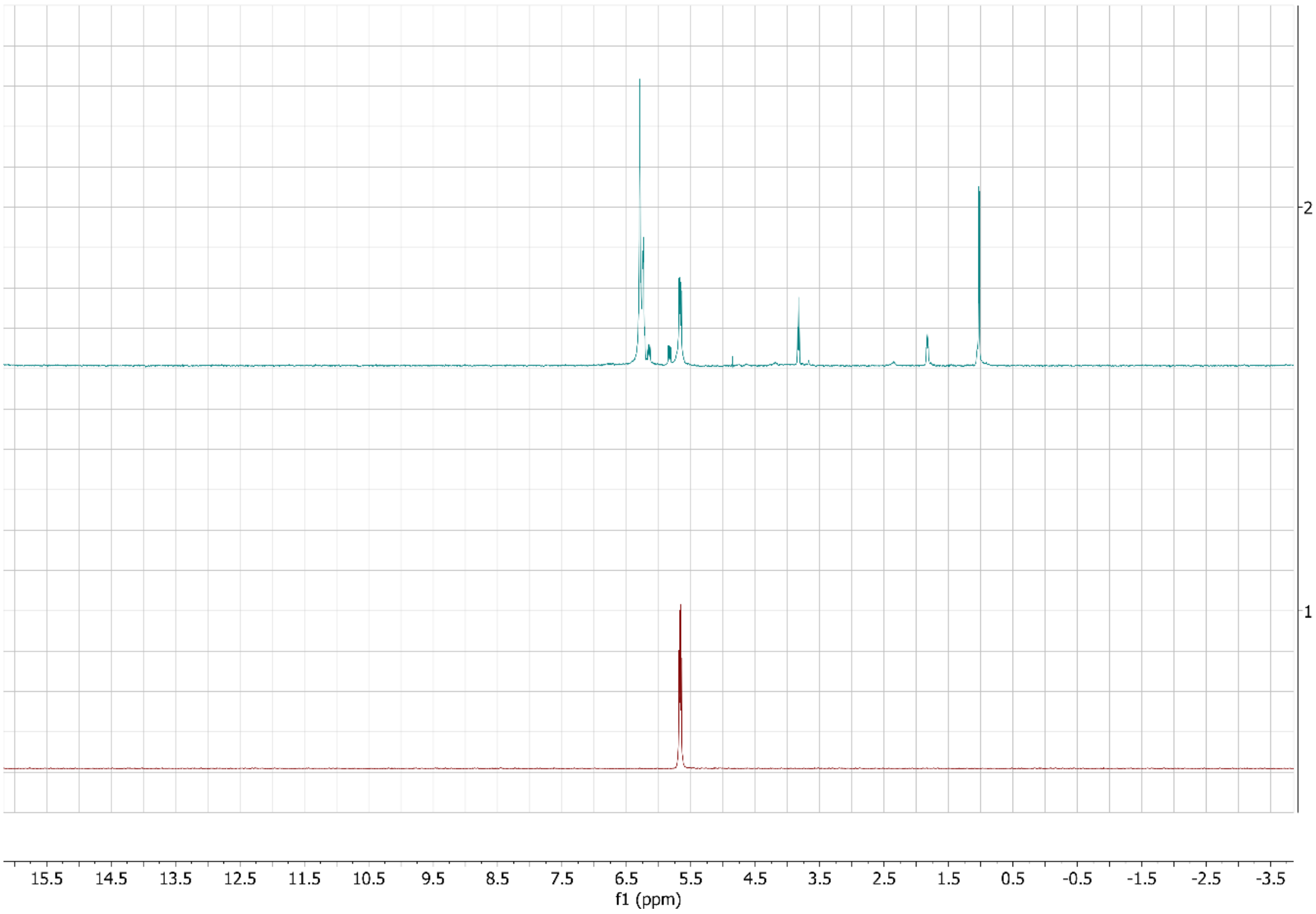

Fig. S35. Selective 1D NMR spectra of strevertene X (CD_3OD, 600 MHz). Bottom (maroon) – selective gradient ^1H NMR excitation of the proton H16 with δ_{H16} = 5.666 ppm. Top (teal) – selective gradient ^1H TOCSY NMR spectrum with the excitation of H16, and with a 150 ms spin lock duration.

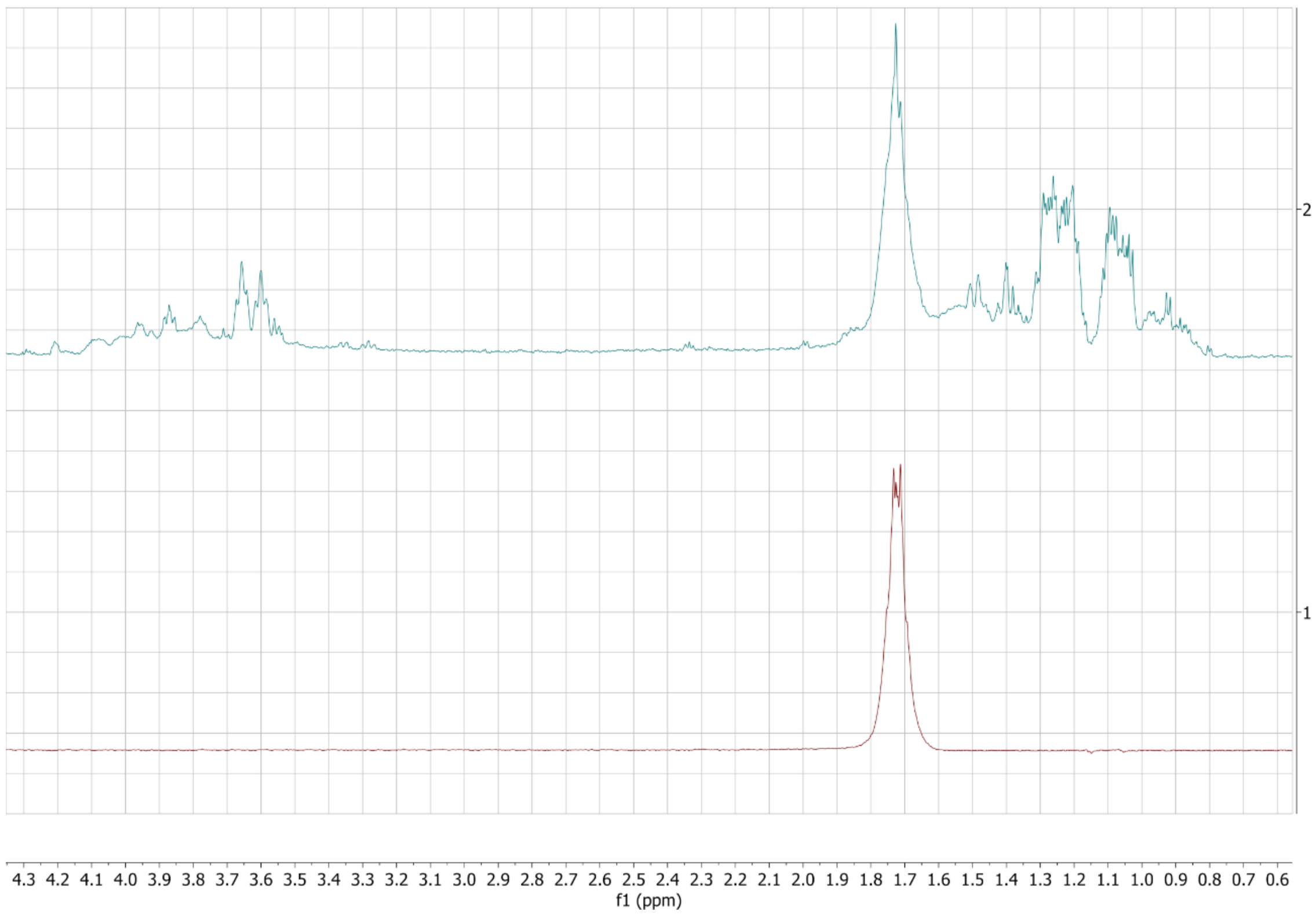

Fig. S36. Selective 1D NMR spectra of strevertene X (CD_3OD, 600 MHz). Bottom (maroon) – selective gradient ^1H NMR excitation of the proton H7 with δ_{H7} = 1.723 ppm. Top (teal) – selective gradient ^1H TOCSY NMR spectrum with the excitation of H7, and with a 90 ms spin lock duration.

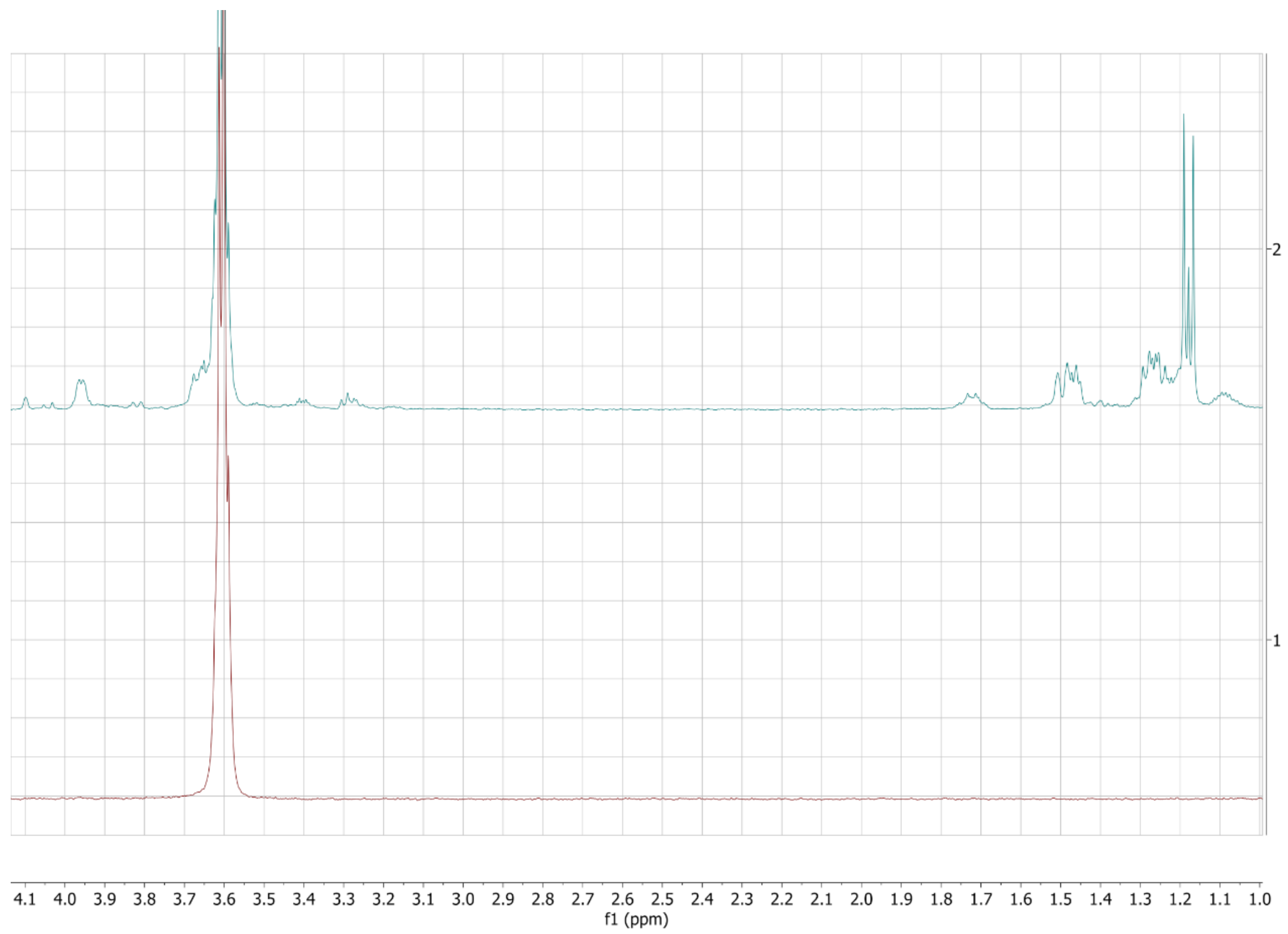

Fig. S37. Selective 1D NMR spectra of strevertene X (CD_3OD, 600 MHz). Bottom (maroon) – selective gradient ^{1}H NMR excitation of the proton H9 with δ_{H9} = 3.602 ppm. Top (teal) – selective gradient ^{1}H TOCSY NMR spectrum with the excitation of H9, and with a 90 ms spin lock duration.

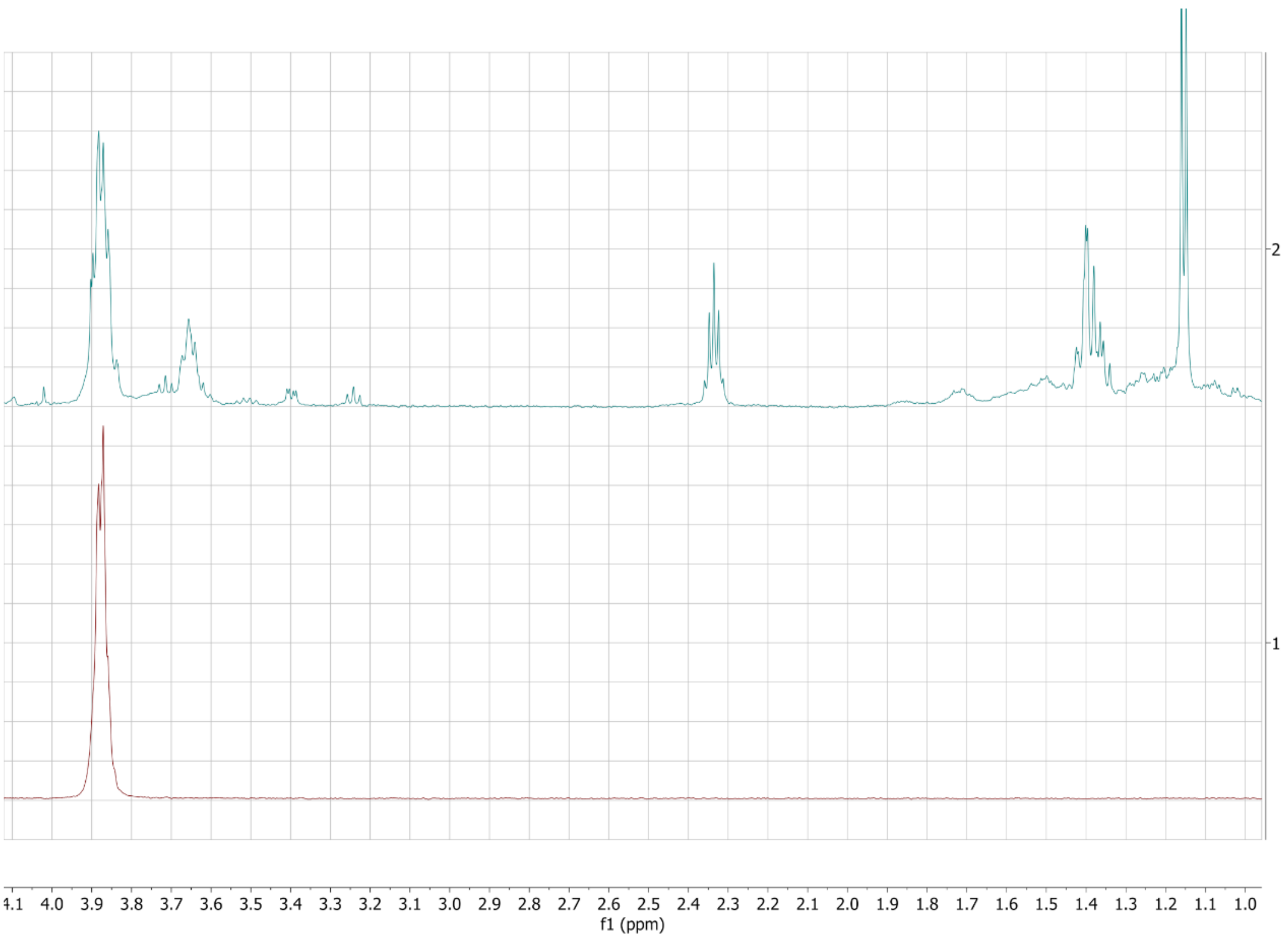

Fig. S38. Selective 1D NMR spectra of strevertene X (CD_3OD, 600 MHz). Bottom (maroon) – selective gradient ^{1}H NMR excitation of the proton H3 with δ_{H3} = 3.872 ppm. Top (teal) – selective gradient ^{1}H TOCSY NMR spectrum with the excitation of H3, and with a 90 ms spin lock duration.

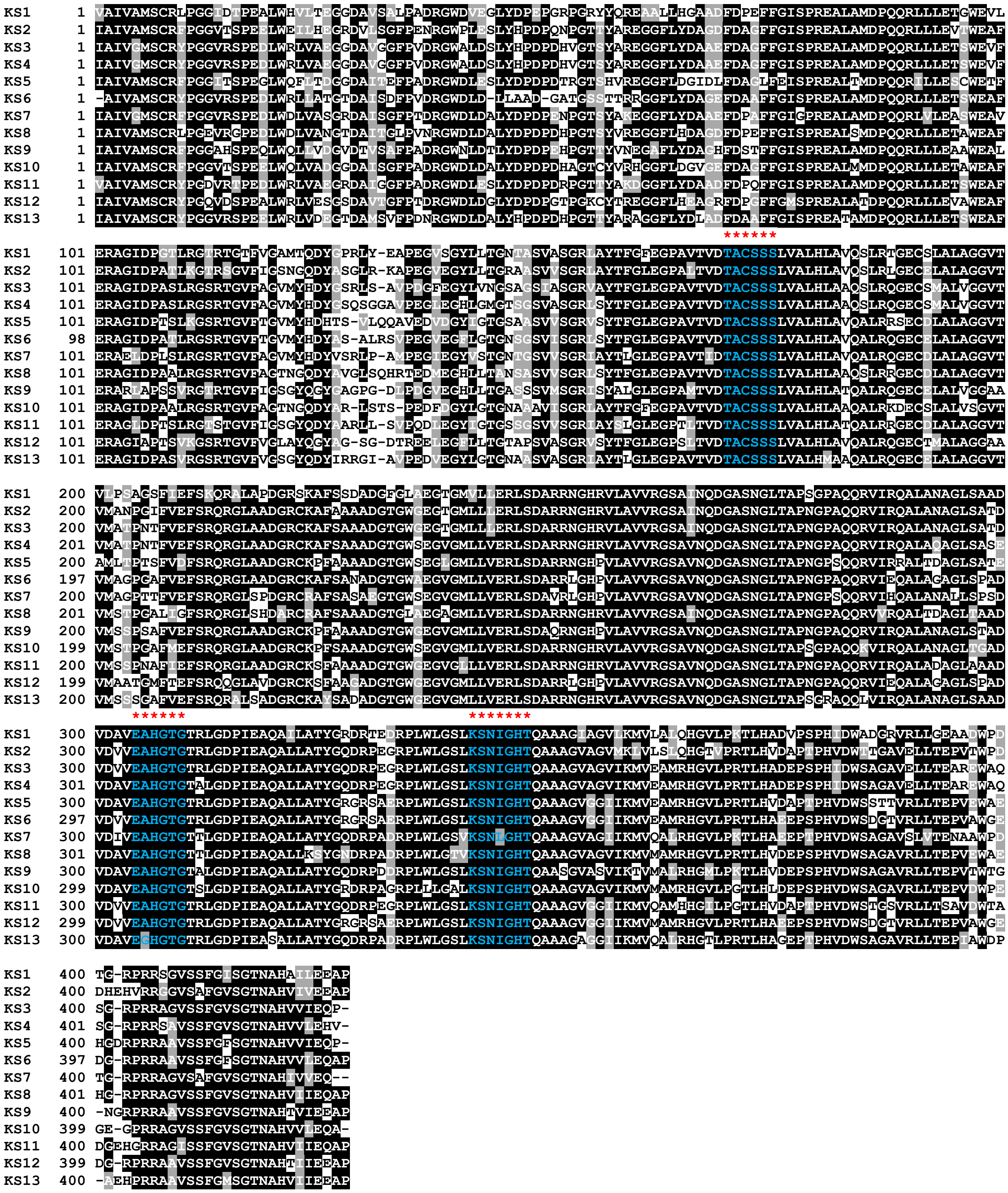

Fig. S39. Amino acid alignment of KS domains from the *stv* BGC. KS domain designations correspond to their location within the modules (Fig. 6). Numbers indicate the amino acid positions starting from the first residue. Identical residues are highlighted in black, and similar residues are shaded. Conserved motifs required for catalytic activity (Keatinge-Clay 2012) are in blue and marked with red asterisks.

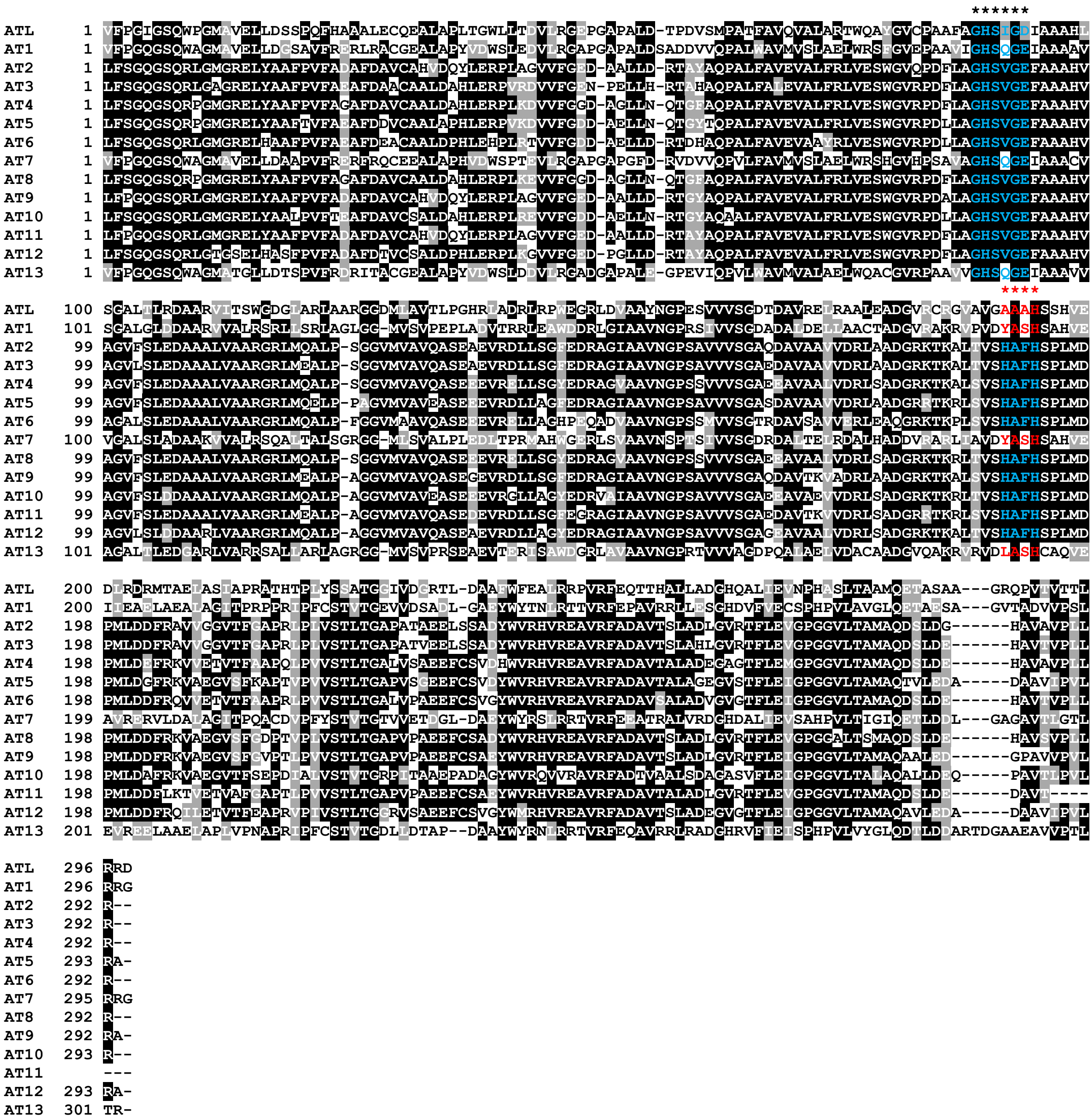

Fig. S40. Amino acid alignment of AT domains from the *stv* BGC. AT domain labels correspond to their location within the modules (Fig. 6). Numbers indicate the amino acid positions from the first residue. Identical residues are highlighted in black, and similar residues are shaded. Conserved motifs essential for AT activity (Yadav et al. 2003) are marked with red asterisks. Residues specific for malonyl-CoA are in blue, and those specific for methylmalonyl-CoA are in red (Yadav et al. 2003).

```
KR1      1 GTVLITGGTGTLGAHVARWLAR-SGAAHLVLTSRSGPAAPGAAELRDELAALGTR-TTIA
KR2      1 GTVLVTGATGTLGRLVARHLVTRHGVRHLVLTGRRGPGAEGAAGFEAELTALGAR-VVTA
KR3      1 GTVLVTGASGVLGRLVARHLVTEHGVRHLLLASRRGPDAEGAPEFAEELGALGAE-VRAV
KR4      1 GTVLVTGASGLLGRHVARHLVTGHGVRDLLLVSRSGALAAGMAELEAELAERGAR-VTVA
KR5      1 GTVLVTGASGALGRVVARHLVTRHGVRRLVLASRRGPAADGAREFAAELGALGAA-VETV
KR6      1 GTVLVTGATGALGRLVARHLVAAHGVRHLVLTSRRGPDADGAGAFAAELAALGAE-VATL
KR7      1 GTALITGGTGALGRLVAAHLVTAHGVRNIVLTGRRGPDADGADALREELAALGAT-VTIA
KR8      1 GTVLITGGTGALGARVARYLAA-RGAEHVVLTGRRGPDAPGAAQIAEEIEALGAE-VTLV
KR9      1 GTVLITGGTGSLGGHTARWLAR-GGAEHLVLTSRRGEAAEGALELAAELGALGAR-TTIA
KR10     1 GTVLITGGTGALGAHVARWLAD-AGAAHLVLTSRRGEAAAGADALRAELEERGAE-VTIE
KR11     1 GTVLVTGATGALGGLVARHLVTRHGARHLLLAGRRGPAAEGAAALRDDLLARGAETVTLA
KR12     1 GTVLITGGTGALGGHVARLLAA-RGAEHLVLTSRRGPAADGAGELRAELEALGAR-VTLA
KR13     1 GTVLVTGGTGFIGSRVAHWLAA-AGAEHLVLTSRRGPAAEGAEELRAELEALGAR-VTLA
                                                         1
                                                        ***
KR1     59 ACDVTDREDLGRLLDSLPGEH----PLTGVIHAAGVLDDGVLDAMTTERLEHVLLPKVDA
KR2     60 ACDVADREALAALLATIPAAH----PLTAVVHAAGVTDDGIVPALTPERVDHVLRPKVDA
KR3     60 RCDVADRTALAGLLAAVPADR----PLTGVVHAAGVTDDGIVSALTPERVDHVFRPKADA
KR4     60 ACDVADRDALARLLAEVPAEH----PLTGVVHVAGVTDDGIVTGLTAERIDRVFRPKVDA
KR5     60 ACDVADREALVRLLDSLPDAH----PLTGVVHAAGVTDDGVVPSLTPERVRRVFDPKVAG
KR6     60 ACDAADRDALAAVLHGIPAAH----PLTAVVHAAGVLDDAPVGSLTARRLDDVLGPKADA
KR7     60 ACDAADREALAALLATLPADR----PLTAVVHAAAVVAGGLVDTLTPEELHQVLRPKVDA
KR8     59 ACDVSDREEVAALLRSVPEDR---HPLTAVVHAAGMPQFAPTDSLDLGDFAEVVSAKVAG
KR9     59 ACDVADRAALAALLDGLADDP---APLTAVVHAAGLPQFSPVADTSLTELAAVVSAKVAG
KR10    59 ACDIADRTAVARLLDGLPGDH----PLTAVVHAAGVGTPGMLAETTPERFADVLAAKAAG
KR11    61 ACDAADRDALAALLAGIPADR----PLTAVVHAAGVLDDGLVATLTGDRLDAVLRPKADA
KR12    59 ACDVSDRDALSRLLTGLTDPAGTDGPLTAVVHTAGVDTTALLADTDPAAFAGVLAAKAAA
KR13    59 ACDMADRTAVSALLESLAGLP----PLSAVVHAAGIGTPALLADTTVAETAEVMGAKAAG
                                                  2     3   4 5 6
                                               *     *     *  *  *
KR1    115 ALHLHELT-----AGLDLDFFVLFSSIAGVVGNGGQGGYAAGNAFLDALAHQRRAQGLPA
KR2    116 ALNLHELT-----RDADLSAFILFSSVSATLGGAGQANYAAANSFLDALAQLRRAAGLPA
KR3    116 ALNLHELT-----RDADLTAFVLFSSAAGTLGSAGQANYAAANAYLDALAETRRAAGLPA
KR4    116 ALHLDELT-----RDMELSAFVLFSSAAGVFGAAGQGNYAAANSFLDALAQQRRAAGLPA
KR5    116 ALHLDELT-----RGLDLSAFVLFSSAAGVFGNAGQGNYAAANAALDALAHTRRAAGLPG
KR6    116 ALHLHELT-----RDADLAAFVLFSSLAGTFGNAGQANYGAANAFLDALAQSRRAAGLPA
KR7    116 ALNLHALT-----RDLDLSAFVLFSSFAGTLGGAGQAAYAAGNAFLDALAHQRRAQGLPA
KR8    116 ASHLDELL-----GDRPLDAFVLFSSVAGVWGSGRQAAYAAANAFLDGLAERRRADGLAA
KR9    116 AVHLDELL-----ADRELDAFVLFSSVSGVWGSGSQAAYSAGNAFLDALAQHRRARGLAG
KR10   115 ARHLDELL-----GDRPLDAFVLFSSISGVWGAAGQAAYAVANASLDALAEHRRSRGLTA
KR11   117 ALNLHELT-----RGLDLSEFVLFSSVAATLGSAGQGNYAAANAFLDALAQRRAAAGLPA
KR12   119 AVHLDELLDALPGGPAELDAFVLFSSIAGVWGAGGQAAYSAANAHLDALAAARRARGLPA
KR13   115 AAHLDALL-----GDRALDAFVLFSSGAAAWGSGAQSAYAAANASLDALAEHRRARGLAA

KR1    170 TAIAWGSWG-
KR2    171 VSMAWGLWAE
KR3    171 VSIAWGLWAE
KR4    171 VSLAWGLWAE
KR5    171 LSLAWGLWA-
KR6    171 VSLAWGPWAE
KR7    171 SSLAWGVWDE
KR8    171 TSVAWGPWAD
KR9    171 TAVAWGPWAE
KR10   170 TAVAWGPWA-
KR11   172 TAIAWGPWAD
KR12   179 TALAWGPWAD
KR13   170 TSIAWGAWG-
```

Fig. S41. Amino acid alignment of KR domains from the *stv* BGC. KR domain designations correspond to their location within the modules (Fig. 6). Numbers indicate the amino acid positions from the first residue. Identical residues are highlighted in black, and similar residues are shaded. Three conserved amino acids residues (Ser, Tyr, Asn) essential for catalytic activity ((Reid et al. 2003) are marked with red asterisks and are in red. Amino acid residues in six regions (1-6) responsible for stereospecificity (Keatinge-Clay 2007) are indicated by red asterisks and corresponding region numbers. Subtypes A1, A2, B1, B2, C1, and C2 (Keatinge-Clay 2007) are indicated by a dstinct colour: A1 in green, B1 in blue. Other invariant specific residues are in yellow.

```
                                                    *   *    *
DH2     1 HPVLGATVLLPDG-AIVLTGRLSLATHPWIADHAVSGVVLVPGTAFVELALRAG--REAG
DH3     1 HALFGAAALLPDGAGSLLTGRLSLATHPWLADHAISGLTLLPGTAFLEMAVRAG--DELD
DH4     1 HPLLGAAVALADGEGALLTGRLSPATHPWLTDHTVMGTVLLPGTALVDLALRAA--EEVG
DH5     1 HALLGAVVTLADGEGVLLTGRVSLATHPWLADHVVLGAALLPGTALVELALRAG--DQVG
DH6     1 HALLGAAVPLADGDGLVLTGRLSPATHPWLADHAVMGAVLLPGTALVDLARRAALDEAVG
DH7     1 HPLLGAVVSLADSDGLLLTGRLAAHTHPWLADHVVLGAVVLPGTAFVELAVRAG--DHVG
DH11    1 HALLGAAVALADGEGVVLTGRLSTAAHPWLAEHQVMGAVLLPGTALVDLAIRAA--DEAG
DH12    1 -VLTAMAQAVLEDADAAVIPVLRADRPEGLAVTTALARLHVHGIPVDWTALLAG----RG

DH2    58 CEQIEELTLEAPLVLPEHGGVQLRLTVGTRDASGRCAIELYSRPED---APGTRDWTRHA
DH3    59 CGRVDELTLAAPLVLPEHGAVQIRVVVGAADEADRRTVRIYSRTDANDEAATELPWTRHA
DH4    59 CDRVDELTLGAPLVLRERAALRLQAVVGGPDATGHRTVNVYSRPES---ADVAEPWTCHA
DH5    59 YGRIEELTLEAPLLLPEQGGAQLQVVVDGPDTNGARAVRVYARHDD---AMEADAWTCHA
DH6    61 CDRLDELTLGAPLVLPDDTAVRIQVRVGSPDGAGRRPIEIHSRTED---APADEPWTRHA
DH7    59 CDRLAELTLQAPLVLPERGGVVVQVAVGPAEGTGERTFTIHSRPDT---AAPEDGWTCHG
DH11   59 CDRVDELTLQAPLVLPSRGGVQLQVTVAGPDDTGRRTVRVHSRPDG---AGPDEPWSTHA
DH12   56 ARRVDLPTYAFQREQYWLETAPAAGGRQSTIEDWQYEVTWQRLPDP-----ATDPATVTA

                                                         **  *** *
DH2   115 AGTLSAGPPAPASS-------ELVAWPPQGAERVATDD---LYDHLDDLGFGYGPVFRGL
DH3   119 TGFLVPDTGAAHAPDVEGPSGTAEAWPPAGSTPVDVHD---CYGRLADAGFAYGPAFQGL
DH4   116 TGVVSVDPRRDAGGGQP---HDLAVWPAPGADRLDTGG---AYERLAGLGFGYGPVFQGL
DH5   116 SGLLSAGTGAAPG---------LAEWPPAGAEPLDAGA---LYDRMAGLGLEYGPVFRGV
DH6   118 AGTLSTAEATADATADA---AALADWPPSEAVAVPEEALDSLYGRMAGLGLDYGPVFRGV
DH7   116 TGVLTSAAEDRPPVPAP------AAWPPAGAAPVDLGG---FYQELAARSFAYGPAFQGL
DH11  116 VGVLATHAPEAAAPAREDPAADLSQWPPAAARPVPTEG---YYERLAELGFGYGPVFQGL
DH12  111 DGHWLLVLP--------------AGTDHDGATERDAAG---LRDALAAHGCAVTSLTVDA

                                            *   *
DH2   165 TEAWRHG-DTLYAAAALPEESAPDAG--AYGLHPALLDAGLHASWLGLLS---GSETGN-
DH3   176 RAVWRRGEDELFAEVGLPDGTSVDG----FAVHPALLDAALHAMVVGD-----VPAIGHE
DH4   170 HALWRRG-DEVFAEVRLPDGTETGG----FGIHPALLDSALHAIGLGG----LLPDAGH-
DH5   164 RAGWRRG-EELFAEIALPDGTDVTG----FGLHPALEDAALHTVGLGD----PAAPADG-
DH6   175 RAAWTRE-DALFAEVELPEGTDVSG----FGLHPALLDAALHTVALDPGLLGPDADAGDR
DH7   167 RAAWRLG-DEVFAEAVLPPDASAPSDAARYGLHPALLDAALHGVGFG-----PLGDMGT-
DH11  173 RALWQRD-GEVFAEVALPQDTTADG----FGLHPALLDSALHAVGLGG----LLPDTGQ-
DH12  154 AEDRETLTARLRGATDATPPRAVVS---LLGLDERPVEGLAALTGGVAATLTLLQALGDA

            *** *
DH2   218 GLLPFSWSAVHLH----AAGATAVRIKLAAA-------GTDAVSVLVADGTGRPLATIGA
DH3   227 GLLPFAWSGVSVH----GTGAETLRVRLTRADS-----AADSVSLAITDGLGR-------
DH4   220 GRIPFAWSGVTVH----ATGATALRVRITPA-------GADAVALLATDTAGRPVASVDS
DH5   214 PRIPFAWSGVTLH----ATGATALRLRLAPAPSSATAAGEGAVSLSLADATGSAVLSVDS
DH6   230 ARIPFSWSGVTAHPAPSAGAVEALRIRLTPA-------GPDTVSLLAA------------
DH7   220 GRMAFSWEDVRLY----ATGATRLRIRLTPV-------GAEAVAVTATDDSGRAVATVAA
DH11  223 GRIPFAWSGVRLD----ATGATGLRVRLTSP-------APDTVSLLVADATGRPVASVE-
DH12  211 GVGAPLWCATRGA---VSTSPDDHPVTAPGQAQ---IWGLGRVAALEHPGRWGGILDLPP
```

Fig. S42. Amino acid alignment of DH domains from the *stv* BGC. DH domain designations correspond to their location within the modules (Fig. 6). Numbers indicate amino acid positions from the first residue. Identical residues are highlighted in black, and similar residues are shaded. Conserved residues within the four conserved motifs required for the catalytic activity of a functional DH domain (Keatinge-Clay 2008) are indicated by asterisks. Amino acid differencies in the DH7 domain compared to the conserved residues are highlighted in yellow and red.

```
ER11      1 GTLDDLALTENPAATAPLGRGQVRIAVRAAGVNFRDALIALGMYPGAATLGSEAAGVVAE
EryER4    1 GAIDSVAFEPAPDVEQPLRAGEVRVDVRATGVNFRDVLLALGMYPQKADMGTEAAGVVTA

ER11     61 TGPGVTGLAVGDRVFGMVPEAFGPLAVADHRMVARIPQGWTFTEAASVPIVFLTAYYALV
EryER4   61 VGPDVDAFAPGDRVLGLFQGAFAPIAVTDHRLLARVPDGWSDADAAAVPIAYTTAHYALH

                       * *   ****  *   *
ER11    121 DLAGLKAGQSLLVHSAAGGVGMAATQLARHLGAEVYGTAGPGKWAALRADGLDEEHLASS
EryER4  121 DLAGLRAGQSVLIHAAAGGVGMAAVALARRAGAEVLATAGPAKHGTLRALGLDDEHIASS

ER11    181 RDLGFEQRFLAATGGRGVDVVLNSLAGAYVDASLRTLTDGGRFLEMGKTDVRDPERTAAE
EryER4  181 RETGFARKFRERTGGRGVDVVLNSLTGELLDESADLLAEDGVFVEMGKTDLRD----AGD

ER11    241 HRGAAYTAFDTIEAGPERIGAMLRELVALFETGALRPLPVTCWDVRRAPDALRHLSQARH
EryER4  237 FRG-RYAPFDLGEAGDDRLGEILREVVGLLGAGELDRLPVSAWELGSAPAALQHMSRGRH

ER11    301 VGKLVL
EryER4  296 VGKLVL
```

Fig. S43. Comparison of ER11 domain from the *stv* BGC with the EryER4 domain from the erythromycin PKS. Numbers indicate amino acid positions from the first residue. Identical residues are highlighted in black, and similar residues are shaded. Conserved residues within the conserved motif LxHxxxGGVGxxAxxxA essential for ER domain function (Kakavas et al. 1997) are marked by red asterisks and in blue.

```
StvA      1 MS---------QGAPVIFSTVRDGYFNPPPMTQSLRRTEPIARMEFADGHLGWLVTGYSA
NysN      1 MSTEADARTAAPQCPVAFPLRRPGRPFPPPEYATYRGGAGLVRSELP-SGPVWLVTRHED
AmphN     1 MTAETEMTTFAPGCPVAFPLRRPGRPFPPPEYADYRAGEGLVRSELPASGPVWLVTRHED
FscP      1 MT-TSPGP-----TVVDFPRRTPREPLPLSQYAEHRKQNGLVQTHLPNGRPIWLVTRHED
PimG      1 MTYTDPAAPETDPPAVDFPQRKPGVPFPPPDYADYRDRKGLVLSQLSDGKRVWLVTRHED
ScnG      1 MTHTDPAL----PPTVDFPLRKPGVPFPPPDYADYRGRKGLVLSHLSDGKRVWLVTRHED

StvA     52 ARAVLADNRFSARGELKHPPVPRAATLEEAPAAP--PGMFIQHDAPEHQRYRKLLVGQFT
NysN     60 VRAVLTDPRISA--DPSKPGFPKAGRTGGAPSQYEVPGWFVAMDPPEHGRFRKTLIPEFT
AmphN    61 VRTVLTDPRISA--DPSRPGFPRARRTGGAPSQSEIPGWFVALDPPEHDRFRKTLIPEFT
FscP     55 VRAVLTHPRISA--NPDNEGFPNVGETMGVPKQEQIPGWFVGLDSPEHDRFRKVLIPEFT
PimG     61 VRAVLTSPSISS--NPEHKGFPNVG-NLGVPKQDQIPGWFVGMDSPEHDRFRKALIPEFT
ScnG     57 VRAVLTSPSISS--NPEHKGFPNVGTTLGVPKQDQIPGWFVGMDSPEHDRFRKALIPEFT

StvA    110 VRRMRMLTEWIEGIVAERLDAMERLGSEADLFEHFALPIPSLVICELLGVPYADRAGFQH
NysN    118 VRKVRELRPVIQQIVDERIDAMLAAGTSADLVESFALPVPSLVISSLLGVPKVDRDFFED
AmphN   119 VRKVRELRPAIQQIVDERIDALLAAGNSADLIADFALSVPSLVISDLLGVPKADRDFFEA
FscP    113 VRRVRELRPAIERTVDERIDAMLAGGNTADLVNDFALPVPSLVISALLGVPSADRDFFES
PimG    118 VRRVRAMKPAIERTVDAQLDAMLAAGNTADLVADFALPIPSLVISALLGVPPADREFFES
ScnG    115 VRRVRAMKPAIERTVDAQLDAMLAAGNTADLVADFALPIPSLVISALLGVPPADREFFES

StvA    170 DTHVWSSFGESTEVVT-AAFLSLGTYLQGLVRLKRAEPTDDILSGLIAADPDLTDEELTS
NysN    178 RTRVLVRL-SSTDEERDKATQALLRYIGRLIQIKQRRPGDDLISRLIAAG-TLSRQELSG
AmphN   179 KTKVLVTL-SSTDEQRDEASKALLRYINRLIQIKGRRPGEDLISRLLQAG-TMNRQELSG
FscP    173 RTRTLVAIRTSTDEERAEATRQLLRYINRLIVIKKKWRGEDLISRLLSTG-KLSDEELSG
PimG    178 RTRVLVSLRSSTDDDRMAAAKDLLRYINRLVEIKQKWGGDDLITRLLATG-AIAPHEMSG
ScnG    175 RTRVLVSLRSSTDDDRMAAAKDLLRYINRLVEIKQKWGGDDLITRLLATG-AIAPHEMSG

StvA    229 IAFLLLVAGHETTANQLALGTFALLRHPEQLAALRADPSLIEGAVEESLRYLSIVHHGPT
NysN    236 VAMLLLIAGHETTANNIGLGVVQLLTNPRWIGDDR--------IVEELLRYYSVADLVAF
AmphN   237 VSMLLLIAGHETTANNIGLGVVQLLTNPQWIGDDR--------IVEEMLRYYSVADLVSF
FscP    232 VLLLLLIAGHETTANNIGLGVVTLLSHREWIGDDR--------LVEELLRLHSVADMVAL
PimG    237 VLMLLLIAGHETTANNIALGVVTLLANPQWIGDDR--------AVEETLRFHSVADLVSL
ScnG    234 VLMLLLIAGHETTANNIALGVVTLLTNPQWIGDDR--------AVEETLRFHSVADLVSL

StvA    289 RAALEDVEIDGVLIKEGEVVMVSLAAANRDPERFPDPEAFDVSRRAGGHLGFGHGIHQCL
NysN    288 RVAVEDVEIGGQLIRAGEGIVPLIAAANHDATAFAAPSEFDPERSARSHVAFGYGVHQCL
AmphN   289 RVAVEDVEIGGQLIKAGEGIVPLIAAANHDGSVFDKPEEFNPERSARSHVAFGYGVHQCL
FscP    284 RVAVDDVEIAGQTIRKGEGIVPLLASANHDTEAFGCPHAFNPERTERRHVAFGYGVHQCL
PimG    289 RVAVQDVEIAGQLIKAGEGIVPLVAAANHDENAFECPHAFDPSRSARHHVAFGYGVHQCL
ScnG    286 RVAVQDVEIAGQLIKAGEGIVPLVAAANHDENTFECPHAFDPSRSARHHVAFGYGVHQCL

StvA    349 GQQLARIELRVGFTALLERFPALRLACPPEEIRLRHDMQVYGVYELPVAW-
NysN    348 GQNLVREEMDIAYRTLFARIPSLTLAVPVEELPLKYDGVLFGLHELPVTWK
AmphN   349 GQNLVRVEMEIAYRTLFERIPTLELAVPVEELPLKYDGVLFGLHELPVTWS
FscP    344 GQNLVRVEMEIAYRKLFERIPELRLAVPEDQLAYKYDGILFGLHELPVRW-
PimG    349 GQNLVRIEMEVAYRKLFERIPNLELAVPTDGLDIKYDGVLYGLNELPVRW-
ScnG    346 GQNLVRIEMEVAYRKLFERIPNLELAVPTDGLDIKYDGVLYGLNELPVRW-
```

Fig. S44. Comparison of StvA (Slav_02825) from the *stv* BGC with cytochrome P450 monooxygenases from several polyene macrolide BGCs: NysN for nystatin (Brautaset et al. 2008), AmphN for amphotericin (Carmody et al. 2005), FscP for candicidin (Chen et al. 2009), and PimG and ScnG for pimaricin (Aparicio et al. 2016; Qi et al. 2015). Numbers indicate the amino acid positions from the first residue. Identical residues are highlighted in black, and similar residues are shaded.

```
StvB      1 VTGDPLRIEVSRDLCRGAGMCTLLAPEVFDQSDEGVVLLLDARPPAALRAAVREAADQCP
NysM      1 -----VRITVDPGRCVGAGQCVLTAPDLFDQDDDGLVTVLAGAADAADPGDVRDAAALCP
AmphM     1 ----MVHVNVDVDRCVGAGQCVLTASDVFDQDDDGLVTVLAQPSDPSTVETVREAAVVCP
FscFE     1 -----MRVTVDSEQCVGAGQCVLNAPEVFDQDDDGVVVLLRAEPDERDHEAVRTAGDLCP
PimF      1 -----MRITVDPDRCVGAGQCALNAPALFDQDDDGLVTLLTEPG-ADQYDKARLAGALCP
ScnF      1 -----MRITVDPDRCVGAGQCALNAPALFDQDDDGLVTLLTEPG-ADQHDKARLADALCP

StvB     61 CAAITLHKPSPMPEVPEVPEVPETP
NysM     56 SGAISVAAD----------------
AmphM    57 SMAITVVEN----------------
FscFE    56 SASVVLQED----------------
PimF     55 SGAITVHEG----------------
ScnF     55 SGAITVHEG----------------
```

Fig. S45. Comparison of StvB (Slav_02820) from the *stv* BGC with ferredoxin-like proteins from several polyene macrolide BGCs: NysM for nystatin (Brautaset et al. 2008), AmphM for amphotericin (Carmody et al. 2005), FscFE for candicidin (Chen et al. 2009), and PimF and ScnF for pimaricin (Aparicio et al. 2016; Qi et al. 2015). Numbers indicate the amino acid positions from the first residue. Identical residues are highlighted in black, and similar residues are shaded.

```
StvC      1 --------------MLGLAALGAAAMAGSTTIG-ATRALAADR----AGTPAFVPAVVIG
PimE      1 MFEN---QHLSRRRLLGLAALGGAAAAGMTTITSAPHAAAADRRSPQARSGSFVPAVVIG
ScnE      1 MFENQQNQHLSRRRLLGLAALSGAAVAGMTTISAAPRAAAADKRSPKAGSGSFVPAVVIG

StvC     42 TGYGAAVTALRLGEAGVRTVMLEMGQLWNQQGPDGNVFSGMLKPDKRSSWFKTRTEAPLG
PimE     58 TGYGAAVSALRLGEAGIPTLMLEMGQLWNKPADDGNVFCGMLSPDRRSSWFKSRTEAPLG
ScnE     61 TGYGAAVSALRLGEAGIPTLMLEMGQLWNKPADDGNVFCGMLKPDRRSSWFKSRTEAPLG

StvC    102 SFLWLDLANRDIDPYAGVLDRVNYDQMSVYVGRGVGGGSLVNGGMAVAPKRSYFEEVLPR
PimE    118 SFLWLDVINRDIDPYAGVLDKVHFDQMSVYVGRGVGGGSLVNGGMAVVPKRSYFEEVLPR
ScnE    121 SFLWLDVINRDIDPYAGVLDKVHFDQMSVYVGRGVGGGSLVNGGMAVVPKRSYFEEVLPR

StvC    162 VDSAAMYDRYFPRANSMLRVNHIDNGWFEGTDWYKFARVSRDQAQKAGLGTVHVPNVYDF
PimE    178 VDAAEMYDRYFPRANSMLKVNHIDKGWFEETEWYKFARVSREQAGKAGLGTTFVPNVYDF
ScnE    181 VDAAEMYDRYFPRANSMLKVNHIDKGWFEETEWYKFARVSREQAGKAGLSTTFVPNVYDF

StvC    222 DYMRREANGEVPKSALAAEVIYGNNHGKQSLDKTYLAAALGTGKVTIETLHQARTIRQQK
PimE    238 DYMRREANGESPKSALATEVIYGNNHGKQSLDKTYLAAALGTGKVTIETLHQVRAIHQQP
ScnE    241 DYMRREANGESPKSALATEVIYGNNHGKQSLDKTYLAAALGTGKVTIETLHQVKAIHQQP

StvC    282 DGTYLLTVEQKDADGRLLATREISCRHLFLGAGSLGSTELLLRARETGTLPDLSSEIGAG
PimE    298 DGSYVLSVDQIDTAGQTVAHKEISCRHLFLGAGSLGSTELLVRARDTGALPDLNAEVGAG
ScnE    301 DGSYVLSVDQIDTAGQTVAHKEIACRHLFLGAGSLGSTELLVRARDTGALPDLNAEVGAG

StvC    342 WGPNGNIMTARANHVWNPTGASQSSIPALGIDDWDNPAAPVFAEIAPMPAGVETWVSLYL
PimE    358 WGPNGNIMTGRANHVWNPTGAHQSSIPALGIDDWNNPTAPVFAEIAPMPAGLETWVSLYL
ScnE    361 WGPNGNIMTGRANHVWNTTGAHQSSIPALGIDDWDNPAAPVFAEIAPMPAGLETWVSLYL

StvC    402 AITKNPERGAFVYDAANDRANLRWTRDQNTPAVNAAKSLFDRVNRANTTMYRYDLFGKQL
PimE    418 AITKNPERGTFVYDKATDRAALRWTRDQNTPAVNAARSLFDRINKANGTMYRYDLFGPQL
ScnE    421 AITKNPERGTFVYDKATDRAALRWTRDQNTPAVNAAKSLFDRINKANTTMYRYDLFGPQL

StvC    462 KAFSDDFCYHPLGGCVLGKATDDYGRVSGYKNLYVTDGALIPGSIGVNPFVTITALAERN
PimE    478 KNFSDDFCYHPLGGCVLGKATDGYGRVAGYHNLYVTDGALIPGSIGVNPFVTITALAERN
ScnE    481 KNFSDDFCYHPLGGCVLGKATDGYGRVAGYRNLYVTDGALIPGSIGVNPFVTITALAERN

StvC    522 IERVIQQDIKAS
PimE    538 IERIIAEDVKAA
ScnE    541 IERIIAEDVKAA
```

Fig. S46. Comparison of StvC (Slav_02815) from the *stv* BGC with cholesterol oxidases PimE and ScnE from pimaricin BGCs (Aparicio et al. 2016; Qi et al. 2015). Numbers indicate the amino acid positions from the first residue. Identical residues are highlighted in black, and similar residues are shaded.

(a)

```
AmphRII     1 ---------------------MTGSTPSPQPLYQARPATATAEPP---AGVRARERATVA
FscRIII     1 -------------------------------MYDLH--MPPIELP---LLERDRELAALS
NysRII      1 ---------------------VPRSKARNQPTTCTPQCAPDAHGDPTMLLECGREQRLIG
StvRII      1 -------------------MERTTAAHPVRATPPVHRARPGGAGEPAALVGRDTEAGLLH
NysRI       1 -------------MRKQSGSSG----------------------LLTTLVGRDDELRTLA
FscRIV      1 MWLRWAGDRSRGAAPGRSGSAGPPSLAPSDARESPVPHRSRAVPPAPPLVGRAHQLDVLA
AmphRI      1 -----------------------------------------------VIHGRDNELTALG
StvRI       1 ----------VVVTVGTARAHG----------------------PGRSLDTHTAALDDLL
NysRIII     1 -----------------------------------------------MLLERENELARIR
FscRII      1 -----------------------------------------------MLLERTTEIALVD
AmphRIII    1 -----------------------------------------------MLLERGAELAAVR
StvRIII     1 ---------------------------------------------MRPLLHRQKELDLIT

                             Walker A
                         ****************
AmphRII    37 AVVDGLGTSGP--VLVTLSGRSGFGQSALVRLAADRAR-AAGLRVLRARVTPAESELPYT
FscRIII    25 AVIGELGSGRP--AVVTVTGEPGLGQNDLLRWAAAYAT-DAGLRVLSAHATPAEHEVRYG
NysRII     40 DLLHRLGQGRP--SVLSLTGRPGHAQNALVRWGACRAR-HDGLRVLRAQATPAERELRYG
StvRII     42 GLLGQLRTGRP--ALVEVHGPPGIGRSALLDHTAALAH-GTGVRVAAVRATAEEAGLPHG
NysRI      26 RHAAAARDGRA--GLVLLHGPAGMGKTSLLRSFTASD-VCRGMTVLYGTCGETVAGAG--
FscRIV     61 RHADAARAGRP--RLVLLDGPAGIGKTALLRAALAEDGPLAGMTTLYGSCRAVDVATG--
AmphRI     14 AQAAAASHGET--RCAVLTAPSGYGKSALLDALLRSP-ACQDMTVLRGRCGGASAGAGA-
StvRI      29 RHAAGARAGTA--RAVLLRGPSGIGRSRLL-AVASERLGAEGMAVR--SVAGLPGGASR-
NysRIII    14 AALDAAEAGDS--SLLLINGPLGSGRSALLRRIPELAG--DGTRVLRASAAWRERDFPFG
FscRII     14 AALGAATEGRS--SLVLLTGPLGIGRSALLQRLSCLADDRDDVRVLRAHAAPMEQDFAFG
AmphRIII   14 QALSAAAEGRS--SHLVLAGPFGMGRSTLLHELPRCAD-EGTVHVMRANAAPAEQDLAYG
StvRIII    16 GAMDAARHGSGGGSLVVVTGGLGTGKTALLRALPVRAE-RRGLRVVTASGAAFERDFEAG

AmphRII    94 AVTQLLEPLD-----------ARPAERPATSQGPNRREPFP----------------GLA
FscRIII    82 VVAQLLAEEN-----------RALAPRLFLTD--EQPGGLP----------------GLN
NysRII     97 AVLQLLAVLDGP---------HGSTLDAAIRHDGPPPLPVP----------------GIE
StvRII     99 VAGRLLARLPEAPARTGPVPYGTARPGAGRADGVRPRRTAD----------------LCA
NysRI      81 --YGGVRELLGGLGLSGGDARRSPLLEGLAARALPALTADPAGPDAATG-AYPVLHGLYW
FscRIV    117 --YSGVRALFGGLGLTGRKGRTSPLLVGGARRALPALAADPGELDADPGSTFSVLQGLYW
AmphRI     70 --YAGLRTLFAPADAPAPD--HHPEAAGADDPAVFLPGPGDGRPFTLTS-AYPVFRRLSS
StvRI      83 --IRAVDALLE-------------PPPEGPCARPGGAHEPRD---------THRDWRRLHR
NysRIII    70 IARQLFDHLLSGAGGAGPAERTAGAEH--FSR-LMDTGDRPTGTGPALEVSQAVLQGAQA
FscRII     72 VVRQLFETLLGDSPEDARDRWMDAHAS--FARHVLADDAAPPGADQAIAATEAVLHGLLS
AmphRIII   71 VVRQLFDSLLTAASGPDREQLLADAGA--ARR-VLDDDVVP----DLEAPDCAVLRDLRL
StvRIII    75 VLGQLLAPLLPGTPVAPPGDCAGPSAGPLQDD------------------------GLLA

                           Walker B
                      *****************
AmphRII   127 GLLRAARERPTVLAVEEAHWLDRESLHWLQALLRRS-AGTPLAVVCG---GNDTAAADPG
FscRIII   113 GLLNAVRDRPTLLVVRDTQWLDPASLRWLEALARRL-PRAPVALLTS---TTGTALTRPE
NysRII    132 EVLRRTGTAPTLVVVEDVQWLDPASLTWLQILLRHLGPDTPLAVLASSCGDTTAFDTDPK
StvRII    143 GVLEAAAGGPLLLVVDDVQWADPQSLRGLRSLARRM-TGVPLMLLTSRNSAVPATADERI
NysRI     138 LAARLMAQRPLVLVLDDVHWCDERSLAWIDFLLRRAEDLPLLVVLAW-----RSEAEPVA
FscRIV    175 LAANLMADGPLVLVLDDVHWCDERSLRWLDFLLRRADGLPLLVVAAH-----RTGTGLTA
AmphRI    125 HVLRLTADRPLVLVLDDAHHCDEHSLRWLDFLLRRAAGRPLLVLLAR-----SSGAQLRA
StvRI     120 RALDLLADGPTALVVDDAQWCDEASLRCADFILRRSADQPLLIVLAQ-----RTGVPGPG
NysRIII   127 LLADASAERRLLIIVDDLQWADGPSLRWLAHLTRRLHGLRALLVCTL-----ADGDH-RG
FscRII    130 LLANVSADSRLLIIVDDLQWSDVPSLRWLTYLAKRLHGLRAVVVCAL-----RDGDP-RS
AmphRIII  124 LLAGLSATAPLLILIDDLQWADACSLRWLAGLAPRLDGLRMLVVCTL-----RDGDP-GA
StvRIII   111 LVAEYGAHTPLLMIVDDLQWADAASLRWLGLLAGRVAGLRVTLVVTL-----REGDP-GE

AmphRII   183 WQSALGSVPGPLAHHLTLAPLPPEEVAAEVARVCG--PADDAFTAEARRISAGHPTVLHD
FscRIII   169 WSVGTPLTGLATTIELALPPLTSGGTATAVLRAFG-APGDPAFTEALAEATRGIPAVVHD
NysRII    192 APAVPGPPDTVPVARFVVPALTDRGVAATVRAVCG-TPGDEEFIAALTSATAGNPAILRD
StvRII    202 DVCALAGEDAVVHHALTLGPLPDGAVGEFLAGTWR-APVDEPFLADAVRHAEGSPALMRS
NysRI     193 PAVLADIAAQRRPTVLGLHPLGPDDIGEMVRRVFR-TTAAPSFVSRVAAVSGGNPLALAR
FscRIV    230 PDALADLVAHHLPASLGLGPLDTAEVAELTAHSFPEQDPLPSFVGRILSVTGGSPLEIVR
AmphRI    180 PRAWADLLAHPAVSVTSLGPLGADAVGGLARQVFG-VPAHPLLAQQVAAVTAGNPRTVER
StvRI     175 TRVLTEILAQDRCALLELGPLDERDTQRMITRVLG-APADPAFVRRCTEITGGNPLLLDR
NysRIII   181 RYPLVREVAGAAHTVLRLAPLSRDATRVLLAGPQG-RPPQDALVRAVYEASRGNPLFLTA
FscRII    184 HHTLAREIREAGTQTLRPASLSLTATHELVREHFG-EAGDEFVQACHEASVGNPLFLLS
AmphRIII  178 RDPLVHEVVSTAVRVLRPVPLSPAATGELVEAYLG-DSPDEAFVRACHDLSRGRPLFLRS
StvRIII   165 DDPRLQQLVEGASRVLRLGPLPPEGTADLIGALTG-RPVDPAYARACHEVTGGRPLILTA
```

```
AmphRII     241 LVQRFAALGHRPA-ADRVPALRAIGTEVLGDQLLYAVRGLG-PEAGEVVRALAVCGDLLD
FscRIII     228 VLDRFARAGYSPR-ADRIDPLRALTAEVVGDHATRALSDLRDPAAVDALRALAVCGDLLD
NysRII      251 ALRAFVDHGLPAD-ADHLPELHALTAGVVGDHTVRALDGLP-AEVNAVLRALAVCGDLLD
StvRII      261 IVGRFVRHGWTPG-TGPAGRLEESAAEAVRERTVRTLALLP-DELLGVLRAIAVAGPDCT
NysRI       252 LLDELRAEGVRPD-AAGERRAAEVGSHVLARSVRCLLERRP-PWVRGVARAIAVLG-PEC
FscRIV      290 LLRDLRSAGLGPD-DTGTARLAQAGGKVVAASVRGVLEHQP-DWVRQVARAVAVLG-DED
AmphRI      239 LLGELRSQGAAPD-EAGAQRASELGGAMAAGSVRQLLDREP-PEVRSVATAIALLDGTRP
StvRI       234 LLHTLSTVGIPPG-ADALHQLSTVHQTVLASLVPEFLAAGP-GPVRQVATALAVLG-RAA
NysRIII     240 FRSALRATGRPPG-GDHFGAVRELSPTVLRDRLAGHLRIQP-QPVREVAVAVAALGDHSD
FscRII      243 ILVGTGFLGRRPL-AEHAETARRLRPSQLRERLASILRTQP-APVRDLAAAIAILGEQSD
AmphRIII    237 MLSELALTGCRPL-AENAGLVRTARPTHLRDRLLNCLLLQP-RPVRDAAAAIATLAGQEE
StvRIII     224 VLDALSGPGEGPGSADDVRAVHAVRPAALYERFAVVLRDQP-APVREFATALAVLGDGAE

AmphRII     299 LQVVRTLTRVRSFNENRLRAALARTVGIRTLPNGRLSVEP-ELRVRVLEEMTADDRTELH
FscRIII     287 FPLVCTLAGPHSVSESRLRAALAAS-GLTTLRDGHPRVQDAVVRARVLEEMPAADRAELY
NysRII      309 FHRVRALAGAHSLSEDRIRTLLASV-GLTVSVGDKVHIRFPASKARVIEDMPAAERADLY
StvRII      319 PGLIDALAGPRALGTSRALALLRGT--GLLAPGPRPALRTAAAARAVLAELGSEHREELH
NysRI       309 TELLAALAGVPAATVDEALLVLRRA--GILAAD-RVDFVHDVVRSAVLDDVAPPTLAELR
FscRIV      347 RTYLAALGRVSVVHVEEAVEILRDA--GLIHPG-HLELSHDLVRAAVLDAVGASGVAVLR
AmphRI      297 PECVTVLADVDQAQADEAIAVLRHA--GALTPD-GAGLVHEAVRSELLEPLGSRRTAELH
StvRI       291 VDPLAALSGLSARHVESALGRLRHI--EAVPAD-SPPRMREGIRTAVLAALDCEELRGLR
NysRIII     298 PVLLAQLAGVDEIGFAGARRALVDA--GLLARGRDVRFVHGVVRDAVDSLLTLDERERSH
FscRII      301 APTLARLAGLDSIGYAGALRALGAL--GALAAPDEPRFIHRSVRDAAESTLTMVQRERMH
AmphRIII    295 PALLTRLAGLDDVGFTTALRVLHQL--GLTAEEDRARFAHPAVREAVECSMSAAERQCWH
StvRIII     283 EELIGRLAGLDGTGRIEALRVLRRL--GLVCEGPALRFVHPVVGEAVEEAMTPAEAESIR

AmphRII     358 IRAAELAHRAGADDRAVAELLLAARPTGSAWATRTLRRDAEHAVREGEHARAAVLLAR--
FscRIII     346 ARAAGLAQRVAADDQGIADLLLLARPVGDPWAVDTLRRGFTSALRGGRRDLAVAYLAR--
NysRII      368 VRAAELTHSCGVNDEDVAHLLLRSSPLGAPWVVPLLRRGFAAALRREDHHRACACLSR--
StvRII      377 ARAADWAHRAALPDRALARMLLDARRIGATWAVQALRREAEECRATGRPGRAARLLER--
NysRI       366 TNAALLLSDAGRPSEELAGQLMLLPVLDQPWMAAVLRDAAAQAESRGAPEAGVRCLY---
FscRIV      404 RRAARLLSDVGRSPEEIATQLLLVPGTPDDWAVSVLRDAAAQAEQRGACEAAARYLE---
AmphRI      354 ARAALLLSDAGRPVEQVAQHLMRMPCAQEPWMSAVLRDAAARAEQRGAFAEAAGYLR---
StvRI       348 VRAARVLSDAGRPAAEVAEQLALLERIDEPWMFAVLREALAEAPTRATVAAAGRWLRGAP
NysRIII     356 DDAADLLYRCGRPAEQVAGHLLAVVHPGRPWSEAVLRSAAHNALRAGRPADAARYLRR-A
FscRII      359 DEAAALLYAAGSPAEQVAAQLMAVVTRRQPWAVDVLRAAADTALRRGAPDTAAGYLCR-A
AmphRIII    353 EAAAEVLYESGLPAEQVAAQLMAIPTARYPWAVPVLRSAADAARRRGTPEPAARYLRR-A
StvRIII     341 VHAALLLHLGGHPAERAAAQLLAASSCHEDWESDVLRAAAASALRRSAPDTAARYLRR-A

AmphRII     416 ASEEEQDPAEHARLGLELAAVQLIIEPEAGDLRIERLIRT-------------PGTPAAV
FscRIII     404 ALDEPLAAEDRARIEFQLASVEMVTAPTAAERRLGGLIRATR-----------PGPGAGL
NysRII      426 ALQEPLDPRERSLLTLELAAAEAVARPEAGDRRLGELVRSTVA------DTDPTSSGEGV
StvRII      435 ALREPVPPALRIRLLTDLSAAVLPTEPEAASRHLYRALQTPADGSAAVADTAADAETGRA
NysRI       423 --RVLEVEPDNVAVRIQMARALAEINPPEAMRLLKEALSLAGD----------VRTRAQV
FscRIV      461 --RVREAEPKDPDVLSRLGKALAETDPARSVTLLHEAHSLTTD----------VRARAAT
AmphRI      411 --RVLEAEPDDAEARLRLALAVAPEDPLSAVPLFRTALAGTPD----------TFARAGI
StvRI       408 GRRLTTAERADLHVELARAAARTIVAPADALWHLRQALALLPH----------PRDRAPV
NysRIII     415 LLHHRTQDGCRARILVDLATAERALDPDACVRHVSQAVALLDT----------SRDRAAA
FscRII      418 LLDSPAAGVGRGRLLVELGTAERGFDPLACERHIAQAMTLLPE----------PRDRAVA
AmphRIII    412 LLEVAEQDRKRAHLLVELATVERSFDPPAAESHIAQALPLLPA----------ARDRAAA
StvRIII     400 LLGSSPEGPDRAELLVELAMVERDFAPRAALRHLAQALFLFPE----------TDQRAPV

AmphRII     463 RLYAADLGLTAGGSETVSRSLADALTVT----------------------SGTERDDLLA
FscRIII     453 RARATDLCLLGGDTRAARHALAGAIDSAPAAPEPRRGPTGAPAVPPEAEPASTPRTHLVP
NysRII      480 GVRAIDLGFARGNSEWVRRTAGEALPYAG-----------------------PADREELVA
StvRII      495 LLRAAELLVARGDVDTPRPRIARAADAVR--------------------PGSAERSGLRA
NysRI       471 AVQYGFTCLAVQESPSGVRMLEDALAELTAELGPE------------PGPVDRELRTLVE
FscRIV      509 AVQLGLTCLAVQQSPDGARALTEALDALDAELGPE------------PEPADRELRTLTE
AmphRI      459 AVQYAMACLSLPLPATCRAEILGALEAARDALGS--------------GPERDRLRDQVA
StvRI       458 AVQYAMMALGTRRAPEALRLLERVLGDLCSGTGAG------------PGPADREVRVTVE
NysRIII     465 VLRIPPSLLAAP-SPSAVELVRQAAAGLDEPGQRD------------EEGADELALRLEA
FscRII      468 ALRISPTALGPA-PLTAVDLLRQAAEDLG-PADGL------------TGTDRDLALRLEA
AmphRIII    462 VLRLSPWVTSPM-TRSGRALFRRAADDLRDPYTR-------------DDPGAEEVLRLEA
StvRIII     450 AGRIPPFLLDGA-PSTTVDTVVRTAAELGEPATL-------------TGTARGHALRLEA
```

```
AmphRII     501 LF-WAADPTGQH------------------------------------------------
FscRIII     513 HRPYAGTPPGATGPDGRRSAAPDLPGTPRAHDAPRSPGPEAAGRPDTAGPDEDPAVAERH
NysRII      518 LFWLAAVR----------------------------------------------------
StvRII      535 LHWITEYS----------------------------------------------------
NysRI       519 SVLLIVGADEKV------------------------------------------------
FscRIV      557 SALLIVGSDEKA------------------------------------------------
AmphRI      505 AVLLLMGRRGTG------------------------------------------------
StvRI       506 SALLATAVNDRS------------------------------------------------
NysRIII     512 WLRHSGHENPVE------------------------------------------------
FscRII      514 RLRHCGHEDPHE------------------------------------------------
AmphRIII    508 RLRYTGIDDYAQ------------------------------------------------
StvRIII     496 RLRHPALAGPEE------------------------------------------------

AmphRII     512 -------------HGDRALSLPTVPPLPQSPATPAQAAVRAWQLALYGESLHEVRSLAQV
FscRIII     573 ELTVLCRVASFLRHDDADLDVLPVPALPDAVHNPAVAGVWAWEHGVAGVHREEVRRLARS
NysRII      526 --------------DDDAPMIPVVPRLPDRPVPPAQAGARAWQLATAGEDADKARKLARI
StvRII      543 -------------RPDPADGVEPLLPDSCPLERSAEAAVAAWRTAQRGRNIDRARRLART
NysRI       531 ----------TIGAVRDRAARLTMPPGDTPAQRQMLAMTTVLTAMDGRDARSAVDQARRA
FscRIV      569 ----------TLPDILRRTEGLTPQPGDTPAQRQQLAMLSVLSAAEGGGAEETVGRARRA
AmphRI      517 ----------VAARQHPVTAALPYQPRTDLLQAEALAG--LRTALAGHSRETALKEARHA
StvRI       518 ----------ALPAARARAAALPVPSGHSPADRRLLAALSAFAALDGGSAGRAAALARQA
NysRIII     524 ----------LASSVARLRRMGARPPVDSVAERELVAVLLSAGALSGRLSAAEIADTGNR
FscRII      526 ----------LAASAHRLKELGPEPPVDTGGERELTIALLGAATLGCHLPASEVVRLAGR
AmphRIII    520 ----------LADAVERLRAL--EPTVRLRGGRELLAVLLYAATLTADGPSAEVARQART
StvRIII     508 ----------PAACGARLRALGPAPSLDTAAERELVTVLLHGAALAQTVNAAEAVALAER

AmphRII     559 ALAHDRGPDGPLVQPVLAACKALCLTDRLDDAEFALNRLLGALGGSRRRLAAPQILALRG
FscRIII     633 AFVPAATGRPLLVVPRLLGCRALLLTDDGDEAENHLGALLDESHRERAAISVAHILTVRA
NysRII      572 ALTG--GVNESLMMPKLAACAALFATDDNDEAVHGLDTMLTAARSAHLRSMAARIFNLRA
StvRII      590 ALAPQVREHVPLTVR-AAAAHALVLTGDTAEARRALDEVLVQAERSDARAVAGLALLVTC
NysRI       581 LRAPGVELEP---WSLLSASFALSLADEVADAQYALDLMLQYGQDNAAVWTYVLALSTRA
FscRIV      619 LRAPGVPLGV---WSLLPTSLALSLADENEAAEEVLETVLRGSGDTAAVWTYVLALSTRS
AmphRI      565 VAAASPQQPP---WLLFTAAITLGLADETTEAHRALDAMLRRGGDEPTAWVRVLAGSLRS
StvRI       568 LRADEPGTAG---WGVFCCSAVLALADSVDEALTALDQALSPAETQPPLWPRRAALAGRS
NysRIII     574 ILEREPATAAHAHTPLPLVMLSLFVAESVQGVASWLASEQHTRRRYATGADDVLLTAERA
FscRII      576 VLDREPATSARIHSTLPLAIITMIAADSVEAVDSWLAVEHRANERGTTPAN-ALVHIEQA
AmphRIII    568 ILDWEPATAPGGGSVMQLVMFALVGADALQDAESWLNAAAGSGP-GDDGAIDAMGHARQA
StvRIII     558 LLQHEPAATDHVHTALPLLTDVLVAADSLATIGPWLDTAYERARRENADVPRALIAVELT

AmphRII     619 ELNVRRGRFAQAERDLEAAERGARELGRSAALLPHLYGIRTVVDVESGREESARERAATP
FscRIII     693 ELHIRHGRPDAAARDLAAAQAELPLDRLHPLFLPYWLALSMITDLQNGHTERARETAARP
NysRII      630 RIHLCAARLEAAERDLDSAERALPPTSWHPRALPNLIATRILVSMETGRPDRARRLAEAP
StvRII      649 LAELRAGRDTAAAEALDRCEGIMPGRCWHPLMAPGPLALRALLHLRRGDRTAAERALAAE
NysRI       638 LLHHGVGAFPEALADAQTAVEILGEERWADGAVLPRVALATALVDRGEPERAEHVLDGIT
FscRIV      676 LFRLENGAVPDAMADAQTALEILGQERWGDTATMPHTAYASVLTERGDPGRALEALDAIK
AmphRI      622 FLLHRDGAVLDAAAEARSAIDTVDRTGCDVRLVAARAVLASVLVDRGEPRQAERLLSRMG
StvRI       625 LIRHGTGDVPGAARDAWAAVEVVGP---AGAPPFARIALSSVLLSQGEARQAAAVLDALA
NysRIII     634 FVLVTQGRPAAAREHVERALVMDAG-DWS---EPAVMMFAAVAFELRDPALSERILERIR
FscRII      635 LVHLGRGRLAQAREQSETALGMTEP-AHQGLDTAATMTHAIVALESRDPSLAEKVLRRAS
AmphRIII    627 LFHVARGRLPLAREQAALVLEGAGT-IGEESRCFVYGVLGALALSARDVQLGSRIRAAVA
StvRIII     618 RLALARGDLTGAAAHAAEALAQDAT-DWATLQTLAPVVLA--ALEARDTALCERLLSGRR

AmphRII     679 LP--AAAEDSEHWPRLLFARGVVALRRGDPRAARDQLQECGRRLLSRHHLNPALVPWRSL
FscRIII     753 LP--PLAHESATTAQLLFARGVLARTDDEPHQAREHFRACGRWLLRHGCANPAVQPWRSL
NysRII      690 VP--AGGEEGVWWPALLLARARVAADDGDWEEALRLSRECGRWLFRRHWANPAMLSWRPL
StvRII      709 LP--PGAEGGLPWAYLLYARGRVALAGGTREQAFADLLECGRQLLARQAVNPAVLPWRSA
NysRI       698 R--PRLERFVIEYHWYLQARAYARWVRGDFQGALDLLLACGRSLEESRFSNPAFVPWWAD
FscRIV      736 R--PHLDRFVWEYHWYLMARGRALAADGDLDGALQVFGSCGASMAEAGLTNPVLAPWWLE
AmphRI      682 R--TEPHRSALASLLYLHARARTRWATGHPAQALRLLRECGWAQETAGVQNPVLAPWWAD
StvRI       682 RSAPDLDRSVWEWQHYLYTKGRILRELGDPEGALELWRRCGRSEEEAGIANPVLAPWWLP
NysRIII     690 DR---RPAGLALTATGQMLQAAVDVHFGRGRDALDTLLACGRRLETVGWRNSALLPWRPY
FscRII      694 RI---RPQGLTLTGILRLLEATREIAAGDQTGALDSLHSCGRQLEAAGWHNPVLFPWRPW
AmphRIII    686 D----RTLAPVPAALLGLLRMAEQARSGDAAAALDTAMDCGRQLECAGWRNPVLFPWRPW
StvRIII     675 E----EAAPSHRPSLRRLIHGSAAVLRDDLPTALDCFLEWGRAAERAHWRNPAIVPWRTW
```

```
AmphRII      737 TALACHTMGEPAEARRLTEQELALARRWGAGSTVAWAKLS--AVLTAGREDRLTGIRSAM
FscRIII      811 AAEAAHTLGDTEEAARLVHEEVRLARRWGAAAPLGRAQLS--LAVVTE-ENRVENLRAAV
NysRII       748 AAEACLKLGDVTEARRLRDEELFFADRWGTASARGIARLTTRRLFDDDGDRAVRRIREAA
StvRII       767 AARAREPG--DPLAAALLAEERRRALLWGSPAAMAAALGEGAAAAGAGGTPAPGRGRPVS
NysRI        756 GAVLLATLDRHDQARELAAYGSELAERWGTARGLGLAFMA--QGVAAPGRAGIDHLTEAV
FscRIV       794 TACLLGEAGRGEEAGRAAAHGTRLAERWGTRRALGYAALA--RGAAARGTARTGALREAV
AmphRI       740 TCLILAAVRRPADGRELAEQGAERARRWGTPRALGLAALA--EGVLTPGRAGLDLLTEAA
StvRI        742 TATTLGGLGRGAEAAGIVAAARERARRWGTPRALGLGLVA--QAAVVEGRDRTGLLARAV
NysRIII      747 AIGLHQRLGETDAALQLAEDELRWAREWGATTNLGRALRL--KGWLLQD-EGLDLLRESV
FscRII       751 AVGVHRRLGDIRTARSLAEEEYARAEQWGAPVGVGRALRL--LGRLEDGGRGIGLLREAV
AmphRIII     742 AISFSDQLGDHHLARTLAAEEYARAREWGAPAAIGRALRL--RARTDAADERVELLREAV
StvRIII      731 ASALHYRMGRPDRAHDLMEEEYARALSWGSPVAIGRAQRG--KGAITEGVRGIALLRESA

AmphRII      795 GTVRSAPTGPEYARVLAEYAALEMTESGGDRRTAAVSLAELSALTVAHP-----------
FscRIII      868 TTLSAASARTAYTRAVIELAAAEREEN--ERRTAVALGPAATVLPPVAPPP---------
NysRII       808 ALLRDSPARLAYLWSRLSQAGAETAHG--DTAAAARSWQAVARMTAAHP-----------
StvRII       825 WQYRQALVALGTAPSSGHLTVDSLLGS--DRGAAAAPRPATPALPLPSRSASGSASGSDS
NysRI        814 SLLADSPARAMEARAELLLGHAHLKRDDLRAAREHLRAAADLAQRCGAV-----------
FscRIV       852 ALLADSPARSGHARGLLLLGRALVEDGAVREGREHLREAVGLARRCGCV-----------
AmphRI       798 HQLSTSPAVAEYARAEHALGQALLQYGDEAGARERLRRAAGIAGGCGAL-----------
StvRI        800 DLLSESPARLELAKAEYQLGRELLALGDARGARGHLRRAIGLATRCGYR-----------
NysRIII      804 EILRASSYATELARTLVVLGRRLPGG---PEAEAVLREAAGIAAACGVP-----------
FscRII       809 TVLRGSANQLELARALSTLAERLGGG---AETEALAREAATLATSCGAP-----------
AmphRIII     800 ATLRGSANELELFMAVRDLGRMLGDG---PEGREVLREAGELAESCGVP-----------
StvRIII      789 LTLEGSANALERARTSLLLGRRLLAQGRQAEAEDRLTRARTEALDCGAP-----------

                                                                LuxR family HTH motif
                                                                  *******************
AmphRII      844 ------DAPMAVR----ARALARAATGTVSVPG-----WDALSAAEQQSALLAGRGRSNR
FscRIII      917 ----GPHVPSAVARPPGPRPAVLTGRRTAKAPGHDAAVWHGLTAVERETAALAAQGLGNR
NysRII       855 ----ASRLATAARTLTVPSVPVATAPPTAVVPPG----WRDLSEAEKDTVLLAARGHGNR
StvRII       883 RSASGPLPPSRFPSVSPPPPASVRTPAGGAPGRTARGGGPLTRAEQRVAALAAGGLGNR
NysRI        863 --------KLGVDARKLLVTAGGRVRRMTASPLD------MLTGMERTVADLAVTGASNR
FscRIV       901 --------ALARRARDELIAAGGRMREVTASPLD------MLTGTERTVAGLVASGAGNR
AmphRI       847 --------ALAESARRLLVSAGGRMGHTPGTPAGG-----LLTTTELKVAGLAVSGASNR
StvRI        849 --------VLAAVARDALVTAGGRMPQLATVPLD------SLTHSERRIATLAERGHSNK
NysRIII      850 --------WLAERAELGLGSAIVP----PVAT---------LTPSERRVASLVSRGLTNQ
FscRII       855 --------GLAERAARGAGHRSGPPA-TPEDV---------LTPTERRVVGLACRGLTNQ
AmphRIII     846 --------WLPG-PVAAEGRPSAP---AVRTA---------LTRTESRVASLVVDGLTNQ
StvRIII      838 --------WMVERAVRALQGIAGSRGEDVAAT---------LTRTERRVAALASRGASNK

                 ********************************
AmphRII      889 DIAALLQISSRAVELRLARVYRKLRLDGRRELRELVRAMEGH-----------------
FscRIII      973 EIATELAVTTRAVELRLSGVYRKLRIRGREELRALVQEAEGS-----------------
NysRII       907 QIAEQLAVSRRTVELRLSNAYRKLRIGGRKELYLLLEALEGPVADAS------------
StvRII       943 AVAEELAVTPRTVELHLTKVYRKLGIRGRPQLAAALRRTAGGAPGA-------------
NysRI        909 AIAEALFVTVRTIETHLTSVYRKLGVGGRAELSAVLETRTATSGRQPPAWVSQARGRA-
FscRIV       947 EVAESLFVTVRTVELHLTSVYRKLGVARRGDLTEALREAGATARPAAERRPGHAKRRNP
AmphRI       894 QIAQRLFVTVRTVESHLTNVYRKLGVRRRDQLVVALRAVHGRSWPLPGWPVPEGA----
StvRI        895 GIAEALFITPRTVEMHLTNVYRKLDVRGRADLPRGLGRHPGAPRPADGLSATS------
NysRIII      889 AIATELGVSSRAVEKHLTSAYRKLGVSGRRELVNALPGR--------------------
FscRII       897 EIAGALGVSSRAVEKHLTHAYRRLGISGRRELIVLFSRAEYAGTGD-------------
AmphRIII     885 EIAKELQVSARAVEKHLTNSYRKLGITGRRELAAVLREIEEHVPG--------------
StvRIII      881 AIAERLEVSSRAVEKHLTHVYRKLRVDGRTGLAAMARLLSDGAGPVHP-----------
```

(b)

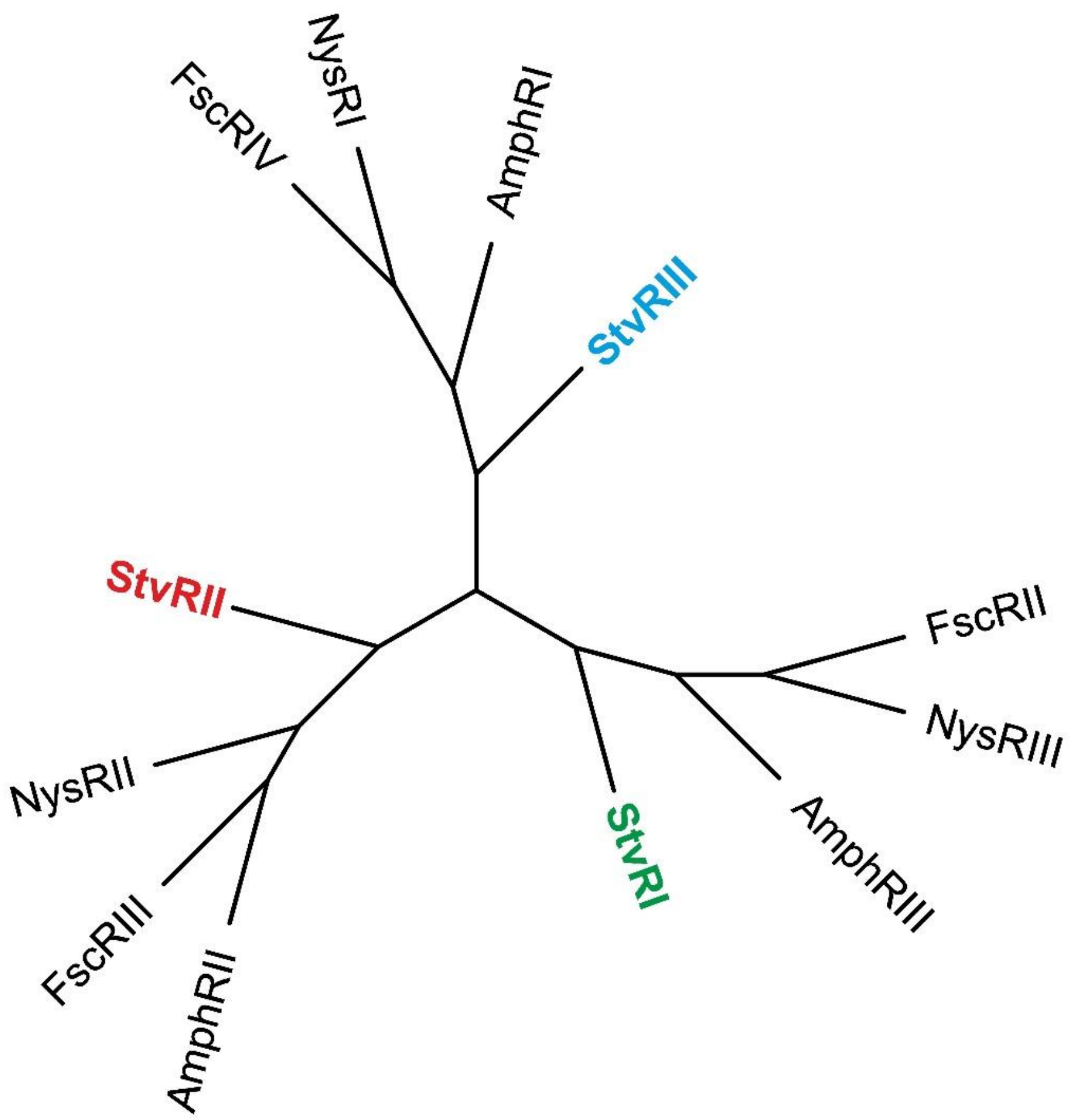

Figure S47. a Comparison of the putative regulators StvRI, StvRII, StvRIII from the *stv* BGC with the ATP-binding LuxR family (LAL) transcriptional regulators from several polyene macrolide BGCs: NysRI, NysRII, NysRIII from nystatin BGC (Sekurova et al. 2004), AmphRI, AmphRII, AmphRIII from amphotericin BGC (Carmody et al. 2004), and FscRI, FscRII, FscRIII from FR-008/candicidin BGC (Zhu et al. 2020). Numbers indicate the amino acid positions from the first residue. Identical residues are highlighted in black, and similar residues are shaded. The NTP binding domains Walker A and B (Walker et al. 1999) are indicated by red asterisks, and the LuxR-type HTH domain (De Schrijver and De Mot, 1999) is indicated by blue asterisks. **b** Phylogenetic tree of these homologous regulators.

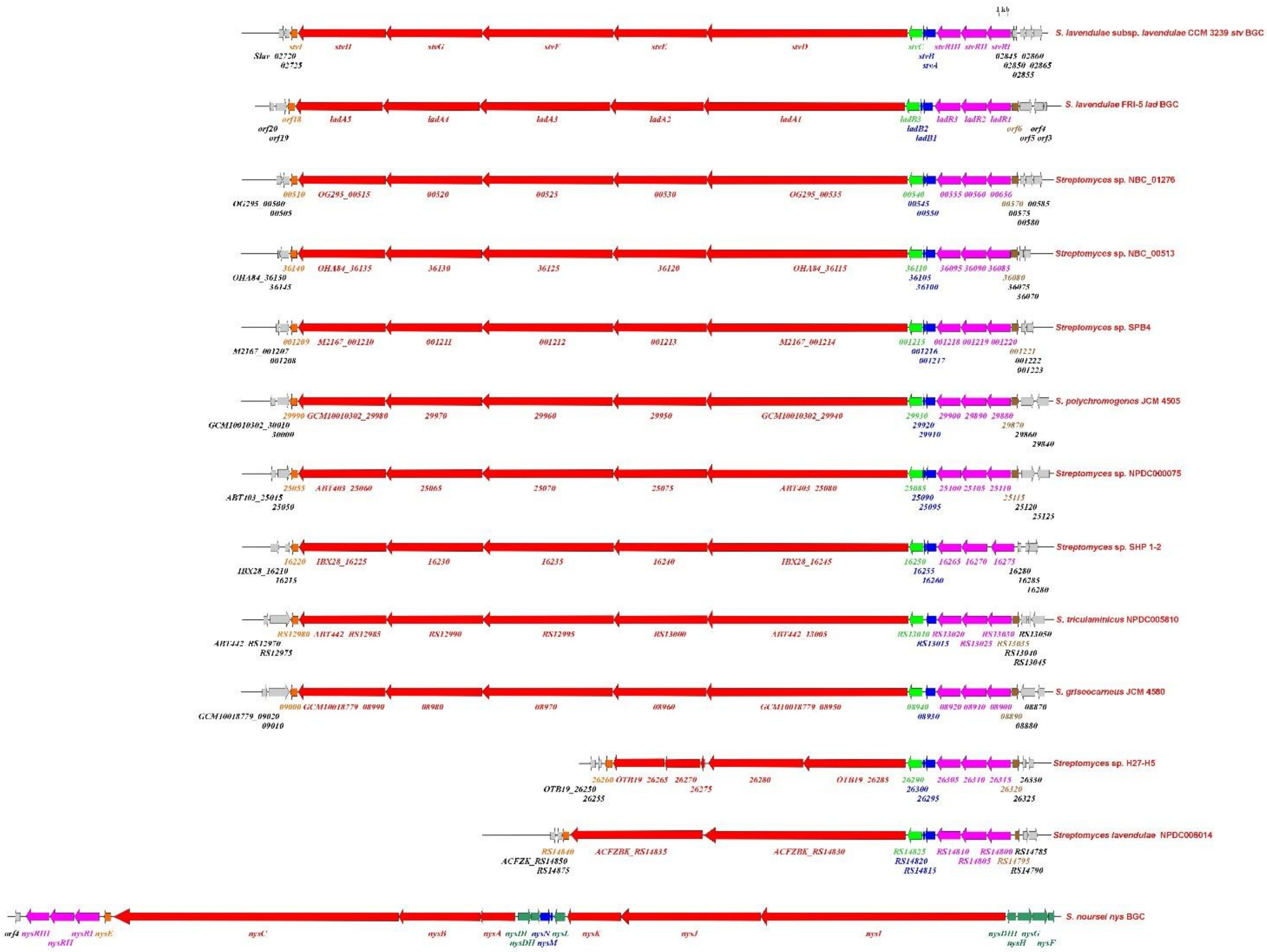

Fig. S48. Physical maps of the *stv* BGC from *S. lavendulae* subsp. *lavendulae* CCM 3239 and related BGCs. Thick arrows indicate gene direction and size; red arrows, PKS I biosynthetic genes; blue arrows, P450 monooxygenase and ferredoxin coupled genes; green arrows, cholesterol oxidase genes; orange arrows, type II thiesterase genes; brown arrows, 4'-phosphopantetheinyl transferase genes; pink arrows, regulatory genes; and grey arrows, genes with no function in polyene biosynthesis. BGC accession numbers are: *S. lavendulae* subsp. *lavendulae* CCM 3239 *stv* BGC (CP024985), *S. lavendulae* FRI-5 *lad* BGC for lavendicin (LC815377), *Streptomyces* sp. NBC_01276 (CP108442), *Streptomyces* sp. NBC_00513 (CP107845), *Streptomyces* sp. SPB4 (JARXYD010000003), *S. polychromogenes* JCM 4505 (BAAABV010000015), *Streptomyces* sp. NPDC000075 (JBEPFL010000023), *Streptomyces* sp. SHP 1-2 (JACVCW010000013), *S. triculaminicus* NPDC005810 (NZ_JBEXXL010000010), *S. griseocarneus* JCM 4580 (BNBL01000001), *Streptomyces* sp. H27-H5 (JAPNLK010000057), and *S. lavendulae* NPDC008014 (NZ_JBIBIU010000008). As an example of a well-characterized macrolide BGC, the *nys* BGC for nystatin in *S. noursei* (AF263912) is included.

```
LOCUS       CP024986              89942 bp    DNA     linear
DEFINITION  Streptomyces lavendulae subsp. lavendulae strain CCM 3239
            strevertene A2 biosynthetic gene cluster, complete sequence.
ACCESSION   CP024986
SOURCE      Streptomyces lavendulae subsp. lavendulae
  ORGANISM  Streptomyces lavendulae subsp. lavendulae
            Bacteria; Actinobacteria; Streptomycetales; Streptomycetaceae;
            Streptomyces.
COMMENT     the region from the position 572,874 to 662,815 in the strain
            genomic sequence of the Acc. No. CP024986
FEATURES             Location/Qualifiers
     source          1..89942
                     /organism="Streptomyces lavendulae subsp. lavendulae"
                     /mol_type="genomic DNA"
                     /strain="CCM 3239"
                     /sub_species="lavendulae"
                     /culture_collection="CCM:3239"
                     /db_xref="taxon:58340"
     gene            174..836
                     /locus_tag="SLAV_02720"
     CDS             174..836
                     /locus_tag="SLAV_02720"
                     /inference="ab initio prediction:Prodigal:2.6"
                     /codon_start=1
                     /transl_table=11
                     /product="hypothetical protein"
                     /protein_id="ATZ22467.1"
                     /translation="MYEIKGVRATPGGRISVRFHQHGYVERPDRRAGRVAETLFGEGA
                     GCLTALVLLASVGLFTLLRKNGAPEAAVTACMWVFVVAVCYGAVIALLHWASGVADVL
                     LIAVTFPALLIPGYRRMLLRRWRGGAPESEPGWVPGAALSGVWHQVDPQGGAVVTVQR
                     TDGSVTAYVPPADKARNLHDHFDTLLRQGRPAPAPYQQPAYGAPGYPPQPWQGNPGHR
                     HD"
     gene            complement(979..1476)
                     /locus_tag="SLAV_02725"
     CDS             complement(979..1476)
                     /locus_tag="SLAV_02725"
                     /inference="ab initio prediction:Prodigal:2.6"
                     /codon_start=1
                     /transl_table=11
                     /product="hypothetical protein"
                     /protein_id="ATZ22468.1"
                     /translation="MGDHGGRAPTPEPSPATGPCSPFRWRTDWACTSSENAGDDAGVH
                     YLITHPDWEHAEFLARDDGHWRGPGLSWPELIGAADNGLPGGTTTDPDTRLLLLLPAL
                     GDADLTPRQGAAPLGSTPFHGDRSPEDCRPGACRATPRATATCPRRAEYTTWLAALTT
                     DSPPF"
     gene            complement(1529..2308)
                     /locus_tag="SLAV_02730"
     CDS             complement(1529..2308)
                     /gene="stvI"
                     /locus_tag="SLAV_02730"
                     /EC_number="1.1.-.-"
                     /inference="ab initio prediction:Prodigal:2.6"
                     /inference="similar to AA sequence:UniProtKB:Q70LM8"
                     /codon_start=1
                     /transl_table=11
                     /product="Thioesterase II"
                     /protein_id="ATZ22469.1"
                     /translation="MSTPTPAPVSTDNGLWTRRFQPRPDAAVRLVCLPHAGGSASFYL
                     PMARTMPDFADVLCVQYPGRQDRRAEPLIDSVPELADRVFTALLPWADRPLAFFGHSM
                     GASVAYEVARRFEREKGIVAAALFASGRRAPSAPRHETVHLRDERGLIEEVKSLSGTD
                     TQLLGDEEVLRMILPAIRADYRAAETYAPEPGEPLHCPLVAMIGSEDPKVTPQEAAAW
                     AAHTEGPFTLKVFSRGHFYLVRDQAEVIRTMTDHLRTPVTP"
```

```
gene            complement(2374..12531)
                /locus_tag="SLAV_02735"
CDS             complement(2374..12531)
                /gene="stvH"
                /locus_tag="SLAV_02735"
                /EC_number="2.3.1.94"
                /inference="ab initio prediction:Prodigal:2.6"
                /inference="similar to AA sequence:UniProtKB:Q03131"
                /codon_start=1
                /transl_table=11
                /product="Strevertene A2 type I PKS, modules 12 and 13"
                /protein_id="ATZ22470.1"
                /translation="MQNQHDDLSGQNENEQKLLDYLKRVTADLKQTRRRLHEVEAKDQ
                EPIAIVAMSCRYPGQVDSPEALWRLVESGSDAVTGFPTDRGWDLDGLYDPDPGTPGKC
                YTREGGFLHEAGRFDPGFFGMSPREALATDPQQRLLLEVAWEAFERAGIAPTSVKGSR
                TGVFVGLAYQGYAGSGDTREELEGFLLTGTAPSVASGRVSYTFGLEGPSLTVDTACSS
                SLVALHLAVQALRQGECTMALAGGAAVMAATGMFTEFSRQQGLAVDGRCKSFAAGADG
                TGWGEGVGMLLVERLSDARRLGHPVLAVVRGSAVNQDGASNGLTAPNGPAQQRVIEQA
                LAGAGLSPADVDVVEAHGTGTRLGDPIEAQALLATYGRGRSAERPLWLGSLKSNIGHT
                QAAAGVGGIIKMVMAMRHGVLPRTLHAEEPSPHVDWSDGTVRLLTEPVAWGEDGRPRR
                AAVSSFGVSGTNAHTIIEEAPPAPEAEEPPSGQDRTDPLTGPAPVAWTLSGRNGAALR
                EQAERLLTHVLGLGDVSPVDVAHSLATSRAALEHRAAVVGTDLPALVAGVRALAEGTA
                AAQVVEGTGGRDGDTAFLFSGQGSQRLGTGSELHASFPVFADAFDTVCSALDPHLERP
                LKGVVFGEDPGLLDRTAYAQPALFAVEVALFRLVESWGVRPDFLAGHSVGEFAAAHVA
                GVLSLDDAARLVAARGRLMQALPAGGVMVAVQASEAEVRDLLAGYEDRAGIAAVNGPS
                AVVVSGAGEAVAALVDRLSADGRKTKALTVSHAFHSPLMDPMLDDFRQILETVTFEAP
                RVPIVSTLTGGRVSAEEFCSVGYWMRHVREAVRFADAVTSLADEGVGTFLEIGPGGVL
                TAMAQAVLEDADAAVIPVLRADRPEGLAVTTALARLHVHGIPVDWTALLAGRGARRVD
                LPTYAFQREQYWLETAPAAGGRQSTIEDWQYEVTWQRLPDPATDPATVTADGHWLLVL
                PAGTDHDGATERDAAGLRDALAAHGCAVTSLTVDAAEDRETLTARLRGATDATPPRAV
                VSLLGLDERPVEGLAALTGGVAATLTLLQALGDAGVGAPLWCATRGAVSTSPDDHPVT
                APGQAQIWGLGRVAALEHPGRWGGLLDLPPHRDGPVPDRILAALTAGGPEDQLAVRAD
                GVFARRLSRAGRNDVRTAGEWRPRGTVLITGGTGALGGHVARLLAARGAEHLVLTSRR
                GPAADGAGELRAELEALGARVTLAACDVSDRDALSRLLTGLTDPAGTDGPLTAVVHTA
                GVDTTALLADTDPAAFAGVLAAKAAAAVHLDELLDALPGGPAELDAFVLFSSIAGVWG
                AGGQAAYSAANAHLDALAAARRARGLPATALAWGPWADAGMAAGPGIAENLRRRGLDP
                LAAASALTVLERAVARNATAVTVADVDWARFAPAFTVGRPSPLLADLPEVREAAVARP
                GHPDGAADTTDALRRALDRADPAERDRTLVELVRAEAASVLGHPGPEAVEPGRAFRDL
                GFDSLTAVELRNRLVKATGLRLPTTMVFDYPSVLDLAGALLVELLGTADTAGSAAAPV
                RADDEPIAIVAMSCRYPGGVRSPEELWRLVDEGTDAMSVFPDNRGWDLDALYHPDPDH
                PGTTYARAGGFLYDLADFDAAFFGISPREATAMDPQQRLLLETSWEAFERAGIDPASV
                RGSRTGVFVGSGYQDYIRRGIAVPEDVEGYLGTGNAASVASGRIAYTLGLEGPAVTVD
                TACSSSLVALHMAAQALRQGECELALAGGVTVMSSSGAFVEFSRQRALSADGRCKAYS
                ADADGTGWGEGVGMLLVERLSDARRNGHRVLAVVRGSAVNQDGASNGLTAPSGRAQQL
                VIRQALANAGLSAADVDAVEGHGTGTRLGDPIEASALLATYGQDRPADRPLWLGSLKS
                NIGHTQAAAGAGGIIKMVQALRHGTLPRTLHAGEPTPHVDWSAGAVRLLTEPIAWDPA
                EHPRRAAVSSFGMSGTNAHVIIEEAPEAGEAPEAPQPGPEEPAPRPPAGAAAVPLPLS
                ARTGAALEQQAGRLAALLREDGTRPRDIGYALATTRTLFDERAVVVGGDREEMVAALE
                ALASGGSHELAVRGSAGSPAQPVFVFPGQGSQWAGMATGLLDTSPVFRDRITACGEAL
                APYVDWSLDDVLRGADGAPALEGPEVIQPVLWAVMVALAELWQACGVRPAAVVGHSQG
                EIAAAVVAGALTLEDGARLVARRSALLARLAGRGGMVSVPRSEAEVTERISAWDGRLA
                VAAVNGPRTVVVAGDPQALAELVDACAADGVQAKRVRVDLASHCAQVEEVREELAAEL
                APLVPNAPRIPFCSTVTGDLLDTAPDAAYWYRNLRRTVRFEQAVRRLRADGHRVFIEI
                SPHPVLVYGLQDTLDDARTDGAAEAVVPTLTRSAGDARRFLASLGEAHAHGVPVDWPA
                VFGGPTGTPAELPTYPFQRQRYWLDAPDAAAGTAPAPQSAADAEFWAAVEREDLASLT
                EELRFDEDTTLAAALPALHAWHRGRRDEAATRAWRYRIDWKPVPDTAQPSLTGRWLAV
                VPRDRFEDPAAELALDALAAHGADTVRLAVTGDEDRQTLADRLREAGPGVRGVLSLLA
                LADHAHPDHPAVPLGLATTLLLVQALGDADVDAPLWCVTRGAVSTRPADPVHAVPQAT
                VAALGRVVALEHPGRWGGLVDLPADVGATATWTGRRLAAALSGATGEDQIALRADGAH
                ARRMVRATDTATDTDSPAGPGWQPRGTVLVTGGTGFIGSRVAHWLAAAGAEHLVLTSR
                RGPAAEGAEELRAELEALGARVTLAACDMADRTAVSALLESLAGLPPLSAVVHAAGIG
                TPALLADTTVAETAEVMGAKAAGAAHLDALLGDRALDAFVLFSSGAAAWGSGAQSAYA
                AANASLDALAEHRRARGLAATSIAWGAWGGGGMVDRAAEERLRLRGLDPMDPGLAVLA
```

```
                     LRRAVDHGDTSVIVADVDWKRFLPGFTAARPSPLLGGLPEVVLLLAAEETGADAESGG
                     EPLLVRRLTGLPATERDAAVLDFVRSEAAAVLGHLSAEAVRERDVFLELGFDSLTAVR
                     LAKRLGGATGLKLPSTLVFDHPTPEAVATRLLALLAEASGGPHGAGPAPRDGQDDLLV
                     GLYRRAGELGRLSEGIGMLTAAARLRPVFDAASAADHRPVPVRLAHGDEGPPLICFGP
                     YMAPSGVHQYARFAAAFGGRRTVWALPEPGFGPGEALPQDVAALVEVHVRAVADCAGE
                     EPVVLVGYSSGGWVAHAVACRLEELGRPVEGIVMLDSFTREEGMADRFQSAVVREQSE
                     RFDFISAPGTQLTAMGGYLAVFEDWDAPVAEAPTLVVRAEERMADGAEGTDDRPPAPE
                     HAQQVTEVPGDHYTLMERHARTTATAVDEWVLKLP"
     gene            complement(12613..23847)
                     /locus_tag="SLAV_02750"
     CDS             complement(12613..23847)
                     /gene="stvG"
                     /locus_tag="SLAV_02750"
                     /EC_number="2.3.1.94"
                     /inference="ab initio prediction:Prodigal:2.6"
                     /inference="similar to AA sequence:UniProtKB:Q03132"
                     /codon_start=1
                     /transl_table=11
                     /product="Strevertene A2 type I PKS, modules 10 and 11"
                     /protein_id="ATZ22471.1"
                     /translation="MTTSNEKIVQALRASLQETERLRRQNRKLTSDSHEPIAIVAMSC
                     RFPGGVTSPEELWQLVADGGDAISGFPADRGWDLDALYDPDPDHAGTCYVRHGGFLDG
                     VGEFDAGFFGISPREALMMDPQQRLLLETAWEAFERAGIDPAALRGSRTGVFAGTNGQ
                     DYARLSTSPEDFDGYLGTGNAAAVISGRLAYTFGFEGPAVTVDTACSSSLVALHLAAQ
                     ALRKDECSLALVSGVTVMSTPGAFMEFSRQRGLAADGRCKPFSAAADGTGWSEGVGML
                     LVERLSDARRNGHRVLAVVRGSAVNQDGASNGLTAPSGPAQQKVIRQALANAGLTGAD
                     VDAVEAHGTGTSLGDPIEAQALLATYGRDRPAGRPLLLGALKSNIGHTQAAAGVAGVI
                     KMVMAMRHGVLPGTLHLDEPSPHVDWSAGAVRLLTEPVDWPEGEGPRRAGVSSFGVSG
                     TNAHVVLEQAAPDTEGAAAPAPVPDSRTPLLWPLSARSAEALPEQARRLLAYVREHPR
                     IPAADIARALATTRAGLPHRAAVVGRDHDELVTALASFARHEATGHAFEGTAARKKKA
                     AFLFSGQGSQRLGMGRELYAALPVFTEAFDAVCSALDAHLERPLREVVFGDDAELLNR
                     TGYAQAALFAVEVALFRLVESWGVRPDLLAGHSVGEFAAAHVAGVFSLDDAAALVAAR
                     GRLMQALPAGGVMVAVEASEEEVRGLLAGYEDRVAIAAVNGPSAVVVSGAEEAVAEVV
                     DRLSADGRKTKRLTVSHAFHSPLMDPMLDAFRKVAEGVTFSEPDIALVSTVTGRPITA
                     AEPADAGYWVRQVVRAVRFADTVAALSDAGASVFLEIGPGGVLTALAQALLDEQPAVT
                     LPVLRPDRPEDLAVASALARLHVHGVTPDWNALLGAPGPVLPDLPTYAFQRRRYWLEG
                     TAFSGQRTETGAADPAEDGFWQAVESADLEAFARRLDVADDAPLSSVLPALTMWRRRN
                     RERSTLDGLRYHTVWKPAATAATEIRLPGTWLVAVPAGHPTDEDPWVASALRALRDHG
                     AEVRTVPVEPGTGRTDLADRLRTALDGTTAGGVLSLLAVDERPHPVHTALPEGLAATL
                     ALVQALGDAAPGASLWCVTRGAVSTGPSDPLRSPLQAQVWALGRTAALEHSDRWGGLL
                     DLPGELDDRAARRFAGALAGAHAAEDQLAVRAAGVLVRRLRRAPRAAAGPDGTTTGWR
                     PRGTVLITGGTGALGAHVARWLADAGAAHLVLTSRRGEAAAGADALRAELEERGAEVT
                     IEACDIADRTAVARLLDGLPGDHPLTAVVHAAGVGTPGMLAETTPERFADVLAAKAAG
                     ARHLDELLGDRPLDAFVLFSSISGVWGAAGQAAYAVANASLDALAEHRRSRGLTATAV
                     AWGPWAGGGMVEDGDAEERLRKRGLPALDPASALAALRGALDRDETAVTVADVAWERF
                     APSFTLLRPSPLIAEVPEAAAALTAAAADPAPGGAGRGEATEFVRRTRTLTGPERARA
                     VLALVRGEAAAVLGHQDTDAVAADRAFRELGFDSLTAVELRNRLNKATGVHLPATLVF
                     DHPSPAVLAAHLLTELTTADTGESSGAARATAAARTSGDDEPVAIVAMSCRYPGDVRT
                     PEDLWRLVAEGRDAIGGFPADRGWDLESLYDPDPDRPGTTYAKDGGFLYDAADFDPQF
                     FGISPREALAMDPQQRLLLETSWEAFERAGLDPTSLRGTSTGVFIGSGYQDYAARLLS
                     VPQDLEGYIGTGSSGSVVSGRIAYSLGLEGPTLTVDTACSSSLVALHLATQALRRGEC
                     DLALAGGVTVMSSPNAFIEFSRQRGLAADGRCKSFAAAADGTGWGEGVGLLLVERLSD
                     ARRNGHRVLAVVRGSAVNQDGASNGLTAPNGPAQQRVIRQALADAGLAAADVDVVEAH
                     GTGTRLGDPIEAQALLATYGQDRPEGRPLWLGSLKSNIGHTQAAAGVGGIIKMVQAMH
                     HGILPGTLHVDAPTPHVDWSTGSVRLLTSAVDWTADGEHGRRAGISSFGVSGTNAHVI
                     IEQAPPQPAAPTGTRPAPGVVPWLVSGTSPAALRDQAERLLAHIEGHPELEPVDVALS
                     LATTRAALEHRAVALGTDRGEFLPALRALAARDTTGPADPALRGTAATDRRTAFLFPG
                     QGSQRLGMGRELYAAFPVFADAFDAVCAHVDQYLERPLAGVVFGEDAALLDRTAYAQP
                     ALFAVEVALFRLVESWGVRPDFLAGHSVGEFAAAHVAGVFSLEDAAALVAARGRLMEA
                     LPAGGVMVAVQASEDEVRDLLSGFEGRAGIAAVNGPSAVVVSGAEDAVTKVVDRLSAD
                     GRRTKRLSVSHAFHSPLMDPMLDDFLKTVETVAFGAPTLPVVSTLTGAPVPAEEFCSA
                     EYWVRHVREAVRFADAVTALADLGVRTFLEVGPGGVLTAMAQDSLDEDAVTSPLLRTD
                     RPEGLAVTTALAHLHVHGTPVDWSAVLAGRGARRVDLPTYAFQRSRYWLDTRPAAGDL
```

ASAGLRTADHALLGAAVALADGEGVVLTGRLSTAAHPWLAEHQVMGAVLLPGTALVDL
AIRAADEAGCDRVDELTLQAPLVLPSRGGVQLQVTVAGPDDTGRRTVRVHSRPDGAGP
DEPWSTHAVGVLATHAPEAAAPARPDPAADLSQWPPAAARPVPTEGYYERLAELGFGY
GPVFQGLRALWQRDGEVFAEVALPQDTTADGFGLHPALLDSALHAVGLGGLLPDTGQG
RIPFAWSGVRLDATGATGLRVRLTSPAPDTVSLLVADATGRPVASVESLVLRAVTADQ
PATARRVEHQDTLYRMDWPALPLGEPGSTPDPTRVADHTGLLALLAAPGGADGLPALP
DTVLIPAPHAPGDATAQDVREVTQHGLELLRTWLGDDRTAHARLVLLTRGAVAPVPGT
TVTDPAQSALWGLVRSAQSENPGRLVLVDTDDPDGHGPSGRALHAALATGEAQFALRD
GAAYVPRLARRSPGDGLRPAPGATAWRLTTGATGTLDDLALTENPAATAPLGRGQVRI
AVRAAGVNFRDALIALGMYPGAATLGSEAAGVVAETGPGVTGLAVGDRVFGMVPEAFG
PLAVADHRMVARIPQGWTFTEAASVPIVFLTAYYALVDLAGLKAGQSLLVHSAAGGVG
MAATQLARHLGAEVYGTAGPGKWAALRADGLDEEHLASSRDLGFEQRFLAATGGRGVD
VVLNSLAGAYVDASLRTLTDGGRFLEMGKTDVRDPERTAAEHRGAAYTAFDTIEAGPE
RIGAMLRELVALFETGALRPLPVTCWDVRRAPDALRHLSQARHVGKLVLTVPAAPDPG
GTVLVTGATGALGGLVARHLVTRHGARHLLLAGRRGPAAEGAAALRDDLLARGAETVT
LAACDAADRDALAALLAGIPADRPLTAVVHAAGVLDDGLVATLTGDRLDAVLRPKADA
ALNLHELTRGLDLSEFVLFSSVAATLGSAGQGNYAAANAFLDALAQRRAAAGLPATAI
AWGPWADSGMASGMDERGRGRMARSGLVSFRAEEGLALFDAARTGEDAVAVPVRFDTT
APATGPDPAGEVPAVLRGLVRRTVRRTAREAVAAEGADALRERLGALSDPERDGVLLD
LVRTQVAAVLGIGGPRDVDRNREFKQLGFDSLTSVELRNRLNAATGLRLPATLVFDNP
TPVALAERIRLDLVPDPAPGASALAVFGELDRLETALAAVGADDDLVRSRIRARLHGV
LAALGEGTAADGSGGGSSPDEASDERLRSATVDDIFALIDQELDGA"

gene complement(23874..39131)
/locus_tag="SLAV_02760"

CDS complement(23874..39131)
/gene="stvF"
/locus_tag="SLAV_02760"
/EC_number="2.3.1.94"
/inference="ab initio prediction:Prodigal:2.6"
/inference="similar to AA sequence:UniProtKB:Q03131"
/codon_start=1
/transl_table=11
/product="Strevertene A2 type I PKS, modules 17, 8 and 9"
/protein_id="ATZ22472.1"
/translation="MTTDVNKLRDYLKRATVDLRHAHRRLREAEGKNREPIAIVGMSC
RFPGGVRSPEDLWDLVASGRDAISGFPTDRGWDLDALYDPDPENPGTSYAKEGGFLYD
AAEFDPAFFGIGPREALAMDPQQRLVLEASWEAVERAELDPLSLRGSRTGVFAGVMYH
DYVSRLPAMPEGIEGYVSTGNTGSVVSGRIAYTLGLEGPAVTIDTACSSSLVALHLAV
QALRQGECDLALAGGVTVMAGPTTFVEFSRQRGLSPDGRCRAFSASAEGTGWSEGVGM
LLVERLSDAVRLGHPVLAVVRGSAVNQDGASNGLTAPNGPSQQRVIHQALANALLSPS
DVDIVEAHGTGTTLGDPIEAQALLATYGQDRPADRPLWLGSVKSNLGHTQAAAGVAGI
IKMVQALRHGVLPKTLHAEEPTPHVDWSAGAVSLVTENAAWPDTGRPRRAGVSAFGVS
GTNAHIVVEQHLAPAARPEAAAMDDAGPDAPSVAAPAADGPSGAPGTATADGNGLPLV
PWTLSGKTAAALTAQAARLRSHLAAPRPGPDPATDPAAGPAAYPAAGPAAYPAAGPAA
YPAAGPAAYPAAGPAADPDAGPAADPDALDIAYSLATTRTAFPHRAVLLAPDRAGLDR
ALDALADGEPDAAGAALVRGTSDATRQVAFVFPGQGSQWAGMAVELLDAAPVFRERFR
QCEEALAPHVDWSPTEVLRGAPGAPGFDRVDVVQPVLFAVMVSLAELWRSHGVHPSAV
AGHSQGEIAAACVVGALSLADAAKVVALRSQALTALSGRGGMLSVALPLEDLTPRMAH
WGERLSVAAVNSPTSIVVSGDRDALTELRDALHADDVRARLIAVDYASHSAHVEAVRE
RVLDALAGITPQACDVPFYSTVTGTVVETDGLDAEYWYRSLRRTVRFEEATRALVRDG
HDALIEVSAHPVLTIGIQETLDDLGAGAVTLGTLRRGEGGPGRFLRSAAEAHAHGVAV
DWAAVLSGTGARRVALPTYAFQHERYWLDAPPAAADATGLGLASSDHPLLGAVVSLAD
SDGLLLTGRLAAHTHPWLADHVVLGAVVLPGTAFVELAVRAGDHVGCDRLAELTLQAP
LVLPERGGVVVQVAVGPAEGTGERTFTIHSRPDTAAPEDGWTCHGTGVLTSAAEDRPP
VPAPAAWPPAGAAPVDLGGFYQELAARSFAYGPAFQGLRAAWRLGDEVFAEAVLPPDA
SAPSDAARYGLHPALLDAALHGVGFGPLGDMGTGRMAFSWEDVRLYATGATRLRIRLT
PVGAEAVAVTATDDSGRAVATVAALTFREVREEHLRAALTDHHESLYRVDWPTLPPAG
TAPDDSTHWAVVGVTPRADADAVADALRGAGPRPDVHPDLAALAAAITEGAPVPRTVA
AVVPVAASDASDASDADTVRATARATLALVQAWLADERFVSSRLVVLTRDAVPVPSGA
EDRVPRTRPAHAPVWGLIRSAQSEHADRLVLADTDGEPESLRRLAAAVATGEPQLALR
EGRMSVPRLARVPVTAGTAPARALDPHGTALITGGTGALGRLVAAHLVTAHGVRNIVL
TGRRGPDADGADALREELAALGATVTIAACDAADREALAALLATLPADRPLTAVVHAA
AVVAGGLVDTLTPEELHQVLRPKVDAALNLHALTRDLDLSAFVLFSSFAGTLGGAGQA

```
                     AYAAGNAFLDALAHQRRAQGLPASSLAWGVWDERGDQTRLETGDLRRMARAGLVPLGA
                     EEGLRLLDTARTLDDAALAPVRLDLAAMRAQAAAHPVPHLLRGLIRTPARRSAETAPG
                     PGGHSAELSRLAALPPAERRDTVLALVRNQVAAVLGHSAPEAIDPGRAFRELGFDSLA
                     AMELRNRLATATGVRLPATAVFDHPTPAALAGHLLDEIPGGHPGTALVTAAPDASTTP
                     GRLPGTDTDEPIAIVAMSCRLPGEVRGPEDLWDLVANGTDAITGLPVNRGWDLDALYD
                     PDPDHPGTSYVREGGFLHDAGDFDPEFFGISPREALSMDPQQRLLLETAWEAFERAGI
                     DPAALRGSRTGVFAGTNGQDYAVGLSQHRTEDMEGHLLTANSASVVSGRLSYTFGLEG
                     PAVTVDTACSSSLVALHLAAQSLRRGECDLALAGGVTVMSTPGALIGFSRQRGLSHDA
                     RCRAFSAAADGTGLAEGAGMLLVERLSDARRNGHRVLAVVRGSAINQDGASNGLTAPN
                     GPAQQRVVRQALTDAGLTAADVDAVEAHGTGTTLGDPIEAQALLKSYGNDRPADRPLW
                     LGTVKSNIGHTQAAAGVAGVIKMVMAMRHGVLPRTLHVDEPSPHVDWSAGAVRLLTEP
                     VEWAEHGRPRRAGVSSFGVSGTNAHVIIEQAPAEGADGTNHGPGEETGPRPAVPWLIS
                     AKDETGLRAQAERLLGHVRSEAHGTDETDIAFSLATTRAAFPHRAAVIGADRELLVQG
                     LTALAHGEPSGTETVRGTATASGSRTAFLFSGQGSQRPGMGRELYAAFPVFAGAFDAV
                     CAALDAHLERPLKEVVFGGDAGLLNQTGFAQPALFAVEVALFRLVESWGVRPDFLAGH
                     SVGEFAAAHVAGVFSLEDAAALVAARGRLMQALPSGGVMVAVQASEEEVRELLSGYED
                     RAGVAAVNGPSSVVVSGAEEAVAALVDRLSADGRKTKRLTVSHAFHSPLMDPMLDDFR
                     KVAEGVSFGDPTVPLVSTLTGAPVPAEEFCSADYWVRHVREAVRFADAVTSLADLGVR
                     TFLEVGPGGALTSMAQDSLDEHAVSVPLLRTDRAEDLAVTTALARLHVHGVPVDWTGV
                     FAGRGARRTDLPTYAFRRTPYWLLPADPQETGPLSADPADAGFWEAVESQDLASLTEQ
                     LDLDGDSPLSSVLPALSRWRRRQRDASVVDALRYRTSWKPLTGREAAELTGAWLLAVP
                     AGAEDAPTVAAVTAALSRQGAEPVPLVVRADEDRAALAARLRAQPGDTAPAGVLSLLA
                     LAEEPHPVHKELPTGLAQTTLLVQALGDAEISAPLWCVTTGAVTVGRSDPLTSPGQGM
                     VWGLGRSVALEFPERWGGLVDLAGAPDERTARRLASVLSAPAHASAAPADDQIAIRPS
                     GIFVRRLARALTGAVPADGAWQPRGTVLITGGTGALGARVARYLAARGAEHVVLTGRR
                     GPDAPGAAQIAEEIEALGAEVTLVACDVSDREEVAALLRSVPEDRHPLTAVVHAAGMP
                     QFAPTDSLDLGDFAEVVSAKVAGASHLDELLGDRPLDAFVLFSSVAGVWGSGRQAAYA
                     AANAFLDGLAERRRADGLAATSVAWGPWADGGMADDDEAEAHLRRRGLPPMSPDTAIA
                     ALQRALNHDETTVVVADVDWDRFAPAFTIARPAPLLADLPEARQALAGTGSATGGDGR
                     PDTTAPSLGAGLAALAEPERRRALLDLVRTEVARALGHPGRDAVASDRSFKDLGFDSL
                     TAVELRNRLNAATGLRLPTTLVFDHPNADALADHLTSELPGHGAATVRADAATPGERP
                     AATPDEPIAIVAMSCRFPGGAHSPEQLWQLLVDGVDTVSAFPADRGWNLDTLYDPDPE
                     HPGTTYVNEGAFLYDAGHFDSTFFGISPREALAMDPQQRLLLEAAWEALERARLAPSS
                     VRGTRTGVFIGSGYQGYGAGPGDLPDGVEGHLLTGASSSVMSGRISYALGLEGPAMTV
                     DTACSSSLVALHLATQALRQGECELALVGGAAVMSSPSAFVEFSRQRGLAADGRCKPF
                     AAAADGTGWGEGVGMLLVERLSDAQRNGHPVLAVVRGSAVNQDGASNGLTAPNGPAQQ
                     RVIRQALANAGLSTADVDAVEAHGTGTALGDPIEAQALLATYGQDRPDDRPLWLGSLK
                     SNIGHTQAASGVASVIKTVMALRHGMLPKTLHVDEPSPHVDWSAGAVRLLTEPVTWTG
                     NGRPRRAAVSSFGVSGTNAHTVIEEAPATVPTEEGEHGTPAAGHCLPWTLSGKDEASL
                     RAQARNLLAHTSDHPNLAPADLALSLAESRTAMTHRAVVLGRTTAELAAGLEALSKGE
                     TATDLVRGVARPADRRTAFLFPGQGSQRLGMGRELYAAFPVFADAFDAVCAHVDQYLE
                     RPLAGVVFGEDAALLDRTGYAQPALFAVEVALFRLVESWGVRPDALAGHSVGEFAAAH
                     VAGVFSLEDAAALVAARGRLMEALPAGGVMVAVQASEGEVRDLLSGFEDRAGIAAVNG
                     PSAVVVSGAQDAVTKVADRLAADGRKTKALSVSHAFHSPLMDPMLDDFRKVAEGVSFG
                     VPTLPVVSTLTGAPVPAEEFCSAEYWVRHVREAVRFADAVTSLADLGVRTFLEIGPGG
                     VLTAMAQAALEDGPAVVPVLRADRPEEVAVTTALARLHVHGTPVDWSAVLAGRGARRV
                     DLPTYAFQREHYWLETAIAPAAPAHQPSGSDNPAETEFWEAVENGRPEALAERLGLAE
                     GAPLDSVFDALATWREESRNRSTLDGWRYHTVWQPLADPSSGPLPGSWLVVAPDAGAA
                     EPYARTLRDRGAEPLCVTVADPAADRSALVELLRDAVAEHSGPVSGVLSLLALDERSH
                     PTHPGLPAGLTATVALVQALGELEPDAPLWCVTAGAVSVSGAQPVTRPLQSLVWGLGR
                     AVALETPQRWGGLVDLPTAPDGRALARLADVLADAAGEDQLAVRASGVLVRRLARMPR
                     ATGGTDGAWKPRGTVLITGGTGSLGGHTARWLARGGAEHLVLTSRRGEAAEGALELAA
                     ELGALGARTTIAACDVADRAALAALLDGLADDPAPLTAVVHAAGLPQFSPVADTSLTE
                     LAAVVSAKVAGAVHLDELLADRELDAFVLFSSVSGVWGSGSQAAYSAGNAFLDALAQH
                     RRARGLAGTAVAWGPWAEGGMAADGGAEEYLRRSGLPSLPPALAVSALQLAMDRDETT
                     VVVADVDWPRFAPSFTIGRPSALLAALPEARAALDSGGADGGPGGDRGEGAASDLVRR
                     LPAGAPEDRHELLLELVRKEAAAVLGHPRTDAVEPDRAFRDLGFDSLTAVELRNRIGT
                     ATGLRLPMTMVFDHPTPTALTDRLLGDLFPGGADGPHPDAGPDEARVRQTLASIPLSR
                     LREAGLMDALLALAPGAPGGVATDTGTDGDADSAIAEMDLENLIELALGDSEA"
     gene            complement(39175..50097)
                     /locus_tag="SLAV_02775"
     CDS             complement(39175..50097)
                     /gene="stvE"
```

/locus_tag="SLAV_02775"
/EC_number="2.3.1.41"
/inference="ab initio prediction:Prodigal:2.6"
/inference="similar to AA sequence:UniProtKB:B2HIL7"
/codon_start=1
/transl_table=11
/product="Strevertene A2 type I PKS, modules 5 and 6"
/protein_id="ATZ22473.1"
/translation="MSDLQANNNEEKLRYFLKRVSADLHEARERLRDLDAAQHEPIAI
VAMSCRFPGGITSPEGLWQFLTDGGDAITEFPADRGWDLESLYDPDPDTRGTSHVREG
GFLDGIDLFDAGLFEISPREALTMDPQQRILLESCWETFERAGIDPTSLKGSRTGVFT
GVMYHDHTSVLQQAVEDVDGYIGTGSAASVVSGRVSYTFGLEGPAVTVDTACSSSLVA
LHLAVQALRRSECDLALAGGVTAMLTPTSFVDFSRQRGLAADGRCKPFAAAADGTGWS
EGLGMLLVERLSDARRNGHPVLAVVRGSAVNQDGASNGLTAPNGPSQQRVIRRALTDA
GLSATEVDAVEAHGTGTRLGDPIEAQALLATYGRGRSAERPLWLGSLKSNIGHTQAAA
GVGGIIKMVEAMRHGVLPRTLHVDAPTPHVDWSSTTVRLLTEPVEWAEHGDRPRRAAV
SSFGFSGTNAHVVIEQPPRPEPGPADGESAPAQDPARATPVLLSAKNEAALRGQAARL
LRHTEEHPDQSLADLGLSTATTRAALDHRAAIVASDREDLRRALERLAQDAPTAGAGS
GGLVTGVVAPRQTVAFLFSGQGSQRPGMGRELYAAFTVFAEAFDDVCAALAPHLERPV
KDVVFGDDAELLNQTGYTQPALFAVEVALFRLVESWGVRPDLLAGHSVGEFAAAHVAG
VFSLEDAAALVAARGRLMQELPPAGVMVAVEASEEEVRDLLAGFEDRAGIAAVNGPSA
VVVSGASDAVAAVVDRLAADGRRTKRLSVSHAFHSPLMDPMLDGFRKVAEGVSFKAPT
VPVVSTLTGAPVSGEEFCSVDYWVRHVREAVRFADAVTALAGEGVSTFLEIGPGGVLT
AMAQTVLEDADAAVIPVLRADRDAEEVAVTTALARLHVHGIPVDWTAVLAGRGARRVD
LPTYAFQREHYRLETRPAAGDLTRTGLRPADHALLGAVVTLADGEGVLLTGRVSLATH
PWLADHVVLGAALLPGTALVELALRAGDQVGYGRIEELTLEAPLLLPEQGGAQLQVVV
DGPDTNGARAVRVYARHDDAMEADAWTCHASGLLSAGTGAAPGLAEWPPAGAEPLDAG
ALYDRMAGLGLEYGPVFRGVRAGWRRGEELFAEIALPDGTDVTGFGLHPALFDAALHT
VGLGDPAAPADGPRIPFAWSGVTLHATGATALRLRLAPAPSSATAAGEGAVSLSLADA
TGSAVLSVDSLDLRPVAAEQLAAGGSGAGGLFRLEWKPAGDLLTAPAGELPVAVLDAL
PRPPAAEGTPVPDVVAVRLPDPTGADAAARPAVAAQAALALVQDWLADERWAASRLVV
LTRGAVAVDPSSAADPAQAAVWGLVRAAQSENPGRLVLLDLDEDPGEEDPAALAAAAA
TGEPQVAVRGKALFAPRLVRAADAPAGGGAPGPAVFAPSGTVLVTGASGALGRVVARH
LVTRHGVRRLVLASRRGPAADGAREFAAELGALGAAVETVACDVADREALVRLLDSLP
DAHPLTGVVHAAGVTDDGVVPSLTPERVRRVFDPKVAGALHLDELTRGLDLSAFVLFS
SAAGVFGNAGQGNYAAANAALDALAHTRRAAGLPGLSLAWGLWASTGTMTAGLAEADH
RRIGRSGLVALETEEGLGLLDRALGSPEPLLVPVRWDLTVLREQARTGAPHPLLRDLV
PAPVRRTEAAAAGSADAARSALSERLRGLTEAARTDLLVDLVRGHAAAVLGHDRPATV
QATQAFRELGFDSLTAVELRNRLVSATGARLPAGLVFDYPTPDALARHLLGQLDGARP
AGTVRAATRAGGAADSDEPLAIVAMSCRYPGGVRSPEDLWRLLATGTDAISDFPVDRG
WDLDLLAADGATGSSTTRRGGFLYDAGEFDAAFFGISPREALAMDPQQRLLLETSWEA
FERAGIDPATLRGSRTGVFTGVMYHDYASALRSVPEGVEGFLGTGNSGSVISGRLSYT
FGLEGPAVTVDTACSSSLVALHLAVQALRQGECELALAGGVTVMAGPGAFVEFSRQRG
LAADGRCKAFSANADGTGWAEGVGMLLVERLSDARRLGHPVLAVVRGSAVNQDGASNG
LTAPNGPAQQRVIEQALAGAGLSPADVDVVEAHGTGTRLGDPIEAQALLATYGRGRSA
ERPLWLGSLKSNIGHTQAAAGVGGIIKMVEAMRHGVLPRTLHAEEPSPHVDWSDGTVR
LLTEPVAWGEDGRPRRAAVSSFGFSGTNAHVVLEQAPAVSRTAPAARTKPAPPFTAPW
LLSAKSPEALREQARRLLEHAREHVPERDGDREAAEIAYSLATSRTALEHRAAVVAPD
TAGVLEGLAALAHGDPARSVVRAVAEPGRKRAFLFSGQGSQRLGMGRELHAAFPVFAE
AFDEACAALDPHLEHPLRTVVFGDDAELLDRTDHAQPALFAVEVAAYRLVESWGVRPD
LLAGHSVGEFAAAHVAGALSLEDAAALVAARGRLMQALPFGGVMAAVQASEEEVRELL
AGHPEQADVAAVNGPSSMVVSGTRDAVSAVVERLEAQGRKTKPLSVSHAFHSPLMDPM
LDDFRQVVETVTFAAPRLPVVSTLTGALVPAEEFCSVGYWVRHVREAVRFADAVSALA
DVGVGTFLEIGPGGVLTAMAQDSLDEHAVTVPLLRTDRPEGLAVTTALAHLHAHGTPV
DWTAVFAGHGLRRVDLPTYPFQRTRYWLESAPASGNVAAAGLAEAGHALLGAAVPLAD
GDGLVLTGRLSPATHPWLADHAVMGAVLLPGTALVDLARRAALDEAVGCDRLDELTLG
APLVLPDDTAVRIQVRVGSPDGAGRRPLEIHSRTEDAPADEPWTRHAAGTLSTAEATA
DATADAAALADWPPSEAVAVPEEALDSLYGRMAGLGLDYGPVFRGVRAAWTREDALFA
EVELPEGTDVSGFGLHPALLDAALHTVALDPGLLGPDADAGDRARIPFSWSGVTAHPA
PSAGAVEALRIRLTPAGPDTVSLLAADPTGRIRTSVDSLVLRPVSAEQLAAARAGGSR
DSLFRVDWTPLPLPRTPDAPPAGDRVVLFATDGPSEAGAVRSVAGRALALVQEWLADA
EAEADADTGTDARGLVIVTRNAVAATAGDAARLDPAQAAVWGLVRSVQAEHPERFVLV
DADHADRAQPEGLTAVLATGEPQSALRNGRAFVPRLARATGAGAPGPTPSLGLDPAGT

VLVTGATGALGRLVARHLVAAHGVRHLVLTSRRGPDADGAGAFAAELAALGAEVATLA
CDAADRDALAAVLHGIPAAHPLTAVVHAAGVLDDAPVGSLTARRLDDVLGPKADAALH
LHELTRDADLAAFVLFSSLAGTFGNAGQANYGAANAFLDALAQSRRAAGLPAVSLAWG
PWAEAGGMRGMLGGAGAGALARMARAGVSPLSPEEGLALFDTASAPPTAETPTAGTAA
DTPGTAPDAVLLPVRLNLPALRRRATTEPVHALLHGLVRAPARPAREPTPGTAALAGQ
LAGASEEKRRRILLGVVRTQVATVLGHASSEAIGAAQAFRDLGFDSLTAVELRNHLGA
ITGLRLPASLLFDYPTPADLAGHLSAALPAAGPGGAEPAPSLLSEIDRLEAAFARSPY
DRGTRATAALRLEVLLAKWREAAHDTGHGPGDDDSGTEVEEASDEELFELLDGELGTH
"

```
gene            complement(50191..73599)
                /locus_tag="SLAV_02776"
CDS             complement(50191..73599)
                /gene="stvD"
                /locus_tag="SLAV_02776"
                /codon_start=1
                /transl_table=11
                /product="Strevertene A2 type I PKS, loading module and
                modules 1, 2, 3, and 4"
                /translation="MGESVSVPEESGTSTHDVIVVTGSAHRPPGGPEADPAFDAEFLA
```

DATAGPSDPRLTGALPHGPAAAEVWWQALEDAGVVLAAEPAAGTAVFLAAPDADALPQ
DGDPAAAVRAVLRTAPGADPEVLSTPSGTDALRAAHERLRRGETTLALAAGRDIRLPD
TDPAPADFPSGAAGSGAAGPEAAGSEPAGADPGAGGCVGGYGAVVLKTLARALADGDR
VRAVLSADGRLTAPGADLTEDTGPAGSGADLTADAGPAGSGAGPVEHPLPWILSAAGP
EALRAQAERLHAHLSAHPGLDPAAVGRALAVTRHRFPHRAVVLGEDTAQLLDALATLA
RGGFAAEIVRGSAGPAATGPTAFVFPGIGSQWPGMAVELLDSSPQFHAAALECQEALA
PLTGWLLTDVLRGEPGAPALDTPDVSMPATFAVQVALARTWQAYGVCPAAFAGHSIGD
IAAAHLSGALTLRDAARVITSWGDGLARLAARGGDMLAVTLPGHRLADRLRPWEGRLD
VAAYNGPESVVVSGDTDAVRELRAALEADGVRCRGVAVGAAAHSSHVEDLRDRMTAEL
ASIAPRATHTPLYSSATGGIVDGRTLDAAFWFEALRRPVRFEQTTHALLADGHQALIE
VNPHASLTAAMQETASAAGRQPVTVTTLRRDQGGPRRFRTALAEAYVQGVHIDWERAF
PGAPAARPPLPTYPFRRTAAADGPALSERLRALPEAERERTVGALVRSVTAALLGLSD
PAGVGAGHAFRDLGLDSVTAIELRNRLNEATGLALPHTVVFDHPTPRSLTGRIVADAL
GTRDEAADDTTTAAWDEPVAIVAMSCRLPGGIDTPEALWHVLTEGGDAVSALPADRGW
DVEGLYDPEPGRPGRYYQREAALLHGAADFDPEFFGISPREALAMDPQQRLLLETGWE
VLERAGIDPGTLRGTRTGTFVGAMTQDYGPRLYEAPEGVSGYLLTGNTASVASGRLAY
TFGFEGPAVTVDTACSSSLVALHLAVQSLRTGECSLALAGGVTVLPSAGSFIEFSKQR
ALAPDGRSKAFSSDADGFGLAEGTGMVLLERLSDARRNGHRVLAVVRGSAINQDGASN
GLTAPSGPAQQRVIRQALANAGLSAADVDAVEAHGTGTRLGDPIEAQAILATYGRDRT
EDRPLWLGSLKSNIGHTQAAAGIAGVLKMVLALQHGVLPKTLHADVPSPHIDWADGRV
RLLGEAADWPDTGRPRRSGVSSFGISGTNAHAILEEAPDAHEAADAPTADAPTAPAAG
TPAADATPVPWPLSARTAEALAAQARTLLAHTLTHPGTAPADTGHSLATTRAAFEHRA
VVVGSTEAELTEGLRALAEGGSTPRAVQGTATVGRRVVFVFPGQGSQWAGMAVELLDG
SAVFRERLRACGEALAPYVDWSLEDVLRGAPGAPALDSADDVVQPALWAVMVSLAELW
RSFGVEPAAVIGHSQGEIAAAAVSGALGLDDAARVVALRSRLLSRLAGLGGMVSVPEP
LADVTRRLEAWDDRLGIAAVNGPRSIVVSGDADALDELLAACTADGVRAKRVPVDYAS
HSAHVEIIEAELAEALAGITPRPPRIPFCSTVTGEVVDSADLGAEYWYTNLRTTVRFE
PAVRRLLESGHDVFVECSPHPVLAVGLQETAESAGVTADVVPSLRRGDGGPARFLTSL
ADAYVHGVPTDWSAAFAGLRPRTVALPTYPFQRRRFWLDAGAPTAQAGAAAGHSEEEA
RFWEAVENADVRALTSSLDLAPDASLDSMVNRLAHWRRRTTESALIDGWRYRVTWTPT
ATQARRGNRTPLSGTWLLVTPVDRHGGAHAATAAVHEALTARGARVERVVLDPAADSD
RETVARLLTDGLGGAGLEPDGVSGVLSLLAGTGAATGRAGDAVPGTPPAPRVLPALAA
TLALVQALGDTGIGAPLWCATSGAVATGPADPPRRPEQAQLWGLGRVVGAESPQRWGG
LVDLPETLDERTLEHLTALLADPDGEEEIAVRATGTLARRLVRADTDTTAPTTAATPD
HVLARPHGTVLITGGTGTLGAHVARWLARSGAAHLVLTSRSGPAAPGAAELRDELAAL
GTRTTIAACDVTDREDLGRLLDSLPGEHPLTGVIHAAGVLDDGVLDAMTTERLEHVLL
PKVDAALHLHELTAGLDLDFFVLFSSIAGVVGNGGQGGYAAGNAFLDALAHQRRAQGL
PATAIAWGSWGSGRMMGDLAEQHLTRRGILPMPADLGIAALRRAMEHHDTAVTLADID
WDRFVPAFAISGDYPLLRRLPEARRILDAAATPATGAAGGAGGPVLAQRLTGLSGTER
LRAVVELVRTQAATVLGHSGVEAVPPRRAFRDTGFDSLTAIELRNRLAGATGMRLPTT
VVFDYPNPTELAGYLHDRIAGSPGAAGHDDGHPGTPLAPPADDEPIAIVAMSCRFPGG
VTSPEELWEILHEGRDVLSGFPENRGWPLESLYHPDPQNPGTTYAREGGFLYDAGDFD
AGFFGISPREALAMDPQQRLLLEVTWEAFERAGIDPATLKGTRSGVFIGSNGQDYASG
LRKAPEGVEGYLLTGRAASVVSGRLAYTFGLEGPALTVDTACSSSLVALHLAVQSLRL

GECELALAGGVTVMANPGIFVEFSRQRGLAADGRCKAFAAAADGTGWGEGTGMLLLER
LSDARRNGHRVLAVVRGSAINQDGASNGLTAPNGPAQQRVIRQALANAGLSATDVDVV
EAHGTGTRLGDPIEAQALLATYGQDRPEGRPLWLGSLKSNIGHTQAAAGVAGVMKLVL
SLQHGTVPRTLHVDAPTPHVDWTTGAVELLTEPVTWPDDHEHVRRGGVSAFGVSGTNA
HVIVEEAPEPDPEPEDTATAATPATPVAAVPWLLSAKDATALRVQADRLRSHLADHPE
AAPADIGLSLATGRSALQRRAAVVGTDRDELLRGLAALADAGPAGSSSHVITGATDSG
GDDEGSRTAFLFSGQGSQRLGMGRELYAAFPVFADAFDAVCAHVDQYLERPLAGVVFG
EDAALLDRTAYAQPALFAVEVALFRLVESWGVQPDFLAGHSVGEFAAAHVAGVFSLED
AAALVAARGRLMQALPSGGVMVAVQASEAEVRDLLSGFEDRAGIAAVNGPSAVVVSGA
QDAVAAVVDRLAADGRKTKALTVSHAFHSPLMDPMLDDFRAVVGGVTFGAPRLPLVST
LTGAPATAEELSSADYWVRHVREAVRFADAVTSLADLGVRTFLEVGPGGVLTAMAQDS
LDGHAVAVPLLRADRAEGTAVTTALAHLHVHGVPVDWTTFFTGSGARRVDLPTYAFQR
EHYWLHVPATADVTAIGVADTEHPVLGATVLLPDGAIVLTGRLSLATHPWIADHAVSG
VVLVPGTAFVELALRAGREAGCEQIEELTLEAPLVLPEHGGVQLRLTVGTRDASGRCA
LELYSRPEDAPGTRDWTRHAAGTLSAGPPAPASSELVAWPPQGAERVATDDLYDHLDD
LGFGYGPVFRGLTEAWRHGDTLYAAAALPEESAPDAGAYGLHPALLDAGLHASWLGLL
SGSETGNGLLPFSWSAVHLHAAGATAVRIKLAAAGTDAVSVLVADGTGRPLATIGALV
LRPVSADRLRLAAEPRHDSLYRLDWTPAHAPADGPVPEFDVATPGRPDTGADDPADDT
AARVRTTSHRALRLIQQRIADDAADPAPLVLVTRHAVATDAAERGALDPAQAAVWGMV
RSAQSENPGRFVLLDLDPDLDPDGDLGRAAVRAAVSTGEPQLAVRAEAILVPRLTRTS
SAAEEGGAGSGPGRTFPDGGTVLVTGATGTLGRLVARHLVTRHGVRHLVLTGRRGPGA
EGAAGFEAELTALGARVVTAACDVADREALAALLATIPAAHPLTAVVHAAGVTDDGIV
PALTPERVDHVLRPKVDAALNLHELTRDADLSAFILFSSVSATLGGAGQANYAAANSF
LDALAQLRRAAGLPAVSMAWGLWAEGSGMTGKLDSADLARIRRMGLAAMDSATGLALF
DAACAAGDDVLFPVPLDHAGLRVQAAGDKVPALLRGLVKAPVRKARAAGDATATGAAD
RATALAASLHGLPAAEQERVLGDLVRLQIATVLGHASPERIEPERQFRDLGFDSLTAV
ELRNLLGSVTGTRLPATLVFDHPTPLALTRFLRGEILGGEADAGPPGGVPSVSVDEPI
AIVGMSCRYPGGVRSPEDLWRLVAEGGDAVGGFPVDRGWALDSLYHPDPDHVGTSYAR
EGGFLYDAAEFDAGFFGISPREALAMDPQQRLLLETSWEVFERAGIDPASLRGSRTGV
FAGVMYHDYGSRLSAVPDGFEGYLVNGSAGSIASGRVAYTFGLEGPAVTVDTACSSSL
VALHLAAQSLRQGECSMALVGGVTVMATPNTFVEFSRQRGLAADGRCKAFSAAADGTG
WGEGTGMLLLERLSDARRNGHRVLAVVRGSAINQDGASNGLTAPNGPAQQRVIRQALA
NAGLSATDVDVVEAHGTGTRLGDPIEAQALLATYGQDRPEGRPLWLGSLKSNIGHTQA
AAGVAGVIKMVEAMRHGVLPRTLHADEPSPHIDWSAGAVELLTEAREWAQSGRPRRAG
VSSFGVSGTNAHVVIEQPEQPEQSERQPRADGPTNTPTAGTATATAVAWPLSAKDPAA
LREQAARLLTHLMEHPDLGTADIGLSLAASRAALDLRSAAVGTGREELMAALGALAEG
RPAPGALARDETSTGTPAGGGRTAFLFSGQGSQRLGAGRELYAAFPVFAEAFDAACAA
LDAHLERPVRDVVFGENPELLHRTAHAQPALFALEVALFRLVESWGVRPDFLAGHSVG
EFAAAHVAGVLSLEDAAALVAARGRLMEALPSGGVMVAVQASEAEVRDLLSGFEDRAG
IAAVNGPSAVVVSGAEDAVAAVVDRLAADGRKTKALTVSHAFHSPLMDPMLDDFRAVV
GGVTFGAPRLPLVSTLTGAPATVEELSSADYWVRHVREAVRFADAVTSLAHLGVRTFL
EVGPGGVLTAMAQDSLDEHAVTVPLLRTDRAEDLAVTTALAHLHVHGVPVDWTTFFTG
SGARRVDLPTYAFQYEHYWLDVPTAPGDVAAAGLAAAGHALFGAAALLPDGAGSLLTG
RLSLATHPWLADHAISGLTLLPGTAFLEMAVRAGDELDCGRVDELTLAAPLVLPEHGA
VQIRVVVGAADEADRRTVRIYSRTDANDEAATELPWTRHATGFLVPDTGAAHAPDVPG
PSGTAEAWPPAGSTPVDVHDCYGRLADAGFAYGPAFQGLRAVWRRGEDELFAEVGLPD
GTSVDGFAVHPALLDAALHAMVVGDVPAIGHEGLLPFAWSGVSVHGTGAETLRVRLTR
ADSAADSVSLAITDGLGRPVASVDSLTLRAVTPGQLAGEGGGQAASTLRDSLFRVDWE
PCDPAPGAEHVGVLVCEGLPDAQATQAPAPHTVAVTVPGNAHGSADTSAAATAQRVLA
LIQEWLAEPRFAESRLVIVTRGAVNTGTAADVAGVDPAQASVWGLVRSAQSENPGRFV
LVDLADGAETLAEAPLAAAVASGEPQVAVRDGALLAPRLTRIDATSTERPADADADTT
GLRPAFSAGGTVLVTGASGVLGRLVARHLVTEHGVRHLLLASRRGPDAEGAPEFAEEL
GALGAEVRAVRCDVADRTALAGLLAAVPADRPLTGVVHAAGVTDDGIVSALTPERVDH
VFRPKADAALNLHELTRDADLTAFVLFSSAAGTLGSAGQANYAAANAYLDALAETRRA
AGLPAVSIAWGLWAETSALTADLAAADRRRISRSGLVALEAQEGLALFDAACRSALAT
VVPVRLDTTALRAHARAGALPPLFRQLVRGSVRRALHGAPQNATGSSPADRIAALPPA
DRYDALLALVRTQAATVLGHESVRSVAVDRQFRDLGFDSLNAVELRNSLNTATGLRLP
ATLVFDHPTPEALTRHLLTELVGTRSGTPEHAPAAVVSVDEPIAIVGMSCRYPGGVRS
PEDLWRLVAEGGDAVGGFPVDRGWALDSLYHPDPDHVGTSYAREGGFLYDAAEFDAGF
FGISPREALAMDPQQRLLLETSWEVFERAGIDPASLRGSRTGVFAGVMYHDYGSQSGG
AVPEGLEGHLGMGTSGSVASGRLSYTFGLEGPAVTVDTACSSSLVALHLAAQSLRQGE
CSMALVGGVTVMATPNTFVEFSRQRGLAADGRCKAFSAAADGTGWSEGVGMLLVERLS
DARRNGHRVLAVVRGSAVNQDGASNGLTAPNGPAQQRVIRQALAQAGLSASEVDAVEA

```
                     HGTGTALGDPIEAQALLATYGQDRPEGRPLWLGSLKSNIGHTQAAAGVAGVIKMVEAM
                     RHGVLPRTLHADEPSPHIDWSAGAVELLTEAREWAQSGRPRRSAVSSFGVSGTNAHVV
                     LEHVPGTEQATAVEDAPAPVAVPWVLSAKDEVSLRAQAQRIAAHLDAHPALGVAEVGR
                     SLATSRAAMEVRAAVVGTRREELLEGLTALAAGRPAAGVVHGEPEPGGSRTAFLFSGQ
                     GSQRPGMGRELYAAFPVFAGAFDAVCAALDAHLERPLKDVVFGGDAGLLNQTGFAQPA
                     LFAVEVALFRLVESWGVRPDFLAGHSVGEFAAAHVAGVFSLEDAAALVAARGRLMQAL
                     PSGGVMVAVQASEEEVRELLSGYEDRAGVAAVNGPSSVVVSGAEEAVAALVDRLSADG
                     RKTKALSVSHAFHSPLMDPMLDEFRKVVETVTFAAPQLPVVSTLTGALVSAEEFCSVD
                     HWVRHVREAVRFADAVTALADEGAGTFLEMGPGGVLTAMAQDSLDEHAVAVPLLRTDR
                     AEDLAVTTALAHLHAHGTSVDWSAVFAGHGTPRQVDLPTYAFHRRRYWLAPGSPAGDV
                     ASAGLVAAEHPLLGAAVALADGEGALLTGRLSPATHPWLTDHTVMGTVLLPGTALVDL
                     ALRAAEEVGCDRVDELTLGAPLVLRERAALRLQAVVGGPDATGHRTVNVYSRPESADV
                     AEPWTCHATGVVSVDPRRDAGGGQPHDLAVWPAPGADRLDTGGAYERLAGLGFGYGPV
                     FQGLHALWRRGDEVFAEVRLPDGTETGGFGIHPALLDSALHAIGLGGLLPDAGHGRIP
                     FAWSGVTVHATGATALRVRITPAGADAVALLATDTAGRPVASVDSLVLRPVSAEQLDR
                     AGREHGPYDSLYRVDWTALPLTSEASVSEPAGQWTLIGGDDGLRAALEDSGASVSYRP
                     GPDGAAALGGVVLAAVDVHPDGDHPVAHAHESAHRTLELLQHWLSDERNADTRLVVLT
                     ANAVVAKDRDQEDHDKEVDPAQAAIWGLVRSAQSENPGRFVLVDLDRDAASVRALPAL
                     LASGEEQFAVRGGSVLVPRLARAETPASLDDPSPAFTAGGTVLVTGASGLLGRHVARH
                     LVTGHGVRDLLLVSRSGALAAGMAELEAELAERGARVTVAACDVADRDALARLLAEVP
                     AEHPLTGVVHVAGVTDDGIVTGLTAERIDRVFRPKVDAALHLDELTRDMELSAFVLFS
                     SAAGVFGAAGQGNYAAANSFLDALAQQRRAAGLPAVSLAWGLWAESSGITGALSDADR
                     GRMARSGVLPLATDEALGLFDAATARPQGTVVPVRLNQAALRGRAAAGSLPALLTSLV
                     RVPARRPAADEAGYAASGAATGAGAEGTERRELSARLAGLAEAEQRKLLLDLVCEHVA
                     AVLDLASPRAVRPGQALRDLGFDSLTAVEFRNRLGKDTGLRLPPTLVFDHPTPAELVE
                     YLQAEAAPPAPGVAGMLAELDRIETALRNTAAAGGEGGEAIASRLRDLLDQWATTTGA
                     ADGGTAPEASGTSGATGPADVADGLHAATDDEMFDFIGKEFGIS"
     gene            complement(73736..75337)
                     /locus_tag="SLAV_02815"
     CDS             complement(73736..75337)
                     /gene="stvC"
                     /locus_tag="SLAV_02815"
                     /EC_number="1.1.3.6"
                     /inference="ab initio prediction:Prodigal:2.6"
                     /inference="similar to AA sequence:UniProtKB:P12676"
                     /codon_start=1
                     /transl_table=11
                     /product="Cholesterol oxidase precursor"
                     /protein_id="ATZ22474.1"
                     /translation="MLGLAALGAAAMAGSTTIGATRALAADRAGTPAFVPAVVIGTGY
                     GAAVTALRLGEAGVRTVMLEMGQLWNQQGPDGNVFSGMLKPDKRSSWFKTRTEAPLGS
                     FLWLDLANRDIDPYAGVLDRVNYDQMSVYVGRGVGGGSLVNGGMAVAPKRSYFEEVLP
                     RVDSAAMYDRYFPRANSMLRVNHIDNGWFEGTDWYKFARVSRDQAQKAGLGTVHVPNV
                     YDFDYMRREANGEVPKSALAAEVIYGNNHGKQSLDKTYLAAALGTGKVTIETLHQART
                     IRQQKDGTYLLTVEQKDADGRLLATREISCRHLFLGAGSLGSTELLLRARETGTLPDL
                     SSEIGAGWGPNGNIMTARANHVWNPTGASQSSIPALGIDDWDNPAAPVFAEIAPMPAG
                     VETWVSLYLAITKNPERGAFVYDAANDRANLRWTRDQNTPAVNAAKSLFDRVNRANTT
                     MYRYDLFGKQLKAFSDDFCYHPLGGCVLGKATDDYGRVSGYKNLYVTDGALIPGSIGV
                     NPFVTITALAERNIERVIQQDIKAS"
     gene            complement(75471..75728)
                     /locus_tag="SLAV_02820"
     CDS             complement(75471..75728)
                     /gene="stvB"
                     /locus_tag="SLAV_02820"
                     /inference="ab initio prediction:Prodigal:2.6"
                     /inference="similar to AA sequence:UniProtKB:P18325"
                     /codon_start=1
                     /transl_table=11
                     /product="Ferredoxin-2"
                     /protein_id="ATZ22475.1"
                     /translation="MTGDPLRIEVSRDLCRGAGMCTLLAPEVFDQSDEGVVLLLDARP
                     PAALRAAVREAADQCPCAAITLHKPSPMPEVPEVPEVPETP"
     gene            complement(75725..76921)
```

```
                     /locus_tag="SLAV_02825"
     CDS             complement(75725..76921)
                     /gene="stvA"
                     /locus_tag="SLAV_02825"
                     /EC_number="1.14.-.-"
                     /inference="ab initio prediction:Prodigal:2.6"
                     /inference="similar to AA sequence:UniProtKB:P18327"
                     /codon_start=1
                     /transl_table=11
                     /product="Cytochrome P450-SU2"
                     /protein_id="ATZ22476.1"
                     /translation="MSQGAPVIFSTVRDGYFNPPPMTQSLRRTEPIARMEFADGHLGW
                     LVTGYSAARAVLADNRFSARGELKHPPVPRAATLEEAPAAPPGMFIQHDAPEHQRYRK
                     LLVGQFTVRRMRMLTEWIEGIVAERLDAMERLGSEADLFEHFALPIPSLVICELLGVP
                     YADRAGFQHDTHVWSSFGESTEVVTAAFLSLGTYLQGLVRLKRAEPTDDILSGLIAAD
                     PDLTDEELTSIAFLLLVAGHETTANQLALGTFALLRHPEQLAALRADPSLIEGAVEES
                     LRYLSIVHHGPTRAALEDVEIDGVLIKEGEVVMVSLAAANRDPERFPDPEAFDVSRRA
                     GGHLGFGHGIHQCLGQQLARIELRVGFTALLERFPALRLACPPEEIRLRHDMQVYGVY
                     ELPVAW"
     gene            complement(77230..80016)
                     /locus_tag="SLAV_02830"
     CDS             complement(77230..80016)
                     /gene="stvRIII"
                     /locus_tag="SLAV_02830"
                     /inference="ab initio prediction:Prodigal:2.6"
                     /inference="similar to AA sequence:UniProtKB:Q10550"
                     /codon_start=1
                     /transl_table=11
                     /product="Putative HTH-type transcriptional regulator"
                     /protein_id="ATZ22477.1"
                     /translation="MRPLLHRQKELDLITGAMDAARHGSGGGSLVVVTGGLGTGKTAL
                     LRALPVRAERRGLRVVTASGAAFERDFEAGVLGQLLAPLLPGTPVAPPGDCAGPSAGP
                     LQDDGLLALVAEYGAHTPLLMLVDDLQWADAASLRWLGLLAGRVAGLRVTLVVTLREG
                     DPGEDDPRLQQLVEGASRVLRLGPLPPEGTADLIGALTGRPVDPAYARACHEVTGGRP
                     LLLTAVLDALSGPGEGPGSADDVRAVHAVRPAALYERFAVVLRDQPAPVREFATALAV
                     LGDGAEEELIGRLAGLDGTGRIEALRVLRRLGLVCEGPALRFVHPVVGEAVEEAMTPA
                     EAESIRVHAALLLHLGGHPAERAAAQLLAASSCHEDWESDVLRAAAASALRRSAPDTA
                     ARYLRRALLGSSPEGPDRAELLVELAMVERDFAPRAALRHLAQALFLFPETDQRAPVA
                     GRIPPFLLDGAPSTTVDTVVRTAAELGEPATLTGTARGHALRLEARLRHPALAGPEEP
                     AACGARLRALGPAPSLDTAAERELVTVLLHGAALAQTVNAAEAVALAERLLQHEPAAT
                     DHVHTALPLLTDVLVAADSLATIGPWLDTAYERARRENADVPRALIAVELTRLALARG
                     DLTGAAAHAAEALAQDATDWATLQTLAPVVLAALEARDTALCERLLSGRREEAAPSHR
                     PSLRRLIHGSAAVLRDDLPTALDCFLEWGRAAERAHWRNPAIVPWRTWASALHYRMGR
                     PDRAHDLMEEEYARALSWGSPVAIGRAQRGKGAITEGVRGIALLRESALTLEGSANAL
                     ERARTSLLLGRRLLAQGRQAEAEDRLTRARTEALDCGAPWMVERAVRALQGIAGSRGE
                     DVAATLTRTERRVAALASRGASNKAIAERLEVSSRAVEKHLTHVYRKLRVDGRTGLAA
                     MARLLSDGAGPVHP"
     gene            complement(80064..83030)
                     /locus_tag="SLAV_02835"
     CDS             complement(80064..83030)
                     /gene="stvRII"
                     /locus_tag="SLAV_02835"
                     /inference="ab initio prediction:Prodigal:2.6"
                     /inference="similar to AA sequence:UniProtKB:Q10550"
                     /codon_start=1
                     /transl_table=11
                     /product="Putative HTH-type transcriptional regulator"
                     /protein_id="ATZ22478.1"
                     /translation="MERTTAAHPVRATPPVHRARPGGAGEPAALVGRDTEAGLLHGLL
                     GQLRTGRPALVEVHGPPGIGRSALLDHTAALAHGTGVRVAAVRATAEEAGLPHGVAGR
                     LLARLPEAPARTGPVPYGTARPGAGRADGVRPRRTADLCAGVLEAAAGGPLLLVVDDV
                     QWADPQSLRGLRSLARRMTGVPLMLLTSRNSAVPATADERIDVCALAGEDAVVHHALT
                     LGPLPDGAVGEFLAGTWRAPVDEPFLADAVRHAEGSPALMRSIVGRFVRHGWTPGTGP
```

```
                     AGRLEESAAEAVRERTVRTLALLPDELLGVLRAIAVAGPDCTPGLIDALAGPRALGTS
                     RALALLRGTGLLAPGPRPALRTAAAARAVLAELGSEHREELHARAADWAHRAALPDRA
                     LARMLLDARRIGATWAVQALRREAEECRATGRPGRAARLLERALREPVPPALRIRLLT
                     DLSAAVLPTEPEAASRHLYRALQTPADGSAAVADTAADAETGRALLRAAELLVARGDV
                     DTPRPRIARAADAVRPGSAERSGLRALHWITEYSRPDPADGVEPLLPDSCPLERSAEA
                     AVAAWRTAQRGRNIDRARRLARTALAPQVREHVPLTVRAAAAHALVLTGDTAEARRAL
                     DEVLVQAERSDARAVAGLALLVTCLAELRAGRDTAAAEALDRCEGIMPGRCWHPLMAP
                     GPLALRALLHLRRGDRTAAERALAAELPPGAEGGLPWAYLLYARGRVALAGGTREQAF
                     ADLLECGRQLLARQAVNPAVLPWRSAAARAREPGDPLAAALLAEERRRALLWGSPAAM
                     AAALGEGAAAAGAGGTPAPGRGRPVSWQYRQALVALGTAPSSGHLTVDSLLGSDRGAA
                     AAPRPATPALPLPSRSASGSASGSDSRSASGPLPPSRFPSVSPPPPASVRTPAGGGAP
                     GRTARGGGPLTRAEQRVAALAAGGLGNRAVAEELAVTPRTVELHLTKVYRKLGIRGRP
                     QLAAALRRTAGGAPGA"
     gene            complement(83121..85964)
                     /locus_tag="SLAV_02840"
     CDS             complement(83121..85964)
                     /gene="stvRI"
                     /locus_tag="SLAV_02840"
                     /inference="ab initio prediction:Prodigal:2.6"
                     /inference="similar to AA sequence:UniProtKB:Q10550"
                     /codon_start=1
                     /transl_table=11
                     /product="Putative HTH-type transcriptional regulator"
                     /protein_id="ATZ22479.1"
                     /translation="MVVTVGTARAHGPGRSLDTHTAALDDLLRHAAGARAGTARAVLL
                     RGPSGIGRSRLLAVASERLGAEGMAVRSVAGLPGGASRIRAVDALLEPPPEGPCARPG
                     GAHEPRDTHRDWRRLHRRALDLLADGPTALVVDDAQWCDEASLRCADFILRRSADQPL
                     LIVLAQRTGVPGPGTRVLTEILAQDRCALLELGPLDERDTQRMITRVLGAPADPAFVR
                     RCTEITGGNPLLLDRLLHTLSTVGIPPGADALHQLSTVHQTVLASLVPEFLAAGPGPV
                     RQVATALAVLGRAAVDPLAALSGLSARHVESALGRLRHIEAVPADSPPRMREGIRTAV
                     LAALDCEELRGLRVRAARVLSDAGRPAAEVAEQLALLERIDEPWMFAVLREALAEAPT
                     RATVAAAGRWLRGAPGRRLTTAERADLHVELARAAARTIVAPADALWHLRQALALLPH
                     PRDRAPVAVQYAMMALGTRRAPEALRLLERVLGDLCSGTGAGPGPADREVRVTVESAL
                     LATAVNDRSALPAARARAAALPVPSGHSPADRRLLAALSAFAALDGGSAGRAAALARQ
                     ALRADEPGTAGWGVFCCSAVLALADSVDEALTALDQALSPAETQPPLWPRRAALAGRS
                     LIRHGTGDVPGAARDAWAAVEVVGPAGAPPFARIALSSVLLSQGEARQAAAVLDALAR
                     SAPDLDRSVWEWQHYLYTKGRILRELGDPEGALELWRRCGRSEEEAGIANPVLAPWWL
                     PTATTLGGLGRGAEAAGIVAAARERARRWGTPRALGLGLVAQAAVVEGRDRTGLLARA
                     VDLLSESPARLELAKAEYQLGRELLALGDARGARGHLRRAIGLATRCGYRVLAAVARD
                     ALVTAGGRMPQLATVPLDSLTHSERRIATLAERGHSNKGIAEALFITPRTVEMHLTNV
                     YRKLDVRGRADLPRGLGRHPGAPRPADGLSATS"
     gene            86326..86559
                     /locus_tag="SLAV_02845"
     CDS             86326..86559
                     /locus_tag="SLAV_02845"
                     /inference="ab initio prediction:Prodigal:2.6"
                     /codon_start=1
                     /transl_table=11
                     /product="hypothetical protein"
                     /protein_id="ATZ22480.1"
                     /translation="MQSSLELACPPPGWAVSDWPTTRPPPDEGSLDLWLLHVSDAGLR
                     SAVLTGPPWTRRNARGSRPSSTPPTATATWPRT"
     gene            86544..86849
                     /locus_tag="SLAV_02850"
     CDS             86544..86849
                     /locus_tag="SLAV_02850"
                     /inference="ab initio prediction:Prodigal:2.6"
                     /codon_start=1
                     /transl_table=11
                     /product="hypothetical protein"
                     /protein_id="ATZ22481.1"
                     /translation="MAAHLTLRRVLSAHRGVAPAELRFARRACPCCVGPHGRPVLAGS
                     GTPHFSMSHTGGLVLIAVAGRPVGVDVERLPAPHKATTGPELAVHSHFLGSLGRNSA"
```

```
     gene            87407..87925
                     /locus_tag="SLAV_02855"
     CDS             87407..87925
                     /locus_tag="SLAV_02855"
                     /inference="ab initio prediction:Prodigal:2.6"
                     /codon_start=1
                     /transl_table=11
                     /product="hypothetical protein"
                     /protein_id="ATZ22482.1"
                     /translation="MDLTPYVDTLRRELAVAAEAGGDEARELAERLTAPLESATRLTM
                     LHVLSAAMDEITRELAPGSVDVRLRGLDPDFVVTLPPADTGASEAASAPVEPVGAQVP
                     AEGDETGTARVNLRLPAHLKARAEEAAGREGLSVNAWLVRAVSAAVEGGTRPHTTQKS
                     QTVGQSFTGWVR"
     gene            88050..88892
                     /locus_tag="SLAV_02860"
     CDS             88050..88892
                     /locus_tag="SLAV_02860"
                     /inference="ab initio prediction:Prodigal:2.6"
                     /codon_start=1
                     /transl_table=11
                     /product="hypothetical protein"
                     /protein_id="ATZ22483.1"
                     /translation="MPSFDTPEPIAVTARVEAGSIQFTAGDRPDTVVEVRPRDPKRDL
                     DVRTADQTEVTYTGGVLTIRTPKSNLLGRTGTVDLTVELPTGSRIDTTGAWTQVVGEG
                     RLGEVRVKTSSGDVRLDTTGPLKLTASHGSITVDRIEGTAEITTSSGSLRVGVVDGPA
                     VLKNSHGTTTVGAATGEIRVSGANGDIEIRRAEGPVTATTAHGTLRLGEVTRGTVQLE
                     TSYGAIEVGVREGTAAWLDVNSGHGQVRNSLTASETPGEAEDTVKIRARTRYGNIDVR
                     RAKA"
     gene            88995..>89942
                     /locus_tag="SLAV_02865"
     CDS             88995..>89942
                     /locus_tag="SLAV_02865"
                     /EC_number="3.6.3.-"
                     /inference="ab initio prediction:Prodigal:2.6"
                     /inference="similar to AA sequence:UniProtKB:P32010"
                     /codon_start=1
                     /transl_table=11
                     /product="Daunorubicin/doxorubicin resistance ATP-binding
                     protein DrrA"
                     /protein_id="ATZ22484.1"
                     /translation="MPSSVMPTSNHNDGHPSQVAISAAGLRKSYGDKTVLDGIDLNIP
                     AGSVFALLGPNGAGKTTAVKILSTLITADAGQARVGGHGLTTNPQAVRAAIGVTGQFS
                     AVDGLITGEENMLLMADLHHLPKPEGRRVAAELLERFDLTAAAKKPASTYSGGMKRRL
                     DIAMTLVGSPRIIFLDEPTTGLDPRSRHTMWQIIRELVTGGVTVFLTTQYLEEADQLA
                     DRIAVLNNGKIAAEGTAEQLKRLVPGGHVRLRFTDPTAYRTATHTLPTATGDNESLTL
                     QIPSGATQHELRTLLDHLHTTGIEADELTVHTPDLDDVFFALTDTAHIPHQPTEATR"
BASE COUNT    11253 a  31796 c  35862 g  11031 t
ORIGIN
        1 cggtgtgtac gtggacgaca aggcccgcgt gacgtactgg aacagcagcg cggaggccgc
       61 cgtgtcgccg ctgtccgccc actgacgggg ccgcgtgggc gcgaacgcgc acgagggccc
      121 gctccagggg gccggtcccc ggcggggcgg gcgccgacgc tagcgtgatc accatgtacg
      181 agatcaaggg cgtgagagcc acgccgggcg gacgtatcag cgtccggttc caccagcacg
      241 gctacgtcga aagaccggac cggcgcgccg gccgcgtcgc cgagacactc ttcggtgagg
      301 gagccgggtg cctcaccgca ctggtgctgc tcgcatcggt gggcctgttc accctgctcc
      361 gcaagaacgg ggcgccggag gcggccgtca cggcctgcat gtgggtgttc gtcgtggcgg
      421 tctgctacgg cgcggtcatc gccctgttgc actgggcctc cggggtcgcc gacgtcctgt
      481 tgatcgcggt gacgttcccg gcgctgctga tcccgggcta ccggcgcatg ctgctgcgcc
      541 gctggcgcgg cggtgctccg gagagcgagc cggggtgggt tccgggggcc gctctctccg
      601 gggtgtggca ccaggtggac ccgcagggcg gcgccgtggt gacggtccag cggaccgacg
      661 gatccgtcac cgcgtacgtt cccccggccg acaaggcccg caacctgcac gaccacttcg
      721 acacactgct gcgccagggc cgcccggccc cggccccgta tcagcagccg gcctacggcg
      781 ctccggggta cccgccccag ccctggcagg ggaacccggg ccaccggcac gactgacccg
      841 gccccaccca gccacccgct acgcggcccg ctccggcggc gggccgctcg gccggtcacg
```

901 agaatcctga tcacgatcac gccgaccgtg ggcgcgcgtg cggtatgccc ggccgtggag
961 ttcggcgcag gaccgtggtc agaagggtgg ggagtccgtc gtcagggcgg cgagccaggt
1021 ggtgtactcg gcccggcggg ggcaggtggc ggtggcgcgg ggggtggccc ggcaggcgcc
1081 cggtcggcag tcctcggggc tgcggtcgcc gtggaatggg gtgctgccga ggggcgccgc
1141 gccctgacgc ggcgtgaggt cggcgtcccc gagcgcgggc agcagcagga gcaggcgggt
1201 gtcgggatcg gtggtggttc cgccgggcag gccgttgtcc gcggcgccga tcagctcggg
1261 ccaggacagg cccggtcccc tccagtggcc gtcgtcccgg gcgaggaact cggcgtgctc
1321 ccagtcgggg tgggtgatca ggtagtggac gcccgcgtcg tccccggcgt tctcggagga
1381 cgtgcaggcc cagtccgtcc gccaccggaa cggtgaacac gggccggtcg cgggtgatgg
1441 ctcgggcgtg ggtgcacgtc cgccatgatc gcccaagcgt ccgcggtggc gccgggcggg
1501 aacgcggagt tcccgcccgc ggacggggtc aggggtcac gggcgtgcgg aggtggtccg
1561 tcatggtgcg gatcacctcg gcctggtcgc gtaccaggta gaagtgcccc cgggagaaga
1621 ccttcagggt gaacgggcct tcggtgtgcg cggcccaggc cgccgcctcc tgcggcgtca
1681 ccttcgggtc ctcgctgccg atcatggcga ccagggggca gtgcaacggc tcccccggct
1741 cgggggcgta ggtttcggcg gcccggtagt cggcgcggat ggccgggagg atcatccgga
1801 gcacctcctc gtccccgagc agctgtgtgt cggtgccgct gaggctcttg acctcctcga
1861 tcagaccgcg ctcgtcgcgc aggtgaacgg tctcgtggcg gggcgccgac ggggcccggc
1921 gtcccgaggc gaagagcgcc gccgcgacga tgcccttctc gcgttcgaaa cggcgcgcga
1981 cctcgtaggc caccgaggcg cccatgctgt ggccgaagaa cgccaacggc cggtccgccc
2041 agggcagcag tgcggtgaac acccggtcgg cgagttccgg gacgctgtcg atcaggggct
2101 cggctcggcg gtcctggcgg ccggggtact ggacgcacag gacgtcggcg aagtccggca
2161 tggtccgggc catgggcagg taaaagctcg cggagccgcc ggcgtgcggc aggcagacca
2221 gtcgcacggc ggcgtccggc ctcggctgga accggcgtgt ccacaggccg ttgtcggtgg
2281 agacgggggc gggggtcggg gtgctcatgg tgtcgggtgg ttcccttcgg tgaaacgggc
2341 ggagcggggg ggaacgggtg cggtgtcggc gggtcagggc agcttgagga cccactcgtc
2401 gaccgcggtc gccgtggtcc gtgcgtgacg ttccatcagc gtgtagtggt cgccgggtac
2461 ttcggtgacc tgctgggcgt gttcgggcgc cggcggccgg tcgtcggtcc cctccgcgcc
2521 gtccgccatc cgctcctcgg cgcggacgac cagggtgggg gcctcggcaa cgggggcgtc
2581 ccagtcctcg aagacggcga ggtagccgcc catcgcggtg agctgggtgc cgggtgccga
2641 gatgaagtcg aagcgctccg actgttcccg tacgaccgcg gactggaacc ggtcggccat
2701 gccctcttcg cgggtgaagc tgtcgagcat cacgatgcct tcgacgggcc gccccagctc
2761 ctccagccga caggccacgg cgtgcgccac ccagcctccc gaggagtagc cgacgagcac
2821 cacgggctcc tcgccggcgc agtcggcgac ggcccggacg tgcacctcga cgagggccgc
2881 gacgtcctgc ggcagcgcct cccccgggcc gaagcccggc tcgggcaggg cccagacggt
2941 gcgccggccg ccgaaggcgg cggcgaagcg cgcgtactgg tgcaccccgg aggggggccat
3001 gtaggggccg aagcagatca gtgggggccc ttcgtccccg tgggccagcc ggaccgggac
3061 cggcctgtgg tcggcggccg acgcggcgtc gaagaccggt cgcagccggg ccgcggcggt
3121 gagcatcccg atgccctcgg agagcctgcc cagttcaccg gcccggcggt agaggccgac
3181 gagcaggtcg tcctgcccgt cgcgcggggc cggtccggcc ccgtggggcc cgccggaggc
3241 ctccgccagg agggccagga gccgggtggc caccgcctcg gggtggggt ggtcgaagac
3301 cagggtgctg ggcagtttga ggccggtggc cccgccgagg cgcttggcga gccggacggc
3361 ggtcagcgag tcgaagccga gttccaggaa gacgtcccgt tcccgtacgg cttcggcgga
3421 gaggtggccg agtacggcgg cggcttcgga gcgtacgaag tccagcacgg ccgcgtcgcg
3481 ttcggtggcg ggcaggcccg tcagccggcg tacgaggagg ggttcgccgc cggactccgc
3541 gtccgcgccc gtctcctcgg cggccagcag gaggaccacc tcgggcaggc cgccgagcag
3601 cgggctcggc cgggcggcgg tgaagccggg caggaagcgc ttccagtcca cgtcggcgac
3661 gatcaccgag gtgtccccgt gatcgaccgc gcggcgcagg gccagtacgg cgagtccggg
3721 gtccatcggg tccaggccgc gcagcctcag ccgttcctcg gcggcccggt cgaccatgcc
3781 gccgccgccc caggcgcccc aggcgatgga ggtggcggcc aggccgcgcg cccgccggtg
3841 ttcggcgagg gcgtcgaggg aggcgttcgc cgccgcgtag gcggactggg cgccgctgcc
3901 ccaggcggcg gcgccggagg agaacaggac gaaggcgtcc agtgcgcggt ctccgagcag
3961 ggcgtcgagg tgggccgcgc cggccgcctt cgcgcccatg acctcggcgg tctcggccac
4021 ggtggtgtcg gcgagcaggg cgggcgtgcc gatgccggcg gcgtggacca cggcgctcag
4081 cggcggcagg ccggcgaggg actccaggag cgcgctcacc gccgtccggt cggccatgtc
4141 gcaggcggcg agcgtgacgc gggcgcccag cgcctccagt tcggcgcgca gctcttcggc
4201 gccttcggcg gcggggcctc gtcggctggt cagcaccagg tgttcggctc cggcggccgc
4261 cagccagtgg gcgacccggc tcccgatgaa gccggtgccc ccggtgacca gtacggtgcc
4321 gcgcggctgc cagccgggcc cggcgggact gtcggtgtcg gtggcggtgt cggtggcgcg
4381 gaccatgcgg cgggcgtggg cgccgtcggc gcgaagggcg atctgatcct ccccggtggc
4441 tccggagagg gcggcggcca gtcggcgtcc ggtccaagtg gcggtggccc ctacgtcggc
4501 gggcaggtcg acgaggccgc cccagcgtcc cgggtgctcc agggccacga cgcggcccag
4561 cgcggccacc gtcgcctggg gtacggcgtg gacggggtcg gcggggcggg tggacacggc
4621 gccccgggtc acgcaccaca ggggcgcgtc gacgtcggcg tcgccgaggg cctgcacgag

4681 gagcagggtg gtggcgaggc cgagcgggac ggccgggtgg tcggggtggg cgtggtccgc
4741 gagcgcgagc agggacagga ccccgcgtac gccggggccc gcttcccgca gcctgtcggc
4801 gagggtctgg cggtcctcgt caccggtgac ggccagccgg acggtgtcgg ctccgtgcgc
4861 ggcgagggcg tcgagcgcca gctccgcggc cgggtcctcg aaccggtccc gggggacgac
4921 cgcgagccag cgcccggtga gggaaggctg cgccgtgtcg gggaccggct tccagtcgat
4981 ccggtagcgc caggcccggg tggcggcctc gtcgcggcgg ccgcggtgcc aggcgtgcag
5041 tgcgggcagc gcggcggcga gggtggtgtc ctcgtcgaag cgcagttcct cggtgaggga
5101 ggccaggtcc tcgcgttcca cggctgccca gaactcggcg tccgcggcgg actgcggcgc
5161 cggggccgtc ccggccgccg cgtcgggcgc gtcgagccag taccgctgcc gctggaaggg
5221 gtacgtgggc agttccgcgg gcgtacccgt ggggccgccg aacaccgccg gccagtcgac
5281 cgggacgccg tgcgcgtgcg cctctccgag ggaggcgagg aagcgccggg cgtcgccggc
5341 ggagcgggtc agggtcggca cgacggcttc ggcggcgccg tccgtgcgcg cgtcgtcgag
5401 ggtgtcctgg agtccgtaca ccagcaccgg gtgggggctg atctcgatga agacccggtg
5461 cccgtccgcg cgcaggcgcc ggacggcctg ctcgaagcgt acggtccggc gcaggttgcg
5521 gtaccagtac gcggcgtcgg gcgccgtgtc gagcaggtcg cccgtcacgg tcgagcagaa
5581 cgggatccgc ggcgcgttcg ggacgagcgg ggcgagttcc gcggcgagtt cctcccgcac
5641 ctcctcgacc tgggcgcagt gcgaggccag gtccaccctg acgcgcttgg cctgtacgcc
5701 gtcggccgcg caggcgtcga cgagttcggc gagggcctgc gggtcgcctg cgacgacgac
5761 ggtgcgcggc ccgttgacgg cggccacggc gaggcggccg tcccaggcgg agatgcgctc
5821 ggtgacctcg gcctcggacc gcgggaccga caccatgccg ccgcgtccgg ccagccgggc
5881 gagcagggcg ctgcggcgtg ccacgagccg ggcgccgtcc tccagggtca gtgccccggc
5941 gacgacggcg gccgctatct cgccctgcga gtggccgacg acggcggccg ggcggacccc
6001 gcacgcctgc cagagttcgg cgagggccac catcaccgcc cacagcaccg gctggatcac
6061 ttcggggccc tccagcgcgg gggctccgtc cgcgccccgc aggacgtcgt cgagggacca
6121 gtccacgtag gggcgagcgg cttcgccgca ggcggtgatc cggtcgcgga agacgggcga
6181 ggtgtcgagg agcccggtgg ccatgcccgc ccactgggaa ccctggccgg ggaagacgaa
6241 caccggctgg gcgggggatc cggccgaacc gcggaccgcg agctcgtggg agccgccgga
6301 ggcgagcgcc tccagggccg cgaccatctc ctcgcggtcc ccgccgacca ccacggcgcg
6361 ttcgtcgaac agcgtacggg tggtggcgag ggcgtacccg atgtcgcgcg gccgggtacc
6421 gtcctcgcgc agcagcgcgg cgaggcgccc cgcctgctgc tccagtgccg ctcccgtgcg
6481 ggcggacagc ggcaggggta cggccgccgc tccggccggg ggccgggggg cgggctcctc
6541 gggacccggc tgcggtgctt ccggtgcctc ccctgcctcc ggggcttctt cgatgatgac
6601 gtgggcgttg gtcccgctca tcccgaagga ggagaccgcg gcccggcggg ggtgctcggc
6661 ggggtcccag gcgatgggct cggtcagcag ccggacggcg cccgcgctcc agtcgacgtg
6721 cggggtcggt tcgccggcgt gcagggtcct ggggagcgtt ccgtgccgca gggcctgcac
6781 catcttgatg atgccgcccg ctccggcggc ggcctgggtg tgcccgatgt tggacttgag
6841 ggagcccagc cacagcggcc ggtcagcggg gcggtcctgg ccgtacgtgg ccagcagcgc
6901 cgaggcctcg atggggtctc cgagccgggt gcccgtgccg tgtccctcga ccgcgtcgac
6961 gtcggcggcg gacagtccgg cgttggcgag ggcctggcgg atgacgagtt gctgggcgcg
7021 gccgctgggg gcggtgaggc cgttggaggc gccgtcctgg ttgacggcgc tgccgcggac
7081 gacggcgagg acgcggtgtc cgttgcggcg ggcgtcggac agccgctcca ccagcagcat
7141 gccgacgcct tcgccccagc ccgtgccgtc ggcgtcggcg gagtacgcct tgcagcggcc
7201 gtcggcggag agggcgcgct ggcggctgaa ctcgacgaac gctccggagc tggacatgac
7261 cgtcaccccg ccggccagcg cgagttcgca ctcgccctgc cgcagggcct gggccgccat
7321 gtggagggcc acgagggagg aggagcaggc cgtgtcgacg gtgacggcgg gtccttcgag
7381 gccgagcgtg taggcgatcc gcccggaggc gacgctggcg gcgttgccgg tgcccaggta
7441 gccctcgacg tcctcgggga cggcgatgcc gcgccggatg tagtcctggt agccggagcc
7501 gacgaacacg ccggtgcggc tgccgcgcac gctcgccgga tcgatgccgg cccgctcgaa
7561 ggcttcccag gacgtctcca ggaggagtcg ctgctggggg tccatggcgg tcgcctcgcg
7621 cggcgagatg ccgaagaagg cggcgtcgaa gtcggcgagg tcgtagagga atccgccggc
7681 gcgggcgtag gtggtgcccg ggtggtcggg gtccgggtgg tagagggcgt ccaggtccca
7741 gccgcggttg tcggggaaga ccgacatggc gtccgtgccc tcgtccacca gccgccacag
7801 ctcttcgggc gagcggaccc ctcccgggta gcggcagctc atggcgacga tggcgatcgg
7861 ctcgtcgtcg gcgcgtacgg gcgcggcggc gctcccggcg gtgtcggcgg tgccgagcag
7921 ttcgacgagc agtgcgcccg cgaggtcgag gaccgacggg tagtcgaaca ccatggtggt
7981 gggcagccgc agtccggtgg ccttgacgag gcggttgcgc agttcgacgg cggtcaggga
8041 gtcgaagccg aggtcgcgga aggcccggcc cggctcgacg gcctcggggc ccgggtgtcc
8101 gagcacggac gcggcctcgg cccgtacgag ttccacgagg gtgcggtcgc gttcggccgg
8161 gtcggcccgg tcgagggccc ggcgcagggc gtcggtggtg tcggccgcgc cgtcggggtg
8221 gccgggtcgt gcgacggccg cctcgcggac ttcgggcagg tcggcgagca gggggctggg
8281 ccggccgacg gtgaaggcgg gggcgaagcg cgcccagtcc acgtcggcga cggtcacggc
8341 ggtcgcgttg cgggcgacgg cgcgttccag cacggtgagg gcggacgcgg ccgccagggg
8401 gtcgaggccg cgccggcgga ggttctcggc gatcccggga ccggcggcca tgcccgcgtc

8461 ggcccagggt ccccaggcca gggcggtggc cggcaggccg cgggcccggc gggcggcggc
8521 gagcgcgtcc aggtgggcgt tggccgcgct gtacgcggcc tggccgcccg cgccccacac
8581 tccggcgatg gaggagaaca ggacgaacgc gtccagttcg gcggggccgc cggggagggc
8641 gtcgaggagt tcgtcgaggt ggacggcggc ggcggccttc gcggccagga cgccggcgaa
8701 ggcggccggg tcggtgtcgg ccagcagggc ggtggtgtcc acgcccgccg tgtgcaccac
8761 cgcggtcagg gggccgtccg tgcccgccgg gtccgtcagg ccggtgagca gccgggacag
8821 cgcgtcacgg tccgagacgt cgcaggcggc caggtcacg cgggcgccca gcgcctccag
8881 ttcggcgcgc agttccccgg cgccgtcggc cgcggggccg cggcggctgg tgagtaccag
8941 gtgttcggcg ccccgggcgg cgagcagccg ggcgacgtgc ccgcccagcg cgccggtccc
9001 gccggtgatc agcacggtgc cgcgggggcg ccactccccg gcggtccgga cgtcgttccg
9061 gccggcacgg gagagccggc gggcgaacac cccgtcggcg cggacggcga gctggtcttc
9121 cgggccaccg gcggtgaggg cggcgaggat ccggtcgggt acggggccgt cgcggtgcgg
9181 gggcaggtcg aggaggccgc cccagcggcc ggggtgctcc agggcggcca cccggccgag
9241 gccccagatc tgggcctggc cgggggcggt caccgggtgg tcgtcgggcg aggtggacac
9301 ggctccgcgc gtggcgcacc acagcggcgc cccgacgccc gcgtcgccga gcgcctggag
9361 cagggtcagg gtggcggcga cgccgccggt cagggcggcc aggccctcga cggggcgttc
9421 gtccagtccg agcaacgaga cgacggcacg cgggggcgtg gcgtccgtgg ccccgcgcag
9481 ccgtgcggtg agcgtctccc ggtcctcggc ggcgtcgacg gtgagggagg tgacggcgca
9541 gccgtgtgcc gcgagcgcgt cccggagtcc ggccgcgtcg cgttcggtgg cgccgtcgtg
9601 gtccgtgccc gcgggcagga ccagcagcca gtggccgtcg gcggtcacgg tcgcggggtc
9661 ggtcgccggg tcgggcaggc gctgccaggt gacctcgtac tgccagtcct cgatcgtgga
9721 ctggcggcca ccggcggccg gggccgtttc gagccagtac tgctcgcgct ggaaggcgta
9781 cgtgggcagg tcgacgcggc gggcgccccg tccggcgagg agggcggtcc agtcgacggg
9841 gatgccgtgg acgtggagcc gggcgagggc ggtggtgacg gccaggccct cggggcggtc
9901 ggcacgcagt acggggatga cggcggcatc ggcgtcttcg aggaccgcct gggccatggc
9961 ggtgaggaca ccgcccggcc cgatctccag gaaggtgccc acgccttcgt cggcgaggga
10021 ggtgacggcg tcggcgaagc ggacggcctc gcggacgtgg cgcatccagt agccgaccga
10081 gcagaactcc tcggccgata cccgaccgcc ggtgagggtg gagacgatcg gtacgcgcgg
10141 ggcctcgaag gtgaccgttt ccaggatctg gcggaagtcg tcgagcatcg ggtccatcag
10201 gggcgagtgg aaggcgtggg agacggtcag cgccttcgtc ttgcggccgt cggccgagag
10261 acggtcgacc agtgcggcca ccgcttcccc ggcgcccgaa accaccactg cggaagggcc
10321 gttgaccgcg gcgataccg cacggtcctc gtacccggcg agcaggtccc gtacctcggc
10381 ctccgaggcc tgcaccgcca ccatcacgcc ccccgccggg agcgcctgca tcagccggcc
10441 acgggcggcc accagccgtg ccgcgtcgtc gagcgagagc actcccgcca cgtgcgctgc
10501 ggcgaactcg cccaccgaat gcccggccag gaagtccggc cggacgcccc aggactccac
10561 cagccggaac agcgccactt cgacggcgaa cagcgcgggc tgcgcgtagg cggtccggtc
10621 gagcagtccg gggtcctcgc cgaacacgac gcccttcaga ggacgttcga ggtgcgggtc
10681 cagtgcggag cagaccgtgt cgaaggcgtc ggcgaacacg ggaaaggagg cgtgcagttc
10741 gcttccggtc ccgagccgct ggctcccctg tcccgagaag aggaacgcgg tgtcgccgtc
10801 gcggccgccg gtgccctcga ccacctgcgc ggcggcggtc ccctcggcca gggcccgcac
10861 cccggcaacg agtgcgggca ggtccgtgcc gaccactgcg gcgcggtgtt ccagtgcggc
10921 gcgcgaggtc gccagggaat gggccacgtc caccgggctg acgtcgccga ggccgaggac
10981 gtgcgtcagc agccgctccg cctgctcgcg caggccgcg ccgttgcgtc ccgagagggt
11041 ccacgccacg ggtgcggggc cggtgagcgg atcggtccgg tcctgccccg agggggttc
11101 ttcggcttcg gggccggcg gggcctcttc gatgatggtg tgggcgttgg tcccgctgac
11161 gccgaaggag gagaccgccg cccggcgggg gcggccgtcc tctccccagg cgacgggttc
11221 ggtgagcagg cgtaccgtcc cgtccgacca gtccacgtgc ggcgagggct cctccgcgtg
11281 cagggtcctg ggcaggacgc cgtggcgcat cgccatgacc atcttgatga tcccgcccac
11341 cccggccgcc gcctgggtgt gcccgatgtt cgacttgagc gaccccagcc acaaaggccg
11401 ctcggccgaa cgcccccgcc cgtacgtcgc cagcaacgcc tgcgcctcga tcggatcacc
11461 cagccgcgtc cccgtcccgt gcgcctccac cacatccaca tcggctggag acagtccggc
11521 accggcgagg gcctgttcga tcacccgctg ctgcgccgga ccgttcggag ccgtcagacc
11581 attcgaagca ccgtcctgat tgaccgcact cccccgcacc accgcaagca ccggatgccc
11641 cagacgccgc gcatccgaca gacgctccac caacagcatc ccgacaccct cgccccagcc
11701 cgtgccgtcg gccccggcgg cgaaggactt gcagcggccg tccacggcga ggccctgctg
11761 gcggctgaac tcggtgaaca tgccggtggc ggccatgacg gcggccccgc cggcgagcgc
11821 catcgtgcac tcgccctgcc gcagggcctg gaccgcaagg tgcagggcga ccagggagga
11881 ggagcatgcg gtgtcgacgg tcagcgaagg gccttcgagg ccgaaggtgt acgacacccg
11941 gccggaggcg acgctgggtg cggtgccggt gagcaggaag ccctccagct cttcccgggt
12001 gtcgccgctg cccgcgtagc cctggtaggc gaggccgacg aagacgccgg tccgggatcc
12061 cttgacggag gtgggggcga tgcccgcgcg ttcgaacgcc tcccaggcca cttcgaggag
12121 cagccgctgc tgcgggtcgg tggcgagggc ctcgcggggt gacatgccga agaagccggg
12181 gtcgaagcgg cccgcttcgt gcaggaaccc gccctcgcga gtgtagcact tgccgggcgt

12241 gcccggatcc gggtcgtaca ggccatcgag gtcccagccc cggtcggtgg ggaagccggt
12301 gacggcgtcc gagccgctct cgaccagtcg ccagagcgct tcggggctgt ccacctgtcc
12361 ggggtagcgg cagctcatcg ccacgatggc gatcggctcc tggtccttcg cctccacctc
12421 gtggaggcgg cgccgggtct gcttgaggtc cgccgtcacc cgcttgaggt agtccagcag
12481 tttctgttcg ttctcgttct ggccgctgag gtcgtcgtgc tggttctgca tgtgtcgttc
12541 acccgccggg agtgtcagtg aggtggtgcg gtggtgcggg ccgcggaagt gtgcaccgtg
12601 tggtccgggt gctcaggcgc cgtccagctc ctggtcgatg agggcgaaga tgtcgtcgac
12661 ggtggcgctg cgcagccgtt cgtcggacgc ctcgtcgggc gaggagccgc cgcccgaccc
12721 gtcggccgcg gtgccctcgc cgagggcggc gagcacgccg tgcaggcggg ccctgatccg
12781 ggaccggacc aggtcgtcgt cggcgcctac ggcggcgagg gccgtctcca gccggtcgag
12841 ttcgccgaag accgcgaggg cggacgcgcc gggcgccggg tccggcacga ggtcgagccg
12901 gatccgctcg gcgagcgcga cgggcgtcgg gttgtcgaac acgagggtgg ccggcagccg
12961 gagtccggtg gcggcattga gccggttgcg cagctccacc gaggtgagcg agtcgaagcc
13021 gagctgcttg aactcccggt tgcggtcgac gtcgcgcggg ccgccgatgc cgagcaccgc
13081 cgccacctgg gtgcgtacca ggtcgaggag gacgccgtcc cgttcggggt cggacagggc
13141 gccgagccgt tcgcgcagcg cgtcggcgcc ttcggccgcc acggcttccc gggcggtgcg
13201 ccggacggtc cggcggacga ggccgcgcag gaccgccggg acctccccgg cggggtccgg
13261 gccggtggcg ggcgcggtgg tgtcgaagcg gacgggtacc gcgacggcgt cttcgcccgt
13321 ccgggccgcg tcgaagagcg ccaggccctc ctcggcgcgg aaggacacca gtccggaacg
13381 ggccatccgc ccgcgtcccc gctcgtccat cccggaggcc atgccgctgt cggcccaggg
13441 gccccaggcg atggcggtgg cgggcaggcc ggccgccgcg cggcgctggg ccaaggcgtc
13501 caggaacgcg ttggcggcgg cgtagttgcc ctgcccggcg ctgcccaggg tcgcggcgac
13561 ggaggagaac aggacgaact ccgacaggtc caggccccgg gtcagctcgt ggaggttgag
13621 cgcggcgtcc gccttggggc gcagtacggc gtccagccgg tccccggtga gcgtggcgac
13681 caggccgtcg tcgagcaccc cggccgcgtg gaccaccgca gtcagcggcc ggtccgcggg
13741 gatcccggcc agcagtgcgg cgagcgcgtc gcggtcggcg gcgtcgcacg ccgccagcgt
13801 gacggtctcg gcgccccgcg cgagcaggtc gtcgcgcagg gcggccgctc cctcggcggc
13861 cgggccgcgc cggccggcga gcagcaggtg gcgtgcgccg tggcgggtca ccaggtggcg
13921 ggccacgagc ccgccgaggg caccggtggc gccggtgacg agtacggtgc cgccggggtc
13981 gggtgcggcg gggacggtga ggacgagctt gccgacgtgc cgggcctggc tcaggtggcg
14041 cagcgcgtcg ggtgcccggc gtacgtccca gcaggtgacg gggagcggcc gcagcgcccc
14101 ggtctcgaac agcgccacca gttcgcggag catggcgccg atgcgttcgg gtccggcctc
14161 gatggtgtcg aaggcggtgt acgcggcccc ccggtgttcg gccgcggtcc gttcggggtc
14221 gcggacgtcc gtcttgccca tctccaggaa gcggccgccg tcggtcagcg tgcgcaggga
14281 ggcgtccacg taggcgccgg cgagggagtt gagcacgacg tccacgccgc ggcccccggt
14341 ggcggcgagg aaccgctgtt cgaagccgag gtcgcgggac gaggcgaggt gctcctcgtc
14401 gaggccgtcc gcccgcaggg ccgcccattt gccgggtccc gcggtgccgt agacctcggc
14461 accgaggtgc cgggccagct gggtcgcggc catgccgacg ccgcccgccg cggagtgcac
14521 cagcagcgac tgcccggcct tcaggccggc caggtcgacg agggcgtagt aggcggtcag
14581 gaacacgatc ggcaccgacg cggcctcggt gaacgtccag ccctgcggga tccgcgcgac
14641 catccggtgg tcggcgacgg cgagcgggcc gaacgcctcg ggaccatgc cgaacacccg
14701 gtcccccacc gccagtccgg tgaccccggg gccggtctcg gcgacgacgc cggcggcctc
14761 gctgcccagg gtggcggccc ccggatacat gccgagcgcg atgagggcgt cacggaagtt
14821 gacgccggcg gcgcgcacgg cgatccggac ctggccccgg ccgagcgggg cggtggccgc
14881 ggggttctcc gtcagggcca ggtcgtcgag ggtgccggtg gcgccggtgg tcaggcgcca
14941 ggcggtggcg ccgggggcgg gccgcaggcc gtcgccgggt gagcggcggg cgagccgggg
15001 gacgtacgcg gcgccgtcgc gcagcgcgaa ctgggcttcg ccggtggcga gcgcggcgtg
15061 cagggcccgt ccggacgggc cgtggccgtc ggggtcgtcg gtgtcgacga ggaccaggcg
15121 tccggggttc tccgactggg cggaccgtac cagaccccac agggcgctct gcgccgggtc
15181 ggtgaccgtg gttccgggta cgggggcgac ggcccccggg gtgagcagga ccagccgggc
15241 gtgtgcggtg cggtcgtcgc cgagccaggt ccgcagcagt tcgaggccgt gctgggtcac
15301 ctcccgtacg tcctgcgccg tagcgtcgcc gggcgcgtgc ggggcgggta tgaggacggt
15361 gtccgggagc gcggggagtc cgtcggcgcc gccgggggcg gccaggagcg cgaggagacc
15421 cgtgtggtcc gccacccggg tggggtcggg ggtggagccg ggttctccca gggggagtgc
15481 gggccagtcc atccggtaca gggtgtcctg gtgctcgacc cgccgcgcgg tggccggctg
15541 gtcggcggtc accgcccgca gcacgaggga ctccacggag gcgaccggac ggcccgtggc
15601 gtcggcgacg aggagggaca ccgtgtccgg ggcgggcgag gtgagccgca cgcgcaggcc
15661 ggtggccccg gtcgcgtcga ggcgtacccc gctccacgcg aacgggatcc gtccctgccc
15721 ggtgtccggg agcaggccgc ccaggccgac ggcgtgcagg gcggagtcga gcagcgccgg
15781 gtggaggccg aagccgtcgg cggtggtgtc ctgcggcagg gcgacctccg cgaacacctc
15841 cccgtcgcgc tgccacaggg cgcgcaggcc ctggaacacc ggtccgtagc cgaagccgag
15901 ttccgccagc cgctcgtagt acccctcggt gggtacgggc cgcgcggcgg cggcggcca
15961 ctgcgacagg tccgccgcgg gatcgggccg tgcgggtgcg gccgcctccg gcgcgtgcgt

16021 ggcgagtacg ccgacggcgt gcgtcgacca gggttcgtcg gggcccgcac cgtccggccg
16081 ggagtggacc cgcacggtcc ggcgcccggt gtcgtcgggt ccggcgacgg tgacctggag
16141 ctggacgccg ccccgggagg gcaggacgag cggggcctgg agggtgagtt cgtcgacccg
16201 gtcgcagccg gcctcgtcgg cggcgcggat cgcgaggtcg accagggccg tgccgggtag
16261 cagcaccgct cccatgacct ggtgttccgc cagccagggg tgtgccgccg tcgacagccg
16321 cccggtgagg acgacgccct cgccgtcggc gagcgccacg gcggcgccga ggagcgcgtg
16381 gtccgccgtg cgcaggccgg cactcgcgag gtccccggcc gcggggcggg tgtcgagcca
16441 gtagcggctg cgctggaagg cgtaggtggg caggtcgacg cgtcgggcgc cgcgtccggc
16501 gaggacggcc gaccagtcga cgggcgtgcc gtggacgtgg aggtgggcga gggcggtggt
16561 gacggccagg ccctcggggc ggtcggtgcg caagaggggg ctggtgacgg cgtcctcgtc
16621 gagggagtcc tgggccatgg cggtcaggac cccgcccgga ccgacctcca ggaaggtacg
16681 gacgcccaga tcggcgaggg ctgtgacggc gtcggcgaag cggacggcct cgcggacgtg
16741 gcgcacccag tactcggcgg agcagaactc ctccgcgggc acgggggcgc cggtgagggt
16801 ggagacgacc ggaagggtcg ggctccgaa ggcgaccgtt tccaccgtct tgaggaagtc
16861 gtcgagcatc gggtccatga gcggggagtg gaaggcgtgc gagacggaca gcctcttcgt
16921 cctgcggccg tcggccgaga gccggtcgac caccttcgtc acggcgtcct ccgcgcccga
16981 aaccaccacg gcggaggggc cgttgaccgc ggcgataccc gcacggccct cgaacccgga
17041 cagcaggtcc cgtacctcat cctccgaggc ctgcaccgcc accatcacgc ccccggccgg
17101 gagcgcctcc atcagacgcc cgcgcgccgc caccagggcc gccgcgtcct ccagtgagaa
17161 caccccggcc acatgggcgg ccgcgaactc gcccaccgag tgacccgcca ggaagtccgg
17221 ccggacaccc cacgactcca ccagccggaa caacgccacc tcgaccgcga acagcgcggg
17281 ctgcgcgtac gccgtccggt ccagcagggc ggcgtcctca ccgaacacca cacccgccag
17341 cggacgttcg aggtactggt ccacatgcgc gcacaccgcg tcgaaggcgt ccgcgaacac
17401 cgggaaggcc gcatacagct cacgccccat ccccagccgc tggctcccct gccccgggaa
17461 caggaacgcc gtacgacggt ccgtggcggc ggtgccacgc agtgccgggt ccgccggtcc
17521 ggtcgtgtcg cgcgccgcca gtgcgcgcag cgccggcagg aactccccac ggtcggtgcc
17581 gagcgccacc gcccggtgct ccagggcggc ccgggtcgtg gccagcgaca gggccacgtc
17641 gacgggttcg agttccgggt ggccctcgat gtgggcgagg aggcgttcgg cctggtcccg
17701 gagggccgcc gggctggttc cggagacgag ccacggcacg acgcccggcg cggggcgcgt
17761 cccggtgggc gccgcgggct gcggcggggc ctgctcgatg atgacgtggg cgttggtgcc
17821 gctcacgccg aaggaggaga ttccggcgcg ccggccgtgt tcgccgtcgg ccgtccagtc
17881 gacggccgag gtcaggagcc ggaccgaacc cgtcgaccag tcgacgtgcg gggtcggcgc
17941 gtccacgtgc agggtcccgg ggaggatgcc gtggtgcatg gcctgcacca tcttgatgat
18001 cccgcccact ccggcggcgg cctgggtgtg gccgatgttg gacttcaggg agcccagcca
18061 cagcggccgg ccctccggcc ggtcctggcc gtacgtggcc agcagcgcct gcgcctcgat
18121 cgggtccccg agccgggtgc cggtgccgtg cgcctccacc acgtccacgt ccgccgcggc
18181 cagtccggcg tcggcgaggg cctgcctgat cacgcgctgc tgcgccggac cgttgggcgc
18241 ggtcaggccg ttggaggcgc cgtcctggtt gacggccgat ccgcggacca ccgccagtac
18301 ccggtggccg ttgcggcggg cgtcggagag ccgctccacc agcaggaggc cgacgccctc
18361 gccccagccc gtgccgtcgg ccgcggccgc gaaggacttg cagcggccgt cggcggccag
18421 gccgcgctgg cggctgaact cgatgaaggc gttcgggctc gacatcacgg tcacgccgcc
18481 ggccagggcc aggtcgcact ctccgcgccg cagggcctgg gtggccaggt gcagggcgac
18541 cagcgaggag gagcacgccg tgtccaccgt cagtgtcggc ccttcgaggc cgagggagta
18601 ggcgatccgg ccggagacga cgctgccgga gcttccggtc ccgatgtagc cctccaggtc
18661 ctgcgggacg ctcagcaggc gcgccgcgta gtcctggtag ccggagccga tgaagacgcc
18721 ggtgctggtg ccccgtagcg aggtggggtc gagtccggcc cgttcgaagg cctcccagga
18781 ggtttccagg aggagtcgct gctgggggtc catggcgagg gcctcgcggg gcgagatccc
18841 gaagaactgc gggtcgaagt cggcggcgtc gtagaggaag ccgccgtcct tggcgtaggt
18901 ggtgccgggc cggtcggggt cggggtcgta gagcgactcc aggtcccagc cccggtcggc
18961 ggggaagccg ccgatcgcgt cacggccctc ggcgaccagg cgccagaggt cctcgggggt
19021 gcgtacgtcg cccgggtagc ggcagctcat ggcgacgatc gcgacgggct cgtcgtcccc
19081 ggaggtacgg gccgcggccg ttgcccgggc ggcgccggag ctctcgccgg tgtcggccgt
19141 cgtcagttcg gtcagcaggt gtgcggcgag cacggccggg gacgggtggt cgaagacgag
19201 ggtggcgggg aggtggacgc cggtggcctt gttcagccgg ttgcgcagtt cgacggcggt
19261 cagggagtcg aagcccagtt cgcggaaggc gcggtcggcg gcgaccgcgt ccgtgtcctg
19321 gtggccgagc acggccgccg cctcgccgcg tacgagcgcg aggaccgctc gggcccgctc
19381 gggcccggtc agggtgcggg tccggcggac gaactcggtc gcctcgcccc ggcccgcccc
19441 tcccggagcc ggatccgccg ccgctgccgt cagggcggcg gcggcctcgg ggacctccgc
19501 gatcagcggg ctgggccgca ggagggtgaa ggagggtgcg aaccgctccc aggcgacgtc
19561 ggcgaccgtc acggccgtct cgtcacggtc cagggctccg cgcagcgccg cgagcgccga
19621 cgccgggtcg agggcgggga ggccgcgctt gcgcagccgt tcctcggcgt cgccgtcctc
19681 gaccattccg ccgcccgccc acggccccca ggcgacggcg gtggcggtga gcccgcgcga
19741 ccggcggtgc tcggcgagcg cgtcgaggga ggcgttcgcc accgcgtacg cggcctgccc

19801 ggccgcgccc cacactccgg agatggagga gaagaggacg aaggcgtcca gcgggcggtc
19861 gccgagcagc tcgtccagat ggcgggcacc ggccgccttg gcggcgagga cgtccgcgaa
19921 ccgctccggc gtcgtctcgg ccagcatgcc gggcgtgccc actccggcgg cgtgtacgac
19981 ggcggtcagc gggtggtccc cgggcaggcc gtcgagcagc cgggcaaccg ctgtccggtc
20041 ggcgatgtcg caggcctcga tggtgacctc cgccccgcgt tcctccagtt cggcgcggag
20101 ggcgtcggcg cccgcggccg cttccccgcg ccggctggtc aggaccaggt gcgccgcgcc
20161 ggcgtccgcc agccagcggg ccacgtgagc gccgagggct ccggtgcctc cggtgatgag
20221 cacggtcccg cgcggccgcc acccggtggt cgtgccgtcc ggaccggccg ccgcccgcgg
20281 cgcgcgccgc agtcgtcgta cgaggactcc cgcggcccgg accgccagct ggtcctcggc
20341 cgcgtgggcc ccggcgagcg ccccggcgaa ccggcgtgcc gcccggtcgt ccagttcgcc
20401 gggcaggtcc agcaggccgc cccagcggtc ggagtgctcc agcgccgccg tgcggccgag
20461 tgcccacacc tgtgcctgga gcgggctgcg cagcgggtcc gaggggcccg tggacacggc
20521 accccgggtc acgcaccaca gggaggcgcc gggggcggcg tcgccgaggg cttggacgag
20581 cgccagggtc gcggcgagcc cttcggggag cgcggtgtgc acggggtgcg gccgctcgtc
20641 gacggcgagc agcgagagga ctccgccggc ggtggtgccg tccagtgcgg tccgcagccg
20701 gtcggcgagg tccgtccgcc cggtgcccgg ctccacgggt acggtgcgca cctcggcgcc
20761 gtggtcgcgc agggcccgta gcgcggacgc cacccagggg tcttcgtccg tcgggtgccc
20821 ggcgggcacc gcgacgagcc aggtgccggg taaccggatc tcggtggccg cggtcgcggc
20881 gggcttccag acggtgtggt agcgcaggcc gtccagggtg gaccgttcgc ggttcctgcg
20941 ccgccacatg gtgagggcgg ggagcaccga gctgagcggc gcgtcgtcgg ccacgtcgag
21001 ccgccgcgcg aacgcttcga ggtcggcgct ctccacggcc tgccagaatc cgtcctccgc
21061 cgggtccgcg gccccggtct cggtccgctg tccggagaag gccgtcccct ccagccagta
21121 ccggcggcgc tggaacgcgt aggtgggcag gtcggggagt acggggcccg gggcgccgag
21181 cagggcgttc cagtcgggcg tgacgccgtg gacgtgcagc cgggccagcg cggaggccac
21241 cgcgaggtcc tcgggacggt ccgggcgcag gaccggcagg gtcaccgccg gctgctcgtc
21301 gagcagtgcc tgggccaggg ccgtcagtac gccgccgggg ccgatctcca ggaacacgga
21361 ggcaccggcg tccgacaggg cggcgaccgt gtccgcgaag cgcacggccc ggacgacctg
21421 acgcacccag tacccggcgt cggccggctc cgcggcggtg atcgggcggc ccgtgaccgt
21481 ggacacgagt gcgatgtccg gctcggagaa ggtcacgccc tcggcgacct tgcggaacgc
21541 gtcgagcatc gggtccatga ggggtgagtg gaaggcgtgg gagacggtca gcctcttcgt
21601 cttgcggccg tcggccgaca gccggtcgac gacctccgcc accgcttcct cggcgcccga
21661 aaccaccacc gcggacgggc cgttgacagc ggcgatggcc acccggtcct cgtacccggc
21721 cagcaggccc cgtacctctt cctccgaggc ctccacggcc accatcacgc ccccggccgg
21781 gagcgcctgc atcagccgtc cccgagcggc caccagggcc gccgcgtcat cgagcgagaa
21841 caccccggcc acatgggcgg cggcgaactc gcccaccgaa tgcccggcca ggaggtccgg
21901 ccggacgccc caggactcca ccagccggaa cagggccacc tcgaccgcga acagggcggc
21961 ctgggcgtaa ccggtccggt tcagcagctc ggcgtcgtca ccgaacacca cctcccgcag
22021 gggccgttcc aggtgcgcgt ccagtgcgga gcagaccgcg tcgaaggcct cggtgaacac
22081 cgggagggcc gcgtacagtt cacgccccat ccccagccgc tggctcccct gtccggagaa
22141 caggaacgcc gccttcttct tccgggccgc cgtgccctcg aaggcgtggc ccgtcgcctc
22201 gtggcgggcg aaggacgcca gggcggtgac cagttcgtcg tggtcccggc ccaccacggc
22261 ggcacggtgc ggcagtccgg cccgcgtcgt cgccagcgca cgggcgatgt cggccgccgg
22321 gatccgcggg tgctcccgca cgtacgcgag gagtcggcgg gcctgttcgg gcagcgcctc
22381 ggcgctgcgg gccgagagcg gccacagcag gggcgtccgg gagtccggta cgggggcggg
22441 ggccgcagcc ccttccgtat cgggggcggc ctgttccagg acgacgtggg cgttggtacc
22501 gctgaccccg aaggaggaca ccccggcgcg gcgcggcccc tccccctcgg gccagtcgac
22561 cggctccgtc agcagccgga cggcgccggc cgaccagtcc acgtgcggcg agggctcgtc
22621 gaggtgcagg gtgcccggga gcaccccgtg gcgcatggcc atgaccatct tgatgacgcc
22681 cgcgacgccc gcggcggcct gggtgtggcc gatgttcgac ttgagcgcgc cgaggagcag
22741 cggccgcccg gccggccggt cccggccgta cgtggcgagg agcgcctggg cttcgatcgg
22801 gtcgccgagc gaggtgccgg tgccgtgcgc ctcgacggcg tccacgtccg ccccggtcag
22861 gccggcgttg gccagtgcct ggcggatgac cttctgctgc gcgggtccgc tggggggccgt
22921 caggccgttg gaggcgccgt cctggttgac ggcgctgccc cggaccacgg ccaggacgcg
22981 atggccgttg cggcgggcgt ccgagagccg ctccacgagc agcatgccga cgccctcgga
23041 ccagcccgtg ccgtcggcgg cggccgagaa cggcttgcac cggccgtcgg cggccagccc
23101 gcgctgccgg ctgaactcca tgaacgcgcc gggggtggac atgaccgtca caccgctgac
23161 cagggccagc gagcattcgt ccttgcggag cgcctgggcg gcgaggtgca gggcgacgag
23221 cgaggacgag cacgccgtgt cgacggtgac ggcgggtccc tcgaagccga aggtgtaggc
23281 gaggcgtccg gagatgacgg cggcggcgtt gcccgtgccc aggtatccgt cgaagtcctc
23341 cggggaggtg gacagccggg cgtagtcctg gccgttggtg cccgcgaaga ctccggtgcg
23401 gctgccgcgc agtgcggccg ggtcgatgcc tgcccgttcg aacgcctccc aggcggtctc
23461 caggagcagc cgctgctgcg ggtccatcat cagcgcctcg cgcggcgaga tgccgaagaa
23521 cccggcgtcg aactcgccga cgccgtcgag gaagccgccg tgccggacgt agcaggtgcc

23581 cgcgtggtcc gggtcggggt cgtacagggc gtccaggtcc cagccgcggt cggcggggaa
23641 gccggatatc gcgtcgccgc cgtccgccac cagctgccac agttcctcgg gcgaggtcac
23701 cccgccgggg aagcggcagc tcatcgccac gatggcgatg ggttcgtggg agtcggacgt
23761 cagtttccgg ttctgccggc gcagccgctc ggtctcctga agggaggccc gcagtgcctg
23821 aacgatcttc tcgttcgatg tggtcatcat gtgctccgta ctcaggccgt tgatcaggct
23881 tcgctgtcgc caagcgccag ttcgatgagg ttttcgaggt ccatctccgc gatcgcggag
23941 tcggcgtcgc catcggtgcc ggtgtcggtg gcgacgccgc cgggcgcgcc cggcgccagg
24001 gccagcaggg cgtccatcag cccggcttcc ctgaggcggg acagcgggat cgacgcgagg
24061 gtctgccgta cgcgggcttc gtccgggccc gcgtcggggt ggggtccgtc cgctcctccg
24121 ggaaacaggt cgccgagcag gcggtcggtg agcgcggtgg gggtgggatg gtcgaagacc
24181 atcgtcatcg ggagccgcag ccccgtcgcg gtcccgatcc ggttgcggag ttcgacggcg
24241 gtgagcgagt cgaagccgag gtcccggaag gcgcggtcgg gttcgacggc gtccgtgcgc
24301 gggtgtccga gtacggccgc ggcctccttc cggaccagtt ccagcagcag ctcgtggcgg
24361 tcctccgggg cgccggcggg gagcctccgt acgaggtcgg aggcggcgcc ctcgccccgg
24421 tcgccgccgg ggccgccgtc ggccccaccg ctgtcgaggg ccgcccgggc ctcgggggagg
24481 gcggcgagca gcgcgctcgg ccggccgatg gtgaaggacg gtgcgaacct cggccagtcg
24541 acgtccgcca cgaccacggt ggtctcgtcg cggtccatgg cgagttggag tgccgagacc
24601 gcgagggccg ggggcagcga gggcagtccg ctgcgccgca ggtactcctc ggctcccccg
24661 tcggcggcca tcccgccttc ggcccagggg ccccaggcga cggcggtccc ggccagtccc
24721 ctggcccggc ggtgctgggc gagcgcgtcg aggaaggcgt tgcccgcgga gtaggcggcc
24781 tggctgccgc tgccccacac gccggagacg gacgagaaga ggacgaaggc gtccagctcg
24841 cggtcggcga gcagttcgtc caggtggacc gcaccggcga ccttggcgga tacgacggcg
24901 gcgagttccg tcaggctcgt gtcggcgacg gggctgaact gcgggagtcc cgcggcgtgt
24961 acgacggcgg tcagcggtgc cgggtcgtcg gcgagcccgt ccagcagggc cgcgagggcc
25021 gcgcggtccg cgacgtcgca cgccgcgatc gtggtcctgg cccccagcgc cccgagttcg
25081 gcggcgagtt cgagcgcccc ctcggccgcc tcgccgcgcc tgctggtgag caccaggtgc
25141 tcggcgccgc cccgggccag ccagcgggcg gtgtgcccgc cgagcgagcc cgtgccgccg
25201 gtgatcagta cggttccgcg gggcttccag gcgccgtcgg taccgcccgt ggcgcggggc
25261 atccgggcca gcctgcgtac caggaccccg gaggcgcgca ccgcgagctg gtcttcgccg
25321 gcggcgtcgg cgagtacgtc ggccagccgg gcgagcgccc gcccgtcggg cgccgtgggg
25381 aggtcgacca ggccgcccca gcgctgcggg gtctccagcg ccacggcgcg cccgaggccc
25441 cacacgaggc tctggagcgg cctggtgacg ggctgggcgc cgctcaccga caccgcaccg
25501 gcggtgacgc accacagcgg ggcgtccggc tccagctccc ccagggcctg tacgagcgcg
25561 acggtggccg tcaggccggc cggcaggccg gggtgggtgg ggtggctccg ctcgtcgagc
25621 gccagcaggg acaggacgcc gctgacgggt cccgagtgct cggccaccgc gtcgcgcaac
25681 agctcgacga gcgccgagcg gtcggccgcc gggtcggcga cggtgacgca cagcggctcg
25741 gcgccccggt cgcgcagggt gcgggcgtag ggctcggcgg ccccggcgtc gggagccacg
25801 accagccagc tgcccggcag gggccggagg gacgggtcgg cgagcggctg ccacaccgtg
25861 tggtagcgcc agccgtcgag cgtggaacgg ttgcggctct cctcccgcca ggtggcgagc
25921 gcgtcgaaca ccgagtccag cggggcccct tccgccaggc ccagccgttc ggcgagggcc
25981 tcgggccgcc cgttctcgac cgcctcccag aactccgttt ccgccgggtt gtcggatccg
26041 gacggctggt gggccggcgc ggcgggggcg atggccgttt cgagccagta gtgctcgcgc
26101 tggaaggcgt aggtgggcag gtcgacgcgg cgggcgccgc gtccggcgag gacggccgac
26161 cagtcgacgg gcgtgccgtg gacgtggagc cgggcgaggg cggtggtgac ggccacctcc
26221 tcgggacggt ctgcacgcag gaccgggacc acggcggggc cgtcttcgag ggcggcctgg
26281 gccatggcgg tcaggacacc gcccggcccg atctccagga aggtacggac gcccagatcg
26341 gcgagggagg tgacggcgtc ggcgaagcgg acggcctcgc ggacgtggcg cacccagtac
26401 tcggcggagc agaactcctc cgcgggcacg gggcgccggg tgagggtgga gacgaccgga
26461 agggtcggga ctccgaagga gacgccctcg gcgaccttgc ggaagtcgtc gagcatggga
26521 tccatcagcg gggagtggaa ggcgtgcgag acggacagcg ccttcgtctt gcggccgtcg
26581 gcggcgagcc ggtcggcgac cttcgtcacg gcgtcctgcg caccggaaac caccacggcg
26641 gaggggccgt tgaccgcggc gatacccgca cggtcctcga acccggacag caggtcccgt
26701 acctcaccct ccgaggcctg caccgccacc atcacgcccc cggccgggag cgcctccatc
26761 agacgcccgc gcgccgccac cagggccgcc gcgtcctcca gtgagaacac cccggccaca
26821 tgggcggccg cgaactcgcc caccgagtga cccgcgaggg cgtccggccg gacaccccag
26881 gactccacca gccggaacaa cgccacctcg accgcgaaca acgcgggctg cgcgtacccc
26941 gtccggtcca gcagggcggc gtcctcaccg aacaccacac ccgccagcgg acgttcgagg
27001 tactggtcca catgcgcgca caccgcgtcg aaggcgtccg cgaacaccgg gaaggccgca
27061 tacagctcac gccccatccc cagccgctgg ctcccctgcc ccgggaacag gaacgccgta
27121 cgacggtccg ccgggcgggc gacgccccgt accaggtctg tcgccgtctc ccccttgctc
27181 agggcctcca ggccggccgc gagttcggcc gtggtccggc cgagcaccac cgcgcggtgc
27241 gtcatggccg tgcgggactc cgcgagggag agggcgaggt ccgccggggc gaggttcggg
27301 tggtccgagg tgtgggccag gaggttgcgt gcctgggccc gcagggacgc ttcgtccttg

27361 ccggacagcg tccacggcag gcagtgcccc gccgcaggcg tcccgtgctc cccctcttcg
27421 gtgggcaccg tggccggggc ctcctcgatg acggtgtggg cgttggtgcc gctcaccccg
27481 aaggaggaga ccgccgcccg gcgcgggcgg ccgttccccg tccaggtcac gggttcggtg
27541 agcaggcgta ccgcgcccgc cgaccagtcg acgtgcggcg agggctcgtc cacgtgcagg
27601 gtcttcggca gcatgccgtg ccggagcgcc atcacggtct tgatcacgct cgccacgccg
27661 gaggcggcct gggtgtggcc gatgttcgac ttgagggaac ccagccacag cggccggtcg
27721 tccggccggt cctgcccgta cgtggccagc agcgcctgcg cctcgatcgg gtcgccgagc
27781 gcggtgccgg tgccgtgggc ctcgaccgcg tccacgtcgg cggtcgacag acccgcgttc
27841 gcgagcgcct gccggatcac gcgctgctgc gccgggccgt tcggggcggt gaggccgttc
27901 gaggcgccgt cctggttgac ggcgctgccc cgtacgaccg cgagtaccgg gtggccgttg
27961 cgctgtgcgt ccgagagccg ctcgaccagc agcatgccga cgccctcgcc ccagccggtg
28021 ccgtcggccg cggccgcgaa cggcttgcag cggccgtccg cggccaggcc gcgctggcgg
28081 ctgaactcga cgaaggcgct ggggctcgac atcacggccg cgccgccgac cagcgccagc
28141 tcgcactcgc cctggcgcag ggcctgcgtg gccaggtgca gggcgaccag cgaggaggag
28201 caggcggtgt ccaccgtcat ggcgggcccc tccaggccga gcgcgtagga gatgcggccg
28261 gacatgacgc tgctggaggc gccggtgagc aggtgcccct cgaccccgtc gggcaggtcg
28321 ccgggggcccg cgccgtatcc ctggtagccg gagccgatga agacgccggt gcgggtgccg
28381 cgtacggacg agggcgcgag ccgggcccgc tccagggctt cccaggccgc ttccaggagc
28441 agtcgctgct gcgggtccat ggcgagggcc tcacgcgggg agatcccgaa gaacgtggag
28501 tcgaagtggc cggcgtcgta gaggaacgcg ccctcgttga cgtaggtggt gccggggtgt
28561 tcggggtccg ggtcgtagag ggtgtcgagg ttccagccgc ggtcggcggg gaacgcggac
28621 acggtgtcca cgccgtccac gagcagctgc cacagctgtt cgggcgagtg cgcgccgccc
28681 gggaagcggc agctcatggc gacgatcgcg atgggttcgt cgggggtcgc cgccgggcgc
28741 tcccccgggg tggcggcatc ggcgcggacg gtggcggcgc cgtggccggg gagctccgag
28801 gtgaggtggt cggcgagggc gtcggcgttg ggtggtcga agaccagggt ggtcggcagc
28861 cgtagcccgg tggccgcgtt cagccggttt cggagttcga cggcggtgag cgagtcgaag
28921 ccgaggtcct tgaaggaccg gtcggatgcc accgcgtccc ggcccgggtg tccgagcgcc
28981 ctggccacct cggtcctgac gaggtcgagc agggcccggc ggcgttccgg ttcggcgagg
29041 gccgcgagtc cggcgccgag ggagggcgcc gtggtgtcgg gccgaccgtc gccgcccgtg
29101 gccgacccgg ttccggcgag ggcctggcgg gcctccggca ggtcggcgag gagtggggcc
29161 gggcgggcga tggtgaaggc gggtgcgaac cggtcccagt cgacgtcggc gacgacgacg
29221 gtggtctcgt cgtggttcag ggcccgctgg agcgcggcga tcgcggtgtc cggggacatc
29281 ggcggcagtc cgcgccggcg caggtgggct tccgcttcgt cgtcgtccgc catgccgccg
29341 tcggcccagg gtccccaggc caccgatgtg gcggcgaggc cgtcggcgcg gcggcgctcg
29401 gcgagtccgt cgaggaaggc gttggcggcc gcgtaggcgg cctgtcggcc gctgccccac
29461 actccggcca ccgacgagaa gaggacgaag gcgtccagcg gccggtcgcc gagcagttcg
29521 tcgaggtggc tggcgcccgc caccttggcg gagaccacct ccgcgaagtc gccgaggtcc
29581 aggctgtcgg tgggggcgaa ctgcggcatc cctgccgcgt ggacgaccgc cgtcagcgga
29641 tgccggtcct cgggcacaga ccggagcagg gcggcgacct cctcgcggtc ggacacgtcg
29701 caggccacga gggtgacctc ggcccccagt gcctcgatct cttcggcgat ctgcgccgcg
29761 ccgggggcgt cggggccccg ccgtccggtc agtacgacgt gttcggcgcc gcgtgcggcc
29821 aggtaccgcg ccacccgtgc gcccagcgcg ccggtgcctc cggtgatcag tacggtgccg
29881 cgcggctgcc aggccccgtc ggccggtacg gcgccggtga gggcgcgcgc cagacgtcgc
29941 acgaagatcc cggacggccg gatggcgatc tggtcatcgg caggagcggc ggatgcgtgt
30001 gcgggtgcgg acagtacgga cgccaagcgg cgggccgtcc gttcgtcggg cgcaccggcc
30061 aggtcgacca gtccgcccca gcgctccggg aactccaggg ccaccgagcg gccgaggccc
30121 cagaccatgc cctggccggg gctggtcagc gggtccgacc ggccgaccgt aacggctccc
30181 gtggtgacgc accagagcgg tgcggagatc tcggcgtcgc ccagcgcctg gacgagcagg
30241 gtggtctggg cgaggccggt gggcagctcc ttgtgtacgg ggtgcggctc ctcggcgagg
30301 gcgagcagcg acaggacccc ggccggcgcg gtgtcaccgg gctgcgcgcg caggcgcgcg
30361 gccagcgccg cgcggtcctc gtcggcccgt acgaccagcg gtacgggttc ggcgccttgg
30421 cgggacaggg cggccgtgac ggcggcgacg gtcggggcgt cctcggcgcc cgccgggacg
30481 gcgagcagcc aggcaccggt cagctcggcc gcctcacggc cggtgagcgg cttccacgag
30541 gtccggtagc gcagtgcgtc gacgacggaa gcgtcccgct gccggcgccg ccaccgggac
30601 agtgccggga gtacggagct cagcggggag tccccgtcga ggtccaactg ctcggtgagc
30661 gaggccaggt cctggctctc cacggcttcc cagaacccgg cgtccgccgg gtcggccgag
30721 aggggtccgg tctcctgcgg gtcggcgggg agcaaccagt agggcgtgcg ccggaaggcg
30781 taggtgggga ggtccgtgcg gcgggcgccg cgcccggcga agacgcccgt ccagtcgacg
30841 gggacgccgt ggacgtggag gcgggcgagg gcggtggtga cggccaggtc ctcggcgcgg
30901 tcggtccgca ggagcggaac gctgacggcg tgctcgtcca gggagtcctg ggccatggag
30961 gtgagcgctc cgcccggggcc gacctccagg aaggtgcgga cgcccagatc ggcgagggag
31021 gtgacggcgt cggcgaagcg gacggcctcg cggacgtggc gcacccagta gtcggcggag
31081 cagaactcct ccgcgggcac gggggcgccg gtgagggtgg agacgagcgg aacggtcggg

31141 tctccgaagg agacgccctc ggcgaccttg cggaagtcgt cgagcatcgg gtccatcagg
31201 ggcgagtgga aggcgtggga gacggtcagc ctcttcgtct tgcggccgtc ggcagagaga
31261 cggtcgacca gtgcggccac cgcttcctcg gcgcccgaaa ccaccacaga agagggcccg
31321 ttgacggcgg cgacgcccgc gcggtcctcg tacccggaca gcagttcccg tacttcttcc
31381 tccgaggcct gcaccgcgac catcacgccc ccggacggga gcgcctgcat cagccggcca
31441 cgggccgcga ccagggccgc cgcgtcctcc agcgagaaca cacccgccac atgggcagcc
31501 gcgaactcgc ccaccgaatg accggccagg aagtccggcc ggacacccca cgactccacc
31561 aggcggaaca acgccacctc gacggcgaac aaggcgggct gcgcgaaacc ggtctggttc
31621 agcagcccgg cgtcgccccc gaacaccact tccttcagcg gccgttcgag gtgcgcgtcc
31681 agcgcggcgc acacggcgtc gaaagccccg gcgaacaccg ggaaggccgc atacagttca
31741 cgccccatcc ccggccgctg gctcccctgc cccgagaaca ggaacgcggt ccggctgccg
31801 ctcgcggtcg ccgttccccg caccgtctcc gtgccggagg gctcgccgtg tgccagggcg
31861 gtcagcccct ggaccagcag ttcgcggtcc gccccgatga ccgccgcgcg gtgcgggaag
31921 gcggcgcgcg tggtcgccag ggagaaggcg atgtcggtct cgtcggtgcc atgggcttcc
31981 gaccggacgt gcccgagcag gcgttccgcc tgggcccgca ggccggtttc gtccttcgcg
32041 gagatcagcc aggggaccgc cggccgcggc cccgtctcct cccccggacc gtggttcgtg
32101 ccgtcggccc cttcggcggg cgcctgctcg atgatgacgt gcgcgttggt gccgctgacg
32161 ccgaaggagg acacgcccgc ccggcgcgga cgcccgtgct ccgcccactc caccggctcg
32221 gtcagcagcc gcaccgcacc cgccgaccag tcgacgtgcg gcgacggctc gtccacgtgc
32281 agcgtccggg gcaggacgcc gtggcgcatc gccatcacca tcttgatcac gcccgcgacg
32341 ccggccgcgg cctgggtgtg accgatgttg gacttgaccg tgcccagcca cagcgggcgg
32401 tcggccgggc ggtcgttgcc gtaggacttc agcagggcct gcgcctcgat cggtcgccg
32461 agcgtcgttc ccgttccgtg ggcctcgacc gcgtcgacgt cggcggccgt caggcccgcg
32521 tccgtcaggg cctgacggac gacgcgctgc tgcgcggccc cgttcggggc ggtcaggccg
32581 ttggacgcgc cgtcctggtt gatcgccgaa ccccgtacca cggccagcac ccggtgcccg
32641 ttgcggcggg cgtccgagag gcgttccacc agcagcatgc ccgcgccctc ggccagaccg
32701 gtgccgtcgg ccgcggccga gaaggcgcgg cagcgggcgt cgtgggagag cccgcgctgg
32761 cggctgaagc cgatcagtgc gcccggggtc gacatgacgg tgacgccgcc ggccagggcc
32821 aggtcgcact cgccccggcg cagggactgc gcggccaggt gcagggcgac gagggaggag
32881 gagcacgcgg tgtccaccgt caccgccggc ccctccagac cgaaggtgta ggagagccgg
32941 cccgagacga cggaggcgga gttggcggtc aggaggtggc cctccatgtc ctcggtccgg
33001 tgctgggaga ggccgacggc gtagtcctgg ccgttggtgc ccgcgaagac tccggtgcgg
33061 ctgccgcgca gtgcggccgg gtcgatgcct gcccgctcga acgcctccca ggcggtctcc
33121 aggaggagcc gctgctgcgg gtccatggag agggcctcgc gcggggagat cccgaagaat
33181 tccgggtcga agtcgcccgc gtcgtgcaag aacccgcctt cgcggacgta gctggtgccg
33241 gggtggtcgg ggtccgggtc gtacagcgcg tcgaggtccc agcctcggtt gacgggcagg
33301 ccggtgatgg cgtcggtgcc gttggccacg aggtcccaga ggtcctcggg gccgcggacc
33361 tcgccgggca gccggcagct catcgcgacg atggcgatgg gctcgtcggt gtccgtaccg
33421 gggaggcggc cgggggtggt ggaggcgtcg ggcgcggccg tgacgagggc cgtgccgggg
33481 tggccgccgg ggatctcgtc caggaggtgg ccggcgaggg ccgcgggggt ggggtggtcg
33541 aagacggcgg tggcgggcag ccgcactccg gtggcggtgg cgagccggtt gcggagttcc
33601 atcgcggcca gggagtcgaa acccagttcg cggaaggccc ggccggggtc gatggcctcg
33661 ggggccgagt ggccgagtac ggcggcgacc tggttgcgta cgagggcgag gacggtgtcc
33721 cgccgttcgg cgggcggcag cgcggccagc cgggagagtt ccgcggagtg gccgccgggc
33781 ccgggggcgg tctccgcgga acgccgggcg gggtgcggga tcaggccgcg cagcaggtgc
33841 ggtaccgggt gcgccgcggc ctgggcgcgc atggcggcca ggtcgaggcg tacgggggcc
33901 agggccgcgt cgtccagggt ccgggcggtg tcgaggagtc gcaggccttc ttccgcgccg
33961 agcgggacga gtcctgcgcg ggccatccgg cgcaggtcgc ccgtctccag gcgggtctgg
34021 tcgccgcgct cgtcccagac gccccaggcg agggaggagg cggggaggcc ctgggcccgg
34081 cgctggtggg cgagggcgtc caggaaggcg ttgcccgccg cgtaggcggc ctgtccggcg
34141 ccgccgaggg taccggcgaa ggaggagaag aggacgaagg ccgagaggtc caggtcgcgg
34201 gtgagggcgt ggaggttgag tgcggcgtcg accttgggcc gcaggacctg gtggagttct
34261 tccggggtga gggtgtcgac gagtcccccg gccaccacgg ccgcagcgtg gacgacggcg
34321 gtgagcggcc ggtcggcggg gagggtggcg aggagggccg ccaggcctc gcggtcggcg
34381 gcgtcacagg cggcgatggt gacggtggcg ccgagcgcgg cgagttcctc gcgcagtgcg
34441 tcggccccgt cggcgtcggg gccccgccga cccgtgagga cgatgttgcg gacgccgtgg
34501 gcggtgacga ggtgcgcggc gacgagccgg ccgagcgcgc cggtgcctcc ggtgatcagt
34561 gcggtgccat gggggtccag ggcgcgggcg ggggcggttc cggcggtgac gggtacccgg
34621 gccagccggg gtacggacat ccggccctcg cgcagggcca gttggggttc tccggtggcg
34681 acggcggcgg cgaggcggcg cagggattcg ggctcgccgt cggtgtcggc caggaccagg
34741 cggtccgcgt gctcggactg ggcggagcgg atcaggcccc agacgggggc gtgcgcgggc
34801 cgtgtccgcg gtacgcggtc ctcggctccg gacgggaccg ggacggcgtc ccgggtgagg
34861 acgaccagcc gggaggagac gaaccgctcg tcggccagcc aggcctggac aagggcgagg

34921 gtggcccgcg cggtggcccg gaccgtgtcg gcgtcggagg cgtcggaggc gtcggaagcg
34981 gcgacgggaa cgacggcggc cacggtccgg ggcaccgggg cgccctccgt gatcgccgcg
35041 gcgagggcgg ccaggtccgg gtggacgtca ggccgcgggc ccgcgccgcg cagggcgtcg
35101 gcgaccgcgt cggcgtccgc ccgcggggtg acgccgacga cggcccagtg ggtgctgtcg
35161 tcgggcgcgg tgcccgccgg cggcagggtg ggccagtcca cgcggtagag ggactcgtgg
35221 tggtcggtga gcgcggcccg caggtgttcc tcgcggacct cgcggaaggt gagcgcggcg
35281 acggtggcca cggcccggcc gctgtcgtcc gtggcggtga cggccacggc ctcggcgccg
35341 accggtgtca gccggatccg cagccgggtg gctccggtgg cgtacaggcg tacgtcctcc
35401 caggagaagg ccatgcgccc ggtccccatg tccccgagcg ggccgaagcc caccccgtgg
35461 agggcagcgt cgagcagggc cgggtgcagc ccgtacctcg cggcgtcgga cggggcggag
35521 gcgtccgggg gcaggacggc ctcggcgaag acctcgtcgc cgaggcgcca ggcggcgcgc
35581 aggccctgga aggcggggcc gtaggcgaag gagcgggcgg cgagctcctg gtagaagccg
35641 ccgaggtcga cgggggccgc gccggcggga ggccaggcgg ccggggccgg tacggggggc
35701 cggtcctcgg cggccgaggt cagcacgccg gtgccgtggc aggtccagcc gtcctcgggc
35761 gccgcggtgt cggggcgcga gtggatggtg aaggtccgct cgcccgttcc ctcggccggg
35821 ccgaccgcga cctggacgac gacgccgccc cgctcgggca ggaccagcgg cgcctggagg
35881 gtgagttcgg cgaggcggtc gcagccgacg tggtcgcccg cgcggaccgc gagttccacg
35941 aaggccgtac ccggcaggac aaccgcgccc agcacgacgt ggtcggcgag ccatgggtgg
36001 gtgtgcgctg ccagccggcc ggtcaggagc agtccgtcgg agtcggcgag gctgacgacg
36061 gcgcccagca gcgggtggtc ggaggaggcc aggccgaggc ccgtggcgtc cgcggcggcg
36121 ggcggggcgt cgagccagta ccgctcgtgc tggaacgcgt aggtgggcag tgcgacccgg
36181 cgggcgccgg tgccggagag gacggccgcc cagtcgacgg cgacgccgtg ggcgtgggcc
36241 tcggcggcgg agcgcaggaa gcggccgggg ccgccctcgc cgcggcgcag ggtgccgagg
36301 gtgacggccc cggcgccgag gtcgtcgagg gtttcctgga tgccgatggt cagtacgggg
36361 tgcgcgctga cctcgatgag ggcgtcgtgc ccgtcccgta cgaggcgcgg ggtggcctcc
36421 tcgaaccgga cggtccggcg caggctgcgg taccagtact cggcgtcgag cccgtcggtc
36481 tccacgaccg taccggtgac cgtggagtag aacggcacgt cgcacgcctg cggggtgatg
36541 ccggcgagcg cgtcgaggac gcgttcgcgg accgcctcga cgtgcgcgga gtgggaggcg
36601 tagtcgacgg cgatcagccg ggcccggacg tcgtcggcgt gcagggcgtc gcgcagttcg
36661 gtcagggcgt cgcggtcgcc cgagacgacg atcgacgtcg ggctgttcac ggcggcgacg
36721 gacagccgct cgccccagtg cgccatccgg ggtgtgaggt cctccagcgg gagggcgacg
36781 gacagcatcc cgccccgccc ggagagcgcg gtcagtgcct ggctgcgcag cgccaccacc
36841 ttcgcggcgt ccgccaggga gagcgcgccg acgacgcacg cggcggcgat ctcgccctgg
36901 ctgtggccgg cgaccgccga ggggtggacg ccgtgggagc gccacagttc ggccagcgag
36961 accatgacgg cgaacagcac gggctggacg acgtcgaccc ggtcgaagcc gggggcgccg
37021 ggcgcgccgc gcaggacctc ggtcggcgac cagtcgacgt ggggtgcgag ggcctcctcg
37081 cactggcgga agcgctcgcg gaagaccggt gcggcgtcga ggagttcgac ggccatgccg
37141 gcccattggg agccctggcc ggggaacacg aaggcgacct gccgggtggc gtcggaggtg
37201 ccgcgcacga gggcggcgcc ggcggcgtcg ggttccccgt cggcgagggc gtccagggcc
37261 cggtcgagcc cggcccggtc cggggcgagg agcacggcgc ggtgggggaa ggcggtacgg
37321 gtggtggcga gggagtacgc gatgtcgagg gcgtcggggt cggcggccgg accggcgtcg
37381 gggtcggcgg ccggacccgc ggcggggtag gcggccggac ccgcggcggg gtaggcggcc
37441 ggacccgcgg cggggtaggc ggccggaccc gcggcggggt aggcggccgg accggccgcg
37501 gggtccgtcg ccgggtccgg gccggggcgc ggggcggcga ggtgggagcg gagccgggcg
37561 gcctgtgccg tgagggcggc ggcggtcttg ccggagaggg tccaggggac gagggggagc
37621 ccgttcccgt ccgccgtcgc cgtgccgggc gcgccggatg gcccgtcggc ggccggagcc
37681 gccacggacg gcgcgtcggg ccccgcgtcg tccatggccg cggcttcggg ccgggccgcg
37741 ggcgcgaggt gctgttcgac gacgatgtgg gcgttggtgc cgctgacgcc gaaggcggag
37801 accccggccc gccggggcct gccggtgtcg ggccaggcgg cgttctcggt gaccagcgag
37861 acggccccgg cggaccagtc cacgtgcggg gtcggctctt cggcgtgcag ggtcttcggc
37921 agcacgccgt ggcgcagcgc ctgcaccatc ttgatgatcc cggcgacgcc ggccgccgcc
37981 tgggtgtgtc ccaggttgga cttgacggag ccgagccaca gcggccggtc cgccgggcgg
38041 tcctggccgt acgtcgcgag gagcgcctgg gcttcgatcg ggtcgccgag ggtggtgccg
38101 gtgccgtggg cctcgacgat gtccacgtcg gacggggaca gcagggcgtt ggccagggcc
38161 tggtggatga cgcgctgctg ggagggggcg ttgggcgcgg tcaggccgtt gctggcgccg
38221 tcctggttga cggcggagcc ccggacgacg gccagcaccg ggtggccgag gcggaccgcg
38281 tcggagagcc gttcgacgag cagcatgccg acgccctcgg accagccggt gccctcggcc
38341 gacgcggaga acgcccggca ccggccgtcg ggcgacaggc cgcgctggcg gctgaactcg
38401 acgaacgtcg tcggaccggc catcacggtc acgccacccg cgagggccag gtcgcattcg
38461 ccctggcgca gggcctggac ggcgaggtgg agtgccacga gggaggagga gcaggcggtg
38521 tcgatggtca cggcggggcc ttcgagcccc agggtgtagg cgatgcggcc ggacaccacg
38581 ctgccggtgt tgccggtgct gacgtatccc tcgatgcctt cgggcatcgc cgggagccgg
38641 gagacgtagt cgtggtacat gactccggcg aacaccccgg tgcggctgcc gcgcagggac

38701 agcgggtcca gttcggcgcg ttccaccgcc tcccaggagg cctccaggac caggcgctgc
38761 tgcgggtcca tggcgagggc ctcgcggggg ccgatcccga agaaggcggg gtcgaactcg
38821 gcggcgtcgt agaggaatcc gccttccttg gcgtagctgg tacccgggtt ctcggggtcc
38881 gggtcgtaga gcgcgtcgag gtcccagccc cggtcggtgg ggaagcccga tatcgcgtcg
38941 cggcccgagg cgaccaggtc ccacaggtcc tcgggggagc ggacgccgcc ggggaagcgg
39001 cagctcatgc ccacgatcgc gatcggttcg cggttcttgc cctcggcctc gcgcagccgc
39061 cggtgcgcgt gacgcagatc gacggtggca cgcttgaggt agtcgcggag tttgttcaca
39121 tctgtcgtca tctgggttct gttcctcaac gcgaagggat gtcgtgctgc tcgatcagtg
39181 ggtgccgagc tcgccgtcga gcagttcgaa gagctcctcg tcggaggcct cctcgacctc
39241 ggtcccggag tcgtcgtccc cgggcccgtg gccggtgtcg tgggcggcct cgcgccactt
39301 ggcgagcagt acctccagcc gcagggccgc ggtggcccgt gttccccggt cgtacgggga
39361 gcgtgcgaac gcggcttcca ggcggtcgat ctcggagagc agcgaggggg ccggctccgc
39421 tccgccgggt cctgccgcgg gcagcgcggc ggacaggtgt ccggcgaggt cggccggggt
39481 cgggtagtcg aacagcaggc tcgccggaag gcgcagcccg gtgatcgcgc cgaggtggtt
39541 gcgcagttcg acggcggtga gcgagtcgaa gccgaggtcg cggaaggcct gtgcggcgcc
39601 gatcgcctcg gaggaggcgt gccccagcac ggtggcgacc tgggtccgga ccacgccgag
39661 gaggatccgc cggcgcttct cttccgatgc cccggccaat tgcccggcga gggcggccgt
39721 cccgggcgtg ggttcccggg ccgggcgggc cggggcccgg accaggccgt gcagcagggc
39781 gtggaccggc tcggtcgtgg cgcgtcggcg cagggcgggc aggttcagcc gtaccggcag
39841 gagcacggcg tccggtgcgg tgccgggggt gtcggcggcg gtcccggcgg tcggggtttc
39901 cgcggtgggg ggcgcgctcg ccgtgtcgaa gagggcgagg ccctcctccg gggagagcgg
39961 cgacaccccg gcgcgcgcca tccgcgcgag ggctcccgct ccggccccgc cgagcatgcc
40021 ccgcatcccc ccggcctccg cccaggggcc ccaggccagc gagacggcgg ggagtccggc
40081 ggcccggcgc gactgcgcga gggcgtcgag gaaggcgttg gcggcgccgt agttggcctg
40141 gcccgcgttg ccgaacgtgc ccgcgagcga ggagaacagg acgaacgccg cgaggtccgc
40201 gtcccgggtc agttcgtgca gatggagggc cgcgtccgcc ttgggtccga ggacgtcgtc
40261 gagccggcgg gcggtcagcg agccgacggg cgcgtcgtcg agcacaccgg cggcgtgcac
40321 caccgcggtc agcgggtggg cggccgggat gccgtggagc acggcggcga gcgcgtcgcg
40381 gtccgccgcg tcgcaggcga gcgtggccac ttcggcgccc agcgccgcca gttcggcggc
40441 gaaggcgccc gcgccgtcgg cgtcgggtcc ccggcggctg gtcagcacca ggtgccgtac
40501 gccgtgcgcc gccaccaggt gccgcgcgac gagccggccc agggcgcccg tcgcaccggt
40561 caccaggacc gtgcccgccg ggtccagccc gagggacggg gtcggcccgg gggccccggc
40621 gccggtcgcg cgggccagcc tcggcacgaa cgcccggccg ttgcggaggg cggactgcgg
40681 ctcgccggtg gcgagcacgg ccgtcagccc ttccggctgc gcccggtccg cgtggtcggc
40741 gtccaccagg acgaagcgtt cggggtgctc ggcctggacg gacctgacca gcccccacac
40801 ggcggcctgt gccgggtcca gccgcgccgc gtccccggcg gtcgcggcga ccgcgttgcg
40861 ggtgacgatg acgagccccc gcgcgtccgt acccgtatcg gcgtcggctt cggcttcggc
40921 gtccgccagc cattcctgta cgagggccag ggcgcggccg gccacggacc gtacggcccc
40981 ggcctccgac gggccgtccg tcgcgaacag caccacccga tcgccggcgg gcggcgcgtc
41041 cggtgtccgg ggcaggggca gcggggtcca gtcgacccgg aacagcgagt cccgggaccc
41101 gcccgcccgg gcggcggcga gctgttcggc ggagaccggg cgcaggacga gcgagtcgac
41161 ggaggtccgg atccggccgg tcgggtcggc ggccagcagc gagaccgtgt caggaccggc
41221 gggggtcagc cggatccgca gggcctccac cgctcccgcc gacggcgcgg ggtgcgccgt
41281 caccccgctc caggagaacg ggatccgggc ccggtcaccg gcatcggcgt cggggccgag
41341 caggccgggg tcgagggcga cggtgtgcag ggccgcgtcg agcagggccg ggtgcaaccc
41401 gaagccggac acgtcggtcc cctcggggag ttccacctcg gcgaagagcg cgtcctcccg
41461 cgtccacgcg gcgcgcaccc cccggaacac ggggccgtag tcgagcccca ggccggccat
41521 ccgcccgtac agggagtcca gcgcctcctc ggggacggcg accgcctccg acgggggcca
41581 gtcggccagg gcggccgcat cggcggtggc gtcggcggtg gcctcggcgg tcgacagcgt
41641 tccggcggcg tggcgcgtcc acggctcgtc ggcaggggcg tcctcggtgc gggagtggat
41701 ctccagcggg cggcggccgg ccccgtcggg ggacccgacg cgcacctgga tccttaccgc
41761 ggtgtcgtcc ggcaggacga gcggggcccc gagcgtcagc tcgtccagcc ggtcgcaccc
41821 gacggcctcg tcgagggccg cgcgccgggc gaggtcgacc agggcggtgc ccgggagcag
41881 cacggcgccc atcacggcgt ggtcggcgag ccacgggtgg gtcgcgggcg acagccgccc
41941 ggtcaggacc agcccgtcgc cgtcggcgag cggaacggcg gccccgagca gggcgtggcc
42001 cgcctcggcc agtccggcgg cggcgacgtt cccggatgcc ggggcggatt cgagccagta
42061 gcgggtgcgt tggaaggggt aggtgggaag gtccacgcgc cggaggccgt gtccggcgaa
42121 gacggcggtc cagtcgacgg gcgtcccgtg ggcgtgcagg tgggcgaggg cggtggtgac
42181 ggccaggccc tcgggccggt cggtccgcag caggggggacg gtgacggcgt gctcgtccag
42241 ggagtcctgg gccatcgcgg tcaggacccc gcccggaccg atctccagga aggtacccac
42301 gcccacgtcg gcgagggcgg agacggcgtc ggcgaagcgg acggcctcgc ggacgtggcg
42361 cacccagtag ccgaccgagc agaactcctc ggccggcacc agggcgccgg tgagggtgga
42421 gacgaccggc aggcgcgggg cggcgaaggt gaccgtctcc accacctggc ggaagtcgtc

42481 gagcatcggg tccatcaggg gcgagtggaa cgcgtgggag acggacagcg gcttcgtctt
42541 gcgaccttgc gcctccaacc gttcgacgac cgcggaaacc gcgtcccgcg taccggaaac
42601 caccatggag gaaggcccgt tgacggcggc gacgtcggcc tgctccgggt ggccggccag
42661 cagttcccgc acctcctcct cggaggcctg taccgcggcc atcacgccgc cgaaggggag
42721 cgcctgcatc agccggccgc gagccgcgac cagagccgcc gcgtcctcca gcgagagcgc
42781 gcccgccaca tgggctgcgg cgaactcgcc caccgaatga cccgcgagca ggtccggccg
42841 gacgccccag gactccacca gccggtacgc cgcgacctcg accgcgaaga gcgccggctg
42901 ggcgtggtcc gtccggtcga ggagctccgc gtcgtctccg aacaccacgg tgcgcagagg
42961 gtgttcgaga tgcgggtcca gtgcggcaca ggcctcgtcg aaggcctcgg cgaacaccgg
43021 gaaggcggcg tgcagttcgc gtcccatccc gagccgctgg ctcccctgcc cggagaagag
43081 gaacgccctc ttcctgccgg gctcggcgac ggcccgcacc accgatcgtg ccgggtcgcc
43141 gtgggcgagg gctgccagcc cctccaggac gccggccgtg tccggtgcca cgacggcggc
43201 gcggtgttcc agggcggtgc ggctcgtggc cagggagtag gcgatttcgg ccgcctcgcg
43261 gtcgccgtcg cgttccggta cgtgttcccg cgcgtgctcc aggagccgcc gcgcctgctc
43321 gcgcaaggcc tccgggctct tcgcggacag cagccagggc gccgtgaacg gggtgcgggg
43381 cttcgttcgc gcagcgggcg cggtccggga gacggcaggc gcctgttcga ggacgacgtg
43441 ggcgttggtc ccgctgaagc cgaaggagga gaccgccgcc cggcgggggc ggccgtcctc
43501 tccccaggcg acgggttcgg tgagcaggcg taccgtcccg tccgaccagt ccacgtgcgg
43561 cgagggctcc tccgcgtgca gggtcctggg caggacgccg tggcgcatcg cctcgaccat
43621 cttgatgatc ccgcccaccc cggccgccgc ctgggtgtgc ccgatgttcg acttcaacga
43681 ccccagccac aaaggccgct cggccgaacg cccccgcccg tacgtcgcca gcaacgcctg
43741 cgcctcgatc ggatcaccca gccgcgtccc cgtcccgtgc gcctccacca catccacatc
43801 ggctggagac agtccggcac ccgcaagggc ctgttcgatc acccgctgct gcgccggacc
43861 gttcggagcc gtcagaccat tcgaagcacc gtcctgattg accgcactcc cccgcaccac
43921 cgcaagcacc ggatgcccca gacgccgcgc atccgacaga cgctccacca acagcatccc
43981 gacaccttcc gcccacccgg tgccgtccgc gttcgcggag aacgccttgc agcggccgtc
44041 ggcggccagc ccgcgctggc ggctgaactc cacgaaggct cccgggcctg ccatgacggt
44101 cacaccaccc gccagggcca gttcgcactc gccctgccgc agggcctgga ccgccaggtg
44161 cagggcgacc agggaggagg agcatgcggt gtcgacggtc accgcggggc cttcgaggcc
44221 gaaggtgtag gagaggcggc cggagatgac gctcccggag ttgccggtgc cgaggaagcc
44281 ctcgaccccc tcgggcaccg accgcagggc ggaggcgtag tcgtggtaca tcacgcccgt
44341 gaagaccccc gtacgggagc cgcgcagcgt ggcggggtcg atcccggccc gctcgaacgc
44401 ctcccacgag gtctccagga gcagccgctg ctgcgggtcc atggcgaggg cctcacgcgg
44461 cgagatcccg aagaacgcgg cgtcgaactc ccggcgtcg tagaggaatc cgcccccggcg
44521 ggtggtgctg gagccggtgg cgccgtctgc ggcgaggagg tcgaggtccc agccccggtc
44581 gacggggaag tcggagatgg cgtcggtgcc cgtggcgagc agccgccaca ggtcctcggg
44641 ggaccgcacg ccgcccgggt agcggcagct catggcgacg atggcgaggg gctcgtcgga
44701 gtcggcggcg cccccggccc gggtggccgc ccggacggtg ccggccggtc gggctccgtc
44761 gagttgaccg agcagatggc gggcgagcgc gtccggggtc gggtagtcga agaccagccc
44821 ggccggcagc cgggcgccgg tcgccgagac gagtcggttg cgcagttcca cggccgtgag
44881 cgagtcgaag ccgagctccc ggaaggcctg ggtcgcctgg acggttgcgg gccggtcgtg
44941 gcccagtacg gcggccgcgt ggccacgcac gaggtcgacg agcagatcgg tccgcgccgc
45001 ctccgtcagc ccccgcagcc gctccgacag cgcggagcgg gccgcgtcgg cggaaccggc
45061 cgcggccgcc tcggtacggc gtacgggggc ggggaccagg tccctcagca gcgggtgcgg
45121 ggcccccgtg cgcgcctgtt cgcgcagcac ggtcaggtcc cagcggacgg ggacgagcag
45181 cggctccggg gagcccaggg cgcggtcgag gagtccgagt ccttcctccg tctccagcgc
45241 gacgagcccg gaacggccga tgcgccggtg gtcggcctcg gccaggccgg cggtcatcgt
45301 gcccgtgctc gcccacaggc cccaggccag cgagaggccg ggcaggcccg cggcgcggcg
45361 ggtgtgggcc agcgcgtcga gggcggcgtt cgcggcggcg tagttgccct ggcccgcgtt
45421 gccgaagacc ccggcggccg aggagaacag cacgaaggcc gacaggtcca ggccgcgggt
45481 cagctcgtcc aggtgcaggg cgcccgccac cttcgggtcg aacaccctcc gcacccgctc
45541 gggggtcagg gacggcacca cgccgtcgtc ggtcacgccc gccgcgtgga ccacgcccgt
45601 cagcgggtgt gcgtccggca gcgagtccag cagccggacc agcgcctccc ggtcggccac
45661 gtcgcaggcg acggtctcca ccgcggcgcc cagtgcgccc agctcggccg cgaactcccg
45721 ggcgccgtcg gcggcggggc cgcggcggct cgccagcacc agccggcgga caccgtgccg
45781 ggtcaccagg tgccgggcca ccacccgccc caacgccccg gatgcgccgg tgaccaggac
45841 cgttcccgac ggggcgaaca ccgcggggcc gggggccccg ccgcccgccg gcgcgtcggc
45901 cgcccggacc agccggggcg cgaacagcgc cttcccgcgt acggccacct gcggctcgcc
45961 cgtcgcggcc gcggcggcga gcgccgccgg gtcctcctcg cccgggtcct cgtcgaggtc
46021 gaggaggacg aggcggccgg ggttctccga ctgcgccgca cgcacgaggc cccagaccgc
46081 ggcctgcgcc gggtcggcgg ccgaggacgg atcgacggcc acggcgcccc gggtgaggac
46141 caccaggcgc gacgcggccc accgctcgtc cgcgagccag tcctgtacga gggccagcgc
46201 ggcctgcgcg gccacggccg ggcgggccgc tgcgtcggcg cccgtggggt ccggcagccg

46261 gaccgccacc acgtccggga cgggagtgcc ctcggcggcc ggcggccggg gaagggcgtc
46321 gaggacggcg acgggtaact ccccggccgg ggcggtcagg aggtccccgg ccggcttcca
46381 ctccagccgg aacagcccgc cggcgccgct tccgccggcc gcgagttgct ccgccgccac
46441 cgggcgcagg tccagggagt cgaccgagag caccgcgctg cccgtggcgt ccgcgaggga
46501 cagcgagacc gctccctccc cggccgccgt ggcggacgaa ggcgcgggcg cgagacgcag
46561 gcgcagcgcg gtggcgccgg tcgcgtggag ggtcaccccg ctccacgcga acgggatccg
46621 gggcccgtcg gccggggccg ccggatcacc gaggccgacc gtgtggaggg cggcgtcgaa
46681 cagcgcgggg tgcaggccga atccggtgac gtcggttccg tcgggcaggg cgatctcggc
46741 gaagagttcc tccccgcgcc gccacccggc ccggactcca cggaagaccg ggccgtactc
46801 caggcccagc ccggccatcc ggtcgtacag ggcgccggcg tcgagcggct cggcccccggc
46861 cgggggccac tcggcgagcc cgggggccgc gccggtgccg gccgacagga ggcccgaggc
46921 gtggcaggtc caggcgtcgg cctccatggc gtcgtcgtgc cgtgcgtaca cgcgcacggc
46981 ccgcgcgccg ttcgtgtcgg ggccgtcgac gaccacctgg agctgtgcgc cgccctgctc
47041 gggcaggagg aggggggctt ccagggtgag ttcctcgatc cggccgtacc ccacctggtc
47101 cccggcccgg agcgccagct cgacgagcgc ggttccgggc agcagtgccg cacccagcac
47161 cacgtggtcg gcgagccagg ggtgcgtggc gagcgacacc ctgccggtca gcagcacgcc
47221 ctcgccgtcg gccagggtga cgacggcacc gagcagggcg tgatcggccg gccggagacc
47281 ggtccgggtg aggtcacccg cggcgggccg cgtctccagc ctgtagtgct cgcgctggaa
47341 ggcgtacgtg ggcaggtcga cgcgtcgggc gccgcgtccg gcgaggacgg cggtccagtc
47401 gacgggggatg ccgtggacgt ggagccgggc cagggcggtg gtgacggcca cctcctcggc
47461 gtccctgtcg gcacgcagga cggggatgac ggcggcatcg gcgtcttcga ggaccgtctg
47521 ggccatggcg gtcaggacac cgcccggacc gatctccagg aaggtgctca ccccttcccc
47581 ggcgagggcg gtgaccgcgt cggcgaaacg gacggcctcg cggacgtggc gcacccagta
47641 gtcgacggag cagaactcct cccccgacac cggagcgccg gtgagcgtcg agacgaccgg
47701 aacggtcggg gctttgaagg aaacaccctc ggctaccttg cggaagccgt cgagcatcgg
47761 gtccatcagc ggggagtgga aggcgtgcga gacggagagc ctcttcgtcc tgcggccgtc
47821 cgccgcgagc cggtcgacga ccgccgccac cgcatccgaa gcgcccgaaa ccaccaccgc
47881 cgagggcccg ttgaccgccg cgatccccgc gcggtcctcg aacccggcca gcaggtcccg
47941 tacctcctcc tccgaggcct ccacggcgac catcacgccc gcgggcggca gctcctgcat
48001 cagccggccg cgcgccgcca ccagggccgc cgcgtcttcg agcgagaaca cgcccgccac
48061 atgggcggcc gcgaactcgc ccaccgagtg acccgcgagc aggtccggcc ggacgcccca
48121 ggactccacc agccggaaca gcgccacctc gaccgcgaac agggcgggct gcgtgtaacc
48181 ggtctggttc agcagctcgg catcatcccc gaacaccacg tccttgaccg gccgttcgag
48241 gtgcggtgcc agcgcggcgc agacgtcgtc gaaggcctcc gcgaacaccg tgaaggccgc
48301 gtacagttca cgccccatgc ccggccgctg gctcccctgt cccgagaaca ggaaggccac
48361 cgtctgccgg ggtgcgacga cccccgtcac caggccgccg ctcccggcac cggccgtggg
48421 ggcgtcctgc gcgagccgtt ccagtgcgcg gcgcaggtcc tcacggtccg atgcgacgat
48481 cgccgcgcgg tggtccaggg ccgcccgggt cgtcgccgtc gacaggccca ggtcggccag
48541 ggactggtcg gggtgctcct ccgtgtggcg caacagccgt gcggcctggc cgcgcagggc
48601 ggcctcgttc ttcgcggaga gcagtacggg cgtcgcgcgt gccgggtcct gcgccggggc
48661 gctctctccg tcggccggcc cgggctccgg ccgcgggggc tgctcgatga cgacgtgggc
48721 gttggtgccg ctgaagccga acgaggagac cgccgcgcgc cggggccggt cgccgtgttc
48781 cgcccactcc acgggctcgg tcaggaggcg gacggtcgtg gacgaccagt cgacgtgcgg
48841 ggtcggcgcg tccacgtgga gggtcctggg caggacgccg tgccgcatcg cctcgaccat
48901 cttgatgatc ccgcccaccc cggccgccgc ctgggtgtgc ccgatgttcg acttgagcga
48961 ccccagccac aaaggccgct cggccgaacg cccccgcccg tacgtcgcca gcaacgcctg
49021 cgcctcgatc ggatcaccca gccgcgtccc cgtgccgtgt gcctccaccg cgtcgacttc
49081 ggtggcggac aggccggcgt ccgtgagtgc ccggcggatc acgcgttgct gcgaggggcc
49141 gttcggggcg gtgaggccgt tcgaggcgcc gtcctggttc acggcgctgc cgcggaccac
49201 cgcgagcacc gggtgcccgt tgcggcgggc gtcggacagc cgctccacca gcagcatgcc
49261 cagcccttcg ctccagccgg tgccgtcggc ggcggccgcg aagggcttgc agcggccgtc
49321 ggcggccagt ccgcgctggc ggctgaagtc gacgaaggag gtcggggtga gcatggcggt
49381 gacgccgccc gcgagcgcca ggtcgcattc gctccgccgg agcgcctgca cggcgaggtg
49441 cagggcgacg agggaggagg agcaggcggt gtcgacggtc acggccggcc cttccaggcc
49501 gaaggtgtac gacacccgtc cggagacgac gctcgccgcg ctgcccgtgc cgatgtagcc
49561 gtcgacgtcc tccacggcct gctggagcac cgatgtgtgg tcgtggtaca tcacgcccgt
49621 gaacacgccg gtgcggctgc ccttgaggga ggtggggtcg attcccgccc gctcgaacgt
49681 ctcccagcag gattccagca ggatgcgctg ctgggggtcc atggtgagtg cctcgcgcgg
49741 ggatatctcg aagaggcccg cgtcgaagag gtcgatcccg tcgaggaatc cgccttcccg
49801 gacgtggctg gtgccgcggg tgtccgggtc ggggtcgtag agggattcga ggtcccagcc
49861 gcggtcggcg gggaattccg tgatggcgtc gccgccgtcc gtcaggaact gccacaggcc
49921 ctcgggtgag gtgatcccgc cggggaagcg gcagctcatc gcgacgatgg cgatcggctc
49981 gtgctgtgcc gcgtcgaggt cgcgcagccg ctcgcgggcc tcgtggaggt ccgccgatac

50041 ccgcttgagg aagtagcgca gcttttcctc gttgttgttc gcctggaggt cagacacctg
50101 gttcaccttt tacgtcgagt tgcgtcgagc cggtggggcc ggaagtcggc agatgggctg
50161 tgcgtggcgg gtggccgcca actccgcggt tcaggagatt ccgaattcct tgccgatgaa
50221 gtcgaacatc tcgtcgtccg tggcggcgtg gagtccgtcc gccacgtccg cgggaccggt
50281 ggcgccggag gtccccgacg cctccggcgc cgtgcctccg tcggccgcgc cggtggtcgt
50341 ggcccactgg tcgagcaggt cccgcaggcg cgaggcgatg gcctcgccgc cttccccgcc
50401 ggccgccgcc gtgttccgca gcgccgtttc gatccggtcg agttcggcga gcatcccggc
50461 gacgcccggg gcgggcgggg ccgcctcggc ctggaggtac tcgaccagtt cggccggggt
50521 cgggtggtcg aagacgagcg tgggggcag ccgcagcccg gtgtccttgc ccagccggtt
50581 gcggaactcg accgcggtca gggagtcgaa cccgaggtcg cgcagggcct gaccgggccg
50641 gaccgcccgg ggcgaggcga ggtcgaggac cgcggcgacg tgctcgcaga ccaggtcgag
50701 cagcagcttg cgctgttcgg cctcggcgag accggcgagg cgcgccgaca gctcccgccg
50761 ctcggtcccc tccgccccgg cccccgtagc ggcgcccgag gctgcgtacc cggcctcgtc
50821 cgcggccggc cggcgggccg ggacccggac gagcgaggtg agcagggccg gcagtgagcc
50881 ggccgcggcg cggccgcgga gtgccgcctg gttcagccgt acgggcacca ccgtgccctg
50941 cggccgggcc gtcgcggcgt cgaagaggcc gagggcctcg tcggtggcca ggggcagcac
51001 cccgctgcgg gccatgcggc cgcggtccgc gtcgctgagc gcgccggtga tcccgctgct
51061 ctcggcccag agcccccagg ccagcgatac ggcgggcagt ccggccgccc ggcgctgctg
51121 ggcgagggcg tccaggaagg agttcgcggc ggcgtagttg ccctggcccg ccgcgccgaa
51181 gaccccggcg gcggaggaga agaggacgaa cgcggacagt tccatgtcgc gggtgagttc
51241 gtccaggtgc agggccgcgt cgactttcgg ccggaacacc cggtcgatcc gttccgcggt
51301 cagaccggtg acgatgccgt cgtcggtcac tccggccacg tgcaccaccc cggtcagggg
51361 gtgttcggcg ggtacctccg ccaacagccg ggccagcgcg tcgcggtcgg ccacgtcgca
51421 ggcggcgacg gtgacccggg ccccccgttc ggcgagttcc gcctccagtt cggccatgcc
51481 cgccgcgagg gcgccgctcc ggctgacgag cagcaggtcg cgcacgccgt gcccggtgac
51541 caggtgccgt gcgacgtggc gccccagcag gcccgaggcg ccggtgacca gcaccgtacc
51601 gccggccgtg aacgcggggc tcgggtcgtc gagggaggcc ggggtctcgg cccgcgccag
51661 ccgcggtacg agcaccgatc cgccccgtac ggcgaactgt tcctcgcccg aggccagcag
51721 cgcgggcagc gcccgtacgg aggcggcgtc tcggtccagg tcgaccagga caaaccggcc
51781 ggggttctcc gactgcgccg aacgcaccag gccccagatc gcggcctgcg ccgggtcgac
51841 ctccttgtcg tggtcctctt ggtccctgtc cttggcgacg acggcgttgg cggtcaggac
51901 gaccagccgg gtgtcggcgt tccgctcgtc ggagagccag tgctggagca gttccagcgt
51961 ccggtgcgcg ctctcgtggg cgtgtgccac cgggtggtcg ccgtccgggt gtacgtcgac
52021 ggccgcgagc acgactcccc ccagcgccgc cgcaccgtcc ggacccggcc ggtacgagac
52081 gctcgcgccg gagtcctcca gggcggcgcg cagaccgtcg tctccgccga tgagggtcca
52141 ctgcccggcc ggttcgctca cagaagcctc cgaggtgagg ggaagggcgg tccagtcgac
52201 gcggtagagg gagtcgtacg gtccgtgctc gcggcccgcc cggtcgagct gctccgcgga
52261 gacggggcgc agcaccaggg agtcgaccga ggcgacgggc cggcccgccg tgtcggtcgc
52321 cagcagggcc acggcgtccg ctccggccgg ggtgatcctg acccgcaggg cggtggcgcc
52381 ggtggcgtgt accgtcaccc cgctccaggc gaaggggatc cgtccgtgtc cggcgtcggg
52441 cagcaggcca ccgaggccga tcgcgtggag cgcggagtcg aggagcgccg ggtggatccc
52501 gaagccgccc gtttccgtgc cgtcggggag ccgtacctcg gcgaacacct cgtcgccgcg
52561 ccgccacagg gcgtgcaggc cctggaacac cggtccgtag ccgaagccga ggccggccag
52621 ccgctcgtag gctccgccgg tgtccaggcg gtcggccccg gggcgggcc agaccgcgag
52681 gtcgtgcggc tgccctcccc cggcgtcccg gcgcgggtcc acggacacga caccggtggc
52741 gtggcaggtc cacggctcgg cgacgtcggc gctctcggga cgcgagtaga cgttgacggt
52801 acggtgcccg gtcgcgtccg ggccgccgac gacggcctgg aggcgtaagg ccgctcgctc
52861 gcgcaggacg agcggggcgc cgagggtcag ctcgtccaca cggtcgcagc ccacctcctc
52921 ggcggcccgc agggcgaggt cgaccagggc ggtgcccggc agcaggaccg tgcccatgac
52981 cgtgtggtcg gtgagccacg ggtgcgtagc gggcgacagc cgtccggtga gcagggcgcc
53041 ctcaccgtcc gcgagggcga cggcggcacc cagcaggggg tgctcggccg ccacgagccc
53101 ggcggaggcg acgtctccgg ccggggaacc cggggcgagc cagtagcggc ggcggtggaa
53161 ggcgtaggtg ggcaggtcga cctgccgcgg cgtgccgtgt ccggcgaaga cggccgacca
53221 gtcgaccgat gtcccgtggg cgtggaggtg ggcgagggcg gtggtgacgg ccaggtcctc
53281 ggcgcggtcg gtccgcagga gggggacggc gaccgcgtgc tcgtccaggg agtcctgggc
53341 catggcggtc aggaccccgc ccgggcccat ctccaggaag gtgcccgcac cctcgtcggc
53401 gagggcggtg acggcgtcgg cgaagcggac ggcctcgcgg acgtggcgca cccagtggtc
53461 gacggagcag aactcctcgg ccgacaccag ggcgccggtg agggtggaga cgaccggcag
53521 ctgcggggcg gcgaaggtga ccgtctccac caccttgcgg aactcgtcga gcatcgggtc
53581 catcaggggc gagtggaacg cgtgggagac ggacagcgcc ttcgtcttgc ggccgtcggc
53641 cgagagacgg tcgaccagtg cggccaccgc ttcctcggcg cccgaaacca ccacagaaga
53701 gggcccgttg acggcggcga cgcccgcgcg gtcctcgtac ccggacagca gttcccgtac
53761 ttcttcctcc gaggcctgca ccgcgaccat cacgcccccg gacgggagcg cctgcatcag

53821 ccggccacgg gccgcgacca gggccgccgc gtcctccagc gagaacacac ccgccacatg
53881 ggcagccgcg aactcgccca ccgaatgacc ggccaggaag tccggccgga caccccacga
53941 ctccaccagc cggaacagcg ccacctcgac ggcgaacaag gcgggctgcg cgaaaccggt
54001 ctggttcagc agcccggcgt cgcccccgaa caccacgtcc ttcagcggcc gttcgaggtg
54061 cgcgtccagc gcggcgcaca cggcgtcgaa agccccggcg aacaccggga aggccgcgta
54121 cagttcacgc cccatccccg gccgctggct cccctgcccc gagaacagga acgcggtccg
54181 gctgccgccg ggctccggtt cgccgtgtac gaccccggct gccggtcggc ctgcggcaag
54241 ggccgtcagg ccctccagca gctcctcgcg ccgggtgccg acgaccgccg cgcgcacctc
54301 catcgccgca cgcgaggtgg cgagcgaccg gccgacctcg gcgacgccca gggccgggtg
54361 cgcgtcgaga tgggcggcga tgcgctgtgc ctgggcgcgc aaggacacct cgtccttggc
54421 cgagagcacc cacgggacgg cgaccggcgc cggtgcgtcc tcgaccgccg tggcctgctc
54481 cgtgcccggt acgtgttcca gtacgacgtg cgcgttggtg ccgctgacgc cgaaggagga
54541 gacggcggac cgccgggggc gtccggactg cgcccattcc cgcgcctcgg tgagcagctc
54601 gaccgctccg gccgaccagt cgatgtgggg tgagggctcg tcggcgtgga gcgtcctggg
54661 caggacgccg tgccgcatcg cctcgaccat cttgatgact cccgcgacgc ctgcggcggc
54721 ctgggtgtgc ccgatgttgg acttgaggga gcccagccac agcggccggc cctccggccg
54781 gtcctggccg tacgtggcca gcagcgcctg cgcctcgatc gggtcgccga gggcggttcc
54841 cgtaccgtgc gcctccacgg cgtccacctc ggaagcggac aggccggcct gggcgagggc
54901 ctggcggatc acccgctgct gcgcgggacc gttgggagcc gtgaggccgt tcgaggcgcc
54961 gtcctggttc accgcgctgc ccggaccac cgccagtacc ggtgtccgt tgcgtcgggc
55021 gtcggagagg cgctccacca ggagcatgcc cacgccctcg ctccagccgg tgccgtcggc
55081 cgcggccgag aaggccttgc agcggccgtc ggcggccagc ccgcgctggc ggctgaactc
55141 gacgaaggtg ttcggggtgg ccatgacggt cacaccgccg accagggcca tcgagcactc
55201 gccctgccgc agcgactgcg ccgccaggtg cagggcgacc agggacgagg agcaggcggt
55261 gtccaccgtc accgccggcc cctccaggcc gaaggtgtag gagagccggc ccgaggcgac
55321 gctgccggag gtgcccatgc cgaggtgtcc ttcgagccct tcgggtacgg ccccgccgga
55381 ctgtgatccg tagtcgtggt acatgacgcc cgcgaagact ccggtgcgcg aaccgcgcag
55441 cgaggcggga tcgatcccgg cccgctcgaa cacctcccac gaggtctcca gcagcaaccg
55501 ctgctgcgga tccatggcaa gggcctcacg cggcgagatc ccgaagaagc cggcatcgaa
55561 ctccgccgcg tcgtaaagga acccaccctc ccgcgcataa ctcgtcccca cgtgatccgg
55621 atccgggtga tacagcgaat ccagcgccca cccacggtcc accgggaaac cacccaccgc
55681 gtcaccccccc tccgcgacca gccgccacag atcctccgga gacctcaccc cacccggata
55741 acggcagctc atccccacga tcgcgatcgg ctcgtcgacg gagacgaccg cggccggggc
55801 gtgttccggg gttccggacc gtgtgcccac gagttcggtg agcaggtggc gggtgagtgc
55861 ctcgggtgtg gggtggtcga agacgagggt ggcgggcagc cgaaggccgg tggccgtgtt
55921 gagggagttg cggagttcga cggcgttgag cgagtcgaag ccgaggtccc tgaactgccg
55981 gtccaccgcg accgaccgga cggattcgtg gccgagcacc gtggccgcct gggtccggac
56041 gagggcgagg agggcgtcgt accggtcggc gggcggcagg gccgcgatcc ggtcggccgg
56101 cgaggacccc gtggcgttct gcggcgcgcc gtgaagcgcg cgccgtacgg agccccgtac
56161 cagttgccgg aacagcgggg gcagggcgcc tgcgcgggcg tgtgcccgga gcgcggtggt
56221 gtccaggcgg accggaacca ccgtggcgag ggcggagcgg caggcggcgt cgaagagcgc
56281 caggccctcc tgggcctcca gcgcgaccag ccccgaccgg ctgatcctgc gccggtccgc
56341 ggcggccagg tcggccgtga gcgcgctggt ctccgcccac agcccccagg cgatggacac
56401 cgcgggcagg ccggcggccc gccgggtttc ggcgagggcg tcgaggtagg cgttcgcagc
56461 ggcgtagttg gcctgtcccg cgctgccgag ggtgccggcg gcggaggaga agaggacgaa
56521 ggcggtgagg tcggcgtcgc gggtgagttc gtgcaggttg agggcggcgt cggccttggg
56581 gcgaaagacg tggtcgacgc gctcgggggt gagtgcggaa acgattccgt cgtcggtgac
56641 tcccgccgcg tggaccactc cggtcagcgg ccggtccgcc ggtaccgccg cgagcagccc
56701 ggccagcgcc gtacggtccg ccacgtcgca gcgtacggcc cgtacctcgg cgcccagggc
56761 ccccagctcc tcggcgaact ccggcgcgcc ctcggcgtcc gggccccgcc ggctcgccag
56821 cagcaggtgc cgcaccccgt gctcggtcac caagtgccgt gcgacgaggc gccccagcac
56881 accggaggcg ccggtcacca ggaccgtccc cccggccgag aaggcgggac gcaggcccgt
56941 ggtgtcggcg tcggcgtcgg cggggcgttc ggtggacgtg gcgtcgatcc gtgtcagccg
57001 cggggcgagc agcgcgccgt cccggaccgc cacctgcggc tcgcccgagg ccaccgcggc
57061 cgcgaggggg gcctcggcga gggtctcggc gccgtccgcg aggtccacca gcacgaaccg
57121 gccggggttc tccgactgcg cggagcgcac cagtccccat accgaagcct gtgccgggtc
57181 cacgccggcg acgtcggcgg cggtgccggt gttcaccgca cctcgggtca cgatgaccag
57241 gcgggactcg gcgaagcgcg gctccgccag ccactcctgg atcagtgcca gcacgcgctg
57301 cgcggtcgcg gcggccgagg tgtccgcact gccgtgggcg ttccccggca ccgtgacggc
57361 cacggtgtgc ggggccggag cctgagtcgc ctgagcgtca ggcaggccct cgcacaccag
57421 cacgcccacg tgctccgctc ccggggccgg atcgcacggc tcccagtcca cccggaagag
57481 ggagtcccgg agcgtggaag ccgcctgccc gccgccttct ccggcgagtt gcccgggtgt
57541 caccgcccgc agcgtcagcg agtccaccga tgccacgggc cggcccaggc cgtcggtgat

57601 cgccagcgac accgagtccg ccgcggagtc ggcccgggtc agacggaccc gcagcgtctc
57661 ggcgccggtt ccgtggacgc tcacaccgct ccaggcgaac ggcagcagtc cctcgtgccc
57721 gatcgccggg acgtcgccca cgaccatggc gtggagcgcc gcgtcgagga gggcggggtg
57781 cacggcgaac ccgtcgacgg aggtgccgtc gggcagcccg acctcggcga agagctcgtc
57841 ctctccccgc cgccacaccg cacgcaggcc ctggaacgcg ggcccgtacg cgaagccggc
57901 gtcggccagc cgcccgtagc agtcgtggac gtcgacgggg gtggagccgg ccggcggcca
57961 ggcctcggcc gtcccggacg ggcccgggac gtcgggcgcg tgcgccgcgc cggtgtcggg
58021 gacgaggaag ccggtggcgt gacgggtcca gggcagctcc gtggcggcct cgtcgttggc
58081 atcggtgcgc gagtagatcc gcaccgtacg ccggtcggct tcgtcggcgg ctccgaccac
58141 cacccgtatc tggaccgcac cgtgttccgg gagcaccagg ggcgcggcga gggtgagttc
58201 gtcgacgcga ccgcagtcca gctcgtctcc ggcccgaacg gccatctcca ggaaggcggt
58261 tcccggcagc agggtcaggc cggagatcgc gtggtccgcc agccaggggt gggtggccag
58321 cgacagccgg ccggtcagca gcgacccagc accgtccggc agcagggccg ccgcgccgaa
58381 cagggcgtgt ccggcagcgg ccaggccggc cgcggcgaca tcgccggggg cggtcggcac
58441 gtcgagccag tagtgctcgt actgaaaggc gtacgtgggc aggtcgacgc gtcgggcgcc
58501 ggatccggtg aagaacgtcg tccagtcgac gggtaccccg tggacgtgga ggtgggcgag
58561 ggcggtggtg acggccaggt cctcggcgcg gtcggtgcgc aggagcggaa cggtgacggc
58621 gtgctcgtcg agggagtcct gggccatggc ggtcaggacc ccgcccggac cgacctccag
58681 gaaggtacgg acgcccaggt gggcgaggga ggtgacggcg tcggcgaagc ggacggcctc
58741 gcggacgtgg cgcacccagt agtcggccga ggagagctct tccacagtgg ccggagcacc
58801 ggtgagggtc gagacgagcg gcaggcgcgg cgctccgaag gtgactccgc ccacgacggc
58861 ccggaagtcg tcgagcatcg ggtccatcag gggtgagtgg aaggcgtggg agacggtcaa
58921 cgccttcgtc ttgcggccgt cggccgcgag ccggtcgacg accgccgcca cggcgtcctc
58981 tgcgccggaa accaccacgg cggaggggcc gttgaccgcg gcgataccgg cacggtcctc
59041 gaacccggac agcaggtccc gtacctcggc ctccgaggcc tgcaccgcca ccatcacccc
59101 gccagagggg agcgcctcca tcagacgccc gcgcgccgcc accagagccg cggcgtcttc
59161 gagcgagagc acgcccgcca cgtgggccgc cgcgaactcg cccaccgaat ggcccgccag
59221 gaagtccggc cggacgcccc acgactccac gagccggaac agggccactt cgagggcgaa
59281 cagggccggc tgggcgtggg cggtccggtg cagcagttcg gggttctcac cgaacaccac
59341 gtccctgacc ggccgttcga ggtgtgcgtc caaggcggcg caggccgcgt cgaaggcctc
59401 ggcgaagacc gggaaggccg cgtacagttc acgtcccgct ccgaggcgct ggctcccctg
59461 ccccgagaac aggaacgcgg tccggccgcc gcccgcgggc gtgccggtcg aggtctcatc
59521 ccgtgcgagc gcgccgggcg ccgggcggcc ctcggcgagg gcgcccaggg cggccatcag
59581 ctcctcgcgg ccggtgccga cggccgcgga ccgcaggtcc agcgcggccc tggaggccgc
59641 cagggacagg ccgatgtcgg ccgtgccgag gtccgggtgt tccatcaggt gcgtcagcag
59701 gcgtgcggcc tgctcgcgca gggccgccgg gtccttggcg gacagcggcc acgcgacggc
59761 tgtggcggtg gccgtacccg cggtcggcgt gttcgtcggc ccgtcggccc gcggctgccg
59821 ctccgactgc tccggctgct ccggctgttc gatgaccacg tgcgcgttgg tgccgctgac
59881 gccgaaggag gacacgcccg cccggcgcgg acgcccggac tgcgcccatt cccgcgcctc
59941 ggtgagcagc tcgaccgctc cggccgacca gtcgatgtgg ggtgagggct cgtcggcgtg
60001 cagggtcctg ggcaggacgc cgtgccgcat cgcctcgacc atcttgatga cgcccgcgac
60061 gcctgcggcg gcctgggtgt ggccgatgtt ggacttcagg gagcccagcc acagcggccg
60121 gccctccggc cggtcctggc cgtacgtggc cagcagcgcc tgcgcctcga tcgggtcccc
60181 gagccgggtg ccggtgccgt gcgcctccac cacgtcgacg tcggtggccg acaggcccgc
60241 gttggcgagc gcctgccgga tgacccgctg ctgcgcggga ccgttcgggg cggtgaggcc
60301 gttggacgcg ccgtcctggt tgatcgccga accccgtacg acggccagca cccggtgccc
60361 gttgcggcgg gcgtccgaga ggcgttcgag gaggagcatg ccggtgccct cgccccagcc
60421 ggtgccgtcg gccgcggccg agaaggcctt gcagcggccg tcggcggcca gcccgcgctg
60481 gcggctgaac tcgacgaagg tgttcggggt ggccatgacg gtcacaccgc cgaccagggc
60541 catcgagcac tcgccctgcc gcagcgactg cgccgcgagg tgcagggcga ccagggacga
60601 ggagcaggcg gtgtccaccg tcaccgccgg cccctccaga ccgaaggtgt aagccacccg
60661 gcccgaggcg atgctgcccg cgctcccgtt gaccaggtat ccctcgaacc cgtcgggtac
60721 ggcggacagg cgcgagccgt agtcgtggta catgacgccc gcgaagactc cggtgcgcga
60781 accgcgcagc gaggcgggat cgatcccggc ccgctcgaac acctcccacg aggtctccag
60841 cagcaaccgc tgctgcggat ccatggcaag ggcctcacgc ggcgagatcc cgaagaagcc
60901 ggcatcgaac tccgccgcgt cgtacaggaa cccaccctcc cgcgcataac tcgtccccac
60961 gtgatccgga tccgggtgat acagcgaatc cagcgcccac ccacggtcca ccgggaaacc
61021 acccaccgcg tcaccccccct ccgcgaccaa ccgccacaga tcctccggag acctcacccc
61081 acccggataa cggcagctca tccccacgat cgcgatcggt tcgtcgacgg agacggacgg
61141 gacaccgccc ggcgggccgg cgtcggcctc gccgccgagg atctcgccgc gcaggaagcg
61201 ggtgagcgcc agcggtgtgg ggtggtcgaa gaccagggtg gcgggcagtc gggtgccggt
61261 gacggagccc aggaggttgc gcagttcgac cgcggtgagc gagtcgaagc cgaggtccct
61321 gaactggcgc tcgggttcga tccgctccgg cgaggcgtgg ccgagcactg tggcgatctg

61381 gaggcgtacc aggtcaccga ggacgcgttc ctgctccgcc gcgggaagcc cgtgcaggct
61441 cgcggccagg gcggtggcgc ggtcggccgc ccccgtggcc gtcgcgtcgc ccgcggcccg
61501 ggccttgcgg accggggcct tgacgagtcc gcgcagcagt gcgggcacct tgtcgccggc
61561 agcctggacc cgcagtcccg cgtggtccag cgggaccggg aagagaacgt cgtccccggc
61621 ggcgcacgcg gcgtcgaaca gggcgaggcc cgtggccgag tccatggcgg ccaggcccat
61681 gcggcggatg cgcgccaggt cggccgagtc cagcttgccg gtcatcccgc tgccctcggc
61741 ccacagcccc caggccatgg acacggcggg cagaccggcc gcgcggcgca gctgggccag
61801 ggcgtcgagg aaggagttcg cggcggcgta gttggcctgt ccggcgccgc cgagggtcgc
61861 ggacaccgag gagaagagga tgaacgcgga caggtccgcg tcccgggtga gttcgtgcag
61921 gttcagtgcg gcgtccacct tcgggcggag cacgtggtcg acgcgttccg ggtgagcgc
61981 ggggacgatg ccgtcgtcgg tgacgcccgc cgcgtgcacc accgccgtca gcgggtgcgc
62041 agcgggggatc gtggcgagca gtgcggcgag ggcctcgcgg tcggcgacgt cgcaggcggc
62101 ggtgacgacg cgggcgccga gggcggtcag ttcggcctcg aagccggccg ctccttcggc
62161 gccggggccc cggcgtccgg tgagcacgag gtgccggacg ccgtgccggg tgacgaggtg
62221 ccgggcgacg aggcggccca gcgtgccggt cgcccccgtg acgaggacgg tccctccgtc
62281 cgggaaggtc cggccggggc cggaccccgc gccgccctcc tccgcggcgc tgctcgtccg
62341 cgtgaggcgg ggcaccagaa tcgcttcagc gcgcaccgcg agctgcggtt cgcccgtgct
62401 cacggcggcc cgtacggccg cgcggccgag gtcgccgtcc ggatccaggt ccgggtccag
62461 gtcgaggagg acgaagcgtc ccgggttctc cgactgcgcc gagcggacca tgccccagac
62521 ggcggcctgg gccgggtcga gcgccccgcg ttccgccgcg tcggtcgcga cggcgtgccg
62581 ggtgaccagg acgagcggcg ccgggtcggc ggcgtcgtcg gcgatccgtt gctggatcag
62641 gcgcagtgcc cggtggctcg tggtgcgcac gcgggctgcc gtgtcgtccg ccgggtcgtc
62701 ggctccggtg tccggacggc cgggcgtcgc cacgtcgaac tcggggacgg gtccgtccgc
62761 cggcgcgtgg gccggggtcc agtcgagccg gtagagggag tcgtggcggg gctccgcggc
62821 cagccgcagc cggtcggcgg agaccgggcg caggacgagt gcgccgatcg tcgcgagcgg
62881 gcggcccgta ccgtcggcga cgaggaccga caccgcgtcc gtcccggcgg cggccagctt
62941 gatccgtacc gcggtcgcgc cggcggcgtg gaggtgcacg gcgctccagg agaacggcag
63001 cagaccgttg ccggtctcgg agccggacag caggccgagc cacgaggcgt gcagcccggc
63061 gtcgaggagg gccgggtgca gtccgtacgc gcccgcgtcc ggcgccgact cctcggggag
63121 ggcggcggcc gcgtacaggg tgtcgccgtg gcgccaggcc tccgtgaggc cgcggaagac
63181 cgggccgtag ccgaagccga ggtcgtcgag gtggtcgtac aggtcgtcgg tggcgacccg
63241 ttcggcaccc tgcggcggcc aggcgacgag ttcggaggat gcgggcgccg gcggcccggc
63301 ggagagggtg ccggccgcgt gtcgggtcca gtcccgtgtc ccgggggcgt cctccggccg
63361 ggagtacagc tccagcgcgc agcgtccgga ggcgtcccgg gtcccgaccg tgaggcggag
63421 ctggaccccg ccgtgctcgg gcaggacgag cggggcctcc agggtcagct cctcgatctg
63481 ctcgcagccg gcttcccggc cggcccgcag cgcgagctcc acgaaggccg tgccggggac
63541 gagcacgacg ccggagaccg cgtggtccgc gatccaggga tgcgtggcga gcgacagccg
63601 gcccgtcagc acgatcgcgc cgtccgggag caggacggtg gctcccagca cggggtgttc
63661 ggtgtcggcg accccgatcg cggtgacgtc ggcggtggcg gggacgtgga gccagtagtg
63721 ctcgcgctga aaggcgtacg tgggcaggtc gacgcgtcgg gcgccggatc cggtgaagaa
63781 cgtcgtccag tcgacgggta ccccgtggac gtggaggtgg gcgagggcgg tggtgacggc
63841 cgttccctcg gcacggtcgg cgcgcaggag ggggacggcg acggcgtgcc cgtcgaggga
63901 gtcctgggcc atggcggtca ggaccccgcc cggaccgacc tccaggaagg tacggacgcc
63961 caggtcggcg agggaggtga cggcatcggc gaagcggacg gcctcgcgga cgtggcgcac
64021 ccagtagtcg gccgaggaga gctcttccgc agtggccgga gcaccggtga gggtcgagac
64081 gagcggcagg cgcggcgctc cgaaggtgac tccgcccacg acggcccgga agtcgtcgag
64141 catcgggtcc atcaggggtg agtggaaggc gtgggagacg gtcaacgcct tcgtcttgcg
64201 gccgtcggcc gcgagccggt cgacgaccgc cgccacggcg tcctgcgcac cggaaaccac
64261 cacggcggag gggccgttga ccgcggcgat acccgcacgg tcctcgaacc cggacagcag
64321 gtcccgtacc tcggcctccg aggcctgcac cgccaccatc accccgccag aggggagcgc
64381 ctgcatcagc cggccgcgcg ccgccaccag ggccgccgcg tcctccagtg agaacacccc
64441 ggccacatgg gcggccgcga actcgcccac cgagtgaccc gccaggaagt ccggctggac
64501 accccaggac tccaccagcc ggaacaatgc cacctcgacc gcgaacagcg cggctgcgc
64561 gtacgccgtc cggtccagca gggcggcgtc ctcaccgaac accacacccg ccagcggacg
64621 ttcgaggtac tggtccacat gcgcgcacac cgcgtcgaag gcgtccgcga acaccgggaa
64681 ggccgcatac agctcacgcc ccatccccag ccgctggctc ccctgccccg agaacaggaa
64741 cgccgtacgg ctgccctcgt cgtcgcctcc ggagtcggtg gcgccggtga tgacgtggga
64801 cgaggacccg gcggggcctg cgtcggcgag ggctgccagt ccgcggagca gctcgtcgcg
64861 gtcggtgccc acgacggcgg cgcgccgctg gagggccgac cggcccgtgg cgagcgagag
64921 gccgatgtcg gcgggggcgg cctccgggtg gtcggccagg tgggagcgca gccggtcggc
64981 ctgtacgcgc agggcggtgg cgtccttcgc ggagagcagc caggggacgg ccgccaccgg
65041 ggtggccggg gtggccgcgg tggcggtgtc ttcgggctcc gggtccggtt cgggggcttc
65101 ctcgacgatg acgtgggcgt tggtgccgct cacgccgaag gccgagacgc cgccccggcg

65161 gacgtgttcg tgatcgtcgg gccaggtcac gggttcggtc aggagctcga ccgcgcccgt
65221 ggtccagtcg acgtgcgggg tcggcgcgtc cacgtgcagg gtcctgggaa ccgtcccgtg
65281 ctggagggac agcaccagct tcatgacgcc cgcgacgcct gcggcggcct gggtgtggcc
65341 gatgttggac ttcagggagc ccagccacag cggccggccc tccggccggt cctggccgta
65401 cgtggccagc agcgcctgcg cctcgatcgg gtccccgagc cgggtgccgg tgccgtgcgc
65461 ctccaccacg tcgacgtcgg tggccgacag gcccgcgttg gcgagcgcct gccggatgac
65521 ccgctgctgc gcgggaccgt tcggggcggt gaggccgttg gacgcgccgt cctggttgat
65581 cgccgaaccc cgtacgacgg ccagcacccg gtgcccgttg cggcgggcgt ccgagaggcg
65641 ttcgaggagg agcatgccgg tgccctcgcc ccagccggtg ccgtcggcgg ccgcggcgaa
65701 cgccttgcag cggccgtcgg cggccagtcc gcgctggcgg ctgaactcga cgaagatgcc
65761 ggggttggcc atgacggtga ccccgccggc gagggccagt tcgcattcgc ccaggcgcag
65821 cgactggacg gccaggtgga gggcgaccag ggaggaggag caggcggtgt cgacggtcag
65881 cgccgggcct tcgaggccga aggtgtaggc gaggcggccg gagacgacgc tggcggcccg
65941 gccggtgagc aggtagccct ccacgccttc gggggccttg cgcaggccgg aggcgtagtc
66001 ctgtccgttg gagccgatga agactccgga ccgggtgccc ttgagggtgg cggggtcgat
66061 gcccgcgcgt tcgaaggcct cccaggtgac ttcgagcagc agccgctgct gggggtccat
66121 ggcgagggcc tcgcgcgggg agatcccgaa gaacccggcg tcgaagtcgc ccgcgtcgta
66181 gaggaagccg ccttcgcggg cgtaggtggt cccggggttc tgggggtcgg ggtggtagag
66241 ggattcgagc ggccagccgc ggttctcggg gaatccggag aggacgtccc ggccctcgtg
66301 gaggatctcc cacagctctt cgggggaggt gacccctccg gggaagcggc agctcatcgc
66361 gacgatggcg atcggctcgt cgtcggcggg cggggcgagc ggtgtaccgg ggtggccgtc
66421 gtcgtggccg gcggccccg ggcttcccgc tatccggtcg tgcaggtatc cggcgagttc
66481 cgtcgggttg gggtagtcga agacgacggt ggtgggcagg cgcatcccgg tggcgccggc
66541 gagccggttg cgcagctcga tcgcggtgag ggagtcgaac ccggtgtcgc ggaaggcccg
66601 ccggggcggg acggcctcga cgccggagtg tccgaggacg gtggcggcct gggtgcggac
66661 gagttcgacg acggcccgga gccgttcggt gccggacagg ccggtcagcc gctgggccag
66721 taccgggccg cccgcgccgc cggcggcacc ggtggcgggg gtcgcggcgg cgtccaggat
66781 gcgccgggcc tcggggagac ggcgcagcag cgggtagtcg ccgctgatgg cgaaggcggg
66841 gacgaaacgg tcccagtcga tgtcggcgag ggtgacggcc gtgtcgtggt gttccatggc
66901 gcggcgcagg gccgcgatgc cgaggtcggc gggcatgggg aggatgccgc gccgggtgag
66961 gtgctgttcg gcgaggtctc ccatcatgcg gccgctgccc caggagcccc aggcgatggc
67021 ggtggccggg agcccctggg cgcggcgctg gtgggcgagg gcgtccagga aggcgttgcc
67081 ggcggcgtac ccgccctggc cgccgttgcc gacgacgccg gcgatggagg agaagaggac
67141 gaagaagtcc aggtcgaggc cggccgtgag ctcgtgcagg tggagcgcgg cgtcgacctt
67201 ggggagcagg acgtgttcga ggcgctcggt ggtcatggcg tcgaggacgc cgtcgtcgag
67261 gacgcccgcg gcgtggatga ctccggtgag gggtgttcg ccgggcagcg agtccaggag
67321 ccggccgagg tcttcgcggt cggtgacgtc gcaggcggcg atggtggtac gggtgccgag
67381 ggcggcgagt tcgtcgcgga gttcggcggc gccgggggcg gccgggccgc tgcggctggt
67441 gaggacgagg tgggcggctc cggagcgggc gagccagcgg gcgacgtggg cgccgagggt
67501 gccggtgccg ccggtgatca ggacggtgcc gtgcgggcgg gccaggacgt ggtcgggtgt
67561 ggcggcggtg gtgggcgcgg tcgtgtccgt gtccgcgcgg acgaggcggc gggcgagggt
67621 gccggtggcg cgcacggcga tctcttcctc gccgtcgggg tcggcgagga gtgcggtgag
67681 gtgttcgagg gtgcgctcgt ccagggtttc ggggaggtcg acgaggccgc cccagcgctg
67741 gggggactcc gcgccgacga cccggccgag gccccacagc tgggcctgct cggcctgcg
67801 cggcgggtcg gcgggcccgg tggcgacggc gccggaggtg gcgcaccaga gggggcgcc
67861 gatgccggtg tcgccgaggg cctggacgag ggcgagggtg gcggcgaggg cgggcaggac
67921 ccgggggcg ggcggggtgc cgggcacggc gtccccggcc ctccccgtgg cggcgccggt
67981 accggcgagg agggagagca ccccgctgac gccgtcgggc tccagtccgg ccccgccgag
68041 gccgtcggtc aggagccggg cgacggtctc ccggtcgctg tcggcggccg ggtcgaggac
68101 gacccgttcg acgcgggcgc cgcgggcggt gagggcctcg tggacggcgg cggtggcggc
68161 gtgggcgccg ccgtgccggt cgacggggt gacgagcagc caggtgccgc tcagcggggt
68221 gcggttgccg cgccgggcct gggtggcggt gggcgtccag gtgacgcggt agcgccagcc
68281 gtcgatgagc gcgctctcgg tggtgcggcg ccgccagtgc gcgagccggt tgaccatgct
68341 gtcgagggag gcgtcggggg ccaggtccag cgagctggtc agggccctta cgtcggcgtt
68401 ctcgacggct tcccagaagc gggcctcctc ctcgctgtgc ccggcggcgg cgcccgcctg
68461 ggcggtcggg gcgccggcgt cgagccagaa ccggcggcgc tggaaggggt aggtgggcag
68521 ggcgacggta cgggggcgca gtccggcgaa ggcggcggac cagtcggtgg gcacgccgtg
68581 gacgtacgcg tccgcgagcg aggtcaggaa gcgggccggg ccgccgtcgc cgcggcgcag
68641 cgagggcacg acgtcggcgg tgacgccggc cgactcggcg gtctcctgga ggccgacggc
68701 gagcacgggg tgcgggctgc attcgacgaa gacgtcgtgg ccggactcca gcaggcggcg
68761 cacggcgggc tcgaagcgga cggtggtgcg gaggttggtg taccagtact cggcgccgag
68821 gtccgcggag tcgacgacct cgccggtgac ggtggagcag aaggggatcc gcgggggccg
68881 cggggtgatg ccggcgagcg cctcggcgag ttcggcctcg atgatctcga cgtgggcgga

68941 gtgcgaggcg tagtcgacgg ggacccgctt ggcccggacc ccgtcggcgg tgcaggcggc
69001 gaggagttcg tcgagggcgt ccgcgtcgcc ggagacgacg atggagcggg gtccgttgac
69061 ggcggcgatg ccgagccggt cgtcccacgc ctccaggcgc cgggtgacgt cggccagggg
69121 ttcgggtacg gacaccatgc ctccgagtcc ggcgagccgg ctcagcaggc ggctgcggag
69181 ggcgacgacg cgcgcggcgt cgtcgaggcc gagggcaccg gacacggcgg cggcggcgat
69241 ctcgccctgg gagtggccga tgacggccgc gggttcgacg ccgaaggagc gccacagttc
69301 ggcgagggag accatgacgg cccacagggc gggctggacg acgtcgtcgg cgctgtcgag
69361 ggcgggcgcg ccgggtgccc cgcgcaggac gtcttcgagg gaccagtcca cgtacggggc
69421 cagggcctcg ccgcaggcgc gcagccgctc gcggaagacg gccgacccgt cgaggagttc
69481 gacggccatg ccggcccatt gggagccctg gccgggggaag acgaacacga cgcggcggcc
69541 cacggttgcg gtgccctgga cggcgcgggg cgtggagccg ccctccgcca gcgccctcag
69601 tccttcggtc agttccgctt cggtgctgcc gacgacgacg gcgcggtgct cgaacgccgc
69661 gcgggtggtg gcgagcgagt ggccggtgtc ggcgggggcc gtgccggggt gggtcagggt
69721 gtgcgcgagg agggtgcggg cctgggcggc gagcgcctcg gcggtacggg ccgacagcgg
69781 ccacggcacg gggtcgcgt cggcggcggg ggtgcctgcg gccggggcgg tcggggcgtc
69841 ggcggtcggg gcgtcggcgg cctcgtgggc gtccggggcc tcttcgagga tggcgtgcgc
69901 gttggtgccg ctgatcccga acgaggacac gccggagcgg cgcgggcggc cggtgtcggg
69961 ccagtcggcc gcctcgccga gcagccgtac ccggccgtcg gcccagtcga tgtgcgggga
70021 cggcacgtcc gcgtgcaggg tcttgggcag gacgccgtgc tgcagggcca ggaccatctt
70081 cagcacgccc gcgatgccgg cggcggcctg ggtgtgcccg atgttcgact tcagcgagcc
70141 cagccacagc ggccggtcct cggtccggtc ccggccgtac gtggcgagga tcgcctgcgc
70201 ttcgatcggg tcgccgagtc gggtgccggt gccgtgcgcc tccaccgcgt ccacgtcggc
70261 cgccgacagg cccgcgttgg cgagcgcctg ccggatgacc cgctgctgcg cgggaccgct
70321 gggcgcggtg aggccgttgg aggcgccgtc ctggttgatc gccgaacccc gtacgacggc
70381 cagcacccgg tgcccgttgc ggcgggcgtc cgagagccgc tccagcagga ccatgccggt
70441 gccctcggcg aggccgaacc cgtcggcgtc ggaggagaag gccttggagc gcccgtccgg
70501 ggccagtgcc cgctgcttgc tgaactcgat gaacgacccg gccgacggca ggacggtgac
70561 gcccccgcc agcgccagcg agcactcgcc ggtgcgcagc gactggacgg ccaggtgcag
70621 ggcgaccagc gaggaggagc aggcggtgtc caccgtgacg gcgggaccct cgaacccgaa
70681 ggtgtaggcg aggcggccgg aggcgacgct ggcggtgttg ccggtcagca ggtagccgct
70741 gacgccctcg ggtgcctcgt acagccgggg cccgtagtcc tgggtcatgg ctccgacgaa
70801 ggtgccggta cgggtgccgc gcagcgtgcc ggggtcgatg cccgcgcgct ccagcacctc
70861 ccagccggtc tccaggagga gccgctgctg cgggtccatg gcgagggcct cgcgcgggga
70921 gatcccgaag aactccgggt cgaagtcggc ggccccgtgg agcagcgccg cctcgcgctg
70981 gtagtaccgg ccggggcggc cgggctccgg gtcgtagagg ccttcgacgt cccagccgcg
71041 gtcggcgggc agggcggaca cggcgtcgcc gccctcggtg agcacgtgcc agagggcttc
71101 gggggtgtcg atgccgccgg gcagccggca gctcatggcc acgatggcga cgggttcgtc
71161 ccaggcggcc gtggtggtgt cgtcggcggc ctcgtcccgg gtgccgaggg cgtccgcgac
71221 gatgcggccg gtcagcgacc gcggggtggg gtggtcgaag acgaccgtgt gcggcagcgc
71281 gagtccggtc gcctcgttca gccggttgcg cagttcgatg gcggtgacag agtccaggcc
71341 caggtcgcgg aaggcgtgtc cggcgccgac cccggccggg tcggacaggc cgagcagtgc
71401 ggcggtgacg gagcggacga gtgcgccgac ggtgcgctcg cgttcggcct cgggcagggc
71461 gcggagccgc tcggacagcg ccggaccgtc ggcggcggcg gtccgccgga aggggtacgt
71521 cggcagcggc ggccgggcgg ccggggcgcc ggggaacgcc cgctcccagt cgatgtgcac
71581 gccctgcacg taggcctcgg cgagggcggt gcggaagcgg cgcgggccgc cctggtcgcg
71641 gcggagggtg gtgacggtga cgggctgccg tccggcggcg gaggccgtct cctgcatcgc
71701 cgcggtgagg gacgcgtggg ggttgacctc gatgagggcc tggtggccgt cggcgaggag
71761 cgcgtgcgtg gtctgttcga accgtaccgg gcggcgcagg gcctcgaacc agaaggcggc
71821 gtccagggtg cggccgtcga cgatgccgcc ggtggcggag gagtagagcg gggtgtgggt
71881 ggcgcggggg gctatggagg cgagttcggc ggtcatccgg tcgcggaggt cctcgacgtg
71941 ggacgagtgc gcggcggctc cgacggcgac gccccggcag cggacgccgt ccgcctccag
72001 cgccgcgcgc agttcgcgca cggcatcggt gtcgccggag accacgacgg attcggggcc
72061 gttgtacgcg gcgacgtcga gccggccctc ccagggccgc aggcggtcgg ccagccggtg
72121 gccgggcagg gtcaccgcga gcatgtcgcc gccgcgggcc gcgagccgtg ccaggccgtc
72181 gccccaggag gtgatgaccc gggcggcgtc ccgcagggtc agcgcgccgg acaggtgggc
72241 ggccgcgatg tcgccgatgc tgtgtcccgc gaacgcggcg gggcaaactc cgtaggcctg
72301 ccaggtccgg gcgagggcga cctggacggc gaaggtggcg ggcatgctga cgtcgggggt
72361 gtcgagggcg gggcgcccg gctccccgcg caggacgtcg gtgagcagcc agccggtgag
72421 cggggccagg gcctcctggc attcgagggc cgcggcgtgg aactgcggtg aggagtcgag
72481 gagttcgacg gccatgccgg gccactgcga gccgatgccg gggaagacga acgcggtggg
72541 gccggtggcg gcggggccgg cgctgcctcg gacgatctcg gcggcgaagc cgccccgggc
72601 cagggtggcc agggcgtcga ggagctgtgc ggtgtcctcg ccgaggacga cggcgcggtg
72661 cgggaagcgg tgccgggtca cggcgagggc gcggcccacg gcggcggggt cgaggccggg

72721 gtgggcgctg aggtgggcgt gcagccgctc ggcctgggcg cgcagcgcct cggggccggc
72781 ggcggagagg atccagggca gggggtgttc gacggggccg gccccggaac cggccgggcc
72841 ggcgtccgcg gtgaggtctg ccccggaacc ggccggaccg gtgtcctcgg tgaggtcggc
72901 cccgggggcg gtgagccgtc cgtcggcgga gagcacggcc cgtacgcggt cgccgtcggc
72961 gagggcccgc gcaagggtct tgagcactac ggccccgtac cccccgacgc aaccacccgc
73021 gcccgggtcg gccccggcgg gctcggaccc agcggcctcc ggcccggcgg ccccggaccc
73081 ggcagccccg gacgggaagt ccgcgggcgc ggggtccgta tcggggaggc ggatgtcccg
73141 gcccgcggcc agggccaggg tggtttcgcc gcggcggagg cgctcgtggg ctgcccgcag
73201 ggcgtcggtg cccgaggggg tgctcaggac ctccgggtcg gcgccggggg cggtccgcag
73261 gacggcgcgg acggcggcgg ccgggtcgcc gtcctgcggg agcgcgtccg cgtccggtgc
73321 ggccaggaag acggcggtgc ccgcagccgg ctccgccgcc aggacgacgc ccgcgtcctc
73381 cagggcctgc caccacacct ccgccgcggc gggaccgtgg ggcagcgccc cggtgaggcg
73441 ggggtccgac gggcctgcgg tggcatccgc gaggaactcc gcgtcgaacg ccgggtcggc
73501 ttcgggcccg cccggcgggc ggtgcgccga tccggtcacc acgatcacgt cgtgcgtgct
73561 ggtcccggat tcttccggca ccgaaacaga ctcgcccata atgtgctcca tcgacccgtt
73621 gaactcatgc caatttgctg gttccgcagc gcatttcggc gtgcgggcat accggggaaa
73681 ccccgtgacg attcgcgctg tgatgaaccc ggacgggccg gtcccggtgc cgggtttacg
73741 acgccttgat gtcctgctgg atgacgcgct cgatgttccg ttccgcgagc gcggtgatgg
73801 tgacgaacgg attcacgccg atggagccgg gaatgagggc gccgtccgtc acgtagaggt
73861 tcttgtatcc ggacacccgg ccgtagtcat cggtcgcctt gcccagcacg cagccgccga
73921 gcgggtggta gcagaagtcg tccgagaacg ccttcagctg cttgccgaag aggtcgtagc
73981 ggtacatcgt cgtgttcgcc ctgttcaccc ggtcgaacag cgacttcgcg gcattgacgg
74041 cgggggtgtt ctggtcccgg gtccagcgca ggttcgcccg gtcgttcgcg gcgtcgtaga
74101 cgaaggcgcc gcgctcgggg ttcttggtga tcgccaggta gaggctgacc caggtctcca
74161 caccggccgg catcggggcg atctcggcga agacgggggc cgcggggttg tcccagtcgt
74221 cgatgcccag tgcggggatg gacgactggc tggcgccggt cgggttccac acgtggttgg
74281 cgcgggcggt catgatgttg ccgttcgggc cccagcccgc gccgatctcg gagctgaggt
74341 cgggcagcgt gccggtctcc cgggcgcgca gcagcagttc ggtggagccg aggctgccgg
74401 ccccgaggaa caggtggcgg caggagatct ccctggtggc gagcagcctg ccgtcggcgt
74461 ccttctgctc gacggtcagc aggtaggtcc cgtccttctg ctgccggatg gtcctggcct
74521 ggtggagggt ctcgatggtg accttgccgg tgcccagggc ggcggccagg taggtcttgt
74581 ccaggctctg cttgccgtgg ttgttgccgt agatcacctc tgcggccagc gcggacttgg
74641 ggacctcgcc gttcgcctcg cggcgcatgt agtcgaagtc gtagacgttg gggacgtgca
74701 cggtgcccag gccggccttc tgggcctggt cgcgggagac gcgggcgaac ttgtaccagt
74761 cggtgccctc gaaccacccg ttgtcgatgt ggttgacgcg gagcatggag ttggcgcgcg
74821 ggaagtagcg gtcgtacatc gcggcggagt cgacgcgcgg gagcacctcc tcgaagtacg
74881 agcgcttcgg ggccaccgcc atgccgccgt tgacgaggga gccgccgccg acgccgcggc
74941 ccacgtacac ggacatctgg tcgtagttga cccggtccag gacaccggcg taggggtcga
75001 tgtcccggtt ggcgaggtcg agccagagga agctgccgag cggcgcctcg gtgcgggtct
75061 tgaaccagct ggagcgcttg tcgggcttga gcatgcccga gaagacgttg ccgtcggggc
75121 cctgctggtt ccacagctgg cccatttcga gcatgacggt gcggacgccg gcctcaccga
75181 gccgcagggc ggtgacggcc gcgccgtagc ccgtcccgat gacgacggcg gggacgaagg
75241 ccggggtccc ggcgcggtcg gcggccaggg cccgggtggc gccgatggtg gtgctgcccg
75301 ccatggcggc ggcgccgagg gccgcgagtc cgagcatgcg gcgacgtgaa aggtgctgtt
75361 ccacggtgtg tacctggctt tcgggaaggg gtgtgagctg tggtgcgggg ggggagggcg
75421 ggtcgcgcgg cgcggcgggc ggggcgggac ctcagggggt cttggggacc tcagggggtc
75481 tcggggacct cggggacctc ggggacctcg ggcatcgggg agggcttgtg caggtgatc
75541 gccgcgcagg ggcactggtc ggcggcctcg cggacggcgg cgcgcagggc ggcgggcgga
75601 cgggcgtcga gcagcagcac gacgccttcg tcgctctggt cgaagacctc gggggcgagc
75661 agggtgcaca tgccggcgcc gcggcacagg tcccggctga cctcgatcct cagcgggtcg
75721 ccggtcacca ggcgacgggc agttcgtaca cgccgtagac ctgcatgtcg tggcggagcc
75781 tgatctcctc gggcgggcag gccaggcgca gtgccgggaa gcgctccagc agggcggtga
75841 agccgacgcg cagttcgatg cgggccagct gctgtccgag gcactggtgg atgccgtgcc
75901 cgaagccgag gtggccgccg gcgcgccggc tcacgtcgaa ggcctcgggg tcggggaacc
75961 gttcggggtc gcggttggcc gcggcgaggg agaccatcac gacctcgccc tccttgatga
76021 ggaccccgtc gatctcgacg tcctccaggg cggcccgcgt ggggccgtgg tggacgatgc
76081 tcaggtagcg caggctctcc tcgacggcgc cctcgatcag ggaggggtcg gcgcgcagcg
76141 cggcgagctg ttcggggtgg cgcagcagcg cgaaggtgcc gagggcgagc tggttcgcgg
76201 tggtctcgtg gcctgcgacg agcagcagga aggcgatcga ggtcagttcc tcgtcggtga
76261 ggtcggggtc ggcggcgatc agcccgctga ggatgtcgtc ggtgggctcg gcgcgcttga
76321 gccgcaccag gccctgcagg taggtgccca gcgagaggaa ggccgcggtc acgacctcgg
76381 tggactcccc gaagctgctc cagacgtgcg tgtcgtgctg gaaacccgca cggtcggcgt
76441 agggcacgcc gagcagttcg cagatgacca gggacgggat gggcagcgcg aagtgctcga

76501 agaggtcggc ctccgatccg aggcgttcca tggcgtcgag gcgttccgcg acgatgccct
76561 cgatccattc ggtgagcatc cgcatccggc ggacggtgaa ctgcccgacg aggagcttgc
76621 ggtagcgctg gtgctcgggg gcgtcgtgct ggatgaacat gccgggcggg gcggccgggg
76681 cctcctccag ggtcgcggcg cgcggtacgg ggggatgctt gagttccccc cgggcgctga
76741 accggttgtc ggcgaggacg gcccgcgcgg cggagtatcc ggtgacgagc cagccgaggt
76801 gcccgtcggc gaattccatc cgggcaatgg gttcggtccg gcgcagcgac tgcgtcatcg
76861 ggggcggatt gaagtatccg tctcggaccg tggaaaaaat caccggtgca ccctggctca
76921 tttccgccct ttcctgtcct gcgtgcattt attgaccgtg gcgggttccg ctggggcccg
76981 aggggaattg ggcatcgaaa tccggcctca cgacggcctc aattcggcct cggccctcgc
77041 agttcgatca ccgccaaaag gaaggtatga agacgcggtg aagggtgtca acgaaatcaa
77101 tccctggtgc actaaccctg tgggcgggga acggagggta cggttaccgc cggtaccgca
77161 ggtgtttcgg ttcggttcgg ttcggttcgg tgcctccccg acgccggggc accacgcccg
77221 cccggagcgc tacgggtgca ccgggcccgc cccgtcggag agcagccggg ccatcgcggc
77281 gagtccggta cggccgtcga cccgcagttt gcggtagacg tgggtgaggt gcttctcgac
77341 cgcgcgcgaa ctcacctcca gccgctcggc gatggccttg ttggaggcac cgcgggaggc
77401 gagcgcggcg acccggcgct ccgtgcgcgt cagcgtcgcc gccacgtcct cgccccggga
77461 accggcgatg ccctggagcg ccctgaccgc ccgctcgacc atccagggcg cgccgcagtc
77521 gagggcttcg gtacgggcgc gggtcagccg gtcctcggcc tcggcctggc gtccctgggc
77581 gagcagccgg cggccgagga ggagcgaggt acgggcccgt tccagggcgt tggccgatcc
77641 ctccagcgtg agcgcggact cccgcagcag cgcgatgccg cgcacgccct cggtgatggc
77701 gcccttgccg cgctgggccc ggccgatggc caccgggctg ccccagctca gggcgcgcgc
77761 gtactcctcc tccatcaggt cgtgggcccg gtcgggccgg cccatccggt agtgcagcgc
77821 ggacgcccag gtgcgccagg gcacgatggc cgggttgcgc cagtgggcgc gttcggccgc
77881 gcggccccat tcgaggaagc agtccagggc ggtggggagg tcgtcgcgca ggaccgcggc
77941 ggagccgtgg atgagccggc gcagggaggg ccggtggctc ggggcggctt cctccctgcg
78001 gccggacagc agccgttcgc acagggcggt gtcccgggcc tccagtgcgg ccagcacgac
78061 gggcgccagg gtctggagcg tggcccagtc ggtggcgtcc tgggcgagcg cctccgcggc
78121 gtgcgccgcg gctcccgtca ggtcgccgcg cgccagggcg aggcgggtga gttcgacggc
78181 gatgagggcg cgcggtacgt cggcgttctc cctgcgggcc cgttcgtacg cggtgtccag
78241 ccagggggccg atggtggcga gggagtcggc ggcgaccagg acgtcggtca gcagtggcag
78301 ggcggtgtgc acgtggtcgg tggcggcggg ctcgtgctgg agcagccgct ccgcgagcgc
78361 cacggcctcc gcggcgttga ccgtctgggc cagggcggct ccgtgcagca gtacggtgac
78421 gagttcgcgt tcggcggccg tgtccaggct gggggcgggg cccagggccc gcagcctggc
78481 gccgcaggcg gccggttcct cggggccggc cagcgccggg tgccgcagcc gtgcttccag
78541 gcgcagcgcg tgcccgcggg cggtgcccgt gagcgtcgcg ggctccccca gttccgccgc
78601 ggtcctgacc acggtgtcga cggtggtgga gggggcgccg tcgaggagga acggcgggat
78661 ccggcccgcc accggggccc gctggtcggt ctccgggaac aggaacaggg cctgggcgag
78721 gtgccgcagc gccgcgcggg gtgcgaaatc ccgttcgacc atggccagtt ccaccagcag
78781 ctcggcgcgg tccgggcctt cgggcgagct gccgagcagg gcgcggcgca gatagcgggc
78841 cgcggtgtcc ggggccgagc gccgcagggc cgaggccgcc gccgcccgca gtacgtcgct
78901 ctcccagtcc tcgtggcagg aggaggcggc gaggagctgg gcggcggccc gttccgccgg
78961 gtggccgccc aggtgcagca gcagggcggc gtgcacccgg atgctctccg cctcggccgg
79021 ggtcatggcc tcttcgacgg cttcgccgac caccgggtgg acgaagcgga gcgcggggcc
79081 ctcgcagacc aggccgaggc gtcggagcac tctgagggct tcgatgcggc cggtgccgtc
79141 gaggccggcg agtctgccga tcagttcctc ctccgcgccg tcgccgagga ccgcgagggc
79201 cgtggcgaac tcccgcaccg gcgcgggctg gtcgcgcagg acgacggcga accgttcgta
79261 cagggccgcc gggcgcaccg cgtggacggc ccggacgtcg tccgcggacc ccggcccttc
79321 cccgggcccg gagagcgcgt cgaggacggc ggtgagcagc aacggccgtc cgccggtcac
79381 ctcgtggcag gcccgggcgt acgccgggtc gacggggcgt cccgtcaggg cgccgatcag
79441 gtcggcggtg ccctcggggg gcagcgggcc cagccgcagg acgcgggagg ccccttcgac
79501 gagctgctgg aggcggggggt cgtcctcgcc ggggtcgcct tcgcgcaggg tgacgacgag
79561 ggtgacgcgc agcccggcca cgcgcccggc cagcaggccc agccagcgca gggaggcggc
79621 gtcggcccac tggaggtcgt cgaccagcat gagcagcggt gtgtgcgcgc cgtactcggc
79681 caccagcgcg aggaggccgt cgtcctggag gggccctgcc gacgggcccg cgcagtcgcc
79741 gggcggcgcg acgggggtgc ccggcagcag gggggccagc agctggccga gtacgcccgc
79801 ctcgaagtcg cgttcgaacg cggctccgct ggcggtgacg acgcgcaggc cgcggcgctc
79861 cgcccgtacc ggcagggccc gcagcagggc ggtcttgccg gtgcccaggc cgccggtgac
79921 gaccaccagc gagccgccgc cggagccgtg gcgggccgcg tccatcgcgc cggtgatcag
79981 gtcgagttcc ttctggcggt gcaacagggg cctcatcgtt cctccgtgtg ttctcggacg
80041 gcccggggag ggccgggtgt ggctcacgcg ccgggagccc cgcccgccgt gcgccgcagg
80101 gcggcggcca gctggggccg tccccggatg ccgagcttgc ggtagacctt ggtcaggtgg
80161 agctctacgg tccgggggt gacggccagt tcctcggcga ccgcccggtt gcccaggccg
80221 ccggcggcga gtgccgccac gcgctgctcg gcgcgggtca gcggtccccc accccctcgcg

80281 gtccggcccg gggccccgcc gcccgcgggc gtccgtacgg acgcgggcgg cggcggggag
80341 accgacggga accgggacgg cggcaggggc ccggacgcgg aacgggaatc ggacccggac
80401 gcggaccctg acgccgaacg ggacggaagc gggagtgcgg gggtcgcggg cctcggggcc
80461 gccgctgccc cccggtcgct gccgagcagg gagtcgacgg tgaggtggcc ggaggacggg
80521 gccgtgccga gggcgaccag cgcctgccgg tactgccagc tgaccggcct cccccggccc
80581 ggggcggggg tccccccggc cccggcggcc gcggccccct cccccagcgc cgcggccatg
80641 gcggcgggcg aaccccacag cagggcccgt cggcgttcct ccgcgagcag cgccgcggcc
80701 agcgggtctc cgggttcgcg ggcccgcgcc gcggccgaac gccacggcag cacggccggg
80761 ttgaccgcct gccgggccag cagctgccgc ccgcactcca gcaggtcggc gaaggcctgt
80821 tcgcgcgttc cgccggccag cgcgacccgg ccccgggcgt acagcaggta ggcccagggc
80881 agcccgccct cggcgccggg cggcagttcg gccgcgaggg cccgctcggc cgccgtccgg
80941 tcgccccgcc gcaggtgcag cagggcccgt agcgcgaggg gaccgggagc catcagcgga
81001 tgccagcagc ggcccggcat gatcccctcg caccggtcca gggcctcggc cgcggcggtg
81061 tcgcggccgg cccgcagctc ggcgaggcag gtgacgagca gggcgaggcc cgcgaccgcg
81121 cgtgcgtcgc tgcgttcggc ttgcaccagg acctcgtcga gcgcacggcg cgcctccgcg
81181 gtgtccccgg tgaggacgag cgcgtgcgcg gccgccgccc ggacggtgag cggaacgtgc
81241 tcgcggacct gcggggccag tgccgtccgg gcgagccgcc gggcccggtc gatgttgcgg
81301 ccgcgctgcg cggtgcgcca ggctgcgacg gccgcctcgg cggaccgttc cagcggacag
81361 gagtcgggca ggagcggctc gacgccgtcc gccgggtccg gccggctgta ctcggtgatc
81421 cagtgcaggg cccgcaggcc cgaccgctcc gcgctgcccg ggcggacggc gtcggcggcc
81481 cgggcgatgc ggggacgggg ggtgtcgacg tcgccgcggg ccacgaggag ttccgcggcg
81541 cgcagcaggg cgcggcccgt ctcggcgtcg gcggcggtgt cggcgacggc cgcggacccg
81601 tctgcggggg tctgcagggc gcggtacaga tggcggctcg cggcctcggg ttcggtgggc
81661 agcacggccg cgctcaggtc ggtcaggagc cggatccgca gggcgggcgg caccggctcg
81721 cgcagcgccc gttcgaggag ccgggcggcg cggccgggcc ggcccgtggc ccggcactcc
81781 tcggcctcgc ggcgcagggc ctgaaccgcc caggtggccc cgatccggcg ggcgtccagc
81841 agcatccggg ccagcgcgcg gtcaggcagg gccgcgcggt gggcccagtc ggcggcccgg
81901 gcgtggagtt cctcgcggtg ttcgctgccg agttcggcga ggacggcccg ggccgcggcg
81961 gcggtccgca acgcgggccg gggcccgggt gcgagcaggc cggtgccgcg cagcagggcc
82021 agggcccggg aggtaccgag ggcccgcggg ccggccagcg cgtcgatcag gccgggggtg
82081 cagtcgggcc cggcgaccgc gatggcgcgc aggaccccga ggagttcgtc gggcagcagg
82141 gccagggtcc gtacggtgcg ctcccgcacc gcctccgccg cgctctcctc cagccggccc
82201 gcggggccgg tgccgggcgt ccagccgtgc cggacgaacc gtccgacgat cgagcgcatc
82261 agggccgggc tgccctcggc gtgccggacc gcatcggcga ggaaaggctc gtcgaccggg
82321 gcccgccagg tcccggcgag gaactcgccg acggcgccgt ccggcagcgg tcccagggtc
82381 agcgcgtggt ggaccacggc gtcctcaccg gccagggcgc agacgtcgat ccgttcgtcc
82441 gcggtggccg ggacggcgct gttgcgggag gtgaggagca tgaggggcac cccgtcatc
82501 ctgcgggcca gtgaccgcag cccgcgcagc gactgcgggt cggcccactg gacgtcgtcg
82561 acgacgagca gcaggggcc gccggcggcg gcctccagga ccccggcgca caggtccgcc
82621 gtccgacggg gccgtactcc gtcggcgcgc ccggctccgg gtcgtgcggt gccgtacggc
82681 acggggccgg tccgggcggg cgcctcgggc agtcgtgcca gaagccgccc ggccacgccg
82741 tggggcagcc cggcctcctc cgccgtcgcc cggacagcgg cgacccgtac gcccgtgccg
82801 tgggccaggg ccgccgtgtg gtcgagcagg gcgctgcgtc cgatgccggg cggcccgtgc
82861 acctcgacga gtgccggccg tcccgtgcgc agctgcccca gcaggccgtg cagcaggccg
82921 gcctcggtgt cccgcccgac gagcgcggcc ggttccccg ccccgcccgg ccttgcgcgg
82981 tgcaccggtg gtgtggcgcg aaccggatgt gccgccgtcg tccgctccat gactgtccgt
83041 cctccagggg tgtcgggtcg atcggggctc ccccggctgc cgcctgggag cgctcccggc
83101 tgtgtgacgt cccccgggga tcaggacgtg gcggagaggc cgtccgccgg tctgggggcg
83161 ccggggtggc ggccgaggcc gcgcggcaga tcggcgcggc cgcgcacgtc gagcttccgg
83221 tagacgttgg tcaggtgcat ctccaccgtg cgcggggtga tgaacagcgc ttccgcgatc
83281 cccttgttgc tgtggccgcg ctcggcgagc gtcgcgatcc ggcgctcgct gtgggtgagg
83341 gagtcgaggg ggacggtcgc gagctgcggc atccgtcctc ccgcggtcac cagcgcgtcg
83401 cgggcgacgg cggcgagcac gcggtagccg cagcgggtgg cgaggccgat ggcccggcgc
83461 agatggcccc gggcgccgcg ggcgtcgccg agggcgagca gttcacggcc gagctggtat
83521 tcggccttgg cgagctccag ccgggccggg gactcggaca gcaggtcgac cgcccgggcg
83581 agcagtcccg tacggtcgcg gccttcgacg accgcggcct gcgcgaccag gcccaggccc
83641 agggcgcgcg gcgtgcccca gcggcgggcg cgttcccggg cggccgcgac gatgccggcg
83701 gcctcggcgc cccggcccag gccgccgagc gtggtggcgg tgggcagcca ccagggggcg
83761 agcaccgggt tggcgatgcc cgcctcctcc tcgctcctgc cgcagcggcg ccacagttcc
83821 agggcgcctt ccggatcgcc gagttcccgc aggatccggc ccttcgtgta caggtagtgc
83881 tgccattccc agacgctccg gtccaggtcc ggtgcggaac gggcgagcgc gtcgagcacg
83941 gcggccgcct gccgggcctc gccctgggag aggagtacgg agctcagcgc gatccgcgcg
84001 aacggtgggg ccccggccgg tccgacgacc tcgacggcgg cccaggcgtc ccgtgctgcg

84061 ccgggtacgt caccggtgcc gtggcggatc agggaccggc ccgccagcgc ggcgcggcgc
84121 ggccacaggg gcggctgggt ctcggccggg gacagcgcct ggtccagcgc cgtcagcgct
84181 tcgtcgacgg agtcggcgag tgcgaggacg gccgagcagc agaagacgcc ccagcccgcg
84241 gtgcccggct cgtcggcgcg cagggcctgc cgggcgagcg cggccgcccg gcccgcggat
84301 ccgccgtcca gggcggcgaa ggcgctgagg gccgcgagca gccgccggtc ggccggactg
84361 tggccgctgg gcacgggcag ggcggcggcc ctggcgcggg ccgcgggcag ggcgctccgg
84421 tcgttcaccg cggtggccag cagggcggat tcgacggtga cgcgcacttc gcggtcggcg
84481 ggcccggggc cggcacccgt accggagcac aggtcgccca gcacccgctc cagtaatcgc
84541 agtgcctcgg gggcccggcg ggtgccgagc gccatcatgg cgtactgcac cgcgacgggc
84601 gcccggtccc gcgggtgggg cagcagggcg agggcctggc gcaggtgcca cagcgcgtcc
84661 gcgggcgcga cgatggtgcg ggcggccgcg cgggccagtt cgacgtgcag gtccgcgcgt
84721 tctgcggtgg tgagccgacg gcccggggcg ccgcgcagcc agcggccggc ggccgcgacg
84781 gtcgcacggg tcggcgcttc ggcgagggct tcgcgcagga ccgcgaacat ccacggttcg
84841 tcgatccgct ccagcagggc gagctgctcg gcgacctcgg ccgcgggacg cccggcgtcg
84901 ctgaggaccc gggcggcccg cacgcgcagt ccccgcagct cttcgcagtc cagggcggcg
84961 aggaccgccg tacggatccc ctcgcgcatc cgcggcgggg agtcggccgg tacggcctcg
85021 atgtggcgca gccggcccag ggcggactcc acgtgccggg cgctcagccc gctgagcgcg
85081 gcgagcgggt cgacggcggc gcggccgagc acggcgaggg cggtggccac ctgccggacg
85141 gggccggggc cggcggcgag gaactcgggt accagggagg ccagtacggt ctggtggacg
85201 gtgctcagct ggtgcagggc gtcggcgccg ggtgggatgc ccacggtgct cagggtgtgg
85261 agcagtcggt cgaggagcag cgggttgccg ccggtgatct ccgtgcagcg ccggacgaac
85321 gccgggtcgg ccggggctcc gaggacacgg gtgatcattc gctgggtgtc gcgctcgtcc
85381 agcggtccga gttccaggag ggcgcagcgg tcctgggcga ggatctcggt gagcacccgg
85441 gtcccggggc ccgggacgcc ggtgcgctgg gccaggacga tcagcagggg ctggtcggcg
85501 gaccggcgca ggatgaagtc cgcgcagcgc agcgaggctt cgtcgcacca ctgggcgtcg
85561 tcgacgacca gggccgtggg accgtcggcg agcaggtcca gggcacgccg gtgcagccgg
85621 cgccagtcgc ggtgcgtgtc ccgcggctcg tgggcgccgc ccggtcgcgc gcagggggccc
85681 tcgggggggcg gttcgagcag ggcgtcgacc gcgcggatcc gggaggcacc tcccggaagg
85741 cccgcgacgc ttcgtacggc catgccctcg gccccgaggc gctcgctcgc gacggccagc
85801 agacggctcc tgccgatccc ggagggccct ctgaggagca cggcgcgcgc ggtgcccgcc
85861 ctggctcccg cggcgtggcg cagcaggtcg tccagtgcgg cggtgtgggt gtccaggctc
85921 cggcccgggc cgtgggcccg ggcagtgccg acggtgacca ccacagtatt tccctcttcc
85981 cctgaaccgt tcccggcgtc cggtgtcccc gacgccataa ccccccgcac aaacaagatc
86041 gtaggcgaca ccaagggaga actcggaaga gatccggcag atttgagcca gaatgcactg
86101 atccttggcc aaggcgcgcc gcgcgcctcc agtgggccgc cgtaaaggac gcatcatttc
86161 ggccgttccg ctcggccgaa ttcccggacc cccgaagaaa cggcgggatc cgtacgaccc
86221 cggacggcgg ggacgggccg atatccggcc atcaattcct tctttatgcc ctgcgctatc
86281 gtgcccgaac aaccgtccgc cgatgccgag agaccggagg gtcccgtgca gtcctccctg
86341 gagctcgcct gcccgccacc gggctgggcc gtctcggact ggcccacgac gcgtcccccg
86401 ccggacgagg gatcgctgga cctgtggctg ctgcacgtct ccgacgcggg gctgcgctcc
86461 gccgtcctga ccggtccccc ctggacgcgg aggaacgcgc gcgggtcgcg gccttcgtcc
86521 accccgccga ccgcgaccgc tacgtggccg cgcacctgac cctgcgccgg gtgctctccg
86581 cccaccgggg cgtcgccccg gcggagctga ggttcgcgcg ccgtgcctgc ccgtgctgcg
86641 ttggaccgca cggccgtccc gtcctggccg gatccggcac tccgcacttc tcgatgtccc
86701 acaccggcgg tctggtactg atcgccgtgg ccggacggcc cgtcggcgtc gacgtcgagc
86761 ggcttcccgc gccacacaag gccaccacgg gccccgagct cgcggtccac tcccacttcc
86821 tgggatcact cggccggaac tcggcctgac gccgggcgga ccccgaccgg aacggatttc
86881 acccgaaccc ttcccgcaca agacacctcc gcgaaaagcc gaatgaccgg gggtgaactc
86941 gcggacttcc ggatccggcc cggaagctcc ttcacccccg atgcggcccg gtccgaagcc
87001 ttttcggctt ccgatccggc ccgcggggat cagcggatcg ggtcggccgg aaaggccgaa
87061 tcgaatacgg ttttccgccc gcgcgacttc ggtggccgaa gacggcgaaa ggccgccctc
87121 ccccgaagcg gccggtctcc ggggcagcac gggccccgac acacccgtcc ccttccggcc
87181 tctggccgac cacacccccg caggcccgcg tggcgccatc cggcgccaca tggcaccaca
87241 tggcgccatc cggcacaccg gcagcccatg gacgcggatg gcaccacgcg acaccccctg
87301 aacaccatca cgttttccca ggtcagaggg tcatcactca gaagccctcc cagaaacacc
87361 cccacccccc ttgcgcatgg caccactatg gccccatgat ggcgccatgg acctcactcc
87421 gtatgtcgac accctccgcc gcgaactggc ggtggccgcc gaagccggcg gcgacgaagc
87481 ccgagagctg gccgagagac tcaccgctcc cctggaatcg gcgacccggc tgaccatgct
87541 ccacgtgctc tccgccgcga tggacgagat cacccgcgaa ctcgcccccg gctcggtcga
87601 cgtacgactg cgcggcctgg accccgactt cgtggtgacg ctgccgcccg ccgacaccgg
87661 cgcctcggag gcggcctccg cgcccgtcga accggtcggc gcccaggtgc ccgccgaggg
87721 cgacgagacc ggaaccgccc gcgtcaacct gcgcctgccc gcccacctca aggcccgggc
87781 cgaggaggcc gcgggccgcg agggcctgtc ggtaaacgcg tggctggtgc gggccgtgtc

```
87841 ggcggccgtc gagggcggca cccggccgca caccacgcag aagtcccaga ccgtcggaca
87901 gagcttcacg ggctgggtcc gctagccgcg cccaccctcc gcgcctccac ccactccact
87961 tcactccgct ccactccacc cgcaccacct cacccacacc gcgtcccacc tgccgggacg
88021 tccagaagag ccaagaggac gggacagcca tgccttcttt cgacactccc gaaccgatcg
88081 cggtcaccgc acgcgtggag gccggttcca tccagttcac cgcgggcgac cgccccgaca
88141 cggtcgtcga ggtgcggccc cgcgacccga agcgggacct ggacgtccgg accgccgacc
88201 agacggaggt cacgtacacc ggcggcgtgc tgaccatcag gacacccaag tccaacctcc
88261 tcggccgtac cggaaccgtc gatctgacgg tcgaactccc cacgggctcg cgcatcgaca
88321 cgacgggggc ctggacccag gtggtcggcg agggccggct cggtgaggtc cgcgtgaaga
88381 cctcgtcggg cgacgtgcgc ctcgacacga ccggaccact gaagctgacc gcctcgcacg
88441 gctcgatcac cgtggaccgg atcgagggca cggccgagat caccaccagc tccggcagcc
88501 tgcgcgtcgg cgtcgtcgac ggccccgccg tcctgaagaa ctcgcacggc accacgaccg
88561 tcggcgccgc caccggcgag atccgggtga gcggcgccaa cggcgacatc gagatccgcc
88621 gggccgaggg cccggtcacc gccaccaccg cccacggcac cctgcgcttg ggtgaagtga
88681 cgcgcggcac cgtccagctg gagacctcct acggcgccat cgaggtcggc gtccgcgagg
88741 gcacggccgc ctggctcgac gtcaactcgg gccacggcca ggtgcgcaac tcgctcaccg
88801 cgtccgagac cccggggag gccgaggaca ccgtgaagat ccgcgcccgc acccggtacg
88861 gcaacatcga cgtccgccgc gccaaggcct gacaccagga cctgacgccc ggagcgcggg
88921 ccccggcaca cggctcacgt ctcacgtctc acccgcccct ccatccattc cggctctcga
88981 aacgggaggg caccatgcct tcttctgtca tgcccacctc aaaccacaac gacggtcacc
89041 cgtcacaggt cgccatctcc gccgccgggc tgcgcaagtc ctacggcgac aagaccgtcc
89101 tggacggcat cgacctgaac atcccggccg ggtcggtgtt cgccctgctc gggccgaacg
89161 gcgcgggcaa gaccaccgcc gtgaagatcc tctccaccct catcaccgcc gacgccgggc
89221 aggcccgggt cgggggccac ggcctgacca ccaaccccca ggccgtccgc gccgccatcg
89281 gtgtcaccgg ccagttctcc gccgtcgacg ggctgatcac cggcgaggag aacatgctcc
89341 tcatggcgga cctccaccac ctgcccaagc ccgagggccg gcgggtcgcc gccgagctcc
89401 tggaacgctt cgacctcacc gccgccgcga agaagcccgc ctccacctac tcaggcggca
89461 tgaaacgccg cctggacatc gccatgaccc tggtcggcag cccccgcatc atcttcctcg
89521 acgaacccac caccggcctc gacccccgct cccgccacac catgtggcag atcatccgcg
89581 aactcgtcac cggcggcgtc accgtcttcc tcaccaccca gtacctcgaa gaagccgacc
89641 agctcgccga ccgcatcgcc gtcctcaaca acggcaagat cgccgccgaa ggcaccgccg
89701 agcagctcaa acgcctcgtc cccggcggac acgtacgcct gcgcttcacc gaccccaccg
89761 cctaccgcac cgccacccac accctgccca ccgccaccgg ggacaacgag tccctcaccc
89821 tccagatccc cagcggcgcc acccagcacg aactgcgcac cctcctcgac cacctccaca
89881 ccaccggcat cgaagccgac gaactcaccg tccacacccc cgacctcgac gacgtcttct
89941 tc//
```

Fig. S49. Nucleotide sequence of the *stv* BGC from the *S. lavenduale* subsp. *lavendulae* CCM 3239 with the annotation of all genes.

References

Aparicio JF, Barreales EG, Payero TD, Vicente CM, de Pedro A, Santos-Aberturas J (2016) Biotechnological production and application of the antibiotic pimaricin: biosynthesis and its regulation. Appl Microbiol Biotechnol 100:61-78.

Blin, K., Shaw, S., Augustijn, H. E., Reitz, Z. L., Biermann, F., Alanjary, M. et al. (2023) antiSMASH 7.0: new and improved predictions for detection, regulation, chemical structures and visualisation. *Nucleic Acids Res.* **51,** W46-W50.

Bortolo R, Spura S, Guglielmetti G, Cassani G (1993) AB023, novel polyene antibiotics II. Isolation and structure determination. J Antibiot 46:255-264.

Brautaset T, Sletta H, Nedal A, Borgos SE, Degnes KF, Bakke I, Volokhan O, Sekurova ON, Treshalin ID, Mirchink EP, Dikiy A, Ellingsen TE, Zotchev SB (2008) Improved antifungal polyene macrolides via engineering of the nystatin biosynthetic genes in *Streptomyces noursei*. Chem Biol 15:1198–1206.

Busche T, Novakova R, Al'Dilaimi A, Homerova D, Feckova L, Rezuchova B, Mingyar E, Csolleiova D, Bekeova C, Winkler A, Sevcikova B, Kalinowski J, Kormanec J (2018) Complete genome sequence of *Streptomyces lavendulae* subsp. *lavendulae* CCM 3239 (formerly "*Streptomyces aureofaciens* CCM 3239"), a producer of the angucycline-type antibiotic auricin. Genome Announc 6:e00103-18.

Carmody M, Byrne B, Murphy B, Breen C, Lynch S, Flood E, Finnan S, Caffrey P (2004) Analysis and manipulation of amphotericin biosynthetic genes by means of modified phage KC515 transduction techniques. Gene 343:107–115.

Carmody M, Murphy B, Byrne B, Power P, Rai D, Rawlings B, Caffrey P (2005). Biosynthesis of amphotericin derivatives lacking exocyclic carboxyl groups. J Biol Chem 280:34420–34426.

Chen S, Mao X, Shen Y, Zhou Y, Li J, Wang L, Tao X, Yang L, Wang Y, Zhou X (2009) Tailoring the P450 monooxygenase gene for FR-008/candicidin biosynthesis. Appl Environ Microbiol 75:1778–1781.

Csolleiova D, Knirschova R, Rezuchova D, Homerova D, Sevcikova B, Matulova M, Núñez LE, Novakova R, Feckova L, Javorova R, Cortés J, Kormanec J (2021) An efficient system for stable markerless integration of large biosynthetic gene clusters into *Streptomyces* chromosomes. Appl Microbiol Biotechnol 105:2123-2137.

De Schrijver A, De Mot R (1999) A subfamily of MalT-related ATP dependent regulators in the LuxR family. Microbiology 145:1287–1288.

Gust B, Challis GL, Fowler K, Kieser T & Chater KF (2003) PCR-targeted *Streptomyces* gene replacement identifies a protein domain needed for biosynthesis of the sesquiterpene soil odor geosmin. Proc Natl Acad Sci USA 18:1541-1548.

Javorova R, Rezuchova B, Feckova L, Novakova R, Csolleiova D, Kopacova M, Patoprsty V, Opaterny F, Sevcikova B, Kormanec J (2024) A new synthetic biology system for investigating the biosynthesis of antibiotics and other secondary metabolites in streptomycetes. J Biotech 392:128-138.

Kakavas SJ, Katz L, Stassi D (1997) Identification and characterization of the middamycin polyketide synthase genes from *Streptomyces caelestis.* J Bacteril 179:7515-7522.

Keatinge-Clay AT (2007) A tylosin ketoreductase revealed how chirality is determined in polyketides. Chem Biol 14:898-906.

Keatinge-Clay AT (2008) Crystal structure of the erythromycin polyketide synthase dehydratase. J Mol Biol 384:941-953.

Keatinge-Clay AT (2012) The structures of type I polyketide synthases. Nat Prod Rep 29:1050.

Kieser T, Bibb MJ, Buttner MJ, Chater KF, Hopwood DA (2000) Practical S*treptomyces* genetics. The John Innes Foundation, Norwich

Kormanec J, Sevcikova B (2000) Identification and transcriptional analysis of a cold shock inducible gene, *cspA,* in *Streptomyces coelicolor* A3(2). Mol Gen Genet, 264:251–256.

Myronovskyi M, Welle E, Fedorenko V, Luzhetskyy A (2011) β-glucuronidase as a sensitive and versatile reporter in actinomycetes. Appl. Environ Microbiol 77, 5370-5383.

Novakova R, Odnogova Z, Kutas P, Feckova L, Kormanec J (2010) Identification and characterization of an indigoidine-like gene for a blue pigment biosynthesis in *Streptomyces aureofaciens* CCM3239. Folia Microbiol 55:119-125.

Qi Z, Kang Q, Jiang C, Han M, Bai L (2015) Engineered biosynthesis of pimaricin derivatives with improved antifungal activity and reduced cytotoxicity. Appl Microbiol Biotechnol 99:6745-6752.

Reid R, Piagentini M, Rodriguez E, Ashley G, Viswanathan N, Carney J, Santi DV, Hutchinson CR, McDaniel R (2003) A model of structure and catalysis for ketoreductase domains in modular polyketide synthases. Biochemistry 42:72-79.

Schlingmann G, Milne L, Borders DB, Carter GT (1999) Strevertenes, antifungal pentaene macrolides produced by *Streptoverticillcum* LL-30F848. Tetrahedron 55:5977-5990.

Sekurova O, Brautaset T, Sletta H, Borgos SE, Jakobsen OM, Ellingsen TE, Strom AR, Valla S, Zotchev SB (2004) In vivo analysis of the regulatory genes in the nystatin biosynthetic gene cluster of *Streptomyces noursei* ATCC 11455 reveals their differential control over antibiotic biosynthesis. J Bacteriol 186:1345–1354.

Walker JE, Saraste M, Runswick MJ, Gay NJ (1982) Distantly related sequences in the alpha- and beta-subunits of ATP synthase, myosin, kinases and other ATP-requiring enzymes and a common nucleotide binding fold. EMBO J 1:945–951.

Yadav G, Gokhale RS, Mohanty D (2003) Computational approach for prediction of domain organization and substrate specificity of modular polyketide synthases. J Mol Biol 328:335-363.

Yoshioka T, Igarashi Y, Namba T, Ueda S, Pait IGU, Nihira T, Kitani S. (2021) Lavencidin, a polyene macrolide antibiotic from *Streptomyces lavendulae* FRI-5. J Antibiot 74:359-362.

Zhu Y, Xu W, Zhang J, Zhang P, Zhao Z, Sheng D, Ma W, Zhang Y-Z, Bai L, Pang X (2020) A hierarchical network of four regulatory genes controlling production of the polyene antibiotic candicidin in *Streptomyces* sp. FR-008. Appl Environ Microbiol 86:e00055-20.
